# Supplementary material for: AltaiR: a C toolkit for alignment-free and temporal analysis of multi-FASTA data
Source: Gigascience. 2024 Nov 26;13:giae086. doi: 10.1093/gigascience/giae086 (PMC11590114; doi:10.1093/gigascience/giae086)
Supplement: giae086_Supplementary_Files [file giae086_supplementary_files.zip › AltaiR_Supplementary_Material (1).pdf]

Supplementary material of
"AltaiR: a C toolkit for alignment-free and spatial-temporal analysis of multi-FASTA data"

J. M. Silva, A. J. Pinho, and D. Pratas

1 NCD Supplementary Material

1.1 NCD profiles on synthetic data

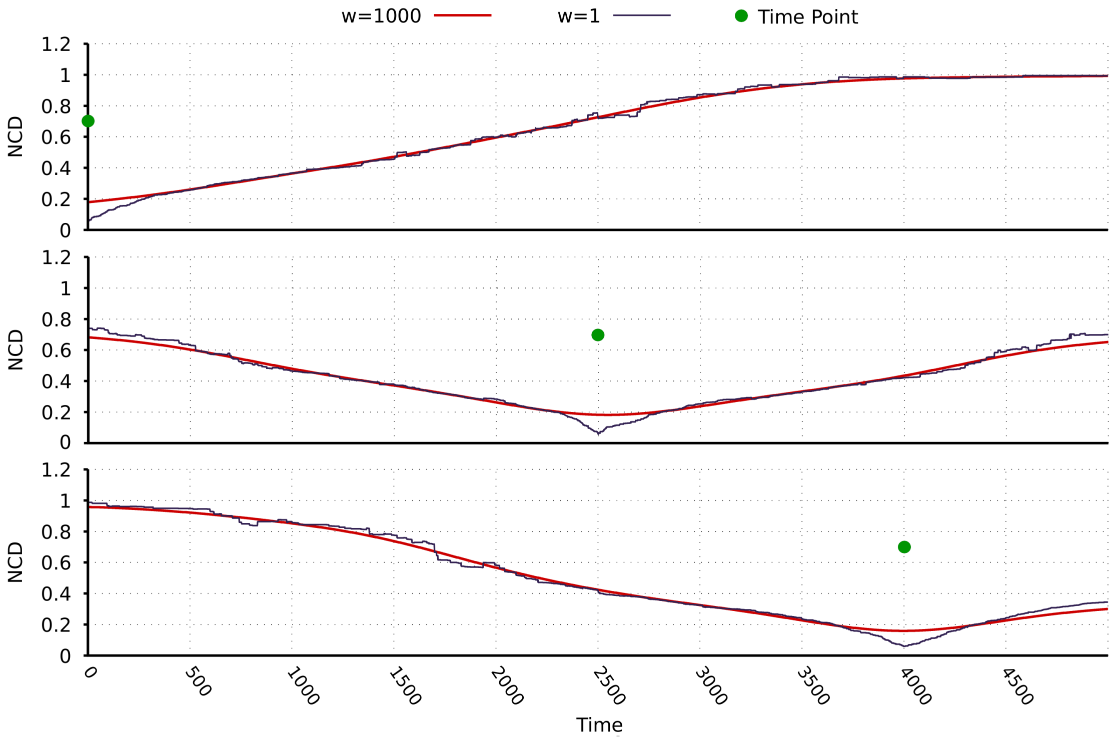

Figure S1: NCD profiles for different time points (from top to down sub-figures: 0, 2500, and 4000) using simulated data with recursive SNP mutations along the time. A time point represents a specific genome sequence at a particular point in the simulated mutation process. The plots are aligned by time (x-axis). The w stands for the size of the filtering window. Lower NCD values represent higher similarity to the chosen time point.

1.2 NCD profiles application - RaTG13 analysis

In addition to the synthetic data analysis presented in the main manuscript, we also explored the application of NCD profiles to a real-world dataset, specifically focusing on the RaTG13 genome sequence and its similarity to SARS-CoV-2 sequences. The public information regarding the RaTG13 shows the isolation of this genome in a Chinese colt mine in 2013. The RaTG13 genome (MN996532.1) contains the most similar known sequence regarding the genomes of SARS-CoV-2. Although the isolation has been described as 2013, several questions emerged, namely if this sequence was from 2013, it was the exact output reconstruction of multiple sequences, or if the reads had low-depth, high diversity and were reconstructed using the SARS-CoV-2 reference. Therefore,

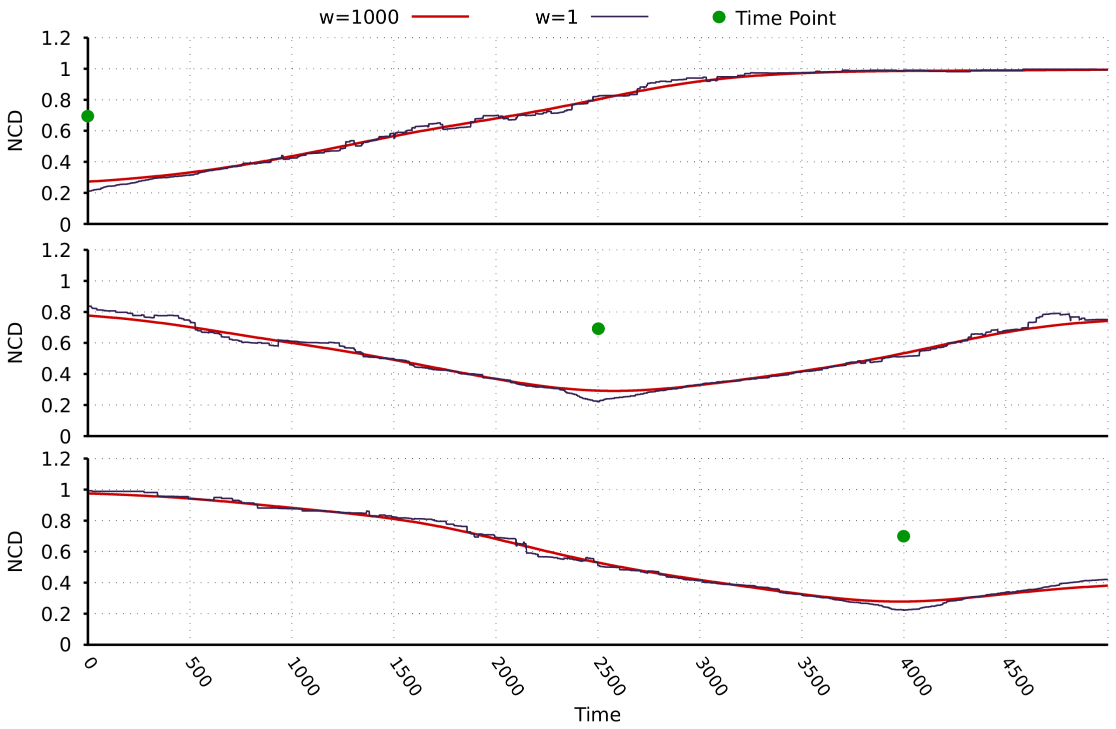

Figure S2: NCD profiles for different time points (from top to down sub-figures: 0, 2500, and 4000) using simulated data with recursive SNP mutations along the time. Each selected time point (green) has been additionally mutated to resemble more with real applications. The plots are aligned by time (x-axis). The w stands for the size of the filtering window. Lower NCD values represent higher similarity to the chosen time point.

we use the NCD profiles to estimate the temporal origin of this sequence. For example, if it is more similar to a SARS-CoV-2 point, the origin is unlikely to be before 2019 (assuming a careful genome reconstruction).

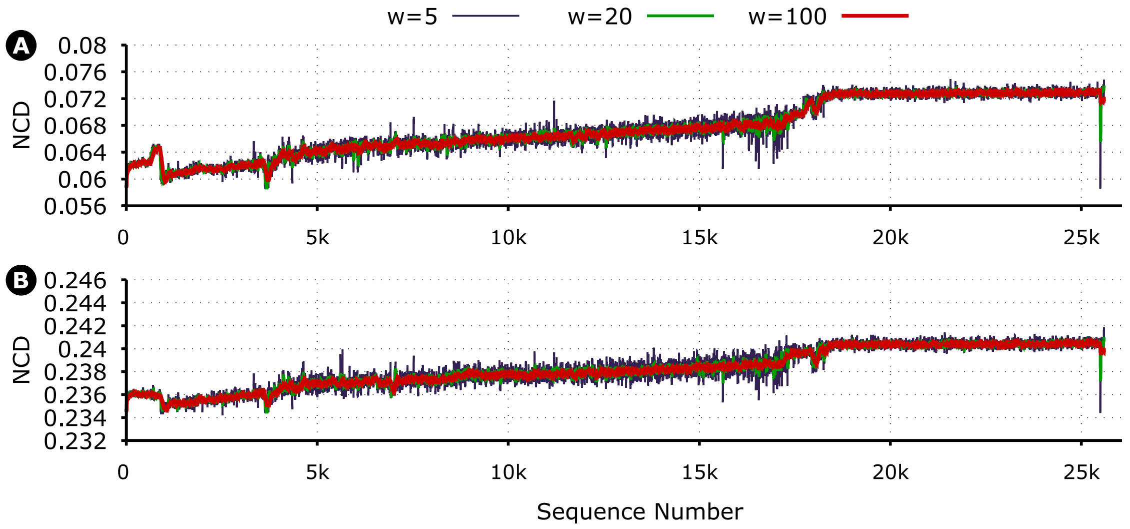

Figure S3: Similarity (NCD) profiles using (A) the SARS-CoV-2 reference genome sequences and (B) the RaTG13 sequence genome. The 'w' represents the average moving window size for 5, 20, and 100. To replicate this analysis, see Supplementary Section Reproducibility, specifically Reproducing the "Similarity (NCD) profiles application"

Figure 3 depicts the temporal similarity profiles of the filtered SARS-CoV-2 genomes according to the RaTG13 sequence, identifying an apparent higher similarity (lower NCD) to the first genomes being isolated and, hence, reinforcing its probable origin before 2019. The SARS-CoV-2 genomes show a continuous evolution with the changes in the genetic code caused by mutations or recombination occurring during genome replication,

thus providing an incremental dissimilarity over time. Despite this similarity, we must consider that the RaTG13 is only one genome, unlike the SARS-CoV-2. The SARS-CoV-2 genomes are approximately 117k. The existence of multiple SARS-CoV-2 genomes, originating from various sources and generated using different techniques, makes it challenging to draw definitive conclusions about the temporal origin of RaTG13 based on its similarity to SARS-CoV-2 sequences alone. Therefore, we highlight the importance of verifying the reconstruction process of RaTG13, namely if it used reference-based assembly (using the SARS-CoV-2 as reference) or de-novo assembly and the diversity load in the samples (High contamination? High diversity of coronaviruses genomes?).

It is important to note that NCD is not equivalent to a molecular clock, and without proper calibration using sequences with known temporal origins, inferring precise temporal relationships based solely on NCD profiles may be limited. The observed similarity patterns could be influenced by various factors, such as the reconstruction process used for RaTG13 and the diversity of the samples used. Further investigation, including the incorporation of additional data points and the use of complementary methods, would be necessary to draw more definitive conclusions about the temporal origin of RaTG13.

Despite these limitations, the NCD profile analysis of RaTG13 demonstrates the potential utility of this tool in exploring similarity patterns between sequences and generating hypotheses for further study. As more data becomes available and the methodology is refined, NCD profiles may contribute to our understanding of the evolutionary relationships between viral genomes and aid in the identification of potential temporal associations.

2 RAWs Supplementary Material

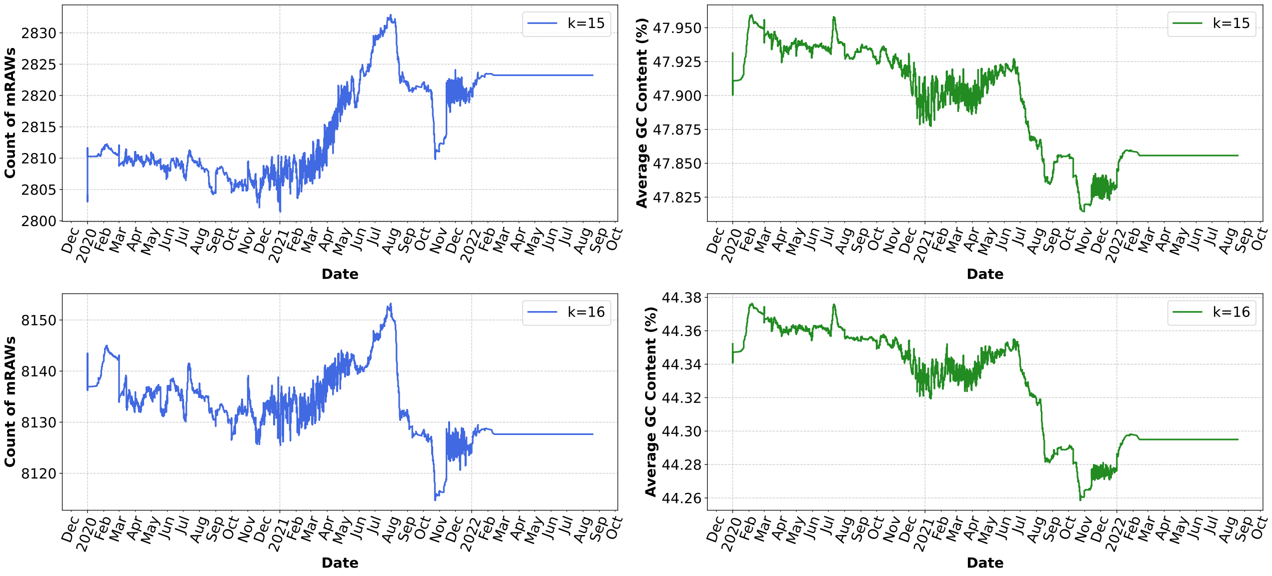

Figure S4: Relative singularity profiles: This set of plots illustrates the variation over time in the number of mRAWs and the average GC content in SARS-CoV-2 sequences. Each subplot corresponds to a different k-mer size (k=15 and 16), showcasing the distribution of mRAWs and GC percentage across various time points. The x-axis represents time, segmented into years and months, while the y-axis shows the count of mRAWs and the GC content percentage, respectively.

3 Reproducibility

3.1 Installation

To install AltaiR in a dedicated environment using Bioconda [1], it is recommended to create a new Conda environment and then install AltaiR. This can be achieved with the following command:

```
mamba create -n altair -c conda-forge -c bioconda altair-mf -y
```

Alternatively, for a direct installation in an existing environment, use:

```
conda install -c bioconda altair-mf -y
```

For manual installation or when reproducing experiments as described in this article, the code available in the repository can be used:

```
git clone https://github.com/cobilab/altair.git
cd altair/src/
cmake .
make
```

Please use the manual compilation and installation method to ensure the reproducibility of the experiments described in the article.

Furthermore, for some scripts, you will require gto installation. This can be achieved by conda installation:

```
conda install -c cobilab gto --yes
```

Or manual installation:

```
git clone https://github.com/cobilab/gto.git
cd gto/src/
make
export PATH="$HOME/gto/bin:$PATH" #make gto executables temporarily runnable from any folder
```

3.2 Requirements

To install the required Python packages for this project, follow these steps:

Ensure Python 3 and pip are installed on your system. Optionally, create and activate a virtual environment to avoid package conflicts:

```
python -m venv venv
# On Windows
.\venv\Scripts\activate
# On Unix or MacOS
source venv/bin/activate
```

Install the packages from the `requirements.txt` file:

```
pip install -r requirements.txt
```

After following these steps, all necessary dependencies will be installed in your Python environment.

3.3 Reproducing the "Filtering sequences"

These instructions assume that the code has been compiled in the alternative way under the src/ folder. Then, in the pipelines/ folder of the AltaiR repository, please type

```
cp ../src/AltaiR .   # Considering the alternative installation
python3 Histogram.py    # Computes the histogram
```

The output is provided in the same folder, including the Histogram.pdf file with the results image.

Then, to filter the data according to the most representative data length cluster and reference genome length (NC_045512.2), the following script must run

```
bash Filter.sh 29885 29921  # Filters sequences by interval length and patterns
```

This filtering approach considered only the sequence length (only ACGT) from a minimum size of 29,885 to 29,921. This approach filtered the results to 25,594 SARS-CoV-2 genomes.

After filtering, you need to order the sequences by date of sequencing. This is done by the information in the header of each sequence in the multi-fasta. This process is followed by a script that removes any strange characters from the genomic sequences file and can be achieved by running the script:

```
 python3 OrderSequences.py FIL-SARS-CoV-2.fa sorted_output.fasta
 python3 ProcessGenome.py sorted_output.fasta sorted_output.fa
```

Finally to compare variants before and after the filtering run:

```
python filtered_vs_unfiltered.py
```

3.4 Reproducing the "Similarity (NCD) profiles application"

These instructions assume the code has been compiled in the alternative way under the src/ folder. Then, in the pipelines/ folder of the AltaiR repository, please type

```
cp ../src/AltaiR .                          # considering the alternative installation
bash Simulation.sh                          # Simulates consecutive SNP mutations
bash Similarity.sh ORIGINAL.fa              # Measures similarity
bash SimProfile.sh sim-data.csv 2 0 1.2     # Plots the measured similarity
mv NCDProfilesim-data.csv.pdf NCD_P1.pdf    # Change name to better distinguish
```

This will simulate temporal data using the ORIGINAL.fa as a starting point and apply a consecutive SNP mutation (edition nature) with a probability of 0.001 of a particular symbol to be mutated. Then, the similarity of all time points is computed to the first point (ORIGINAL.fa), and the profile is saved as NCD_p1.pdf. To perform the same experience to the 2500 time point, please type:

```
bash ExtractPoint.sh 2500 > POINT.fa        # Extract point 2500
bash Similarity.sh POINT.fa                 # Similarity to point 2500
bash SimProfile.sh sim-data.csv 2 0 1.2     # Plots the measured similarity
mv NCDProfilesim-data.csv.pdf NCD_P2500.pdf # Change name to better distinguish
```

An identical experience is performed for a time point 4000 as follows

```
bash ExtractPoint.sh 4000 > POINT.fa        # Extract point 4000
bash Similarity.sh POINT.fa                 # Similarity to point 4000
bash SimProfile.sh sim-data.csv 2 0 1.2     # Plots the measured similarity
mv NCDProfilesim-data.csv.pdf NCD_P4000.pdf # Change name to better distinguish
```

Other experiences can be performed using different time points, SNP rates, or original sequences.

An identical experience is performed below, but instead, with the time points mutated with a mutation probability of 0.05 in each sequence symbol.

```
cp ORIGINAL.fa P0.fa                        # Use first time point as original
bash ExtractPoint.sh 2500 > P2500.fa        # Extract sequence time point 2500
bash ExtractPoint.sh 4000 > P4000.fa        # Extract sequence time point 4000
#
bash Mutate.sh P0.fa 71231 0.05 > P0_MUTATED.fa        # SNP mutation with 0.05 probability
bash Mutate.sh P2500.fa 71231 0.05 > P2500_MUTATED.fa  # SNP mutation with 0.05 probability
bash Mutate.sh P4000.fa 71231 0.05 > P4000_MUTATED.fa  # SNP mutation with 0.05 probability
#
bash Similarity.sh P0_MUTATED.fa                # Similarity to point 0 mutated
bash SimProfile.sh sim-data.csv 2 0 1.2         # Plots the measured similarity
mv NCDProfilesim-data.csv.pdf NCD_P0_M.pdf      # Change name to better distinguish
#
bash Similarity.sh P2500_MUTATED.fa             # Similarity to point 2500 mutated
bash SimProfile.sh sim-data.csv 2 0 1.2         # Plots the measured similarity
mv NCDProfilesim-data.csv.pdf NCD_P2500_M.pdf   # Change name to better distinguish
#
bash Similarity.sh P4000_MUTATED.fa             # Similarity to point 4000 mutated
bash SimProfile.sh sim-data.csv 2 0 1.2         # Plots the measured similarity
mv NCDProfilesim-data.csv.pdf NCD_P4000_M.pdf   # Change name to better distinguish
```

The example with the natural sequences containing the SARS-CoV-2 reference similarity to the SARS-CoV-2 genomes requires the following commands:

```
bash SimilaritySars.sh SARS-CoV-2-REF.fa               # Similarity to SARS-CoV-2 reference
bash SimProfileSars.sh sim-data.csv 0.004 0.056 0.080  # Similarity plots
mv NCDProfilesim-data.csv.pdf NCD_SARS2_REF.pdf        # Change name to better distinguish
```

The example with the natural sequences, containing the RaTG13 similarity to the SARS-CoV-2 genomes, requires the following commands

```
bash SimilaritySars.sh RATG13.fa                       # Similarity to RaTG13.fa
bash SimProfileSars.sh sim-data.csv 0.002 0.232 0.246  # Similarity plots
mv NCDProfilesim-data.csv.pdf NCD_RATG13.pdf           # Change name to better distinguish
```

Phylogenetic Tree Construction Script

Using the similarity file generated by AltaiR, it is possible to construct a phylogenetic tree based on Normalized Compression Distance (NCD) values. The script reads data from a specified file, computes a distance matrix, and then uses the Neighbor-Joining (NJ) algorithm to build the tree. The tree is visualized and saved as a PDF file.

To execute the script, use the following command in the terminal:

```
    python3 tree.py sim-data.csv -N 50
```

Here, `sim-data.csv` is the path to the input data file, and `-N 50` is an optional argument that specifies the number of closest nodes to be used in the tree construction. If this argument is not provided, the script will use all available data points in the file.

The output image will be similar to Figure 5, where in each leaf the sequence number, the location and date of sequencing will be present.

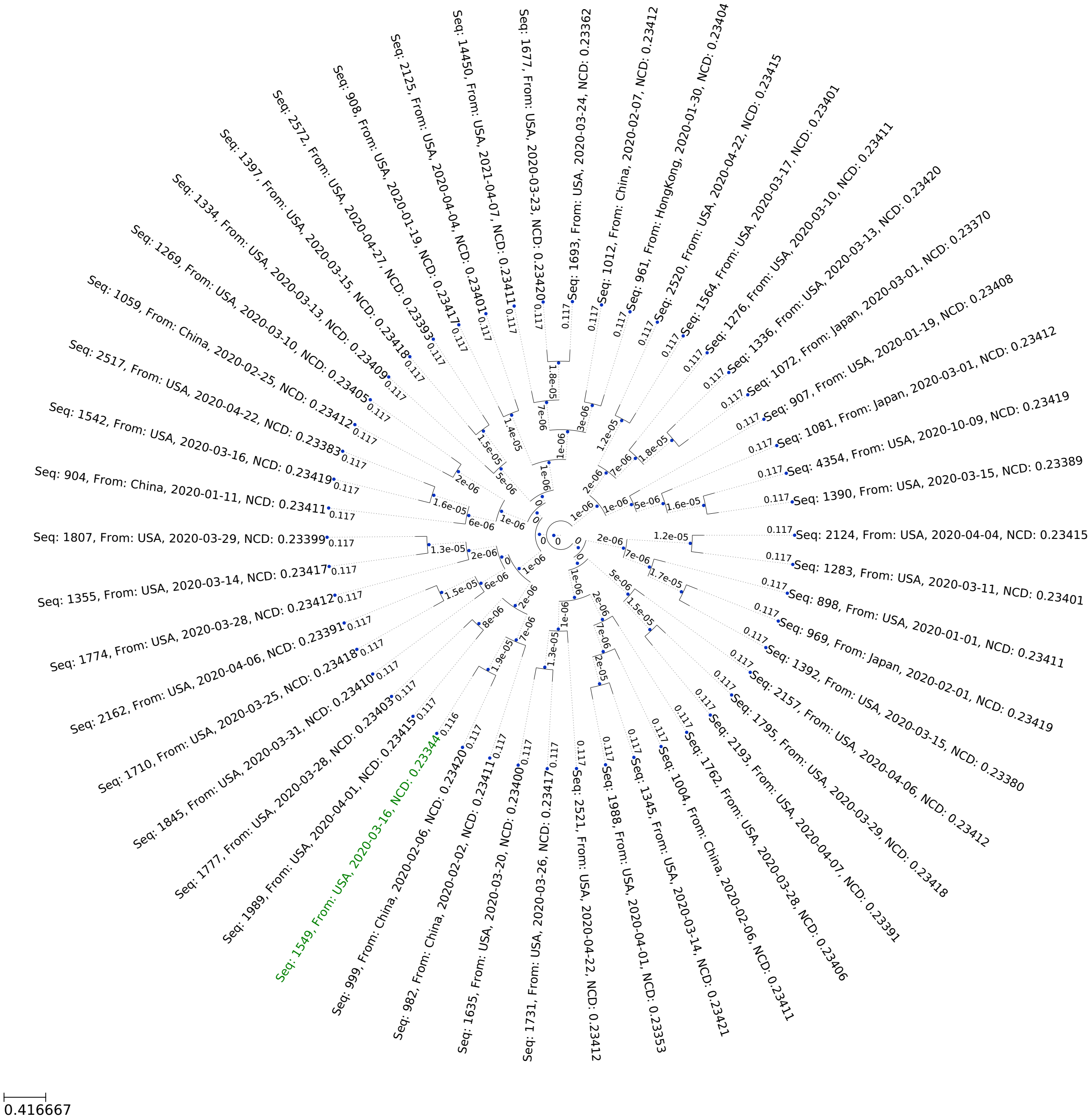

Figure S5: Example of a phylogenetic tree created using Normalized Compression Distance (NCD) values.

3.5 Reproducing the "Complexity profiles (NC) application"

To generate the complexity profiles (NC) plot, run the script below:

```
bash ComplexitySars.sh                                              # Computes the NC
python3 CompProfileSars.py comp-data.csv sorted_output.fa 0.961 0.9617  # Complexity plots
mv NCProfilecomp-data.csv.pdf NC.pdf                                # Change name
```

3.6 Reproducing the "Frequency profiles application"

To generate the frequency profile plot, run the script below:

```
bash FrequencySars.sh                      # Computes the frequency of each base of each sequence
python3 combine_freq_and_date.py           # Frequency (alphabet) plots
mv base_frequencies_plot.pdf Freq.pdf      # Change name to better distinguish
```

3.7 Reproducing the "Relative singularity (RAWs) profiles"

To generate the relative singularity (RAWs) profiles, run the script below:

```
bash RawSars.sh                                        # Computes mRAWS and statistics
python3 RawSarsProfile.py sorted_output.fa             # Creates the relative singularity profiles
mv relativeSingularityProfile.pdf RAWProfiles.pdf      # Change name to better distinguish
```

3.8 Reproducing the "Computational efficiency"

To obtain for your machine the computational time for each task run:

```
python Filter_time.py
python FrequencySars_time.py
python ComplexitySars_time.py
python Similarity_time.py
python RawSars_time.py
```

To obtain a comparison to grep/awk run:

```
python Filter_time_grep_comparisson.py
```

Finally to see peak RAM the script run was:

```
bash measure_performance.sh
```

4 Parameters and options of AltaiR

4.1 Main menu

The command to access the main menu with the options/parameters of AltaiR is

```
AltaiR -h
```

This command will output the following content

```
Program: AltaiR [ Alignment-free and spatial-temporal analysis
                  toolkit for large-scale multi-FASTA data ]
Version: 1.2

Usage: AltaiR <command> [options] < <file>

Commands:
      average      Moving average filter of a column float
                   CSV file (the column to use is a parameter).
      filter       Filters FASTA reads by characteristics:
                   alphabet, completeness, length, CG quantity,
                   multiple string patterns and pattern absence.
      frequency    Computes the alphabet frequencies for each
                   FASTA read (it enables alphabet filtering).
      nc           Computes Normalized Compression (NC) for all
                   FASTA reads according to a compression level.
      ncd          Computes Normalized Compression Distance (NCD)
                   for all FASTA reads according to a reference.
      raw          Computes Relative Absent Words (RAWs) with
                   CG quantity estimation for all RAWs.

Help: AltaiR <command> -h for accessing each command menu.
```

with the sub-commands available (average, filter, frequency, nc, ncd, raw).

4.2 Average menu

For accessing the sub-menu of average, the following sub-command must run

```
AltaiR average -h
```

This command will output the following content

```
NAME
      AltaiR average

DESCRIPTION
      filters a float column file using a moving average.

PARAMETERS
      -h,  --help
           usage guide (help menu),

      -v,  --verbose
           verbose mode (more information),

      -i,  --ignore-first-line
           ignores first line,

      -p,  --position
           show index position for each entry,

      -w [INT],  --window [INT]
           window size (default: 47),

      -c [INT],  --column [INT]
           column to filter (e.g. first column: -c 1),

      < [FILE]
           Input column file target (e.g. data.txt) -- MANDATORY.

SYNOPSIS
      AltaiR average [OPTION]... < [FILE]

EXAMPLE
      AltaiR average -v -w 47 < data.txt
```

with the options/parameters available.

4.3 Filter menu

For accessing the sub-menu of filter, the following sub-command must run

```
AltaiR filter -h
```

This command will output the following content

```
NAME
      AltaiR filter

DESCRIPTION
      Filters a multi-FASTA file by specific characteristics:
      alphabet, completeness, length, CG quantity, string patterns.

PARAMETERS
      -h,  --help
           usage guide (help menu),

      -V,  --version
           display program and version information,
```

```

      -v,  --verbose
           verbose mode (more information),

      -c,  --complete
           considers only genomes with matching alphabet,

      -a [STRING],  --alphabet [STRING]
           alphabet to consider (Default: ACGT),

      -min [INT],  --minimum [INT]
           minimum sequence size,

      -max [INT],  --maximum [INT]
           maximum sequence size,

      -ncg [FLOAT],  --cg-minimum [FLOAT]
           minimum CG quantity (between 0.0 and 1.0),

      -mcg [FLOAT],  --cg-maximum [FLOAT]
           maximum CG quantity (between 0.0 and 1.0),

      -p [STRING],  --pattern [STRING]
           considers reads with pattern(s) in the header,
           repeat flag for multiple patterns (case insensitive),

      -i [STRING],  --ignore [STRING]
           ignores reads with pattern(s) in the header,
           repeat flag for multiple ignores (case insensitive),

      < [FILE]
           Input FASTA target (e.g. SARS-CoV-2.fa) -- MANDATORY.

SYNOPSIS
      AltaiR filter [OPTION]... < [FILE]

EXAMPLE
      AltaiR filter -a ACGT -min 29200 -p SARS < viruses.fa
```

with the options/parameters available.

4.4 Frequency menu

For accessing the sub-menu of frequency, the following sub-command must run

```
AltaiR filter -h
```

This command will output the following content

```
NAME
      AltaiR frequency

DESCRIPTION
      computes the alphabet frequencies for each FASTA read.

PARAMETERS
      -h,  --help
           usage guide (help menu),

      -v,  --verbose
           verbose mode (more information),

      -p,  --first-line
```

```
            print first line with symbols,

      -a [STRING],  --alphabet [STRING]
           alphabet to consider (Default: ACGT),

      < [FILE]
           Input FASTA target (e.g. SARS-CoV-2.fa) -- MANDATORY.

SYNOPSIS
      AltaiR frequency [OPTION]... < [FILE]

EXAMPLE
      AltaiR frequency < viruses.fa
```

with the options/parameters available.

4.5 NC menu

For accessing the sub-menu of nc, the following sub-command must run

```
AltaiR nc -h
```

This command will output the following content

```
NAME
      AltaiR nc

DESCRIPTION
      Normalized compression estimation of multiple FASTA reads.

PARAMETERS

      -h,  --help
           usage guide (help menu),

      -v,  --verbose
           verbose mode (more information),

      -d,  --dna
           considers exclusively DNA alphabet {A,C,G,T},
           it also provides inverted repeats models,
           flag absence considers inversions (without complements),

      -p,  --show-parameters
           show parameters of the models for optimization,

      -s,  --show-levels
           show pre-computed compression levels (parameters),

      -t [INT],  --threads [INT]
           maximum number of threads to compute it,

      -l [INT],  --level [INT]
           compression level (integer),
           it defines compressibility in balance with computational
           resources (RAM & time), use -s for levels perception,

      [FILE]
           input sequence filename (to analyze) -- MANDATORY,
           FASTA file for the analysis (last argument).

SYNOPSIS
      AltaiR nc [OPTION]... [FILE]
```

```

EXAMPLE
      AltaiR nc -v -t 8 -m 11:50:0:1:0:0.9/0:0:0 seqs.fa
```

with the options/parameters available.

This sub-menu has a option to access more information about the compression models, namely through the usage of the following command

```
AltaiR nc -p
```

This option will output the following model help information

```
-m [C]:[D]:[R]:[I]:[H]:[G]/[S]:[E]:[A]

Template of a target context model.

Parameters:
     [C]: (integer [1;20]) order size of the regular context
          model. Higher values use more RAM but, usually, are
          related to a better compression score.
     [D]: (integer [1;5000]) denominator to build alpha, which
          is a parameter estimator. Alpha is given by 1/[D].
          Higher values are usually used with higher [C],
          and related to confiant bets. When [D] is one,
          the probabilities assume a Laplacian distribution.
     [R]: (integer [0;99999999] memory model. The 0 uses the
          full memory.
     [I]: (integer {0,1,2}) number to define if a sub-program
          which addresses the specific properties of DNA
          sequences (Inverted repeats) is used or not. The
          number 2 turns ON this sub-program without the
          regular context model (only inverted repeats). The
          number 1 turns ON the sub-program using at the same
          time the regular context model. The number 0 does
          not contemple its use (Inverted repeats OFF). The
          use of this sub-program increases the necessary time
          to compress but it does not affect the RAM.
     [H]: (integer [1;254]) size of the cache-hash for deeper
          context models, namely for [C] > 14. When the
          [C] <= 14 use, for example, 1 as a default. The
          RAM is highly dependent of this value (higher value
          stand for higher RAM).
     [G]: (real [0;1)) real number to define gamma. This value
          represents the decayment forgetting factor of the
          regular context model in definition.
     [S]: (integer [0;20]) maximum number of editions allowed
          to use a substitutional tolerant model with the same
          memory model of the regular context model with
          order size equal to [C]. The value 0 stands for
          turning the tolerant context model off. When the
          model is on, it pauses when the number of editions
          is higher that [C], while it is turned on when
          a complete match of size [C] is seen again. This
          is probabilistic-algorithmic model very usefull to
          handle the high substitutional nature of genomic
          sequences. When [S] > 0, the compressor used more
          processing time, but uses the same RAM and, usually,
          achieves a substantial higher compression ratio. The
          impact of this model is usually only noticed for
          [C] >= 14.
     [E]: (integer [1;5000]) denominator to build alpha for
          substitutional tolerant context model. It is
```

```
          analogous to [D], however to be only used in the
          probabilistic model for computing the statistics of
          the substitutional tolerant context model.
     [A]: (real [0;1)) real number to define gamma. This value
          represents the decayment forgetting factor of the
          substitutional tolerant context model in definition.
          Its definition and use is analogus to [G].
```

Nevertheless, several compression level models are already pre-computed. For accessing these pre-levels, the following command must run

```
AltaiR nc -s
```

This option will output the following fifteen levels and model setups

```
Level  1: -m 3:10:500:0:1:0.97/0:0:0
Level  2: -m 3:10:500:2:1:0.97/0:0:0
Level  3: -m 3:10:500:1:1:0.97/0:0:0
Level  4: -m 5:20:500:0:1:0.97/0:0:0
Level  5: -m 5:20:500:2:1:0.97/0:0:0
Level  6: -m 5:20:500:1:1:0.97/0:0:0
Level  7: -m 8:20:800:0:0:0.9/0:0:0
Level  8: -m 8:20:800:1:0:0.9/0:0:0
Level  9: -m 8:20:800:2:0:0.9/0:0:0
Level 10: -m 12:50:1000:0:0:0.9/0:0:0
Level 11: -m 12:50:1000:1:0:0.9/0:0:0
Level 12: -m 12:50:1000:2:0:0.9/0:0:0
Level 13: -m 6:20:750:0:0:0.9/0:0:0 -m 13:20:1500:0:0:0.9/2:10:0.9
Level 14: -m 6:20:750:1:0:0.9/0:0:0 -m 13:20:1500:1:0:0.9/2:10:0.9
Level 15: -m 3:1:750:0:0:0.9/0:0:0 -m 12:50:0:1:0:0.9/2:1:0.9
```

4.6 NCD menu

For accessing the sub-menu of ncd, the following sub-command must run

```
AltaiR ncd -h
```

This command will output the following content

```
NAME
      AltaiR ncd

DESCRIPTION
      Normalized Compression Distance (NCD) between a given
      reference sequence and each read in a multi-FASTA file.

PARAMETERS

      -h,  --help
           usage guide (help menu),

      -v,  --verbose
           verbose mode (more information),

      -d,  --dna
           considers exclusively DNA alphabet {A,C,G,T},
           it also provides inverted repeats models,
           flag absence considers inversions (without complements),

      -p,  --show-parameters
           show parameters of the models for optimization,

      -s,  --show-levels
           show pre-computed compression levels (parameters),

      -t [INT],  --threads [INT]
           maximum number of threads to compute it,
```

```

      -l [INT],  --level [INT]
           compression level (integer),
           it defines compressibility in balance with computational
           resources (RAM & time), use -s for levels perception,

      -r [FILE],  --reference [FILE]
           reference sequence to calculate the NCD to the reads,

      [FILE]
           input sequence filename (to analyze) -- MANDATORY,
           FASTA file for the analysis (last argument).

SYNOPSIS
      AltaiR ncd [OPTION]... [FILE]

EXAMPLE
      AltaiR ncd -v -m 11:50:0:1:0:0.9/0:0:0 -r ref.fa seqs.fa
```

with the options/parameters available.

This sub-menu has a option to access more information about the compression models and levels, namely through the usage of the same way as in the nc menu.

4.7 RAW menu

For accessing the sub-menu of raw, the following sub-command must run

```
AltaiR raw -h
```

This command will output the following content

```
NAME
      AltaiR raw

DESCRIPTION
      Computation of minimal Relative Absent Words (mRAWs).

PARAMETERS

      -h,  --help
           usage guide (help menu),

      -f,  --force
           force mode (overwrites old files)

      -v,  --verbose
           verbose mode (more information)

      -vv, --very-verbose
           very verbose mode (much more information)

      -a,  --aminoacids
           use amino acids/proteins models

      -t,  --threads
           does NOT use threads if flag is set (slower)

      -i,  --ignore-ir
           does NOT use inverted repeats if flag is set

      -o,  --stdout
           write overall statistics to standard output

```

```
      -p,  --plots
           print Shell code to generate plots (gnuplot)

      -min [NUMBER],  --minimum [NUMBER]
           k-mer minimum size (usually 10)

      -max [NUMBER],  --maximum [NUMBER]
           k-mer maximum size (usually 16)

      [FILE]
           Input host FASTA (e.g. human) -- MANDATORY.
           This content will be loaded in the models.

      [FILE]
           Input parasite FASTA  (e.g. SARS-CoV-2) -- MANDATORY.
           The mRAWs will be mapped on this content file.

SYNOPSIS
      AltaiR raw [OPTION]... > output.fa

EXAMPLE
      AltaiR raw -v -min 11 -max 16 human.fa SARS-CoV2.fa
```

with the options/parameters available.

References

[1] Björn Grüning, Ryan Dale, Andreas Sjödin, Brad A Chapman, Jillian Rowe, Christopher H Tomkins-Tinch, Renan Valieris, and Johannes Köster. Bioconda: sustainable and comprehensive software distribution for the life sciences. *Nature methods*, 15(7):475–476, 2018.
