# Supplementary material for: AltaiR: a C toolkit for alignment-free and temporal analysis of multi-FASTA data
Source: Gigascience. 2024 Nov 26;13:giae086. doi: 10.1093/gigascience/giae086 (PMC11590114; doi:10.1093/gigascience/giae086)
Supplement: giae086_GIGA-D-23-00393_Revision_1 [file giae086_giga-d-23-00393_revision_1.pdf]

GigaScience

AltaiR: a C toolkit for alignment-free and temporal analysis of multi-FASTA data

--Manuscript Draft--

**Manuscript Number:**	GIGA-D-23-00393R1
**Full Title:**	AltaiR: a C toolkit for alignment-free and temporal analysis of multi-FASTA data
**Article Type:**	Technical Note
**Funding Information:**	European Union's Digital Europe Programme (101081813) — PhD Jorge Miguel Ferreira da Silva<br>Fundação para a Ciência e a Tecnologia (CEECINST/00026/2018) — PhD Diogo Pratas<br>Fundação para a Ciência e a Tecnologia (UIDB/00127/2020) — Not applicable
**Abstract:**	Background: The vast number of viral genome sequences generated during the latest pandemic has presented new challenges for computational analysis. Analyzing millions of viral genomes in multi-FASTA format is computationally demanding, especially when using alignment-based methods. Most existing methods are not designed to handle such large datasets, often requiring the analysis to be divided into smaller parts to obtain results using available computational resources.<br>Findings: We introduce AltaiR, a toolkit for analyzing multiple sequences in multi-FASTA format using exclusively alignment-free methodologies. AltaiR enables the identification of singularity and similarity patterns within sequences and computes static and temporal dynamics without restrictions on the number or size of input sequences. It automatically filters low-quality, biased, or deviant data. We demonstrate AltaiR's capabilities by analyzing more than 1.5 million full SARS-CoV-2 sequences, revealing interesting observations regarding viral genome characteristics over time, such as shifts in nucleotide composition, decreases in average sequence complexity, and the evolution of the smallest sequences not found in the human host.<br>Conclusions: AltaiR can identify temporal characteristics and trends in large numbers of sequences, making it ideal for scenarios involving endemic or epidemic outbreaks with vast amounts of available sequence data. Implemented in C with multi-threading and methodological optimizations, AltaiR is computationally efficient, flexible, and dependency-free. It accepts any sequence in FASTA format, including amino acid sequences. The complete toolkit is freely available at https://github.com/cobilab/altair.
**Corresponding Author:**	Jorge Miguel Ferreira da Silva<br>Universidade de Aveiro Instituto de Engenharia Eletrónica e Informática de Aveiro<br>Esmoriz, Seleccione um PORTUGAL
**Corresponding Author Secondary Information:**	
**Corresponding Author's Institution:**	Universidade de Aveiro Instituto de Engenharia Eletrónica e Informática de Aveiro
**Corresponding Author's Secondary Institution:**	
**First Author:**	Jorge Miguel Ferreira da Silva, PhD
**First Author Secondary Information:**	
**Order of Authors:**	Jorge Miguel Ferreira da Silva, PhD
	Armando J Pinho, PhD
	Diogo Pratas, PhD
**Order of Authors Secondary Information:**	
**Response to Reviewers:**	Editor and Reviewer Comments:<br><br>We are grateful for the reviewer's positive appreciation of our work and the helpful comments and suggestions. We carefully considered the reviewers' comments and have revised the manuscript to address each comment. (please see if the response is

Powered by Editorial Manager® and ProduXion Manager® from Aries Systems Corporation

not complete here, due to a system problem, I have attached the response as well as one of the supplementary materials).

Reviewer #1:

Summary

The authors present a toolkit (AltaiR) for analysing multiple sequences in multi-FASTA format that is exclusively characterized by alignment-free methodologies.

It computes:

-- Normalized Compression Distance profiling

-- Normalized Compression (complexity) analysis

-- temporal nucleotide composition variation

-- the identification of Relative Absent Words,

The manuscript is hard to read. However, the tool is well implemented and the GitRepo is user-friendly.

Response:

We appreciate the comments and hope the revised manuscript satisfies the reviewer.

Comments

Major

- The term "spatial-temporal" in the title is misleading because the method takes neither spatial nor temporal dependencies into account.

Response: Thank you for your observation. Firstly, we would like to clarify an oversight regarding the title; it mistakenly referred to "spacial-temporal" rather than the correct term "spatiotemporal." Secondly, we recognize the current title's phrasing as "spatial-temporal" might be misleading. In response, we updated the title and document to focus solely on the "temporal" dimension. Although our initial aim was to indicate the paper's capacity for both space and time analysis, we admit that the space analysis aspect is not fully flushed out in this paper. Specifically, the NCD and NC profiles are the most evident AltaiR functions that can serve to preform space analysis due to their ability to identify similar sequences, and complexity trends across different regions.

However, the paper's strength lies in its temporal analysis, especially evident in tracking pathogen behaviour over time, as demonstrated in the COVID-19 case study through effective metadata utilization and efficient scripting. As such, we will maintain the emphasis on temporal analysis in the title and document.

Update installation instructions:

The one on GitHub doesn't work instead:

mamba create -n altair -c conda-forge -c bioconda altair-mf

Response: We apologize for the oversight. We could not replicate the issue on our side, however, since this issue has occurred in the reviewer’s environment, we added the command to the supplementary material of the paper and the GitHub repository.

Without comparison to other methods or toolkits, sentences like "ALtaiR is a significant advancement in the field" (see Discussion) don't seem appropriate. Therefore, it would be useful to include the following comparisons:

Please include a comparison (as a table?) to other toolkits that are mentioned in the

introduction (SeqAn, khmer, GTO, etc) in terms of functionalities and efficiency (programming language, parallel computing)

It seems like one of the main advantages of this tool is its clever header filtering. The authors should provide a runtime comparison to the grep method.

Response: Thank you for the suggestion to include a comparison of AltaiR with other bioinformatics toolkits mentioned in the introduction. We agree that providing a comprehensive comparison of AltaiR's functionalities and efficiency concerning other state-of-the-art tools will strengthen the manuscript and help readers better understand AltaiR's unique features and advantages.

We have added to the discussion a detailed comparison of AltaiR with SeqAn, khmer, and GTO, and incorporated this comparison in a table that highlights key features such as programming language, parallel computing support, alignment-free analysis capabilities, genome filtering options, and other relevant functionalities.

The input requirements are not clear:

- the authors should specify explicitly for which pathogens the tool is suitable

Response: Thank you for the valuable feedback. The alignment-free methods and efficient handling of large, complex datasets make AltaiR well-suited for studying the genomic and proteomic sequences of these diverse pathogens. As such it can be used for analyzing a wide range of pathogens, including both small and large viruses (e.g., SARS-CoV-2, influenza viruses, herpesviruses), bacteria (e.g., Escherichia coli, Salmonella species), fungi (e.g., Candida albicans, Aspergillus fumigatus), and protozoa (e.g., Plasmodium species, Toxoplasma gondii).

- what is the minimum input AltaiR requires?

Response: Thank you for the question. The minimum input depends on the task the user desires to accomplish. Although this tool is developed to run with millions/thousands of sequences, you can run it for some tasks with one sequence (filtering, NC and frequency analysis); and for NCD and RAWs you only require 2. For NCD the user only requires a refence sequence to compare toward and another to compare. In the case of the RAWs computation, the process requires one as a reference (the sequence of the host: genome, transcriptome or both) and the genome of the pathogen.

- is the tool using the information of when the sequence was submitted/samples for the temporal mapping? How are these passed to the tool?

Response: Indeed. The tool is assuming order in the multi-fasta. Meaning the fasta provided to the tool is ordered by the analysis intended to be performed. The information of this order is obtained from the headers of the sequences, which have information regarding, date of submission, sample collection data, location, etc. In the case of this paper, we first retrieved the multi-fasta from NCBI with this information. Then, we ordered the sequences based on the header's information (date), after ordering the sequences were fed to AltaiR for analysis. For example, the NCBI viral database already provide this sorting feature.

- what is the minimum number of sequences you would recommend users to use?

Response: Regarding the minimum number of sequences recommended for analysis with AltaiR, we suggest using as many high-quality sequences as possible to obtain robust and meaningful results. The more sequences included in the analysis, the better the tool can capture the diversity and evolutionary patterns within the pathogen population.

However, the exact number of sequences required may vary depending on the specific research question and the stage of the analysis. For example, initial exploratory analyses may benefit from a larger, more inclusive dataset, while downstream analyses may focus on a smaller subset of high-quality, filtered sequences.

- how does the tool behave on substantially larger genomes?

Response: The tool is designed to scale efficiently with increasing sequence sizes. The time complexity of AltaiR's algorithms is approximately linear with respect to the input sequence size, making it highly scalable for analysing larger genomes, such as those of fungi, bacteria, and even eukaryotic organisms, without a significant increase in computational time.

- What is the overall runtime of your SARS-CoV-2 application? Can you put this in relation to other tools? This would give users a better understanding of what to expect.

Response: Directly comparing the runtime of our SARS-CoV-2 with other tools is challenging due to the unique set of features and functionalities offered by AltaiR. Our toolkit focuses on alignment-free methods, such as generating NCD profiles, and complexity profiles, and identifying RAWs, which are not commonly found in other tools. We have also added a section with the runtimes of the program to the paper, so it is possible to evaluate the execution time of the program in different scenarios.

The method section appears to be unnecessarily hard to read (concrete cases are listed in the minor comments section). It contains a lot of seemingly complex formulas for already established and well-known summary statistics (e.g. GC content, nucleotide-/amino acid frequency, etc.). Also, it is not described how AltaiR filters sequences by "completeness". Additionally, the manuscript would benefit from a clear overview figure.

Response: Thank you for your feedback. We have updated the manuscript to address your concerns:

We have already included an overview figure (Figure 1) that illustrates the workflow of the AltaiR toolkit, including the main methods and input types. This figure has been updated to provide a clear overview of the toolkit's functionality.

Regarding the formulas, while they may be well-known to some readers, we believe that including them improves the readability of the paper and allows a broader audience to understand the underlying concepts. The formulas serve as a reference for readers who may not be familiar with the specific methods and provide a clear mathematical foundation for the implemented techniques.

Completeness, in the context of genomic sequences, refers to the availability of the full, uninterrupted sequence of an organism's genome, rather than just partial or fragmented sequences. Filtering based on completeness ensures that only high-quality, contiguous sequences are included in the analysis, minimizing the potential bias introduced by incomplete data. To improve the readability of the manuscript this information was also updated in the manuscript.

Minor

- General: Missing line numbers impedes the reviewing process as it makes it harder to point out things while reading the manuscript.

Response: We thank the reviewer for this comment, in fact the lack of line numbers complicates the reviewing process. We now provide line numbers in the manuscript.

- abstract: Please define sequence complexity!

- abstract: can you use a more specific term than "flexible"?

Response: Thank you for your questions about the abstract. In this context, sequence complexity refers to the Kolmogorov complexity, which is a measure of the computational resources needed to specify an object, such as a sequence. It quantifies the amount of information contained within a sequence, with higher complexity indicating a greater degree of randomness or unpredictability, and lower complexity suggesting more repetitive or predictable patterns. In our study, we approximate the

Powered by Editorial Manager® and ProduXion Manager® from Aries Systems Corporation

Kolmogorov complexity using compression-based methods.

Regarding the term "flexible", we used it to convey that AltaiR is highly parameterizable and can be easily integrated into various pipeline configurations. The toolkit's modular design allows users to customize the analysis based on their specific requirements and seamlessly combine AltaiR's tools with other bioinformatics software.

- p.2, Introduction: spatial-temporal characteristic: what are those characteristics? And where is the spatial or temporal data coming in?

Response: Thank you for your question. The spatio and temporal characteristics are the characteristics that arise from analysing these multi-fasta across a long period of time and across different countries. It is possible to order the sequences using headers' metadata (date of sequencing, region, etc) by doing so and analysing this information it is possible to extrapolate how the behaviour of the pathogen changes, across region and/ period of time.

- p.3, left column: what is "compact version of the data"? Be more precise compressed data? " to compact data." → is this compressed?

Response: Thank you. We have changed the text to compressed data.

- p.3, left column: "The workflow contains seven main methods" → are those all applied to all the three data inputs?

Response: Indeed, not all of the seven methods utilize the same inputs.
The methods that use only pathogen sequences (e.g. only a large multi-fasta) are filtering, NC computation and nucleotide sequence frequence computation. Some methods require additional sequences. NCD requires a reference sequence (the sequences which all other pathogenic sequences are compared to), whereas the RAWs requires the host omics (can be only the genome of the host, the transcriptome or both).
All profiles are created using the information from these computations (e.g. simple bash or python scripts), so these are derivative and do not require the source data per se.

- p.3, Figure 1: Maybe you can use different symbols for data and methods, and in general make clear which method is used for which data. What is the minimum input?

Response: Thank you for your feedback. The manuscript was updated to accommodate these changes in the image.

Can the input be sequences from different pathogens?

Response: Yes, it is possible in some cases that the input is a sequence for another host. A good example would be to see the similarity over time of a given virus towards another (using the NCD). This could be done as an example with another coronavirus as a reference and analyse the similarity towards sars-cov-2. Another example is in filtering, a user can accidentally place a sequence that does not belong in the mix of the input and by filtering it would be very straightforward to remove such samples from the pool of sequences.

Which method is for which input data (maybe color-coding in the figure could help)

Response: Thank you for your feedback. The manuscript was updated to accommodate these changes in the image.

- p.3, right column: "The computation can be applied to different sequences;" → the word computation is irritating, this is a filtering step.

Response: Thank you, the text was updated.

- p.3, right column: "The computation can be applied to different sequences;"

Powered by Editorial Manager® and ProduXion Manager® from Aries Systems Corporation

→ is the essence of this and the following sentence that different filtering characteristics can be applied to different viruses, and they can be changed while the program is running? If so please, make it precise

Response: Thank you, for your comment. Although filtering is highly parameterizable, and you can apply different filtering characteristics to different virus, this is not possible to do while the program is running. It has to be defined prior to running the filtering step.

- p.3, right column: the phrase "intersected through a single run" is not clear. Are you allowed to change the filter criteria while running? this seems not in accordance with reproducibility

Response: Thank you, you are correct, it is not possible to change the filtering criteria while the program is running. What we meant to explain is that several filtering criteria can be applied at the same time (i.e. doing an AND). This was clearly not well explained and as such this paragraph was changed.

- p.3, right column: term "Alphabet composition" is not clear.

Response: By alphabet composition we mean the number of unique letters (and what letters) are contained in the sequence (e.g. in genomes it is usually ACGT, while for proteins are different amino acids).

- p.3, right column: "Alphabet composition": what you are doing with symbols that don't conform with the given alphabet, is the whole sequence excluded? Is only that symbol excluded, and does that influence the analysis?

Response: The filtering method is done to exclude sequences that are not in conform with the given alphabet. If the user wishes to still use sequences that have a given symbol, they must specify it and treat it priorly (e.g. sometimes sequences contain N symbol, an option is to include the sequence and afterwards run a script to change that symbol randomly to ACGT).

- p.4, left column: "Header patterns and anti-patterns"; it doesn't seem necessary to introduce the mathematical notation "yi", which is not used subsequently.

Response: We prefer to keep notation to provide a mathematical congruence to the text.

- p.4, left column, last paragraph: what is the "reference sequence"? How is it defined by the user and how should it be chosen?

Response: We apologize, in page 4, we did not find any mention of reference sequence only reference genome (which are used interchangeably). If we understood correctly, this section corresponds to the NCD. In this case the reference sequence is the sequence that is going to be compared towards all the other sequences in the multi-fasta. This sequence is defined by the user in the parametrization. Usually is a sequence that is not comprised in the multi-fasta (although it can be), and the objective is to assess the similarity, or “evolution/trend” of the fasta compared to an original sequence. The selection depends on what the user desires to measure. For instance in sars-cov-2 it might be useful to assess similarity to other different virus that may originated it; in other scenarios such as multi-resistant bacteria, it might be useful to test (within the same species) the variation of a non-resistant strain towards another strains that have evolved resistance to bacteria in order to understand the evolution of this strain over time relatively to the original non-resistant strain.

- p.4, right column, mentions a time complexity of y + xn. It's not quite clear how the value of x is calculated. Does this somehow correspond to the previously mentioned number of bits to compress each individual x^i? If yes, how is this generalized to a single value of x?

Response: The time complexity mentioned, y + xn, represents the total time required to compute the NCD values for n target sequences (x^1, ..., x^n) against a single

reference sequence y. Here, y represents the time needed to compress the reference sequence and freeze its compression models. This is done only once, regardless of the number of target sequences. x represents the average time needed to compress a single target sequence x^i, given the frozen compression models of the reference sequence y. Since the compression models for y are already available, compressing each target sequence x^i takes, on average, x units of time. Therefore, the total time complexity to compute the NCD values for all n target sequences against the reference sequence is y (for compressing and freezing the models of the reference sequence) plus x * n (for compressing each of the n target sequences using the frozen models of the reference sequence). The value of x is not directly related to the number of bits needed to compress each x^i. Instead, it represents the average time needed to compress a single target sequence, given the frozen compression models of the reference sequence.

- p.4, right column, "Complexity (NC) Profiles": how does the sorting by time affect the analysis? the first sentence of the section suggests that.

Response: As we have previously explained, the way the set of sequences is ordered will impact the results, since the program assumes the order of sequences in the multi-fasta (e.g. in temporal analysis, the first sequence is the oldest, where the most recent is the last). As such, the way data is provided to the program is of extreme important to obtain a valuable analysis of the data. A good example is the evolution of Sars-Cov-2 across time. If the order was incorrect, different profiles and analysis would be performed.

- p.4, right column, formula (8) introduces Σ as a new symbol for the alphabet of a sequence. We understand that a symbol for the alphabet of a sequence has already been introduced in p.3 as Ξ.

Response: Thank you for noticing this. Yes, the text has been changed accordingly.

- p.5, left column, 2nd paragraph: typo? "For genomic sequences, Σ is 4 for any" should it be the absolute value of Σ?

Response: Thank you for noticing this. Yes, the text has been changed accordingly.

- p.5, left column, 2nd paragraph: "alphabet trimming" - it would be helpful to add a sentence explaining alphabet trimming and why it is solving this issue

Response: Thank you for your suggestion. We agree that clarifying the concept of alphabet trimming would be beneficial for readers. In the context of proteomic sequences, the alphabet size can vary due to the presence of ambiguous or non-standard amino acid characters. This variation can lead to changes in the Normalized Compression (NC) profile, as the NC value is calculated using the alphabet size. Alphabet trimming is a preprocessing step that reduces the alphabet size by removing or substituting non-standard characters. By applying alphabet trimming, we ensure a consistent alphabet size across all sequences, mitigating the impact of alphabet variations on the NC profile. This step helps maintain the comparability of NC values and facilitates the interpretation of the sequence complexity analysis. We added a sentence in the revised manuscript to explain the concept of alphabet trimming and its role in addressing the issue of varying alphabet sizes in proteomic sequences.

- p.5, left column, section Relative singularity (RAWs) profiles, first sentence is hard to read, also add another sentence to say why these regions are of interest

Response: We appreciate your feedback on the clarity of the first sentence in the "Relative singularity (RAWs) profiles" section. We have revised the sentence to improve readability and added another sentence to explain the significance of these regions.

- p.6, left column, "Results", could you provide another sentence of the motivation or what you want to find out with the SARS-CoV-2 data?

Response: Thank you for your suggestion. We agree that providing additional context

Powered by Editorial Manager® and ProduXion Manager® from Aries Systems Corporation

about our motivation for analyzing the SARS-CoV-2 data would improve the clarity of the Results section. We have added a sentence to address this.

- p.6, left column, 2nd paragraph: "In some cases" is ambiguous, can you explain those cases

Response: Thank you for pointing out the ambiguity in the phrase "In some cases." We have clarified this by providing specific examples of when reading from standard input and writing to standard output are useful in AltaiR. The revised text is as follows:

"The toolkit allows reading and writing from standard input and output, respectively, in certain cases. For example, this feature enables the direct piping of the output from the filtering tool to the NC, NCD, and RAWs tools, streamlining the analysis process. Additionally, it allows for the output profiles of the frequency, NC, NCD, and RAWs tools to be easily processed by other tools, such as those performing averaging or visualization. This functionality provides easier integration to build custom pipelines. Furthermore, most tools (except average) use multi-threading to speed up the analysis."

The added examples clarify the specific instances where reading from standard input and writing to standard output are beneficial in AltaiR, such as directly connecting the output of one tool to another and facilitating the integration of AltaiR's tools with other software in custom pipelines.

- p.6, left column, 2nd paragraph: typo "README.am" > "README.md"

Response: Thank you for noticing this. Yes, the text has been changed accordingly.

- p.6, right column, 1st paragraph: "sufficient for accurate downstream analysis. This sequence filtering step shows its critical importance, proven by the high variability between these sequences, which can harm downstream analyses without proper filtering." → could you elaborate what potential effects could be?

Response: Thank you for your suggestion to elaborate on the potential effects of high variability between sequences on downstream analyses. We have expanded on this point in the revised text.

- p.6, "In some cases, the toolkit allows reading and writing from standard input and output". What are these cases and what are the limitations? What's exactly meant by "stand input and output"?

Response: Yes, as we have explained previously, the software can make use of stand input and output (i.e. use pipes: "|" in the command line environment), this allows for a more efficient execution of code. E.g. It is possible to filter the sequences, directly perform a NC analysis of the filtered sequences and preform and average of the results to them plot them. Of course, some pipe's don't make sense such as feeding the NC results to NCD.

- p.6, Which of the listed tools utilize multithreading? We suggest mentioning this either in a table or directly in the bullet points in the section "Toolkit Implementation".

Response: All of them, with the exception of average use multithreading. Since all tasks are computationally intensive and evolve a large number of sequences tasks the toolkit is developed in low-level language (C language) and make abundant use of multi-threading to make the computations as efficient and as fast as possible given the available resources.

- p.6, Figure 2: Consider limiting the x-axis range to (or closer to) the filtered interval

Response: We appreciate the reviewer insight. Although not visible, data points exist in the points shown in the Instagram, so for congruency, we will keep this graphic as is.

-p.6, right column, 2nd paragraph: "time point sequence" - what is that?

	Response: A "time point sequence" refers to a sequence that represents a specific point in time within a series of sequences generated through a mutational process. In the context of the synthetic data example, the original sequence (time-0) is the starting point, and subsequent sequences (time-1, time-2, etc.) are generated by applying consecutive SNP mutations to the previous sequence. Each of these sequences represents a specific time point in the simulated evolutionary process.<br><br>In the first example, the time point sequences are used directly as references for computing the temporal similarity profiles. However, in the second example, mutated versions of the time point sequences are used as references to simulate a more realistic scenario where the available reference sequences already contain mutations compared to the true ancestral sequences.<br><br>- p.7. Figure 3: What is the "Sequence Number"<br><br>Response: In Figure 3 the “Sequence Number” refers to the positional index of the sequence in the multi-fasta file. As explained priorly the program assumes order of the input multi-fasta. Since this multi-fasta was previously ordered by date, the Sequence Number provides a temporal point in the file.<br><br>- p.7, Figure 3: difference between A and B is not clear, what sequences are used in A? what is the reference in B?<br><br>Response: The image “A” was computed with the reference being the Sars-Cov-2 refence genome sequences, and “B” was computed using RaTG13 sequence genome. The caption text was altered to improve readability and understanding of this.<br><br>- p.7. Figure 3, caption: "Similarity (NCD) profiles using (A) the whole filtered SARS-CoV-2 genome sequences and (B) the reference RaTG13 (B)." why twice (B) reference? - not clear<br><br>Response: As previously mentioned, the caption was updated since it was not clear. The first profile was computed using the Sars-Cov-2 reference sequence genomes; the second was computed using the RaTG13 sequence genomes, so the profiles are distinct. Although both are compared toward using the whole filtered SARS-CoV-2 genome sequences the reference sequences are different, and as such the profile is consequentially different as well.<br><br>- p. 7, left column, 5th paragraph: "The SARS-CoV-2 genomes are approximately 117k, resulting in multiple assembled genomes, sources, and techniques." → this sentence is not understandable<br><br>Response: Thank you for pointing out the lack of clarity in the sentence. The sentence has been changed in the manuscript for better clarity.<br><br>- p.9, Table 1: Changes in mRAWs Nucleotide Distribution are hard to make out from the table if the magnitude of the number’s changes. Consider normalizing these table entries so changes in nucleotide distributions across kmers are easy to spot.<br><br>Response: Thank you for your suggestion. While we agree that normalization could help spot relative changes, providing the absolute number of occurrences is important since absolute values give readers a clear understanding of the scale and magnitude of the mRAWs found in the SARS-CoV-2 genome. Furthermore, the absolute numbers provide context for the subsequent plots and analyses, allowing readers to interpret the results better and draw meaningful conclusions.<br><br>- p.9, Conclusion: "AltaiR's technical innovations, particularly in data filtering and processing [...]". AltaiR's filtering revolves on already commonly used and well-known summary statistics. Add more context to this statement.<br><br>Response: Thank you for your feedback regarding the statement about AltaiR's technical innovations in data filtering and processing. We acknowledge that some of the individual filtering methods used in AltaiR, such as sequence length and GC content, are well-established techniques. However, AltaiR's innovation lies in the

integration of these methods with novel and enhanced features into a comprehensive toolkit specifically designed for efficient, large-scale genomic data analysis.

AltaiR introduces several new methods, such as the Frequency method, which provides a more comprehensive and detailed analysis of nucleotide or amino acid occurrence within the reads, enabling alphabet filtering. The Filter method extends beyond the capabilities of other tools by supporting multiple simultaneous pattern searches, absent pattern searches, and filtering by GC content, sequence length, or completeness.

Moreover, AltaiR's NCD method introduces a novel approach that uses a conjunction of reference and target files while freezing the models of one and saving computation time for other sequences. This methodology, specifically designed for AltaiR, allows for substantial time savings in NCD calculations.
The combination of these tools, along with the optimization for analyzing millions of sequences, ensures high portability and ease of installation while providing the necessary capabilities for rigorous analyses. AltaiR's flexible integration into various analytical workflows through standard input and output combinations makes it a versatile choice for bioinformatics research.
We have revised the text in the to provide more context and clarity regarding AltaiR's technical innovations and their significance in the field of genomic data analysis.

- Supplementary Figure 1: Please add to the figure caption what the Time point is

Response: Thank you for the suggestion. We have updated the figure captions to include the definition of a "time point" in the context of the synthetic data experiment.

Reviewer #2:
In their manuscript, Silva et al introduce Altair, a toolkit written in the C programming language for the rapid analysis of sets of sequences in multi fasta format.
The authors showcase potential application of their tool on a collection of more than 6 million genomes from the SARS-CoV-2 coronavirus collected over the first 2 years of the COVID-19 pandemic.
In general, the paper is well written, although sometimes some biological concepts are expressed using inaccurate or imprecise/wording.
In the opinion of this reviewer the paper suffers from several important limitations, which should all be addressed for considering the work by Silva et al for publication.

Response: We appreciate the comments and hope the current revised manuscript satisfies the reviewer.

A list of the main point of criticism follows:
1# Abstract. Authors use some very strong wording to explain the rationale that led to the development of Altair. This reviewer would beg to disagree on the following concepts.
#1.1 Abstract/background: the first sentence needs to be completely rewritten, and specifically the reference to "the current phase of genome sequencing and reconstruction".

#1.2 Abstract/findings: please rephrase evolutionary patterns. What you highlight in the results is not evolutionary patterns, but rather interesting observations regarding viral genome characteristics in time. The epiphenomenon that you describe is more complex, and is the product of several factors, including natural selection, sampling bias, and mitigation policies by health authorities among the other things.

Response: We would like to thank the reviewer for the comments. We agree and the abstract has been updated to accommodate this information.

2# Introduction and rationale.

#2.1 I do not understand/agree with the choice to design Altair to work with information extracted from the headers of fasta files. For SARS-CoV-2 (and several other pathogens) structured metadata are available. See for example: https://nextstrain.org/ncov/open/global/6m and "All sequences and metadata".

Response: Thank you for your feedback. We understand your concern regarding the use of information extracted from the headers of FASTA files in AltaiR. While structured metadata, such as those available for SARS-CoV-2 through platforms like Nextstrain, can provide valuable information for analysis, AltaiR is designed to work at a lower level and serve a different purpose.

AltaiR's approach is intended to be more flexible and adaptable to various datasets, including those where structured metadata may not be readily available or consistently formatted. By extracting relevant information directly from the FASTA headers, AltaiR can be applied to a wider range of datasets and organisms without relying on external metadata sources.

Moreover, AltaiR's low-level approach allows for the analysis of raw sequence data, which can be particularly useful for exploratory studies, quality control, and cases where metadata may be incomplete or inconsistent.

#2.2 Please provide references for the section when you state: "However, creating temporary files ... in not efficiently affordable". Millions of SARS-CoV-2 sequences were analysed during the COVID-19 pandemic by using or adapting previously available tools. See for example: https://github.com/andersen-lab/bjorn and https://github.com/matteo14c/HaploCoV.

Response: Thank you for bringing the HaploCoV and bjorn tools to our attention, we will be sure to add them to the manuscript. These tools demonstrate the successful application of computational methods for analyzing large volumes of SARS-CoV-2 genomic data. However, we believe that AltaiR offers several unique advantages and capabilities that complement and extend the functionality of existing tools.

Firstly, AltaiR is specifically designed to handle vast amounts of genomic data efficiently. Its implementation in C and support for multi-threading enable faster processing times compared to tools primarily written in interpreted languages like Python or Perl. This efficiency is crucial when dealing with the ever-growing number of SARS-CoV-2 sequences.

Secondly, AltaiR's focus on alignment-free methods, such as generating Normalized Compression Distance (NCD) profiles and complexity profiles, sets it apart from tools that rely on alignment-based approaches. These methods enable efficient and scalable comparisons of genomic sequences without the computational burden of sequence alignment, making AltaiR well-suited for analyzing large, diverse datasets.

Moreover, AltaiR offers advanced filtering capabilities based on sequence length, GC content, and specific patterns, ensuring that high-quality, relevant data is used for downstream analyses. This rigorous filtering step, demonstrated in our analysis of the SARS-CoV-2 dataset, is essential for minimizing noise and bias in the results.

Another key strength of AltaiR is its ability to identify Relative Absent Words (RAWs) in genomic sequences. By comparing RAWs in SARS-CoV-2 genomes with those in the human genome, AltaiR can uncover distinctive genomic elements that may be relevant for understanding virus-host interactions and developing diagnostic tools. This functionality is not available in other toolkits (as far as we know).

While HaploCoV and bjorn have demonstrated their utility in specific aspects of SARS-CoV-2 genomic analysis, such as variant detection and data processing, AltaiR offers a more comprehensive suite of tools that enable a wide range of analyses, from similarity and complexity profiling to RAW identification and temporal analysis.

#2.3 Lack of tools for flagging/filtering low-quality sequences: incomplete and low quality sequences are normally flagged by sequence providers/submitters. Authors do explicitly refer to additional guidelines and best practice, see [7,8]. Are these used/implemented by Altair. Please clarify.

Response: Thank you for your question. AltaiR has a highly parameterizable and customizable filtering system, which allows for the adherence to established guidelines

Powered by Editorial Manager® and ProduXion Manager® from Aries Systems Corporation

and best practices to address potential contamination and quality issues. This automated filtering step removes non-complete, low-quality, biased, or deviant data based on criteria such as completeness, sequence length, GC content, and other patterns. By ensuring only high-quality, contiguous sequences are included in the analysis, AltaiR minimizes bias from incomplete or contaminated data, providing accurate and reliable downstream analyses. We have added this information to the text of the paper.

#2.4 The description of the properties of a collection of sequences over time does not necessarily represent evolution (see above). Please refrain from using expressions such as "accelerated evolution".

Response: Thank you for raising this important point. You are correct that the observed changes in the viral genomic characteristics over time may not solely represent evolutionary processes. We acknowledge that our use of the term "evolutionary patterns" and "evolutionary acceleration rates" in the manuscript may be an oversimplification.

In reality, the observed changes in the viral genome composition and complexity over time are likely the result of a complex interplay of various factors, including natural selection, sampling bias, and the implementation of public health measures. While some of these changes may indeed reflect the virus's adaptation and evolution within the host population, others may be influenced by non-evolutionary processes, such as the oversampling of certain viral lineages or the effects of containment strategies on viral transmission dynamics.

To address this, we have revised the manuscript to provide a more nuanced description of the observed temporal changes in the viral genome.

2.5 The long section where authors explain why a benchmark/comparison with other tools can not be performed is not very convincing, and above all it seems a bit out of context to this reviewer. Please adapt or remove.

Response: Thank you for your feedback. We understand that the previous section discussing the challenges of benchmarking and comparing toolkits may not have been entirely convincing or relevant to the overall context of the manuscript.

To address this concern, we have adapted the section to focus on a qualitative analysis of AltaiR's capabilities concerning other established toolkits, such as SeqAn, khmer, GTO, HaploCoV and bjorn. This comparative analysis highlights the key functionalities, efficiency metrics, and capabilities of each toolkit that are crucial for handling large-scale genomic data. By providing a more targeted and contextually relevant comparison, we aim to better illustrate AltaiR's unique features and its potential to complement and extend the capabilities of existing tools in the field of genomic data analysis.

#3 Methods

#3.1 In the opinion of this reviewer authors should better clarify which methods were specifically developed within Altair and which methods are "simply" adapted from other pre-existing software packages.

Response: The current methods include average, filter, frequency, NC, NCD, and RAW. Of these, only the average method was previously established. This is a straightforward program that has undergone a minor modification to allow selection of the column to use.

The AltaiR average method is very similar to the GTO average, with few modifications made.

The Frequency method in AltaiR is designed to compute the alphabet frequencies for each FASTA read, enabling alphabet filtering. Unlike other tools that offer alphabet filtering, AltaiR provides a more comprehensive and detailed analysis by calculating the occurrence of each nucleotide or amino acid within the reads.

Powered by Editorial Manager® and ProduXion Manager® from Aries Systems Corporation

The Filter method shares small similarities with GTO, as it can filter FASTA reads. However, unlike GTO, AltaiR implementation supports multiple pattern searches simultaneously and also it include the ability to perform absent pattern searches. Furthermore, and more importantly, AltaiR's filter method includes the ability to filter by GC content, sequence length, or completeness.

The automatic computation of NCD and NC for multiple files is also a new feature. Previously, this was done using scripting, but AltaiR now provides a single tool for this purpose. Notably, scripting could not accommodate the new NCD methodology. AltaiR's NCD method, which uses a conjunction of reference and target files while freezing the models of one and saving the computation for other sequences, allows for substantial time savings. This novel NCD methodology is specifically designed for AltaiR, including a tool tailored for this specific functionality.

Finally, AltaiR RAW is an evolution of EAGLE2 (from the same authors), now including support for protein sequences, which was a limitation of EAGLE2, besides some other small additions. Thus, AltaiR RAW can be considered an EAGLE3 version (note that EAGLE3 was never published).

Moreover, the combination of these tools in a single package with optimization for millions of sequences ensures high portability and ease of installation while providing the necessary capabilities to perform rigorous analyses. Besides the capability to perform combinations of standard input and output, AltaiR allows for flexible integration into various analytical workflows, making it a versatile choice for bioinformatics research.

#3.2 But see also #2.1 I am not completely convinced that the utility for extracting/filtering information from the fasta header would be desirable and/or useful at all.

Response: Thank you for raising this concern. We understand that the utility of filtering information from the FASTA header may not be immediately apparent or universally applicable. However, we believe that this feature can be valuable in certain scenarios, particularly when dealing with large, heterogeneous datasets from various sources.

In many cases, FASTA headers contain valuable metadata, such as sample origin, date of isolation, sequencing platform, and other relevant information. This metadata can be crucial for downstream analyses, such as temporal studies, geographic comparisons, or quality control. By providing a flexible and efficient way to extract and filter this information, AltaiR enables researchers to quickly subset their datasets based on specific criteria without the need for additional preprocessing steps.

For example, when working with SARS-CoV-2 genomes from public repositories, the ability to filter sequences based on the date of isolation or geographic location can help researchers focus on specific subsets of interest, such as isolates from a particular outbreak or time period. Similarly, filtering based on sequencing platform or assembly method can help identify and remove low-quality or inconsistent sequences that may introduce biases in the analysis.

#4 Results

#4.1 Authors do not benchmark the computational requirements of Altair in terms of resources used. This needs to be addressed, authors need to demonstrate that Altair provide clear advantages w.r.t "alignment based" methods for the analysis of the same data.

Response: While we acknowledge the reviewer's suggestion to benchmark AltaiR's computational requirements against alignment-based methods, it's essential to recognize the inherent differences between alignment-free and alignment-based approaches. AltaiR's methodology, rooted in compression-based techniques like Normalized Compression Distance (NCD) and Normalized Compression (NC), fundamentally diverges from alignment-based methods by focusing on sequence compressibility rather than exact matching. This difference makes direct comparisons

Powered by Editorial Manager® and ProduXion Manager® from Aries Systems Corporation

in computational resource usage challenging, as the methodologies serve different purposes and exhibit distinct computational characteristics. Moreover, alignment-based methods may inadvertently underestimate sequence similarity due to their reliance on exact matches, whereas compression-based techniques, as encapsulated in AltaiR, leverage the Kolmogorov complexity notion to capture similarity more comprehensively. Additionally, AltaiR is developed in optimized C language, theoretically enhancing its efficiency compared to applications developed in higher-level languages such as Python, Bash, R, or Perl. While alignment-based methods may entail significant computational overhead due to sequence alignment requirements, AltaiR's alignment-free approach offers efficiency and scalability, particularly suited for large and diverse genomic datasets. Thus, while quantitative comparisons may not be directly feasible, AltaiR's unique methodology represents a valuable alternative, enriching the toolkit available to researchers for comprehensive genomic analyses.

#4.2 Criteria applied for filtering SARS-CoV-2 sequences are too stringent. In the end only ~25k sequences are retained, starting from 6.3M. This is a very big issue in the opinion of this reviewer. Filters on length in particular are very stringent. I am not an expert in the field, but are alignment-free methods applicable when sequences are not all of the same size? This could be an important limitation. Please discuss.

Response: We must have quality data even if we have quantity. We have analyzed epidemiologic context but also in medical and forensic contexts (papers below) where it was required to perform intra-host analysis between large genomes such as herpesvirus, and therefore in these cases it is better to have the best quality data even if the number of sequences is lower.

Clinic Setting example:
-Pyöriä, Lari, et al. "Unmasking the tissue-resident eukaryotic DNA virome in humans." Nucleic Acids Research 51.7 (2023): 3223-3239.

Forensic setting example:
-Toppinen, Mari, et al. "The human bone marrow is host to the DNAs of several viruses." Frontiers in cellular and infection microbiology 11 (2021): 657245.

Notice that we used of ~25k high-quality sequences to showcase the toolkit. Here, we decided to choose less sequences but of higher quality. The toolkit is flexible to perform other analysis including when the size of the sequences differs substantially.

#4.3 NCD: the whole point of the simulation is unclear to me. The analysis is rather qualitative, to confidently state that "NCD profiles can ... predict where sequences occurred in time", you need to provide accuracy metrics (how confidently can you predict?) and evaluate also additional parameters. Further assume uniform sampling and uniform evolution are assumed: this is not very realistic.

Response: We thank the reviewer for the comments. We agree that the simulation and analysis presented are qualitative and may not provide sufficient evidence to confidently state the predictive power of NCD profiles in determining the temporal occurrence of sequences.

In light of this fact, we have revised the NCD profiles section to clarify that the primary purpose of presenting these experiments is to showcase the functionality and potential applications of the AltaiR toolkit. We have also emphasized that while NCD profiles have shown promising trends and have been successful in some cases, further investigation and more extensive research are necessary to validate their predictive power.

Our current focus is on introducing the AltaiR toolkit and its various features, and we believe that the revised section better reflects this objective. We appreciate your insights and have incorporated them into the manuscript to provide a more correct representation of the NCD profiles application.

#4.4 Since NCD is not equivalent to a molecular clock and non-calibration with sequences with "certain" temporal origins were made authors cannot conclude

Powered by Editorial Manager® and ProduXion Manager® from Aries Systems Corporation

	anything from their analysis of the RaTG13 genome. To be honest I would not include that analysis in the main text.<br><br>Response: Thank you for your comment regarding the analysis of the RaTG13 genome using NCD profiles. You raise a valid point that NCD is not equivalent to a molecular clock, and without proper calibration using sequences with known temporal origins, drawing definitive conclusions about the temporal origin of RaTG13 based solely on this analysis may be premature.<br><br>We agree that the NCD analysis of RaTG13 should be interpreted with caution, as several factors could influence the observed similarity patterns, such as the reconstruction process used for RaTG13 (e.g., reference-based assembly or de novo assembly) and the diversity of the samples used. Additionally, the lack of a robust temporal calibration limits the ability to make precise inferences about the timing of RaTG13's origin. Given these limitations, we have decided to remove the RaTG13 analysis from the main text and instead include it as a supplementary discussion.<br><br>#4.5 The sections "Frequency profile application" and "Relative singularity (RAWs) p...
**Additional Information:**	
**Question**	**Response**
Are you submitting this manuscript to a special series or article collection?	No
**Experimental design and statistics**<br><br>Full details of the experimental design and statistical methods used should be given in the Methods section, as detailed in our Minimum Standards Reporting Checklist. Information essential to interpreting the data presented should be made available in the figure legends.<br><br>Have you included all the information requested in your manuscript?	Yes
**Resources**<br><br>A description of all resources used, including antibodies, cell lines, animals and software tools, with enough information to allow them to be uniquely identified, should be included in the Methods section. Authors are strongly encouraged to cite Research Resource Identifiers (RRIDs) for antibodies, model organisms and tools, where possible.<br><br>Have you included the information requested as detailed in our Minimum	Yes

Standards Reporting Checklist?	
**Availability of data and materials**<br><br>All datasets and code on which the conclusions of the paper rely must be either included in your submission or deposited in publicly available repositories (where available and ethically appropriate), referencing such data using a unique identifier in the references and in the "Availability of Data and Materials" section of your manuscript.<br><br>Have you have met the above requirement as detailed in our Minimum Standards Reporting Checklist?	Yes

Powered by Editorial Manager® and ProduXion Manager® from Aries Systems Corporation

Main tex

Click here to access/download;LaTeX - Main Document (TeX file);main.tex

GigaScience, 2023, 1–0

doi: xx.xxxx/xxxx
Manuscript in Preparation
Technical Note

TECHNICAL NOTE

AltaiR: a C toolkit for alignment-free and temporal analysis of multi-FASTA data

Jorge M. Silva[1,2,*], Armando J. Pinho[1,2] and Diogo Pratas[1,2,3,*]

[1]IEETA/LASI, Institute of Electronics and Informatics Engineering of Aveiro, University of Aveiro, Portugal and [2]DETI, Department of Electronics, Telecommunications and Informatics, University of Aveiro, Portugal and [3]DoV, Department of Virology, University of Helsinki, Finland

[*]Correspondence address. Jorge M. Silva and Diogo Pratas. E-mail: jorge.miguel.ferreira.silva@ua.pt https://orcid.org/0000-0002-6331-6091 and pratas@ua.pt http://orcid.org/0000-0003-1176-552X

Abstract

Background: The vast number of viral genome sequences generated during the latest pandemic has presented new challenges for computational analysis. Analyzing millions of viral genomes in multi-FASTA format is computationally demanding, especially when using alignment-based methods. Most existing methods are not designed to handle such large datasets, often requiring the analysis to be divided into smaller parts to obtain results using available computational resources.
Findings: We introduce AltaiR, a toolkit for analyzing multiple sequences in multi-FASTA format using exclusively alignment-free methodologies. AltaiR enables the identification of singularity and similarity patterns within sequences and computes static and temporal dynamics without restrictions on the number or size of input sequences. It automatically filters low-quality, biased, or deviant data. We demonstrate AltaiR's capabilities by analyzing more than 1.5 million full SARS-CoV-2 sequences, revealing interesting observations regarding viral genome characteristics over time, such as shifts in nucleotide composition, decreases in average sequence complexity, and the evolution of the smallest sequences not found in the human host.
Conclusions: AltaiR can identify temporal characteristics and trends in large numbers of sequences, making it ideal for scenarios involving endemic or epidemic outbreaks with vast amounts of available sequence data. Implemented in C with multi-threading and methodological optimizations, AltaiR is computationally efficient, flexible, and dependency-free. It accepts any sequence in FASTA format, including amino acid sequences. The complete toolkit is freely available at https://github.com/cobilab/altair.

Key words: Alignment-free toolkit; Relative Absent Words; Data compression; Temporal patterns; Viral genomes; multi-FASTA;

Introduction

The recent availability of millions of SARS-CoV-2 complete genomes that emerged from the COVID-19 pandemic has remarkably changed the scientific workflow. It starts with the parallelization of the genome's sequencing and assembly through many scientific and industrial entities, followed by the centralized upload of each SARS-CoV-2 assembled sequence in FASTA format and the respective metadata, while respecting a light data curation, mainly in the following database repositories: GISAID [1], NCBI [2], and INSDC [3], that include GeneBank [4], ENA [5], and DDBJ [6].

Although the incredible growing pace at which these complete genomes emerged and have been made publicly available, their downstream analysis faces new challenges mainly related to the vast number of genomes, characteristics and format of the data, and multiplexed sequencing-reconstruction.

Specifically, one challenge emerged due to the availability of millions of genomes split by the respective headers with basic information in a single file. This file format is known as Multi-FASTA, but it is generically called FASTA. There are alternative and more efficient file format representations, such as those representing only differences according to a reference. Still, the majority of the downstream analysis tools require the FASTA format. Moreover, storing only the differences would require multiple references to perform additional analyses at different taxonomic levels. The alternative way of using a single reference sequence becomes inefficient,

Compiled on: June 18, 2024.
Draft manuscript prepared by the author.

if there is high dissimilarity.

Furthermore, for processing up to a hundred thousand genomes, only a minority of the tools are prepared, while the availability of these tools is very scarce to process millions. The main reason is these programs' use of temporary files to decrease the computational memory and related computational time. However, creating temporary files times millions of entries is not efficiently affordable.

Another challenge is the existence of outlier or recombinant genomes in the set. The outlier entries are unwanted sequences uploaded by entities that show profound differences in sequencing and assembly methodologies. Examples of these features are the presence/absence of targeted capture, the assembly methodology using appropriate/inappropriate references, proper/improper exclusion of contaminants, accurate/inaccurate variant call, accurate/inaccurate base masking, or substantial differences in SNPs caused by postmortem degradation. Although several directives have been provided [7, 8], these genomes still exist and are continuously being uploaded. Recombinant genomes usually show a higher degree of variants or chimeric formations after cell co-infection [9]. Due to these temporary increases in the accumulation of genomic variations, recombinant genomes can be associated with the emergence of outbreak strains, as exemplified by coronaviruses and rhinoviruses [10, 11]. For downstream analysis, the objective is to discard outliers while maintaining the recombinant genomes. However, we currently lack fast genome filtering tools that assert a particular trait distribution to minimize outliers in the data while maintaining the average genome data of these recombinant genomes.

Beyond the SARS-CoV-2 sequences, the upload of large quantities of viral genomes in FASTA format is also substantially increasing. Because viral genomes are sometimes directly associated with hosting health conditions, namely in cancer or autoimmune diseases, the number of these reconstructed genomes is expected to increase dramatically for clinical and forensic purposes [12, 13, 14]. Some examples are the steadily increasing availability of Parvoviruses, Polyomaviruses, Herpesviruses, and Papillomaviruses [15, 16, 17]. Moreover, this increase is also in other types of organisms, such as organisms with larger genome sequences, namely fungi and bacteria [18], especially now with the higher rates of antibiotic resistance and the availability of complete genome sequences [19, 20] associated with the development of the Telomere-2-Telomere (T2T) technology [21, 22].

The current disposal of the massive number of genomes from a single species also provides the opportunity to study a species sequence over time, namely the temporal patterns and characteristics of the genomes or proteomes in a temporal dimension. Accordingly, the following questions emerge:

- How does the nucleotide composition of the genomes change over time?
- How does the sequence entropy of a species change in time?
- How does the similarity of parasitic sequence species vary according to the first known sequence?
- What are the shortest sub-sequences of a parasitic species not in a host?
- How do these shortest sequences change in time?

These complex questions can be answered with this type of data in synergy with the alignment-free method that is provided in this manuscript.

Although alignment methods offer an intuitive and enhanced local resolution that prevails in comparative analysis of specific features in a low number of sequences, large-scale sequence quantities require unfeasible computational resources under a desirable accuracy, limiting their applicability in multiple temporal analyses. On the other hand, the substantial increase in the development and availability of alignment-free methods [23, 24, 25, 26, 27] have provided clear advantages using feasible computational resources.

In the literature, there are multiple toolkits for specific sequence transformation and analyses applications, namely microbiome tools for forensic science [28], for machine-learning analysis and modelling of genomic and proteomic sequence data [29], for visualization of regulatory DNA motif identification and analyses [30], efficient analysis of DNA methylation [31], among many others. On the other hand, there are toolkits or platforms with a much broader application, namely SeqAn [32], khmer [33, 34], GTO [35], GATB [36], Mutalisk [37], CGAT [38], CGtag [39], nanoGalaxy [40], AlcoR [41], Poretools [42], Pycogent [43], SeqKit [44], FASTAptamer [45], fairseq [46], SeqKit [44], TBtools [47], MPI bioinformatics toolkit [48], KBase [49], among many others. Some of these toolkits or platforms are interactive through web browsers offering friendly environments, while others are prone to fast and efficient computation through the command line.

Benchmarking genomic toolkits by direct comparison can be misleading due to their distinctive design philosophies and unique feature sets. Each toolkit is crafted with specific goals in mind, which may not align perfectly with those of another. Hence, simple side-by-side comparisons might not only be uninformative but could obscure the individual strengths of each tool. Instead, a more nuanced approach involves evaluating a toolkit based on its specific features and objectives. Important aspects to consider include the toolkit's innovative features, the research questions it facilitates, its efficiency and ease of use, and any software dependencies it might have. These criteria form the core of high-level benchmarking, focusing on what each toolkit brings to the scientific community rather than how it matches up against others.

On a deeper level, benchmarking should employ both synthetic data, which provides a controlled environment to assess tool performance predictably, and natural data, which offers real-world challenges. Synthetic data testing is essential and should be a standard preliminary test, whereas natural data testing, although occasionally challenging due to the variability of biological data, is crucial for understanding how the tool performs under realistic conditions. Additionally, the reproducibility and, when feasible, the repeatability of results are paramount to confirm the reliability and effectiveness of each tool. This rigorous approach ensures that the tools not only meet theoretical expectations but also hold up under practical application.

In this article, we present AltaiR, a toolkit with alignment-free methods for the temporal analysis of multi-FASTA data, specifically large-scale numbers of genomes or proteomes, including millions (or billions) of sequences in a single FASTA file. The AltaiR toolkit offers an efficient and convenient approach for pathogen-host analyses in endemic or pandemic scenarios (but not limited to). Importantly, the AltaiR method includes a flexible tool to filter sequences undesired from the dataset through parameterized characteristics. AltaiR contains both reference-free and reference-based tools. The reference-free tools are for analysing sequence entropy and nucleotide frequency changes over time. The reference-based tools are divided into two main wings, similarity and singularity. For both, highly efficient implementations are provided. The AltaiR is developed in C language and is provided as open-source software. In the next section, we enumerate the features and characteristics of the AltaiR toolkit, including the description and formalization of the methods. Then, we benchmark each tool from the toolkit using synthetic data while ensuring the full repeatability of the experiment. Afterwards, we present results using natural data, namely an application for a vast SARS-CoV-2 set of genomes. Finally, we discuss the results obtained and draw some conclusions.

Methods

This section describes the methods and their respective implementation into computer tools that constitute the AltaiR toolkit. The details of the parameters and how to reproduce the methods are

available in Supplementary Sections 2 and 3. We recommend using compressed data, namely through the compression of the files using specialized tools. For fast access, NAF [50], AGC [51], and, for bacteria, MBGC [52] can be used. For a higher compression ratio but slower access, we recommend MFCompress [53]. When using sensitive data, Cryfa [54] can be used to compact and encrypt the data.

Figure 1 describes the workflow of AltaiR by dividing the data according to three types: the parasite reference sequences, the parasite sequences (after filtering the outliers or unwanted data), and the host omics sequences. The workflow contains seven main methods.

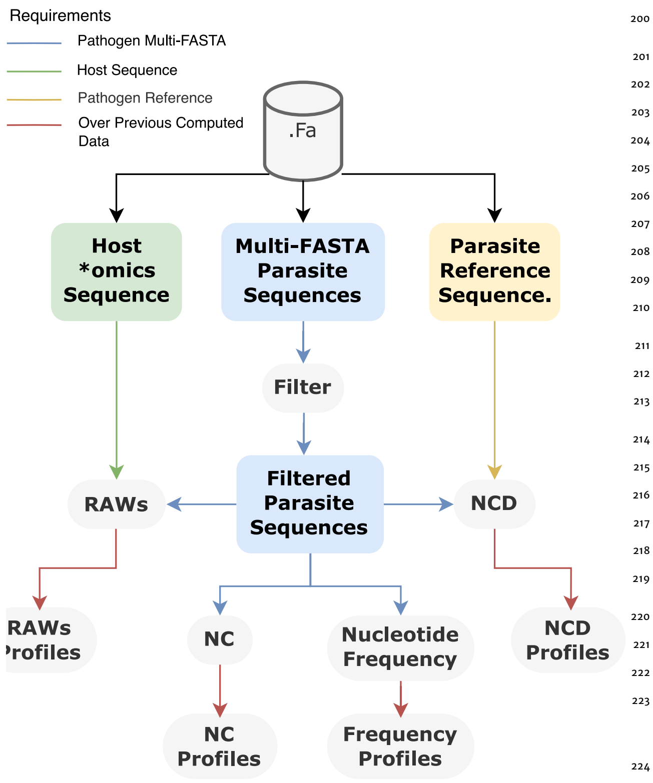

Figure 1. Workflow of the AltaiR toolkit describing the principal seven phases along with the three input types of sequences in FASTA format.

The first method is filtering the sequences by characteristics, essentially removing sequences that contain different traits from what is expected or the average. The second method is the Normalized Compression Distance (NCD) for measuring the similarity between two sequences. The NCD extension is provided in the third method through the use of the NCD profiles. These are generated by the similarity comparison of the existent sequences according to a reference. The fourth method is the Normalized Compression (NC) profiles that depict the compression variability of the sequences. The fifth method is the frequency profiles, allowing to visualize changes in the nucleotide composition over time. The sixth method is the RAWs (Relative Absent Words) mapping that identifies the shortest sequences in the parasite but absent from the host genome/transcriptome. Finally, the seventh method provides profiles regarding the RAWs according to time.

The further subsections provide details on the methods, including their mathematical formalizations and characteristics.

Filter sequences by characteristics

The public databases of multiple genome and proteome sequences from a specific organism usually contain sequences not within the average group. The most frequent reasons are the inclusion of contaminant sequences [55, 56] and differences in the sequencing or assembly procedures. Removing some of these sequences from the dataset is important for downstream analyses. However, manual inspection is slow and can introduce errors. Therefore, automatically filtering these sequences by characteristics is important and can provide unbiased selections while maintaining visible static and uniform characteristics. AltaiR incorporates established guidelines and best practices [7, 8] to address potential contamination and quality issues through automated filtering. This process ensures only high-quality, contiguous sequences are included in the analysis, minimizing bias from incomplete or contaminated data.

Accordingly, this sub-method filters a multi-FASTA file, specifically FASTA reads, by input characteristics. These characteristics can be the alphabet composition, sequence completeness or length, GC quantity, multiple string header patterns, or absence of patterns (anti-patterns). Completeness refers to the availability of the full, uninterrupted sequence of an organism's genome, rather than just partial or fragmented sequences. The computation can be applied to different sequences; for example, a specific filtering can be applied to SARS-CoV-2 while another for B19V. Moreover, the filtering characteristics can be intersected through a single run.

Consider a source that has generated n sequences from a finite alphabet Ξ with size $|\Xi|$. The nature of the source is unknown but in each sequence x^i, where $x^0, ..., x^n$, the internal symbols are known.

The filtering aims to evaluate specific characteristics that occur in each x^i, and those which respect the conditions are used for further downstream analyses. Moreover, each x^i contains a header alphanumeric sequence, y^i, that generically identifies x_i. Specifically, the y^i is the metadata of x^i and usually contains the name of the organism, the date, and a unique ID, among others.

The following definitions provide information on the filtering for each main characteristic or feature, including the sequence length, alphabet composition, minimum and maximum GC percentage, header patterns and anti-patterns.

Sequence length

In multiple cases, sequences labelled as complete are nearly complete, or the length is shorter than a certain length. This can occur, for instance, due to the sequencing depth being very low or some scaffolds could not be linked in the assembly process. AltaiR can filter sequences by the minimal and maximal length to minimise these issues. Therefore, for any string x^i with length $|x^i|$ the set of final sequences, Σ, is composed by

$$x^i \in \Sigma \iff \mathcal{N}_{\max} \geq \mathcal{N}(x^i) \geq \mathcal{N}_{\min}, \tag{1}$$

where $\mathcal{N}$ is the length of the sequence x_i and $\mathcal{N}_{\min}$ and $\mathcal{N}_{\max}$ the minimum and maximum length, respectively.

Alphabet composition

It is common to find some reads of a multi-FASTA format containing characters outside a desired set. For example, the 'R' symbol at the DNA sequence level stands for a purine (an A or G base). This characteristic is frequent with other symbols in protein sequences, leading to an unbalance between the set cardinality of the sequences. This ambiguity generates complexity in the analysis when using many software tools and can create deviant results. Therefore, the AltaiR method efficiently filters sequence symbols containing a certain alphabet. Additionally, it can filter the reads by composition, including completeness, when combined with the length.

Minimum and maximum GC percentage

The GC percentage is usually an important feature to evaluate if the distribution of a particular sequence is within a desirable average set. Specifically, the GC percentage is given by the number of cytosine (C) and guanine (G) bases in a string x^i with length $|x^i|$ according to

$$\mathcal{GC}(x^i) = \frac{100}{|x^i|} \sum_{j=1}^{|x^i|} \mathcal{I}_{\Xi}(x^i_j), \quad (2)$$

where x^i_j is each symbol of x^i (assuming causal order), Ξ is a subset alphabet containing the symbols $\{G, C\}$ and $\mathcal{I}$ an indicator function constrained to

$$\mathcal{I}_{\Xi}(x) = \begin{cases} 1, x \in \Xi \\ 0, x \notin \Xi \end{cases} . \quad (3)$$

Then, the set of final sequences, Σ, is composed by

$$x^i \in \Sigma \iff \mathcal{GC}_{\max} \geq \mathcal{GC}(x^i) \geq \mathcal{GC}_{\min}, \quad (4)$$

where $\mathcal{GC}_{\min}$ and $\mathcal{GC}_{\max}$ are two real values where $\mathcal{GC}_{\max} > \mathcal{GC}_{\min} \in [0; 100]$.

Header patterns and anti-patterns

The header patterns are strings to match in each sequence header, y^i, that can ignore or consider a certain sequence read if the substring pattern is absent or present. This is the analogous process of the well-known grep tool, but instead of looking into the whole FASTA file, it only filters by the header sequence. This type of filtering is very important when only a certain type of sequence is to be considered or sequences with a certain header substring must not pass to the final set of sequences, Σ.

The AltaiR toolkit provides filtering of the header patterns using substrings that occur in the header or that are absent (ignore pattern) using conjoint filtering that is not limited to the number of strings to match or ignore. For example, using the patterns "Human", "Herpes" and "Alpha" and the anti-pattern "Simplex" in the whole viral reference database from the NCBI will select all the FASTA reads that the headers contain the words Human, Herpes, and Alpha, selecting only the human Alphaherpesvirus that are composed by the HSV-1 (Herpes Simplex Virus 1), HSV-2 (Herpes Simplex Virus 2), and VZV (Varicella-Zoster Virus); because the anti-pattern "Simplex" was also used, then, only the VZV will be contained in Σ.

Similarity (NCD) profiles

In temporal analysis, some applications require measuring the similarity of the most recent genomes to the first ones being sequenced and assembled, such as the first reference genome. These measures will allow us to understand the evolution rate over time and if evolutionary acceleration peaks have been found in specific periods. Accordingly, if the genomes are sorted according to sample isolation date (an NCBI VSSI option while downloading the data), then measuring the distance of the first genome according to the remaining in the multi-FASTA file provides this similarity information.

To compute the similarity between the reference genome and the remaining sequences, the Normalized Compression Distance (NCD), which is a similarity distance that approximates the Kolmogorov complexity through data compression, is used [57, 58, 59]. The NCD has many applications for genomic and proteomic assembled sequences [60], including the similarity measure for the COVID-19 pandemics [61, 62].

Formally, the NCD between a reference y and a target x sequence is given by

$$\mathcal{D}(x, y) = \frac{C(x, y) - \min\{C(x), C(y)\}}{\max\{C(x), C(y)\}}, \quad (5)$$

where $C(x)$ and $C(y)$ represents the number of bits needed to lossless compress x and y, respectively. The $C(x, y)$ represents the number of bits needed to compress x and y conjointly, which usually is approximated by string concatenation.

Since in our application, we are required to compute the whole distances of each x^i according to a reference y, then an array of distances is calculated for creating the NCD profile according to

$$\mathcal{D}_i(x^i, y) = \frac{C(x^i, y) - \min\{C(x^i), C(y)\}}{\max\{C(x^i), C(y)\}}. \quad (6)$$

Moreover, because the conjoint compression follows the commutative property $C(x^i, y) = C(y, x^i)$, it can be rewritten as

$$\mathcal{D}_i(x^i, y) = \frac{C(y, x^i) - \min\{C(x^i), C(y)\}}{\max\{C(x^i), C(y)\}}. \quad (7)$$

This change offers a substantial save in computational resources because it is now possible to compress y and, in the end, freeze its models. Then, the number of bits to compress y is saved, and for each x^i, the compression models are initialized with the frozen models of y. Therefore the complexity time to compute $C(y, x^1), ..., C(y, x^n)$ is now $y + xn$. This change is now affordable for an application considering millions of genomes.

The choice of the data compressor is fundamental to better approximating the Kolmogorov complexity and the NCD [63, 58]. Therefore, besides respecting the common distance characteristics [63] and distance density [58], the data compressors that are designed for the specific use of certain types of data offer a much higher approximation of the Kolmogorov complexity than general-purpose tools. This characteristic is provided by the specific-purpose methodology's ability to efficiently model characteristics that would otherwise not be seen with general-purpose models. For example, two characteristics that play a key role in biological sequences are inverted repeats [64] and high-level substitutions in repetitive data [65].

In the case of the COVID-19 pandemic, a specific-purpose data compressor does not offer a wide advantage over a general-purpose data compressor because, on average, the sequences contain high entropy, are small, and have only a few sequence differences between sequences. However, an efficient specific-data compressor is mandatory for larger genomes such as those from larger viruses (e.g. Herpesvirus), bacterial or fungi. Therefore, we use an implementation derived from GeCo3 [66] and AC2 [62] data compressors that have proven to be state-of-the-art data compressors in genomic and proteomic sequences, respectively. The disadvantage of these data compressors is the necessity to have a solid knowledge of the models for extensive optimization. We provide pre-computed models for different biological sequence types to minimise this disadvantage.

For applications where the size of the distances vector is very large or the variation of the instances in the profile high, AltaiR offers the possibility of averaging the signal through a moving average. The Toolkit implementation subsection provides more information about this option.

Complexity (NC) Profiles

Another important temporal analysis is the perception of how the (normalized) Kolmogorov complexity [59] of a certain set of sequences sorted by time varies. The normalized Kolmogorov complexity is approximated through the Normalized Compression

(NC) [67]. The NC is given by the ratio of the uncompressed quantity sum of bits by the size of the sequence representation assuming a uniform distribution. This analysis is analogous to understanding how the genome or proteome sequence entropy varies over time using a random reference.

As in the previous Subsection, the Kolmogorov complexity is approximated using specific-purpose data compression algorithms, namely with implementations derived from GeCo3 [66] and AC2 [62] for genome and proteome sequences, respectively.

Formally, the NC of a certain sequence is provided by

$$\mathcal{E}(x) = \frac{C(x)}{|x| \log_2(|\Xi|)}, \quad (8)$$

where $|x|$ is the size of the sequence and $|\Xi|$ the number of different symbols in the sequence x.

Since in our application, we require to compute the whole sequence complexity of each x^i, then an array of NCs is calculated for creating the NC profile according to

$$\mathcal{E}_i(x^i) = \frac{C(x^i)}{|x^i| \log_2(|\Xi^i|)}, \quad (9)$$

where $|\Xi^i|$ is the number of different symbols in the sequence x^i.

For genomic sequences, Ξ is 4 for any x^i. However, for proteomic sequences, Ξ may vary, creating changes in the profile. For these cases, alphabet trimming, which involves reducing the alphabet size by removing or substituting non-standard characters, can work as a solution to ensure a consistent alphabet size across all sequences and maintain the comparability of NC values.

For applications where the size of the NC vector is very large or the variation of the instances in the profile high, AltaiR offers the possibility of averaging the signal through a moving average. The Toolkit implementation subsection provides more information about this option.

Frequency profiles

One application that provides evidence of evolutionary events is the variation of the nucleotide or amino acid frequency over time. This information is given by the frequency profiles, defined as the percentage symbol distribution of each sequence.

Formally, the frequency of a certain symbol, s, from Σ in a sequence x is provided by

$$\mathcal{F}(s|x) = \frac{1}{|x|} \sum_{j=1}^{|x|} \mathcal{I}_s(x_j), \quad (10)$$

where $\mathcal{I}$ is an indicator function associated to the number of times that s is seen in x.

Since in our application, we require to compute the symbol frequency of each x^i, then an array of frequencies is calculated for creating the frequency profile according to

$$\mathcal{F}_i(s|x^i) = \frac{1}{|x^i|} \sum_{j=1}^{|x^i|} \mathcal{I}_s(x^i_j). \quad (11)$$

For applications where the size of the frequency vector is very large or the variation of the instances in the profile high, AltaiR offers the possibility of averaging the signal through a moving average. The Toolkit implementation subsection provides more information about this option.

Relative singularity (RAWs) profiles

Identifying the shortest sequence regions present in a set of pathogens but absent from the host genome and transcriptome is an important application in endemic and epidemic contexts. These regions, called Relative Absent Words (RAWs), are of interest because they can distinguish between lineages [68], localize higher GC-content regions, and have applications in optimized diagnosis [69]. Furthermore, RAWs can provide insights into the unique genomic characteristics of pathogens, potentially aiding in the development of targeted therapeutic interventions and diagnostic tools [69].

Specifically, RAWs [68, 69, 70] are a particular subset of Minimal Absent Words (MAWs) that have also been referred to as nullomers or forbidden words [71, 72, 73]. Many MAWs-based applications have been created, including optimized models for their detection [74] and the complementary simulation of sequences while avoiding the creation of MAWs [75].

MAWs in genomic and proteomic sequences have been studied [76]. For example, in viruses, MAWs have been found most frequently in regions that are restriction recognition sites [76]. On the other hand, the subset of MAWs (RAWs) has been found in regions with high GC content, contrary to the host and pathogen genome [69].

An approach to finding MAWs is through the flanking subsequence regions. Consider a set, X, constituted of n target sequences, $x^1, x^{\cdots}, x^n$, and a reference sequence, y, both drawn from the finite alphabet Σ. We say that β is a factor of x^i if x^i can be expressed as $x^i = u\beta v$, with uv denoting the concatenation between sequences u and v. We denote by $\mathcal{W}_k(x^i)$ the set of all k-size words (or factors) of x^i. Also, we represent the set of all k-size words *not in* x^i as $\overline{\mathcal{W}_k(x^i)}$. For each word size k, we define the set of all words that exist in x^i but do not exist in y by

$$\mathcal{R}_k(x^i, \overline{y}) = \mathcal{W}_k(x^i) \cap \overline{\mathcal{W}_k(y)}. \quad (12)$$

The subset of minimal words as

$$\mathcal{M}_k(x^i, \overline{y}) = \{\beta \in \mathcal{R}_k(x^i, \overline{y}) : \mathcal{W}_{k-1}(\beta) \cap \mathcal{M}_{k-1}(x^i, \overline{y}) = \emptyset\}, \quad (13)$$

i.e., a MAW of size k cannot contain any MAW of size less than k. In particular, $l\beta r$ is a MAW of sequence x^i, where l and r are single letters from Σ, if $l\beta r$ is not a word of x^i but both $l\beta$ and βr are. We have defined the non-empty set $\mathcal{M}_k(x^i, \overline{y})$ with the smallest k as minimal Relative Absent Words (mRAWs) [68].

Another subset of MAWs and RAWs are persistent mRAWs (PmRAWs) [69]. Formally, let r be a mRAW of $x^i \in X$ and $P(r, x^i)$ be the predicate "r is a RAW of string x^i". Then, if $\forall_{x^i \in X} P(r, x^i)$, we say that r is persistent in X. This property means the full conservation of the identified mRAWs across all the sequences. A particularity of these sequences is to consider PmRAWs at the whole genome level and at a sub-genome level, meaning that a x^i can be considered a subsequence of a whole genome, namely a gene, extending the power of PmRAWs to local observations.

The RAWs and PmRAWs have been computed with the EAGLE tools version 1 [68] and 2 for DNA sequences [69]. This methodology was included in the online detection of RAWs, namely the ADACT [77].

The AltaiR toolkit can compute the RAWs based on the same methodology as in EAGLE version 2. Still, it extends the capability to deal with any other types of sequences, for example, proteomes, as long as they respect the multi-FASTA format. Moreover, for computing the RAWs, the AltaiR toolkit can avoid writing into temporary files while maintaining low memory consumption. This capability enables the computation of RAWs in millions of genomes/proteomes while expending relatively low computational resources.

Moreover, the Altair toolkit enables the automatic computation

of the GC percentage for each RAW, providing the unprecedented ability to create large-scale studies with a higher divergence between the sequences.

Additionally, the AltaiR toolkit includes the capability to compute RAWs profiles. The RAWs profiles are generated with the additional combination of temporal metadata, namely through the time order usage of the target multi-FASTA file that can be downloaded with that property at the NCBI repository. These profiles directly compute the presence of each RAW according to the sequences sorted by temporal characterization.

Toolkit implementation

The AltaiR methodology is implemented in C language and contains no external dependencies. The source code and application result scripts are freely provided at the repository https://github.com/cobilab/altair. The AltaiR toolkit contains one main menu (command: AltaiR) with six sub-menus for computing the methods that it provides, sometimes through the combination of multiple, namely

- **average** - moving average filter of a column float CSV file (the column to use is a parameter);
- **filter** - filters FASTA reads by characteristics: alphabet, completeness, length, GC quantity, multiple string patterns and anti-patterns;
- **frequency** - computes the alphabet frequencies for each FASTA read (it enables alphabet filtering);
- **nc** - computes the Normalized Compression (NC) for all FASTA reads according to a compression level or specific parameters;
- **ncd** - computes the Normalized Compression Distance (NCD) for all FASTA reads according to a reference;
- **raw** - Computes Relative Absent Words (RAWs) with automatic GC percentage estimation for all RAWs.

The toolkit allows reading and writing from standard input and output, respectively, in certain cases. For example, this feature enables the direct piping of the output from the filtering tool to the NC, NCD, and RAWs tools, streamlining the analysis process. Additionally, it allows for the output profiles of the frequency, NC, NCD, and RAWs tools to be easily processed by other tools, such as those performing averaging or visualization. This functionality provides easier integration to build custom pipelines. Furthermore, most tools (except average) use multi-threading to speed up the analysis.

In the next section (results), we provide details of the toolkit features, including the most important parameters and commands to retrieve the results. Nevertheless, more documentation can be retrieved from the Supplementary material and in the README.md file in the code repository.

Results

As a result, we provide the application analysis of the AltaiR toolkit to the challenges described in the previous section. The primary motivation for using the SARS-CoV-2 data is to demonstrate AltaiR's capabilities in handling large-scale genomic datasets and uncover novel insights into the virus's evolution, adaptation, and interaction with the human host. All the results can be repeated using the procedures described in each specific analysis, except for the data collection retrieved manually from the NCBI Virus database. Despite this manual procedure, all the data is included as supplementary material for direct download. The dataset used to compute the results includes a large collection of SARS-CoV-2 genome sequences retrieved from the NCBI viral repository on 29 September 2022 [2]. The SARS-CoV-2 genomes have been filtered from a pool of 6,309,078 genomes according to quality and completeness, namely only considering complete genomes with the host as human, resulting in 1,538,095 genomes [78]. The dataset also contains multiple coronavirus genomes, the reference T2T human genome and transcriptome, and multiple computer-generated sequences; the latter works as an important validation procedure.

Filtering sequences

The SARS-CoV-2 dataset contains nearly 1.5 million sequences labelled as complete genomes in the NCBI viral repository. In the histogram depicted in Figure 2, we notice substantial differences in the length of the sequences. This histogram provides the frequency of the length of the sequences, which was computed considering symbols outside the alphabet $\Sigma = \{A, C, G, T\}$, such as the N symbols and others. Although the plot considers only the sequence length from 29.5k and 30k, there were several sequences with lengths lower than 20k. These smaller sequences were considered complete genomes and showcased the importance of filtering the data by the sequence length.

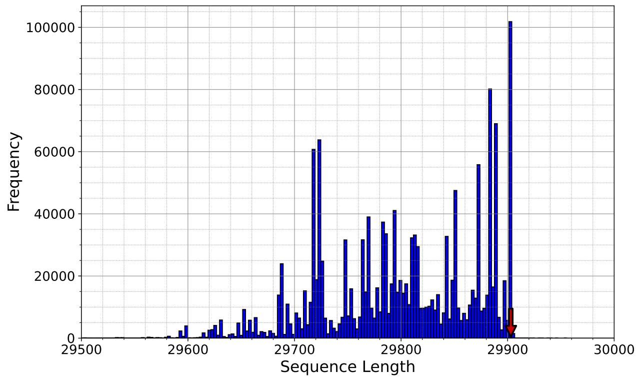

Figure 2. Histogram with the length of the genomes, considering all the existent symbols, from the SARS-CoV-2 dataset. The red arrow line stands for the size of the reference genome. To replicate this analysis, see Supplementary Section Reproducibility, specifically Reproducing the "Data filtering".

The SARS-CoV-2 reference genome (NC_045512.2) has a length of 29,903, and the largest cluster of sequences is almost coincident with this length (around 100k sequences). Therefore, since the SARS-CoV-2 genomes have low variability, this region is a good candidate for sequence length filtering.

However, the SARS-CoV-2 genomes contain a poly(A)-tale with a variable size in the end-tip. This poly(A)-tale is the only low-complexity region and, therefore, it is, ironically, the region more complex to sequence and assemble [69, 41]. To provide some flexibility to this region, we filtered the sequences considering the interval length of 29,885 to 29,921. Notice that many of these sequences contained symbols outside the $\{A, C, G, T\}$ alphabet, for example, N symbols, and, thus, many of them were removed when the total of alphabet symbols was not reached.

Moreover, we included filtering specific patterns and anti-patterns, namely considering only the sequences where the headers did not contain only the year (e.g. "|2020|") but also the month and, if available, the day associated with the virus isolation. Also, sequences without dates were discarded.

In sum, the sequence length and the pattern/anti-pattern decreased by 1.5 million to 25,594 sequences. Although the dataset was reduced substantially, the data quality substantially increased proportionally. Moreover, nearly 25k of high-quality sequences are sufficient for accurate downstream analysis.

This sequence filtering step shows its critical importance,

proven by the high variability between these sequences, which can harm downstream analyses without proper filtering. For instance, including incomplete, low-quality, or outlier sequences can introduce noise and bias in similarity measurements, leading to inaccurate clustering or phylogenetic inference. Inconsistent or missing metadata can hinder the interpretation of temporal patterns and evolutionary trends, while unusual sequence characteristics can skew statistical analyses and obscure biological patterns. As such, rigorous filtering ensures high data quality and consistency, minimizing spurious results and enabling reliable biological interpretations.

Similarity (NCD) profiles application

To benchmark the similarity profiles described in the Subsection Similarity (NCD) profiles, we recurred to two levels, namely, using synthetic and real data.

The synthetic data (denoted as the original sequence) has been computer-generated with the GTO toolkit [35] while assuming a uniform distribution and a length of 5000 DNA symbols. Using this original sequence, consecutive SNP mutations have been applied to the following sequences with a symbol mutation probability of 0.00005. To clarify, the original sequence (time-0) has been mutated while originating the sequence time-1, then the sequence time-1 has been mutated originating the sequence time-2, and this process was followed to the sequence time-10000. Finally, the temporal similarity profiles have been computed using a chosen time point as a reference. Supplementary Figure 1 provides the similarity (NCD) profiles for three time points, namely 0, 2500, and 4000.As depicted, the NCD profiles have a minimum value for the respective time point reference, showing the capability to identify the closest sequence in time under these simplified conditions.

Despite the performance of the previous approach, it is hard to find a real scenario where a time point sequence is perfectly available. In practice, this sequence already contains several mutations. Therefore, to simulate this characteristic, we repeat the above experience, but instead of using a direct time point sequence, we use a mutated time point sequence.

Accordingly, all the time point symbol sequences have been mutated with a probability of 0.05 (mutations in approximately 5% of the sequence) using a uniform distribution and different seeds. Supplementary Figure S2 provides the similarity (NCD) profiles for three-time points mutated, namely 0, 2500, and 4000. Although the average NCD value increased, the similarity is still comparatively high for the selected points. These results suggest that NCD profiles may serve as tolerant mutation measures to predict the temporal occurrence of sequences, but further investigation is required to validate this claim.

In addition to the synthetic data analysis, we also explored the application of NCD profiles to a real-world dataset, specifically focusing on the RaTG13 genome sequence and its similarity to SARS-CoV-2 sequences. This analysis, along with a discussion of its limitations and potential implications, can be found in the Supplementary Material. Applying NCD profiles to this real-world dataset demonstrates their ability to uncover interesting trends and relationships that can guide further research. While the results obtained from NCD profiles should be interpreted cautiously and validated through additional lines of evidence, they serve as a valuable starting point for more focused investigations and hypothesis generation.

It's also worth mentioning that from any NCD results, one can automatically construct a phylogenetic tree for the n closest sequences. The Supplementary Material under the Reproducibility Section illustrates an example of this tree, along with instructions for recreating it.

Complexity profiles (NC) application

Herein, we present a detailed complexity profile (NC) analysis of SARS-CoV-2 sequences using the AltaiR toolkit. By approximating the normalized Kolmogorov complexity through the Normalized Compression metric, we quantitatively assessed the informational content of the viral sequences over time.

The complexity profile, illustrated in Figure 3, captures the temporal fluctuations in the sequence complexity of the virus. The NC values were computed using 5, 20, and 100 window sizes to smooth the data and reveal underlying trends. The plot shows the NC trajectory, where each line represents the complexity calculated over a different window size.

The time frame from January 2020 to January 2022 was particularly interesting due to significant epidemiological events. Our analysis encapsulates this period, showing a dynamic yet subtle evolution of the virus's genomic makeup, namely an average decrease of sequence complexity over time. Usually, to accomplish very similar functions, lower entropy corresponds to less energy required and, hence, more efficiency which can be driven by evolution or selection.

Moreover, the plot indicates periods of relative stability interspersed with spikes and dips in complexity. Notice that some noise exists that can be related to the higher variability of the size of some sequences but that is contained in the filtered interval. On the other hand, some inflexion points may correlate with the emergence of new variants or adaptations in the virus's evolutionary strategy.

Recently, we introduced AlcoR [41]. AlcoR is a mapping and visualization tool for detecting low-complexity regions in biological data. AltaiR complements AlcoR in the sequence complexity analysis, namely by adding the capability to relate sequences using a temporal dimension.

Frequency profiles application

We used the filtered SARS-CoV-2 multi-FASTA to access the frequency application and computed its nucleotide variation over time, as shown in Figure 4.

The figure shows a notable shift in the nucleotide composition of SARS-CoV-2 over the observed period of time. Specifically, the frequency of thymine (T) bases has gradually increased, while there has been a (approximate) corresponding decrease in the frequency of cytosine (C) bases. This trend indicates the virus's ongoing evolutionary process within the human host.

The increase in T bases and the decrease in C bases can be attributed to various factors. Transition mutations, where a purine is substituted for another purine or a pyrimidine for another pyrimidine (in this case, C to T), are common mutations in RNA viruses. These mutations may be driven by the error-prone nature of RNA-dependent RNA polymerase, which is responsible for viral replication [79, 80, 81]. Additionally, such mutations may confer selective advantages to the virus, potentially impacting its transmissibility, pathogenicity, and immune escape capabilities [82, 83].

This trend also raises important considerations for public health and clinical interventions. For instance, changes in the viral genome could affect the efficacy of vaccines and therapeutics, emphasizing the need for continuous surveillance and adaptation of these measures [84, 85]. The observed mutations might also provide insights into the virus-host interaction dynamics, explaining how SARS-CoV-2 adapts to the human host environment over time [86].

Relative singularity (RAWs) profiles

Our study also focused on uncovering the Relative Absent Words (RAWs) of SARS-CoV-2, namely by identifying the shortest words that exist in the SARS-CoV-2 and were absent from the human

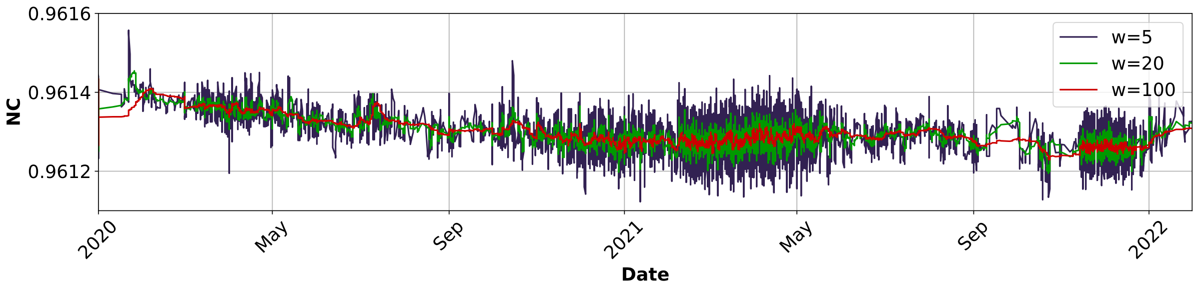

Figure 3. Complexity Plot showing variations in the normalized complexity (NC) values over time. The plot illustrates changes in NC with different window sizes, providing insights into the data trends from 2020 to 2022. To replicate this analysis, see Supplementary Section Reproducibility, specifically Reproducing the "Complexity profiles (NC) application"

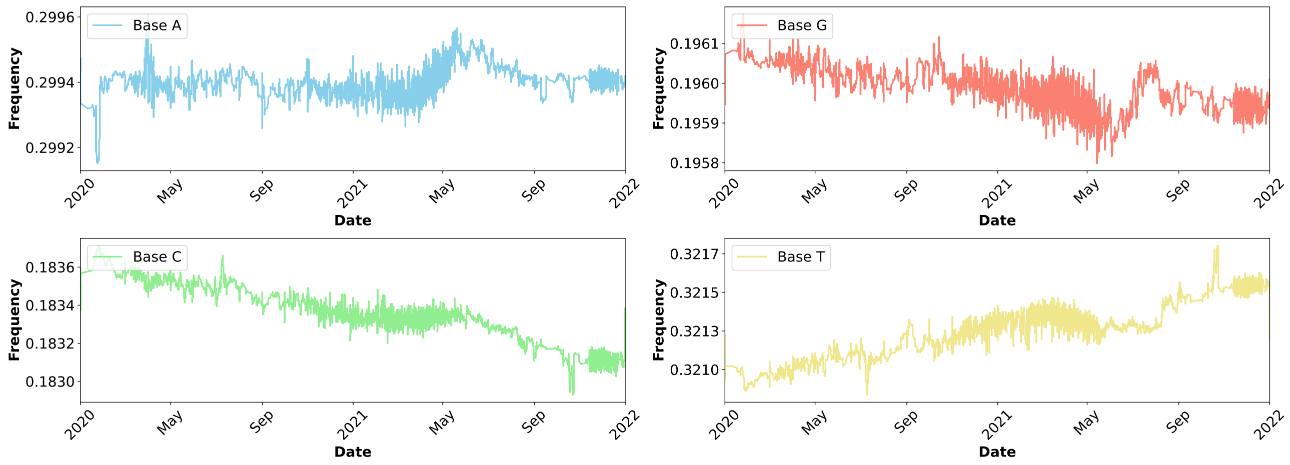

Figure 4. Frequency distribution of nucleotide bases in SARS-CoV-2 from 2020 to 2022, illustrating an increase in thymine (T) bases and a decrease in cytosine (C) bases. To replicate this analysis, see Supplementary Section Reproducibility, specifically Reproducing the "Frequency profiles application"

host genome [87] and transcriptome [88]. Detailed statistics of this analysis are presented in Table 1.

When analyzing the data in the table, it becomes evident that there is a notable shift in nucleotide distribution as the mRAW size increases in the SARS-CoV-2 genome. Specifically, we observe a decrease in guanine (G) and cytosine (C) percentages in larger mRAWs, indicating a declining prevalence of GC content as the sequence size expands. This trend suggests a gradual shift towards AT-rich sequences in the larger mRAWs of the virus.

Interestingly, this finding contrasts with the overall nucleotide composition trend observed in SARS-CoV-2, where globally, GC content approximates 40%, and AT content around 60%. This discrepancy between the overall nucleotide composition of SARS-CoV-2 and the elevated GC percentage in the shortest unique sequences absent from the human genome suggests distinct evolutionary adaptations or functional requirements in the viral genome. Higher GC content in these sequences may confer increased structural stability or efficiency in replication and transcription processes, potentially impacting the virus's interaction with host cellular mechanisms. This pattern may reflect evolutionary pressures shaping these specific genomic regions for optimized functionality within the host environment [89, 90, 91].

On the other hand, from a previous study [69] we found out that some of the mRAWs with high GC content were localized at the surface of important proteins, such as the Spike glycoprotein in the SARS-CoV-2. This region was then found to be related to segments where the protein presents higher dynamics in time, namely higher movement of the protein to interact with the host cell.

Figure 5 and Supplementary Figure S4, through relative singularity profiles, illustrate temporal variations in both the count of mRAWs and their average GC-content in the SARS-CoV-2 genomes. Between June and August 2021, the plots indicate significant shifts in the SARS-CoV-2 genome's mRAWs count and GC content, coinciding with the Delta variant's dominance. The unique mutations of the Delta variant likely drove these genomic changes, reflecting its impact on the virus's nucleotide composition and structure.

The AltaiR tool's efficiency in processing large datasets proves extremely useful for identifying virus evolutionary trends. Additionally, its capability to rapidly analyze and detect mRAWs holds significant potential for diagnostics, as these sequences can be targeted explicitly in tests for accurate and prompt detection of viral infection. Moreover, mRAWs profiles can be used as potential markers for detecting new variants.

Computational efficiency

To evaluate the computational performance of the AltaiR toolkit, using only a single computational thread, we measured the average and total execution times for each of its main methods: filtering, NC calculation, frequency calculation, NCD calculation, and RAWs calculation (Table 2).

Filtering sequences based on length, GC content, and specific patterns took 0.0004 seconds per sequence. NC calculation, which generates Nucleotide Complexity profiles, required an average of 0.0050 seconds per sequence (total 128.26 seconds). Frequency calculation, analyzing nucleotide composition, was the fastest at 0.0001 seconds per sequence on average (total 3.70 seconds). NCD calculation took an average of 0.2294 seconds per sequence (total 2294.13 seconds). RAWs calculation was the most computationally intensive method, requiring an average of 61.5580 seconds per sequence and 1538.95 seconds for the entire dataset. This is expected, as the RAWs method involves complex string matching and

Table 1. Output statistics of AltaiR while computing the RAWs of SARS-CoV-2 that are absent from the human genome and transcriptome.

	Overall mRAWs Statistics			mRAWs Nucleotide Distribution				mRAWs AT/CG Distribution	
**kmer**	**Average**	**Variance**	**Std. Dev.**	**A**	**C**	**G**	**T**	**AT%**	**CG%**
11	0.00	0.00	0.01	3	3	4	1	36.4%	63.6%
12	8.29	0.39	0.62	584,523	561,473	720,587	679,229	49.6%	50.4%
13	119.70	11.26	3.36	8,500,431	10,168,609	11,774,411	9,386,300	44.9%	55.1%
14	726.01	46.92	6.85	60,566,946	65,041,615	67,333,051	67,209,832	49.1%	50.9%
15	2813.56	147.05	12.13	269,913,035	251,842,372	260,427,726	298,014,612	52.6%	47.4%
16	8132.19	227.32	15.08	900,499,378	717,952,376	740,116,031	971,728,183	56.2%	43.8%

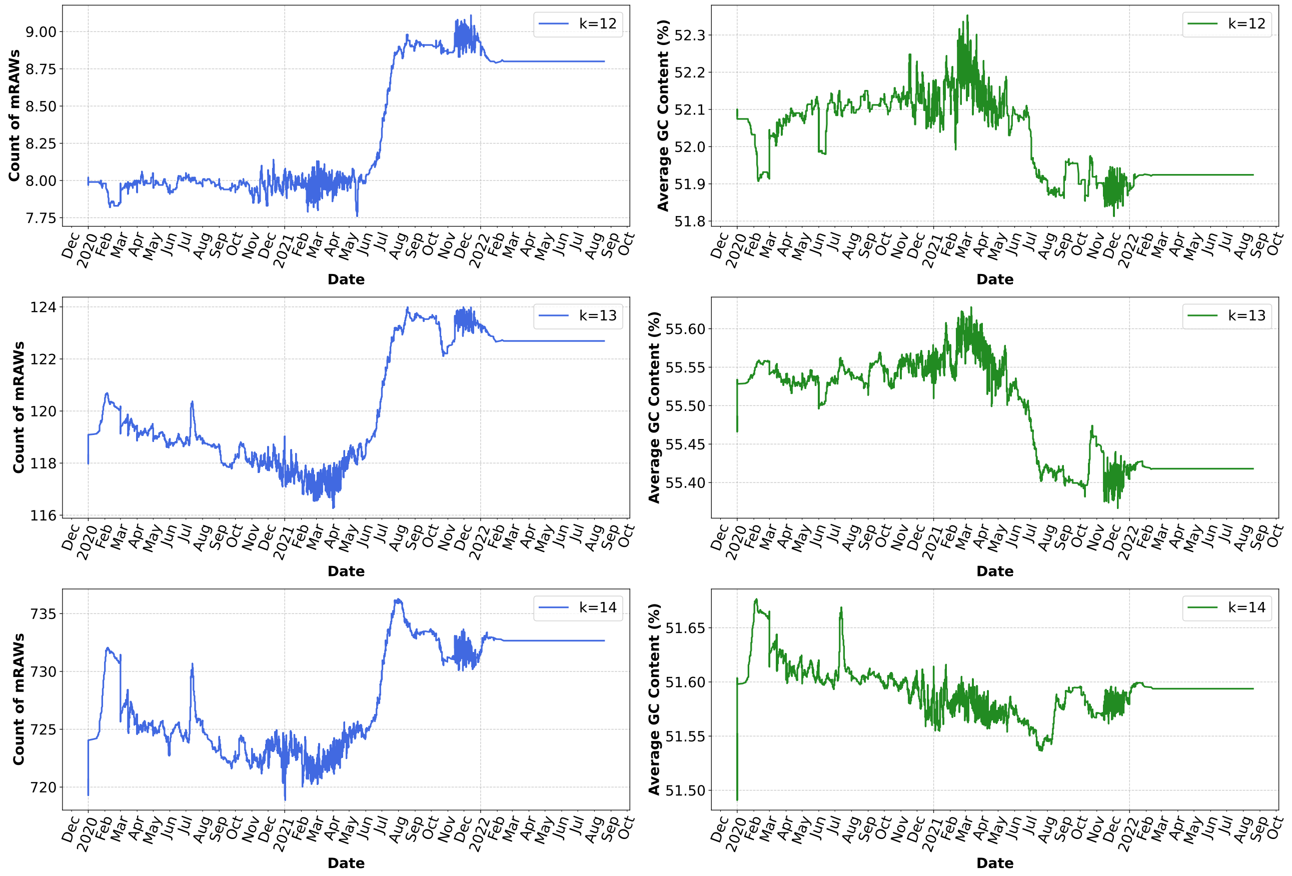

Figure 5. Relative singularity (RAWs) profiles: This set of plots illustrates the variation over time in the number of mRAWs and the average GC content in SARS-CoV-2 sequences. Each subplot corresponds to a different k-mer size (k=12, 13 and 14), showcasing the distribution of mRAWs and GC percentage across various time points. The x-axis represents time, segmented into years and months, while the y-axis shows the count of mRAWs and the GC content percentage, respectively. To replicate this analysis, see Supplementary Section Reproducibility, specifically Reproducing the "Relative singularity (RAWs) profiles"

Table 2. Average computation times for each method in the AltaiR toolkit.

Method	Average time per sequence (seconds)	Total time (seconds)
Filtering	0.0004	598.435
NC	0.005	128.2557
Frequency	0.0001	3.701
NCD	0.229	2294.132
RAWs	61.558	1538.950

comparison operations to identify unique substrings. The modular design of AltaiR allows for the integration of these methods into customizable pipelines, enabling researchers to select the most relevant analyses for their specific studies. The toolkit's ability to efficiently process large datasets, with most methods requiring less than a second per sequence on average, makes it well-suited for the rapidly growing field of genomic data analysis. While the RAWs calculation is more computationally demanding, its unique capabilities in identifying distinctive genomic elements justify its inclusion in the toolkit. These results showcase the overall computational efficiency of AltaiR, demonstrating its potential to streamline the analysis of large-scale genomic datasets across various research domains, from infectious disease studies to broader investigations in biology and genetics.

Discussion

The development of the AltaiR toolkit responds to the growing need for advanced tools capable of analyzing large and complex genomic datasets. This need arises not only from studies on infectious diseases but also from the broader fields of biology and genetics, where researchers are increasingly focusing on diverse organisms, including viruses, bacteria, protozoa, plants, and eukaryotes.

To contextualize AltaiR's capabilities, we performed a qualitative analysis with established toolkits—SeqAn [32], khmer [33, 34],

Feature / Toolkit	AltaiR	SeqAn	khmer	GTO	HaploCoV	bjorn
**Programming Language**	C	C++	Python, C++	C	Perl	Python, Bash
**Parallel Computing**	✓(Multi-threading)	✓	✗	✓	✗	✗
**Alignment-Free Analysis**	✓	✗	✓	✓✗(some cases)	✗	✗
**Genome Filtering**	Advanced (by length, GC content, patterns)	Basic filtering capabilities	Basic filtering capabilities	Basic filtering capabilities	✗	✗
**NCD Profile**	✓	✗	✗	✓✗(with scripting alterations)	✗	✗
**Complexity Profiles (NC)**	✓	✗	✗	✓	✗	✗
**Nucleotide Frequency Analysis**	✓	✓	✓✗(k-mer frequencies)	✓	✗	✗
**RAW Identification**	✓	✗	✗	✗	✗	✗
**Large-Scale Data Handling**	Optimized for millions of sequences	Large data handling but less optimized	Designed for large datasets but focused on k-mer analysis	✗	Designed for large SARS-CoV-2 datasets	Designed for SARS-CoV-2 datasets
**External Dependencies**	None	Some libraries required	Python environment and dependencies	None (if not considering the external tools)	Perl modules	Python package, Docker
**Modular Toolkit Design**	✓	✓	✗	✓	✗	✗
**FASTA Format Support**	✓	✓	✓	✓	✓	✓
**K-mer Counting and Filtering**	✗	✗	✓	✗	✗	✗
**String Matching Algorithms**	✗	✓	✗	✗	✗	✗
**Haplotype Reconstruction**	✗	✗	✗	✗	✓	✗
**Lineage Assignment**	✗	✗	✗	✗	✗	✓

Table 3. Comparative analysis of AltaiR with SeqAn, khmer, GTO, HaploCoV, and bjorn.

GTO [35], HaploCoV [92], and bjorn [93]. Table 3 shows the results of this comparison focusing on key functionalities, efficiency metrics, and capabilities crucial for handling large-scale genomic data.

AltaiR's implementation in C allows for efficient execution and memory management, making it well-suited for handling large genomic datasets. Its support for multi-threading enables parallel computing, resulting in faster processing times compared to toolkits like khmer, HaploCoV [92, 94], and bjorn [93], which lack built-in parallel computing capabilities. AltaiR's strength lies in its alignment-free approach, which is particularly effective for processing large-scale genomic data. While alignment-free methods offer significant advantages in terms of computational efficiency and the ability to handle large-scale genomic datasets, they may not provide the same level of detailed information as alignment-based methods. Researchers should consider the trade-offs between computational efficiency and the depth of information required for their specific research questions when choosing between these approaches.

One of AltaiR's core strengths is its focus on alignment-free analysis methods, such as Normalized Compression Distance (NCD) profiles and Nucleotide Complexity (NC) profiles. These techniques enable efficient and scalable comparisons of genomic sequences without relying on computationally expensive alignment operations, setting it apart from toolkits like SeqAn, which primarily concentrates on alignment algorithms. The toolkit's use of NCD for constructing similarity profiles enables a detailed exploration of temporal patterns in genomic data, providing insights into the genomic evolution of organisms over time. Additionally, AltaiR includes a feature for generating complexity profiles using NC, which is essential for quantitatively assessing the complexity of genomic sequences over time, providing insights into evolutionary pressures and adaptive responses in different organisms.

AltaiR's ability to filter sequences rigorously was demonstrated in the analysis of the SARS-CoV-2 dataset. It allows users to filter sequences based on length, GC content, and specific patterns, offering more comprehensive and customizable functionality compared to the filtering capabilities provided by SeqAn, khmer, and GTO, while HaploCoV and bjorn do not provide explicit filtering options. Like SeqAn, khmer, and GTO, AltaiR performs nucleotide frequency analysis. However, it extends this functionality with its unique NCD profile and RAW (Relative Absent Words) identifica-

tion capability, enabling the detection of rare and atypical words within genomic sequences, a feature not found in the other toolkits. The frequency profile feature, which tracks changes in nucleotide composition, can be used to study molecular adaptations in various organisms and understand their evolutionary biology. AltaiR is optimized for processing millions of sequences efficiently, specifically targeting alignment-free methods. While SeqAn and GTO also handle large datasets, khmer focuses on k-mer analysis, and HaploCoV [92] and bjorn [93] are designed specifically for SARS-CoV-2 datasets. AltaiR's optimization is tailored towards its specific strengths, following a modular toolkit design that allows for flexibility and adaptability in genomic data analysis workflows, similar to SeqAn and GTO. HaploCoV and bjorn are specialized tools for analyzing SARS-CoV-2 datasets, with HaploCoV focusing on haplotype reconstruction and bjorn providing lineage assignment capabilities. These specialized tools complement the broader functionality offered by AltaiR and the other general-purpose toolkits.

A unique capability of AltaiR is its ability to identify RAWs in genomic and proteomic sequences. By comparing RAWs in pathogens with those in host genomes, AltaiR can uncover distinctive genomic and proteomic elements, with potential applications in pathogen-host interaction studies and the discovery of new genomic and proteomic markers for diagnostic and therapeutic purposes, such as through the combination with aptamers. Aptamers, consisting of short sequences of DNA, RNA, or peptides, serve as molecular tools capable of binding to specific target molecules or families of target molecules. They can modulate the function of specific proteins, influencing signalling pathways or exerting inhibitory or enhancing effects [95]. Notably, aptamers have shown promise in therapeutic applications, as highlighted by Keefe et al. [96]. To discover high-affinity aptamers, contemporary computational methodologies leverage deep learning in conjunction with relevant feature extraction techniques, as Emami et al. [97]. The untapped potential of using mRAWs as pertinent features in aptamer discovery remains uncertain, but their distinctive characteristics suggest the possibility of streamlining subsequent phases of drug discovery and bypassing certain hurdles in the quest for novel therapeutic agents.

The versatility of AltaiR's sub-tools, coupled with its programming efficiency, holds significant importance for seamless integration into intricate pipelines involving multiple tools. This applicability is particularly noteworthy in various domains, such as drug discovery, viral genome reconstruction and analysis, and diversity analysis [98, 99, 100].

Consequentially, AltaiR stands out as a comprehensive and efficient toolkit for alignment-free analysis of large-scale genomic datasets. Its unique features, optimization for handling millions of sequences, and broad applicability position it as a powerful resource for a wide range of genomic and proteomic research endeavours, complementing the functionalities offered by other established toolkits and specialized tools.

Conclusions

In this study, we introduced AltaiR, a versatile and robust toolkit designed for the advanced analysis of large-scale genomic datasets. AltaiR's alignment-free methodology efficiently manages extensive data, exemplified by its role in analyzing a large number of SARS-CoV-2 sequences. The toolkit's capabilities, including Normalized Compression Distance (NCD) profiling, Normalized Compression (NC) analysis, temporal nucleotide composition variation, and the identification of Relative Absent Words (RAWs), showcase its adaptability to diverse genomic data types and research requirements.

AltaiR's innovation lies in the integration of well-established filtering methods, such as sequence length, GC content, and pattern matching, with novel and enhanced features into a comprehensive framework designed for efficient, large-scale genomic data analysis. The toolkit introduces new methods, including the Frequency method for detailed nucleotide or amino acid occurrence analysis, and extends the capabilities of the Filter method by supporting multiple simultaneous pattern searches, absent pattern searches, and filtering by GC content, sequence length, or completeness.

Moreover, AltaiR's NCD method introduces a novel approach that uses a conjunction of reference and target files while freezing the models of one and saving computation time for other sequences. This methodology allows for substantial time savings in NCD calculations. The NC analysis provides a quantitative assessment of the complexity of genomic sequences over time, offering insights into evolutionary pressures and adaptive responses in different organisms.

A unique capability of AltaiR is its ability to identify RAWs in genomic and proteomic sequences. The RAWs method in AltaiR supports genomic and protein sequences. By comparing RAWs in pathogens with those in host genomes, AltaiR can uncover distinctive genomic and proteomic elements, with potential applications in pathogen-host interaction studies and the discovery of new genomic and proteomic markers for diagnostic and therapeutic purposes.

The combination of these tools, along with the optimization for analyzing millions of sequences, ensures high portability and ease of installation while providing the necessary capabilities for rigorous analyses. This versatility enables a streamlined workflow, allowing researchers to process raw, unfiltered data and obtain meaningful insights without the need for multiple, disconnected tools.

AltaiR's application in studying SARS-CoV-2 genomes has provided possible insights into the virus's evolution and adaptations. However, the potential of AltaiR extends beyond virology, making it a crucial tool in broader genomic and proteomic research, such as in the analysis of resistant bacteria, unravelling complex temporal patterns, and facilitating studies on the evolution and diversity of various organisms.

As genomic research advances, driven by technological advancements and the increasing complexity of biological data, AltaiR's scalability, and efficiency position it as a powerful resource for a wide range of genomic and proteomic research.

Availability of source code and requirements

- Project name: AltaiR
- Project home page: https://github.com/cobilab/altair
- Operating system(s): Linux
- Programming language: C; Python; Bash
- Other requirements: Conda v4.3.27.
- License: e.g. GPLv3 License.
- RRID: SCR_024752
- Biotools: altair

Declarations

List of abbreviations

DNA - Deoxyribonucleic acid;
HSV-1 - Herpes Simplex Virus 1;
HSV-2 - Herpes Simplex Virus 2;
ID - Identifier;
MAW - minimal absent word;
mRAW - minimal Relative Absent Word;
PmRAW - Persistent minimal Relative Absent Word;
NC - Normalized Compression;
NCD - Normalized Compression Distance;
RAW - Relative Absent Word;
RNA - Ribonucleic acid;

T2T - Telomere-2-Telomere;
VZV - Varicella-Zoster Virus;

Competing Interests

The authors declare no competing interests.

Funding

This work was partially funded by National Funds through the FCT - Foundation for Science and Technology, in the context of the project UIDB/00127/2020. J.M.S. has received funding from the EC under grant agreement 101081813, Genomic Data Infrastructure. D.P. is funded by national funds through FCT – Fundação para a Ciência e a Tecnologia, I.P., under the Scientific Employment Stimulus - Institutional Call - reference CEECINST/00026/2018.

Acknowledgements

The authors wish to thank the Finnish Computing Competence Infrastructure (FCCI) for supporting this project with computational and data storage resources.

Author's Contributions

D.P. and A.P. designed the experiment. D.P. and J.M.S. coded the tools. J.M.S. executed the data analysis. J.M.S. and D.P. discussed the results. J.M.S. and D.P. wrote the manuscript. All authors have revised the manuscript.

References

1. Khare S, Gurry C, Freitas L, Schultz MB, Bach G, Diallo A, et al. GISAID's Role in Pandemic Response. China CDC Weekly 2021;3(49):1049.
2. Hatcher EL, Zhdanov SA, Bao Y, Blinkova O, Nawrocki EP, Ostapchuck Y, et al. Virus Variation Resource–improved response to emergent viral outbreaks. Nucleic acids research 2017;45(D1):D482–D490.
3. Cochrane G, Karsch-Mizrachi I, Takagi T, Sequence Database Collaboration IN. The international nucleotide sequence database collaboration. Nucleic acids research 2016;44(D1):D48–D50.
4. Sayers EW, Cavanaugh M, Clark K, Pruitt KD, Schoch CL, Sherry ST, et al. GenBank. Nucleic acids research 2021;49(D1):D92–D96.
5. Harrison PW, Ahamed A, Aslam R, Alako BT, Burgin J, Buso N, et al. The european nucleotide archive in 2020. Nucleic acids research 2021;49(D1):D82–D85.
6. Okido T, Kodama Y, Mashima J, Kosuge T, Fujisawa T, Ogasawara O. DNA Data Bank of Japan (DDBJ) update report 2021. Nucleic acids research 2022;50(D1):D102–D105.
7. de Vries JJ, Brown JR, Couto N, Beer M, Le Mercier P, Sidorov I, et al. Recommendations for the introduction of metagenomic next-generation sequencing in clinical virology, part II: bioinformatic analysis and reporting. Journal of Clinical Virology 2021;138:104812.
8. López-Labrador FX, Brown JR, Fischer N, Harvala H, Van Boheemen S, Cinek O, et al. Recommendations for the introduction of metagenomic high-throughput sequencing in clinical virology, part I: Wet lab procedure. Journal of Clinical Virology 2021;134:104691.
9. Simon-Loriere E, Holmes EC. Why do RNA viruses recombine? Nature Reviews Microbiology 2011;9(8):617–626.
10. Li X, Giorgi EE, Marichannegowda MH, Foley B, Xiao C, Kong XP, et al. Emergence of SARS-CoV-2 through recombination and strong purifying selection. Science advances 2020;6(27):eabb9153.
11. Palmenberg AC, Spiro D, Kuzmickas R, Wang S, Djikeng A, Rathe JA, et al. Sequencing and analyses of all known human rhinovirus genomes reveal structure and evolution. Science 2009;324(5923):55–59.
12. Plummer M, de Martel C, Vignat J, Ferlay J, Bray F, Franceschi S. Global burden of cancers attributable to infections in 2012: a synthetic analysis. The Lancet Global Health 2016;4(9):e609–e616.
13. Farrell PJ. Epstein–Barr virus and cancer. Annual Review of Pathology: Mechanisms of Disease 2019;14:29–53.
14. Smatti MK, Cyprian FS, Nasrallah GK, Al Thani AA, Almishal RO, Yassine HM. Viruses and autoimmunity: a review on the potential interaction and molecular mechanisms. Viruses 2019;11(8):762.
15. Pyöriä L, Pratas D, Toppinen M, Hedman K, Sajantila A, Perdomo MF. Unmasking the tissue-resident eukaryotic DNA virome in humans. Nucleic Acids Research 2023;51(7):3223–3239.
16. Toppinen M, Sajantila A, Pratas D, Hedman K, Perdomo MF. The Human Bone Marrow Is Host to the DNAs of Several Viruses. Frontiers in cellular and infection microbiology 2021;11:329.
17. Toppinen M, Pratas D, Väisänen E, Söderlund-Venermo M, Hedman K, Perdomo MF, et al. The landscape of persistent human DNA viruses in femoral bone. Forensic Science International: Genetics 2020;48:102353.
18. Land M, Hauser L, Jun SR, Nookaew I, Leuze MR, Ahn TH, et al. Insights from 20 years of bacterial genome sequencing. Functional & integrative genomics 2015;15(2):141–161.
19. Nurk S, Koren S, Rhie A, Rautiainen M, Bzikadze AV, Mikheenko A, et al. The complete sequence of a human genome. Science 2022;376(6588):44–53.
20. Qi W, Lim YW, Patrignani A, Schläpfer P, Bratus-Neuenschwander A, Grüter S, et al. The haplotype-resolved chromosome pairs of a heterozygous diploid African cassava cultivar reveal novel pan-genome and allele-specific transcriptome features. GigaScience 2022;11.
21. Mc Cartney AM, Shafin K, Alonge M, Bzikadze AV, Formenti G, Fungtammasan A, et al. Chasing perfection: validation and polishing strategies for telomere-to-telomere genome assemblies. Nature Methods 2022;p. 1–9.
22. Alkan C, Carbone L, Dennis M, Ernst J, Evrony G, Girirajan S, et al. Implications of the first complete human genome assembly. Genome Research 2022;32(4):595.
23. Vinga S, Almeida J. Alignment-free sequence comparison—a review. Bioinformatics 2003;19(4):513–523.
24. Reinert G, Chew D, Sun F, Waterman MS. Alignment-free sequence comparison (I): statistics and power. Journal of Computational Biology 2009;16(12):1615–1634.
25. Wan L, Reinert G, Sun F, Waterman MS. Alignment-free sequence comparison (II): theoretical power of comparison statistics. Journal of Computational Biology 2010;17(11):1467–1490.
26. Zielezinski A, Vinga S, Almeida J, Karlowski WM. Alignment-free sequence comparison: benefits, applications, and tools. Genome biology 2017;18(1):1–17.
27. Zielezinski A, Girgis HZ, Bernard G, Leimeister CA, Tang K, Dencker T, et al. Benchmarking of alignment-free sequence comparison methods. Genome biology 2019;20(1):1–18.
28. Metcalf JL, Xu ZZ, Bouslimani A, Dorrestein P, Carter DO, Knight R. Microbiome tools for forensic science. Trends in biotechnology 2017;35(9):814–823.
29. Chen Z, Zhao P, Li F, Marquez-Lago TT, Leier A, Revote J, et al. iLearn: an integrated platform and meta-learner for feature engineering, machine-learning analysis and modeling

of DNA, RNA and protein sequence data. Briefings in bioinformatics 2020;21(3):1047–1057.

30. Yang J, Chen X, McDermaid A, Ma Q. DMINDA 2.0: integrated and systematic views of regulatory DNA motif identification and analyses. Bioinformatics 2017;33(16):2586–2588.
31. Min JL, Hemani G, Davey Smith G, Relton C, Suderman M. Meffil: efficient normalization and analysis of very large DNA methylation datasets. Bioinformatics 2018;34(23):3983–3989.
32. Döring A, Weese D, Rausch T, Reinert K. SeqAn an efficient, generic C++ library for sequence analysis. BMC bioinformatics 2008;9(1):1–9.
33. Crusoe MR, Alameldin HF, Awad S, Boucher E, Caldwell A, Cartwright R, et al. The khmer software package: enabling efficient nucleotide sequence analysis. F1000Research 2015;4.
34. Standage D, Aliyari A, Cohen LJ, Crusoe MR, Head T, Irber L, et al. khmer release v2.1: software for biological sequence analysis. Journal of Open Source Software 2017;2(15):272. https://doi.org/10.21105/joss.00272.
35. Almeida JR, Pinho AJ, Oliveira JL, Fajarda O, Pratas D. GTO: a toolkit to unify pipelines in genomic and proteomic research. SoftwareX 2020;12:100535.
36. Drezen E, Rizk G, Chikhi R, Deltel C, Lemaitre C, Peterlongo P, et al. GATB: genome assembly & analysis tool box. Bioinformatics 2014;30(20):2959–2961.
37. Lee J, Lee AJ, Lee JK, Park J, Kwon Y, Park S, et al. Mutalisk: a web-based somatic MUTation AnaLyIS toolKit for genomic, transcriptional and epigenomic signatures. Nucleic acids research 2018;46(W1):W102–W108.
38. Sims D, Ilott NE, Sansom SN, Sudbery IM, Johnson JS, Fawcett KA, et al. CGAT: computational genomics analysis toolkit. Bioinformatics 2014;30(9):1290–1291.
39. Hiltemann S, Mei H, de Hollander M, Palli I, van der Spek P, Jenster G, et al. CGtag: complete genomics toolkit and annotation in a cloud-based Galaxy. GigaScience 2014;3(1):2047–217X.
40. de Koning W, Miladi M, Hiltemann S, Heikema A, Hays JP, Flemming S, et al. NanoGalaxy: Nanopore long-read sequencing data analysis in Galaxy. GigaScience 2020;9(10):giaa105.
41. Silva JM, Qi W, Pinho AJ, Pratas D. AlcoR: alignment-free simulation, mapping, and visualization of low-complexity regions in biological data. GigaScience 2023;.
42. Loman NJ, Quinlan AR. Poretools: a toolkit for analyzing nanopore sequence data. Bioinformatics 2014;30(23):3399–3401.
43. Knight R, Maxwell P, Birmingham A, Carnes J, Caporaso JG, Easton BC, et al. PyCogent: a toolkit for making sense from sequence. Genome biology 2007;8(8):1–16.
44. Shen W, Le S, Li Y, Hu F. SeqKit: a cross-platform and ultrafast toolkit for FASTA/Q file manipulation. PloS one 2016;11(10):e0163962.
45. Alam KK, Chang JL, Burke DH. FASTAptamer: a bioinformatic toolkit for high-throughput sequence analysis of combinatorial selections. Molecular Therapy-Nucleic Acids 2015;4:e230.
46. Ott M, Edunov S, Baevski A, Fan A, Gross S, Ng N, et al. fairseq: A Fast, Extensible Toolkit for Sequence Modeling. In: Proceedings of the 2019 Conference of the North American Chapter of the Association for Computational Linguistics (Demonstrations) Minneapolis, Minnesota: Association for Computational Linguistics; 2019. p. 48–53. https://aclanthology.org/N19-4009.
47. Chen C, Chen H, Zhang Y, Thomas HR, Frank MH, He Y, et al. TBtools: an integrative toolkit developed for interactive analyses of big biological data. Molecular plant 2020;13(8):1194–1202.
48. Gabler F, Nam SZ, Till S, Mirdita M, Steinegger M, Söding J, et al. Protein sequence analysis using the MPI bioinformatics toolkit. Current Protocols in Bioinformatics 2020;72(1):e108.
49. Arkin AP, Cottingham RW, Henry CS, Harris NL, Stevens RL, Maslov S, et al. KBase: the United States department of energy systems biology knowledgebase. Nature biotechnology 2018;36(7):566–569.
50. Kryukov K, Ueda MT, Nakagawa S, Imanishi T. Nucleotide Archival Format (NAF) enables efficient lossless reference-free compression of DNA sequences. Bioinformatics 2019;35(19):3826–3828.
51. Deorowicz S, Danek A, Li H. AGC: compact representation of assembled genomes with fast queries and updates. Bioinformatics 2023;39(3):btad097.
52. Grabowski S, Kowalski TM. MBGC: Multiple Bacteria Genome Compressor. GigaScience 2022;11.
53. Pinho AJ, Pratas D. MFCompress: a compression tool for FASTA and multi-FASTA data. Bioinformatics 2014;30(1):117–118.
54. Hosseini M, Pratas D, Pinho AJ. Cryfa: a secure encryption tool for genomic data. Bioinformatics 2019;35(1):146–148.
55. Lu J, Salzberg SL. Removing contaminants from databases of draft genomes. PLoS computational biology 2018;14(6):e1006277.
56. Sajantila A, Editors' Pick: Contamination has always been the issue! BioMed Central; 2014.
57. Li M, Chen X, Li X, Ma B, Vitányi PM. The similarity metric. IEEE transactions on Information Theory 2004;50(12):3250–3264.
58. Li M, Vitányi P, et al. An introduction to Kolmogorov complexity and its applications, vol. 3. Springer; 2008.
59. Kolmogorov AN. Three approaches to the quantitative definition of information. Problems of information transmission 1965;1(1):1–7.
60. Pratas D, Silva RM, Pinho AJ. Comparison of compression-based measures with application to the evolution of primate genomes. Entropy 2018;20(6):393.
61. Cilibrasi RL, Vitányi PM. Fast Phylogeny of SARS-CoV-2 by Compression. Entropy 2022;24(4):439.
62. Silva M, Pratas D, Pinho AJ. AC2: An Efficient Protein Sequence Compression Tool Using Artificial Neural Networks and Cache-Hash Models. Entropy 2021;23(5):530.
63. Cebrián M, Alfonseca M, Ortega A. Common pitfalls using the normalized compression distance: What to watch out for in a compressor. Communications in Information & Systems 2005;5(4):367–384.
64. Hosseini M, Pratas D, Pinho AJ. On the role of inverted repeats in DNA sequence similarity. In: International Conference on Practical Applications of Computational Biology & Bioinformatics Springer; 2017. p. 228–236.
65. Pratas D, Hosseini M, Pinho AJ. Substitutional tolerant Markov models for relative compression of DNA sequences. In: International Conference on Practical Applications of Computational Biology & Bioinformatics Springer; 2017. p. 265–272.
66. Silva M, Pratas D, Pinho AJ. Efficient DNA sequence compression with neural networks. GigaScience 2020;9(11):giaa119.
67. Pratas D, Pinho AJ. On the approximation of the Kolmogorov complexity for DNA sequences. In: Iberian Conference on Pattern Recognition and Image Analysis Springer; 2017. p. 259–266.
68. Silva RM, Pratas D, Castro L, Pinho AJ, Ferreira PJ. Three minimal sequences found in Ebola virus genomes and absent from human DNA. Bioinformatics 2015;31(15):2421–2425.
69. Pratas D, Silva JM. Persistent minimal sequences of SARS-CoV-2. Bioinformatics 2020 07;36(21):5129–5132.
70. Pratas D. Compression and analysis of genomic data. PhD thesis, Universidade de Aveiro (Portugal); 2016.
71. Béal MP, Mignosi F, Restivo A. Minimal forbidden words and symbolic dynamics. In: Annual Symposium on Theoretical Aspects of Computer Science Springer; 1996. p. 555–566.
72. Crochemore M, Mignosi F, Restivo A. Automata and Forbidden Words. Inf Process Lett 1998 Aug;67(3):111–117.

73. Pinho AJ, Ferreira PJ, Garcia SP, Rodrigues JM. On finding minimal absent words. BMC bioinformatics 2009;10(1):1–11.
74. Kosche M, Koß T, Manea F, Siemer S. Absent subsequences in words. In: International Conference on Reachability Problems Springer; 2021. p. 115–131.
75. Bernardini G, Marchetti-Spaccamela A, Pissis SP, Stougie L, Sweering M. Constructing strings avoiding forbidden substrings. In: 32nd Annual Symposium on Combinatorial Pattern Matching (CPM 2021), vol. 191; 2021. p. 1–9.
76. Koulouras G, Frith MC. Significant non-existence of sequences in genomes and proteomes. Nucleic acids research 2021;49(6):3139–3155.
77. Akon M, Akon M, Kabir M, Rahman MS, Rahman MS. ADACT: a tool for analysing (dis) similarity among nucleotide and protein sequences using minimal and relative absent words. Bioinformatics 2021;37(10):1468–1470.
78. NCBI, NCBI Virus. NCBI; 2023. https://www.ncbi.nlm.nih.gov/labs/virus/vssi/#/virus?SeqType_s=Nucleotide&VirusLineage_ss=Wuhan%20seafood%20market%20pneumonia%20virus,%20taxid:2697049&Completeness_s=complete&HostLineage_ss=Homo%20sapiens%20(human),%20taxid:9606&CreateDate_dt=2000-01-01T00:00:00.00Z%20TO%202022-09-28T23:59:59.00Z, [Online; accessed 27-November-2023].
79. Singh D, Yi SV. On the origin and evolution of SARS-CoV-2. Experimental & Molecular Medicine 2021;53(4):537–547.
80. Drake JW, Holland JJ. Mutation rates among RNA viruses. Proceedings of the National Academy of Sciences 1999;96(24):13910–13913.
81. Sanjuán R, Nebot MR, Chirico N, Mansky LM, Belshaw R. Viral mutation rates. Journal of virology 2010;84(19):9733–9748.
82. Grubaugh ND, Hanage WP, Rasmussen AL. Making sense of mutation: what D614G means for the COVID-19 pandemic remains unclear. Cell 2020;182(4):794–795.
83. Harvey WT, Carabelli AM, Jackson B, Gupta RK, Thomson EC, Harrison EM, et al. SARS-CoV-2 variants, spike mutations and immune escape. Nature Reviews Microbiology 2021;19(7):409–424.
84. Korber B, Fischer WM, Gnanakaran S, Yoon H, Theiler J, Abfalterer W, et al. Tracking changes in SARS-CoV-2 spike: evidence that D614G increases infectivity of the COVID-19 virus. Cell 2020;182(4):812–827.
85. Plante JA, Liu Y, Liu J, Xia H, Johnson BA, Lokugamage KG, et al. Spike mutation D614G alters SARS-CoV-2 fitness. Nature 2021;592(7852):116–121.
86. Walls AC, Park YJ, Tortorici MA, Wall A, McGuire AT, Veesler D. Structure, function, and antigenicity of the SARS-CoV-2 spike glycoprotein. Cell 2020;181(2):281–292.
87. NCBI, Homo sapiens genome assembly T2T-CHM13v2.0. NCBI; 2023. https://www.ncbi.nlm.nih.gov/datasets/genome/GCF_009914755.1/, [Online; accessed 27-November-2023].
88. NCBI, Human Genome Resources at NCBI. NCBI; 2023. https://www.ncbi.nlm.nih.gov/genome/guide/human/, [Online; accessed 27-November-2023].
89. Wang Y, Mao JM, Wang GD, Luo ZP, Yang L, Yao Q, et al. Human SARS-CoV-2 has evolved to reduce CG dinucleotide in its open reading frames. Scientific Reports 2020;10(1):12331.
90. Takata MA, Gonçalves-Carneiro D, Zang TM, Soll SJ, York A, Blanco-Melo D, et al. CG dinucleotide suppression enables antiviral defence targeting non-self RNA. Nature 2017;550(7674):124–127.
91. Afrasiabi A, Alinejad-Rokny H, Khosh A, Rahnama M, Lovell N, Xu Z, et al. The low abundance of CpG in the SARS-CoV-2 genome is not an evolutionarily signature of ZAP. Scientific reports 2022;12(1):2420.
92. Chiara M, Horner DS, Ferrandi E, Gissi C, Pesole G. HaploCoV: unsupervised classification and rapid detection of novel emerging variants of SARS-CoV-2. Communications Biology 2023;6(1):443.
93. Lab A, Bjorn: Bioinformatics Pipeline for Analyzing SARS-CoV-2 Genomes;. Accessed: 2024-06-13. https://github.com/andersen-lab/bjorn.
94. Chiara M, HaploCoV: A Tool for Haplotype Analysis in SARS-CoV-2 Genomes;. Accessed: 2024-06-13. https://github.com/matteo14c/HaploCoV.
95. Crivianu-Gaita V, Thompson M. Aptamers, antibody scFv, and antibody Fab'fragments: An overview and comparison of three of the most versatile biosensor biorecognition elements. Biosensors and Bioelectronics 2016;85:32–45.
96. Keefe AD, Pai S, Ellington A. Aptamers as therapeutics. Nature reviews Drug discovery 2010;9(7):537–550.
97. Emami N, Ferdousi R. AptaNet as a deep learning approach for aptamer–protein interaction prediction. Scientific reports 2021;11(1):6074.
98. Herzig V, Cristofori-Armstrong B, Israel MR, Nixon SA, Vetter I, King GF. Animal toxins—Nature's evolutionary-refined toolkit for basic research and drug discovery. Biochemical pharmacology 2020;181:114096.
99. Pratas D, Toppinen M, Pyöriä L, Hedman K, Sajantila A, Perdomo MF. A hybrid pipeline for reconstruction and analysis of viral genomes at multi-organ level. GigaScience 2020;9(8):giaa086.
100. Adolfo LM, Rao X, Dixon RA. Identification of Pueraria spp. through DNA barcoding and comparative transcriptomics. BMC Plant Biology 2022;22(1):1–18.

Manuscript

Click here to access/download;Manuscript;Manuscript_review.pdf

GigaScience, 2023, 1–0

doi: xx.xxxx/xxxx
Manuscript in Preparation
Technical Note

TECHNICAL NOTE

AltaiR: a C toolkit for alignment-free and temporal analysis of multi-FASTA data

Jorge M. Silva[1,2,*], Armando J. Pinho[1,2] and Diogo Pratas[1,2,3,*]

[1]IEETA/LASI, Institute of Electronics and Informatics Engineering of Aveiro, University of Aveiro, Portugal and [2]DETI, Department of Electronics, Telecommunications and Informatics, University of Aveiro, Portugal and [3]DoV, Department of Virology, University of Helsinki, Finland

[*]Correspondence address. Jorge M. Silva and Diogo Pratas. E-mail: jorge.miguel.ferreira.silva@ua.pt https://orcid.org/0000-0002-6331-6091 and pratas@ua.pt http://orcid.org/0000-0003-1176-552X

Abstract

Background: The vast number of viral genome sequences generated during the latest pandemic has presented new challenges for computational analysis. Analyzing millions of viral genomes in multi-FASTA format is computationally demanding, especially when using alignment-based methods. Most existing methods are not designed to handle such large datasets, often requiring the analysis to be divided into smaller parts to obtain results using available computational resources.
Findings: We introduce AltaiR, a toolkit for analyzing multiple sequences in multi-FASTA format using exclusively alignment-free methodologies. AltaiR enables the identification of singularity and similarity patterns within sequences and computes static and temporal dynamics without restrictions on the number or size of input sequences. It automatically filters low-quality, biased, or deviant data. We demonstrate AltaiR's capabilities by analyzing more than 1.5 million full SARS-CoV-2 sequences, revealing interesting observations regarding viral genome characteristics over time, such as shifts in nucleotide composition, decreases in average sequence complexity, and the evolution of the smallest sequences not found in the human host.
Conclusions: AltaiR can identify temporal characteristics and trends in large numbers of sequences, making it ideal for scenarios involving endemic or epidemic outbreaks with vast amounts of available sequence data. Implemented in C with multi-threading and methodological optimizations, AltaiR is computationally efficient, flexible, and dependency-free. It accepts any sequence in FASTA format, including amino acid sequences. The complete toolkit is freely available at https://github.com/cobilab/altair.

Key words: Alignment-free toolkit; Relative Absent Words; Data compression; Temporal patterns; Viral genomes; multi-FASTA;

Introduction

The recent availability of millions of SARS-CoV-2 complete genomes that emerged from the COVID-19 pandemic has remarkably changed the scientific workflow. It starts with the parallelization of the genome's sequencing and assembly through many scientific and industrial entities, followed by the centralized upload of each SARS-CoV-2 assembled sequence in FASTA format and the respective metadata, while respecting a light data curation, mainly in the following database repositories: GISAID [1], NCBI [2], and INSDC [3], that include GeneBank [4], ENA [5], and DDBJ [6].

Although the incredible growing pace at which these complete genomes emerged and have been made publicly available, their downstream analysis faces new challenges mainly related to the vast number of genomes, characteristics and format of the data, and multiplexed sequencing-reconstruction.

Specifically, one challenge emerged due to the availability of millions of genomes split by the respective headers with basic information in a single file. This file format is known as Multi-FASTA, but it is generically called FASTA. There are alternative and more efficient file format representations, such as those representing only differences according to a reference. Still, the majority of the downstream analysis tools require the FASTA format. Moreover, storing only the differences would require multiple references to perform additional analyses at different taxonomic levels. The alternative way of using a single reference sequence becomes inefficient,

Compiled on: June 18, 2024.
Draft manuscript prepared by the author.

if there is high dissimilarity.

Furthermore, for processing up to a hundred thousand genomes, only a minority of the tools are prepared, while the availability of these tools is very scarce to process millions. The main reason is these programs' use of temporary files to decrease the computational memory and related computational time. However, creating temporary files times millions of entries is not efficiently affordable.

Another challenge is the existence of outlier or recombinant genomes in the set. The outlier entries are unwanted sequences uploaded by entities that show profound differences in sequencing and assembly methodologies. Examples of these features are the presence/absence of targeted capture, the assembly methodology using appropriate/inappropriate references, proper/improper exclusion of contaminants, accurate/inaccurate variant call, accurate/inaccurate base masking, or substantial differences in SNPs caused by postmortem degradation. Although several directives have been provided [7, 8], these genomes still exist and are continuously being uploaded. Recombinant genomes usually show a higher degree of variants or chimeric formations after cell co-infection [9]. Due to these temporary increases in the accumulation of genomic variations, recombinant genomes can be associated with the emergence of outbreak strains, as exemplified by coronaviruses and rhinoviruses [10, 11]. For downstream analysis, the objective is to discard outliers while maintaining the recombinant genomes. However, we currently lack fast genome filtering tools that assert a particular trait distribution to minimize outliers in the data while maintaining the average genome data of these recombinant genomes.

Beyond the SARS-CoV-2 sequences, the upload of large quantities of viral genomes in FASTA format is also substantially increasing. Because viral genomes are sometimes directly associated with hosting health conditions, namely in cancer or autoimmune diseases, the number of these reconstructed genomes is expected to increase dramatically for clinical and forensic purposes [12, 13, 14]. Some examples are the steadily increasing availability of Parvoviruses, Polyomaviruses, Herpesviruses, and Papillomaviruses [15, 16, 17]. Moreover, this increase is also in other types of organisms, such as organisms with larger genome sequences, namely fungi and bacteria [18], especially now with the higher rates of antibiotic resistance and the availability of complete genome sequences [19, 20] associated with the development of the Telomere-2-Telomere (T2T) technology [21, 22].

The current disposal of the massive number of genomes from a single species also provides the opportunity to study a species sequence over time, namely the temporal patterns and characteristics of the genomes or proteomes in a temporal dimension. Accordingly, the following questions emerge:

- How does the nucleotide composition of the genomes change over time?
- How does the sequence entropy of a species change in time?
- How does the similarity of parasitic sequence species vary according to the first known sequence?
- What are the shortest sub-sequences of a parasitic species not in a host?
- How do these shortest sequences change in time?

These complex questions can be answered with this type of data in synergy with the alignment-free method that is provided in this manuscript.

Although alignment methods offer an intuitive and enhanced local resolution that prevails in comparative analysis of specific features in a low number of sequences, large-scale sequence quantities require unfeasible computational resources under a desirable accuracy, limiting their applicability in multiple temporal analyses. On the other hand, the substantial increase in the development and availability of alignment-free methods [23, 24, 25, 26, 27] have provided clear advantages using feasible computational resources.

In the literature, there are multiple toolkits for specific sequence transformation and analyses applications, namely microbiome tools for forensic science [28], for machine-learning analysis and modelling of genomic and proteomic sequence data [29], for visualization of regulatory DNA motif identification and analyses [30], efficient analysis of DNA methylation [31], among many others. On the other hand, there are toolkits or platforms with a much broader application, namely SeqAn [32], khmer [33, 34], GTO [35], GATB [36], Mutalisk [37], CGAT [38], CGtag [39], nanoGalaxy [40], AlcoR [41], Poretools [42], Pycogent [43], SeqKit [44], FASTAptamer [45], fairseq [46], SeqKit [44], TBtools [47], MPI bioinformatics toolkit [48], KBase [49], among many others. Some of these toolkits or platforms are interactive through web browsers offering friendly environments, while others are prone to fast and efficient computation through the command line.

Benchmarking genomic toolkits by direct comparison can be misleading due to their distinctive design philosophies and unique feature sets. Each toolkit is crafted with specific goals in mind, which may not align perfectly with those of another. Hence, simple side-by-side comparisons might not only be uninformative but could obscure the individual strengths of each tool. Instead, a more nuanced approach involves evaluating a toolkit based on its specific features and objectives. Important aspects to consider include the toolkit's innovative features, the research questions it facilitates, its efficiency and ease of use, and any software dependencies it might have. These criteria form the core of high-level benchmarking, focusing on what each toolkit brings to the scientific community rather than how it matches up against others.

On a deeper level, benchmarking should employ both synthetic data, which provides a controlled environment to assess tool performance predictably, and natural data, which offers real-world challenges. Synthetic data testing is essential and should be a standard preliminary test, whereas natural data testing, although occasionally challenging due to the variability of biological data, is crucial for understanding how the tool performs under realistic conditions. Additionally, the reproducibility and, when feasible, the repeatability of results are paramount to confirm the reliability and effectiveness of each tool. This rigorous approach ensures that the tools not only meet theoretical expectations but also hold up under practical application.

In this article, we present AltaiR, a toolkit with alignment-free methods for the temporal analysis of multi-FASTA data, specifically large-scale numbers of genomes or proteomes, including millions (or billions) of sequences in a single FASTA file. The AltaiR toolkit offers an efficient and convenient approach for pathogen-host analyses in endemic or pandemic scenarios (but not limited to). Importantly, the AltaiR method includes a flexible tool to filter sequences undesired from the dataset through parameterized characteristics. AltaiR contains both reference-free and reference-based tools. The reference-free tools are for analysing sequence entropy and nucleotide frequency changes over time. The reference-based tools are divided into two main wings, similarity and singularity. For both, highly efficient implementations are provided. The AltaiR is developed in C language and is provided as open-source software. In the next section, we enumerate the features and characteristics of the AltaiR toolkit, including the description and formalization of the methods. Then, we benchmark each tool from the toolkit using synthetic data while ensuring the full repeatability of the experiment. Afterwards, we present results using natural data, namely an application for a vast SARS-CoV-2 set of genomes. Finally, we discuss the results obtained and draw some conclusions.

Methods

This section describes the methods and their respective implementation into computer tools that constitute the AltaiR toolkit. The details of the parameters and how to reproduce the methods are

available in Supplementary Sections 2 and 3. We recommend using compressed data, namely through the compression of the files using specialized tools. For fast access, NAF [50], AGC [51], and, for bacteria, MBGC [52] can be used. For a higher compression ratio but slower access, we recommend MFCompress [53]. When using sensitive data, Cryfa [54] can be used to compact and encrypt the data.

Figure 1 describes the workflow of AltaiR by dividing the data according to three types: the parasite reference sequences, the parasite sequences (after filtering the outliers or unwanted data), and the host omics sequences. The workflow contains seven main methods.

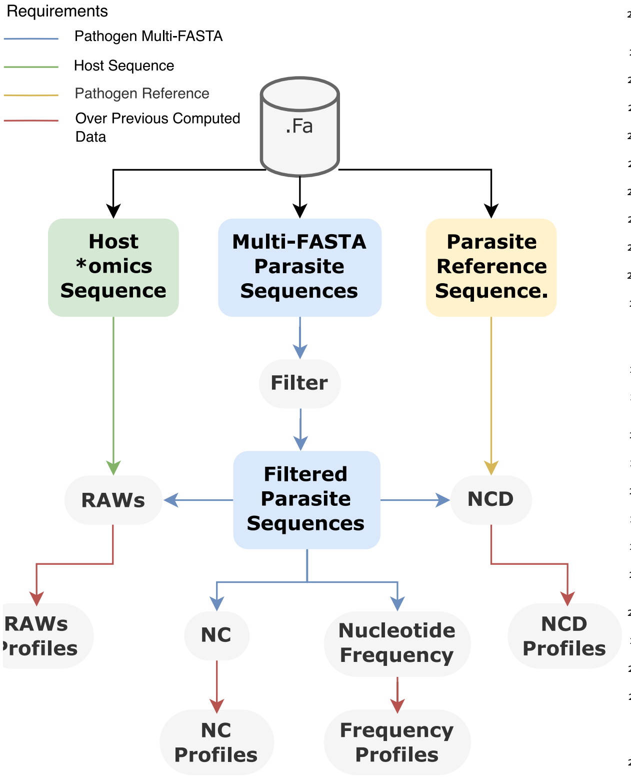

Figure 1. Workflow of the AltaiR toolkit describing the principal seven phases along with the three input types of sequences in FASTA format.

The first method is filtering the sequences by characteristics, essentially removing sequences that contain different traits from what is expected or the average. The second method is the Normalized Compression Distance (NCD) for measuring the similarity between two sequences. The NCD extension is provided in the third method through the use of the NCD profiles. These are generated by the similarity comparison of the existent sequences according to a reference. The fourth method is the Normalized Compression (NC) profiles that depict the compression variability of the sequences. The fifth method is the frequency profiles, allowing to visualize changes in the nucleotide composition over time. The sixth method is the RAWs (Relative Absent Words) mapping that identifies the shortest sequences in the parasite but absent from the host genome/transcriptome. Finally, the seventh method provides profiles regarding the RAWs according to time.

The further subsections provide details on the methods, including their mathematical formalizations and characteristics.

Filter sequences by characteristics

The public databases of multiple genome and proteome sequences from a specific organism usually contain sequences not within the average group. The most frequent reasons are the inclusion of contaminant sequences [55, 56] and differences in the sequencing or assembly procedures. Removing some of these sequences from the dataset is important for downstream analyses. However, manual inspection is slow and can introduce errors. Therefore, automatically filtering these sequences by characteristics is important and can provide unbiased selections while maintaining visible static and uniform characteristics. AltaiR incorporates established guidelines and best practices [7, 8] to address potential contamination and quality issues through automated filtering. This process ensures only high-quality, contiguous sequences are included in the analysis, minimizing bias from incomplete or contaminated data.

Accordingly, this sub-method filters a multi-FASTA file, specifically FASTA reads, by input characteristics. These characteristics can be the alphabet composition, sequence completeness or length, GC quantity, multiple string header patterns, or absence of patterns (anti-patterns). Completeness refers to the availability of the full, uninterrupted sequence of an organism's genome, rather than just partial or fragmented sequences. The computation can be applied to different sequences; for example, a specific filtering can be applied to SARS-CoV-2 while another for B19V. Moreover, the filtering characteristics can be intersected through a single run.

Consider a source that has generated n sequences from a finite alphabet Ξ with size $|\Xi|$. The nature of the source is unknown but in each sequence x^i, where $x^0, ..., x^n$, the internal symbols are known.

The filtering aims to evaluate specific characteristics that occur in each x^i, and those which respect the conditions are used for further downstream analyses. Moreover, each x^i contains a header alphanumeric sequence, y^i, that generically identifies x_i. Specifically, the y^i is the metadata of x^i and usually contains the name of the organism, the date, and a unique ID, among others.

The following definitions provide information on the filtering for each main characteristic or feature, including the sequence length, alphabet composition, minimum and maximum GC percentage, header patterns and anti-patterns.

Sequence length

In multiple cases, sequences labelled as complete are nearly complete, or the length is shorter than a certain length. This can occur, for instance, due to the sequencing depth being very low or some scaffolds could not be linked in the assembly process. AltaiR can filter sequences by the minimal and maximal length to minimise these issues. Therefore, for any string x^i with length $|x^i|$ the set of final sequences, Σ, is composed by

$$x^i \in \Sigma \iff \mathcal{N}_{\max} \geq \mathcal{N}(x^i) \geq \mathcal{N}_{\min}, \tag{1}$$

where $\mathcal{N}$ is the length of the sequence x_i and $\mathcal{N}_{\min}$ and $\mathcal{N}_{\max}$ the minimum and maximum length, respectively.

Alphabet composition

It is common to find some reads of a multi-FASTA format containing characters outside a desired set. For example, the 'R' symbol at the DNA sequence level stands for a purine (an A or G base). This characteristic is frequent with other symbols in protein sequences, leading to an unbalance between the set cardinality of the sequences. This ambiguity generates complexity in the analysis when using many software tools and can create deviant results. Therefore, the AltaiR method efficiently filters sequence symbols containing a certain alphabet. Additionally, it can filter the reads by composition, including completeness, when combined with the length.

Minimum and maximum GC percentage

The GC percentage is usually an important feature to evaluate if the distribution of a particular sequence is within a desirable average set. Specifically, the GC percentage is given by the number of cytosine (C) and guanine (G) bases in a string x^i with length $|x^i|$ according to

$$\mathcal{GC}(x^i) = \frac{100}{|x^i|}\sum_{j=1}^{|x^i|}\mathcal{I}_{\Xi}(x^i_j), \quad (2)$$

where x^i_j is each symbol of x^i (assuming causal order), Ξ is a subset alphabet containing the symbols $\{G, C\}$ and $\mathcal{I}$ an indicator function constrained to

$$\mathcal{I}_{\Xi}(x) = \begin{cases} 1, x \in \Xi \\ 0, x \notin \Xi \end{cases}. \quad (3)$$

Then, the set of final sequences, Σ, is composed by

$$x^i \in \Sigma \iff \mathcal{GC}_{\max} \geq \mathcal{GC}(x^i) \geq \mathcal{GC}_{\min}, \quad (4)$$

where $\mathcal{GC}_{\min}$ and $\mathcal{GC}_{\max}$ are two real values where $\mathcal{GC}_{\max} > \mathcal{GC}_{\min} \in [0;100]$.

Header patterns and anti-patterns

The header patterns are strings to match in each sequence header, y^i, that can ignore or consider a certain sequence read if the substring pattern is absent or present. This is the analogous process of the well-known grep tool, but instead of looking into the whole FASTA file, it only filters by the header sequence. This type of filtering is very important when only a certain type of sequence is to be considered or sequences with a certain header substring must not pass to the final set of sequences, Σ.

The AltaiR toolkit provides filtering of the header patterns using substrings that occur in the header or that are absent (ignore pattern) using conjoint filtering that is not limited to the number of strings to match or ignore. For example, using the patterns "Human", "Herpes" and "Alpha" and the anti-pattern "Simplex" in the whole viral reference database from the NCBI will select all the FASTA reads that the headers contain the words Human, Herpes, and Alpha, selecting only the human Alphaherpesvirus that are composed by the HSV-1 (Herpes Simplex Virus 1), HSV-2 (Herpes Simplex Virus 2), and VZV (Varicella-Zoster Virus); because the anti-pattern "Simplex" was also used, then, only the VZV will be contained in Σ.

Similarity (NCD) profiles

In temporal analysis, some applications require measuring the similarity of the most recent genomes to the first ones being sequenced and assembled, such as the first reference genome. These measures will allow us to understand the evolution rate over time and if evolutionary acceleration peaks have been found in specific periods. Accordingly, if the genomes are sorted according to sample isolation date (an NCBI VSSI option while downloading the data), then measuring the distance of the first genome according to the remaining in the multi-FASTA file provides this similarity information.

To compute the similarity between the reference genome and the remaining sequences, the Normalized Compression Distance (NCD), which is a similarity distance that approximates the Kolmogorov complexity through data compression, is used [57, 58, 59]. The NCD has many applications for genomic and proteomic assembled sequences [60], including the similarity measure for the COVID-19 pandemics [61, 62].

Formally, the NCD between a reference y and a target x sequence is given by

$$\mathcal{D}(x, y) = \frac{C(x, y) - \min\{C(x), C(y)\}}{\max\{C(x), C(y)\}}, \quad (5)$$

where $C(x)$ and $C(y)$ represents the number of bits needed to lossless compress x and y, respectively. The $C(x, y)$ represents the number of bits needed to compress x and y conjointly, which usually is approximated by string concatenation.

Since in our application, we are required to compute the whole distances of each x^i according to a reference y, then an array of distances is calculated for creating the NCD profile according to

$$\mathcal{D}_i(x^i, y) = \frac{C(x^i, y) - \min\{C(x^i), C(y)\}}{\max\{C(x^i), C(y)\}}. \quad (6)$$

Moreover, because the conjoint compression follows the commutative property $C(x^i, y) = C(y, x^i)$, it can be rewritten as

$$\mathcal{D}_i(x^i, y) = \frac{C(y, x^i) - \min\{C(x^i), C(y)\}}{\max\{C(x^i), C(y)\}}. \quad (7)$$

This change offers a substantial save in computational resources because it is now possible to compress y and, in the end, freeze its models. Then, the number of bits to compress y is saved, and for each x^i, the compression models are initialized with the frozen models of y. Therefore the complexity time to compute $C(y, x^1), ..., C(y, x^n)$ is now $y + xn$. This change is now affordable for an application considering millions of genomes.

The choice of the data compressor is fundamental to better approximating the Kolmogorov complexity and the NCD [63, 58]. Therefore, besides respecting the common distance characteristics [63] and distance density [58], the data compressors that are designed for the specific use of certain types of data offer a much higher approximation of the Kolmogorov complexity than general-purpose tools. This characteristic is provided by the specific-purpose methodology's ability to efficiently model characteristics that would otherwise not be seen with general-purpose models. For example, two characteristics that play a key role in biological sequences are inverted repeats [64] and high-level substitutions in repetitive data [65].

In the case of the COVID-19 pandemic, a specific-purpose data compressor does not offer a wide advantage over a general-purpose data compressor because, on average, the sequences contain high entropy, are small, and have only a few sequence differences between sequences. However, an efficient specific-data compressor is mandatory for larger genomes such as those from larger viruses (e.g. Herpesvirus), bacterial or fungi. Therefore, we use an implementation derived from GeCo3 [66] and AC2 [62] data compressors that have proven to be state-of-the-art data compressors in genomic and proteomic sequences, respectively. The disadvantage of these data compressors is the necessity to have a solid knowledge of the models for extensive optimization. We provide pre-computed models for different biological sequence types to minimise this disadvantage.

For applications where the size of the distances vector is very large or the variation of the instances in the profile high, AltaiR offers the possibility of averaging the signal through a moving average. The Toolkit implementation subsection provides more information about this option.

Complexity (NC) Profiles

Another important temporal analysis is the perception of how the (normalized) Kolmogorov complexity [59] of a certain set of sequences sorted by time varies. The normalized Kolmogorov complexity is approximated through the Normalized Compression

(NC) [67]. The NC is given by the ratio of the uncompressed quantity sum of bits by the size of the sequence representation assuming a uniform distribution. This analysis is analogous to understanding how the genome or proteome sequence entropy varies over time using a random reference.

As in the previous Subsection, the Kolmogorov complexity is approximated using specific-purpose data compression algorithms, namely with implementations derived from GeCo3 [66] and AC2 [62] for genome and proteome sequences, respectively.

Formally, the NC of a certain sequence is provided by

$$\mathcal{E}(x) = \frac{C(x)}{|x| \log_2(|\Xi|)}, \tag{8}$$

where $|x|$ is the size of the sequence and $|\Xi|$ the number of different symbols in the sequence x.

Since in our application, we require to compute the whole sequence complexity of each x^i, then an array of NCs is calculated for creating the NC profile according to

$$\mathcal{E}_i(x^i) = \frac{C(x^i)}{|x^i| \log_2(|\Xi^i|)}, \tag{9}$$

where $|\Xi^i|$ is the number of different symbols in the sequence x^i.

For genomic sequences, Ξ is 4 for any x^i. However, for proteomic sequences, Ξ may vary, creating changes in the profile. For these cases, alphabet trimming, which involves reducing the alphabet size by removing or substituting non-standard characters, can work as a solution to ensure a consistent alphabet size across all sequences and maintain the comparability of NC values.

For applications where the size of the NC vector is very large or the variation of the instances in the profile high, AltaiR offers the possibility of averaging the signal through a moving average. The Toolkit implementation subsection provides more information about this option.

Frequency profiles

One application that provides evidence of evolutionary events is the variation of the nucleotide or amino acid frequency over time. This information is given by the frequency profiles, defined as the percentage symbol distribution of each sequence.

Formally, the frequency of a certain symbol, s, from Σ in a sequence x is provided by

$$\mathcal{F}(s|x) = \frac{1}{|x|} \sum_{j=1}^{|x|} \mathcal{I}_s(x_j), \tag{10}$$

where $\mathcal{I}$ is an indicator function associated to the number of times that s is seen in x.

Since in our application, we require to compute the symbol frequency of each x^i, then an array of frequencies is calculated for creating the frequency profile according to

$$\mathcal{F}_i(s|x^i) = \frac{1}{|x^i|} \sum_{j=1}^{|x^i|} \mathcal{I}_s(x^i_j). \tag{11}$$

For applications where the size of the frequency vector is very large or the variation of the instances in the profile high, AltaiR offers the possibility of averaging the signal through a moving average. The Toolkit implementation subsection provides more information about this option.

Relative singularity (RAWs) profiles

Identifying the shortest sequence regions present in a set of pathogens but absent from the host genome and transcriptome is an important application in endemic and epidemic contexts. These regions, called Relative Absent Words (RAWs), are of interest because they can distinguish between lineages [68], localize higher GC-content regions, and have applications in optimized diagnosis [69]. Furthermore, RAWs can provide insights into the unique genomic characteristics of pathogens, potentially aiding in the development of targeted therapeutic interventions and diagnostic tools [69].

Specifically, RAWs [68, 69, 70] are a particular subset of Minimal Absent Words (MAWs) that have also been referred to as nullomers or forbidden words [71, 72, 73]. Many MAWs-based applications have been created, including optimized models for their detection [74] and the complementary simulation of sequences while avoiding the creation of MAWs [75].

MAWs in genomic and proteomic sequences have been studied [76]. For example, in viruses, MAWs have been found most frequently in regions that are restriction recognition sites [76]. On the other hand, the subset of MAWs (RAWs) has been found in regions with high GC content, contrary to the host and pathogen genome [69].

An approach to finding MAWs is through the flanking subsequence regions. Consider a set, X, constituted of n target sequences, $x^1, x^{\cdots}, x^n$, and a reference sequence, y, both drawn from the finite alphabet Σ. We say that β is a factor of x^i if x^i can be expressed as $x^i = u\beta v$, with uv denoting the concatenation between sequences u and v. We denote by $\mathcal{W}_k(x^i)$ the set of all k-size words (or factors) of x^i. Also, we represent the set of all k-size words *not in* x^i as $\overline{\mathcal{W}_k(x^i)}$. For each word size k, we define the set of all words that exist in x^i but do not exist in y by

$$\mathcal{R}_k(x^i, \bar{y}) = \mathcal{W}_k(x^i) \cap \overline{\mathcal{W}_k(y)}. \tag{12}$$

The subset of minimal words as

$$\mathcal{M}_k(x^i, \bar{y}) = \{\beta \in \mathcal{R}_k(x^i, \bar{y}) : \mathcal{W}_{k-1}(\beta) \cap \mathcal{M}_{k-1}(x^i, \bar{y}) = \emptyset\}, \tag{13}$$

i.e., a MAW of size k cannot contain any MAW of size less than k. In particular, $l\beta r$ is a MAW of sequence x^i, where l and r are single letters from Σ, if $l\beta r$ is not a word of x^i but both $l\beta$ and βr are. We have defined the non-empty set $\mathcal{M}_k(x^i, \bar{y})$ with the smallest k as minimal Relative Absent Words (mRAWs) [68].

Another subset of MAWs and RAWs are persistent mRAWs (PmRAWs) [69]. Formally, let r be a mRAW of $x^i \in X$ and $P(r, x^i)$ be the predicate "r is a RAW of string x^i". Then, if $\forall_{x^i \in X} P(r, x^i)$, we say that r is persistent in X. This property means the full conservation of the identified mRAWs across all the sequences. A particularity of these sequences is to consider PmRAWs at the whole genome level and at a sub-genome level, meaning that a x^i can be considered a subsequence of a whole genome, namely a gene, extending the power of PmRAWs to local observations.

The RAWs and PmRAWs have been computed with the EAGLE tools version 1 [68] and 2 for DNA sequences [69]. This methodology was included in the online detection of RAWs, namely the ADACT [77].

The AltaiR toolkit can compute the RAWs based on the same methodology as in EAGLE version 2. Still, it extends the capability to deal with any other types of sequences, for example, proteomes, as long as they respect the multi-FASTA format. Moreover, for computing the RAWs, the AltaiR toolkit can avoid writing into temporary files while maintaining low memory consumption. This capability enables the computation of RAWs in millions of genomes/proteomes while expending relatively low computational resources.

Moreover, the Altair toolkit enables the automatic computation

of the GC percentage for each RAW, providing the unprecedented ability to create large-scale studies with a higher divergence between the sequences.

Additionally, the AltaiR toolkit includes the capability to compute RAWs profiles. The RAWs profiles are generated with the additional combination of temporal metadata, namely through the time order usage of the target multi-FASTA file that can be downloaded with that property at the NCBI repository. These profiles directly compute the presence of each RAW according to the sequences sorted by temporal characterization.

Toolkit implementation

The AltaiR methodology is implemented in C language and contains no external dependencies. The source code and application result scripts are freely provided at the repository https://github.com/cobilab/altair. The AltaiR toolkit contains one main menu (command: AltaiR) with six sub-menus for computing the methods that it provides, sometimes through the combination of multiple, namely

- **average** - moving average filter of a column float CSV file (the column to use is a parameter);
- **filter** - filters FASTA reads by characteristics: alphabet, completeness, length, GC quantity, multiple string patterns and anti-patterns;
- **frequency** - computes the alphabet frequencies for each FASTA read (it enables alphabet filtering);
- **nc** - computes the Normalized Compression (NC) for all FASTA reads according to a compression level or specific parameters;
- **ncd** - computes the Normalized Compression Distance (NCD) for all FASTA reads according to a reference;
- **raw** - Computes Relative Absent Words (RAWs) with automatic GC percentage estimation for all RAWs.

The toolkit allows reading and writing from standard input and output, respectively, in certain cases. For example, this feature enables the direct piping of the output from the filtering tool to the NC, NCD, and RAWs tools, streamlining the analysis process. Additionally, it allows for the output profiles of the frequency, NC, NCD, and RAWs tools to be easily processed by other tools, such as those performing averaging or visualization. This functionality provides easier integration to build custom pipelines. Furthermore, most tools (except average) use multi-threading to speed up the analysis.

In the next section (results), we provide details of the toolkit features, including the most important parameters and commands to retrieve the results. Nevertheless, more documentation can be retrieved from the Supplementary material and in the README.md file in the code repository.

Results

As a result, we provide the application analysis of the AltaiR toolkit to the challenges described in the previous section. The primary motivation for using the SARS-CoV-2 data is to demonstrate AltaiR's capabilities in handling large-scale genomic datasets and uncover novel insights into the virus's evolution, adaptation, and interaction with the human host. All the results can be repeated using the procedures described in each specific analysis, except for the data collection retrieved manually from the NCBI Virus database. Despite this manual procedure, all the data is included as supplementary material for direct download. The dataset used to compute the results includes a large collection of SARS-CoV-2 genome sequences retrieved from the NCBI viral repository on 29 September 2022 [2]. The SARS-CoV-2 genomes have been filtered from a pool of 6,309,078 genomes according to quality and completeness, namely only considering complete genomes with the host as human, resulting in 1,538,095 genomes [78]. The dataset also contains multiple coronavirus genomes, the reference T2T human genome and transcriptome, and multiple computer-generated sequences; the latter works as an important validation procedure.

Filtering sequences

The SARS-CoV-2 dataset contains nearly 1.5 million sequences labelled as complete genomes in the NCBI viral repository. In the histogram depicted in Figure 2, we notice substantial differences in the length of the sequences. This histogram provides the frequency of the length of the sequences, which was computed considering symbols outside the alphabet $\Sigma = \{A, C, G, T\}$, such as the N symbols and others. Although the plot considers only the sequence length from 29.5k and 30k, there were several sequences with lengths lower than 20k. These smaller sequences were considered complete genomes and showcased the importance of filtering the data by the sequence length.

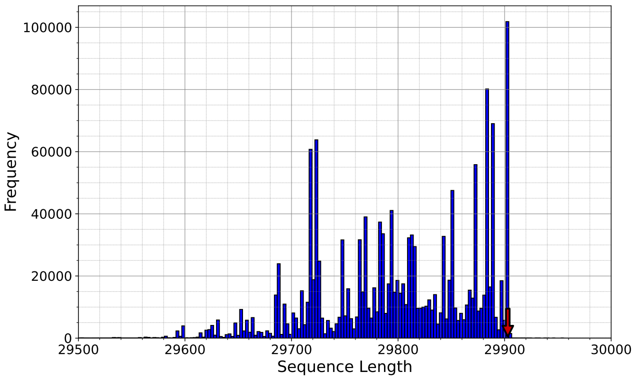

Figure 2. Histogram with the length of the genomes, considering all the existent symbols, from the SARS-CoV-2 dataset. The red arrow line stands for the size of the reference genome. To replicate this analysis, see Supplementary Section Reproducibility, specifically Reproducing the "Data filtering".

The SARS-CoV-2 reference genome (NC_045512.2) has a length of 29,903, and the largest cluster of sequences is almost coincident with this length (around 100k sequences). Therefore, since the SARS-CoV-2 genomes have low variability, this region is a good candidate for sequence length filtering.

However, the SARS-CoV-2 genomes contain a poly(A)-tale with a variable size in the end-tip. This poly(A)-tale is the only low-complexity region and, therefore, it is, ironically, the region more complex to sequence and assemble [69, 41]. To provide some flexibility to this region, we filtered the sequences considering the interval length of 29,885 to 29,921. Notice that many of these sequences contained symbols outside the $\{A, C, G, T\}$ alphabet, for example, N symbols, and, thus, many of them were removed when the total of alphabet symbols was not reached.

Moreover, we included filtering specific patterns and anti-patterns, namely considering only the sequences where the headers did not contain only the year (e.g. "|2020|") but also the month and, if available, the day associated with the virus isolation. Also, sequences without dates were discarded.

In sum, the sequence length and the pattern/anti-pattern decreased by 1.5 million to 25,594 sequences. Although the dataset was reduced substantially, the data quality substantially increased proportionally. Moreover, nearly 25k of high-quality sequences are sufficient for accurate downstream analysis.

This sequence filtering step shows its critical importance,

proven by the high variability between these sequences, which can harm downstream analyses without proper filtering. For instance, including incomplete, low-quality, or outlier sequences can introduce noise and bias in similarity measurements, leading to inaccurate clustering or phylogenetic inference. Inconsistent or missing metadata can hinder the interpretation of temporal patterns and evolutionary trends, while unusual sequence characteristics can skew statistical analyses and obscure biological patterns. As such, rigorous filtering ensures high data quality and consistency, minimizing spurious results and enabling reliable biological interpretations.

Similarity (NCD) profiles application

To benchmark the similarity profiles described in the Subsection Similarity (NCD) profiles, we recurred to two levels, namely, using synthetic and real data.

The synthetic data (denoted as the original sequence) has been computer-generated with the GTO toolkit [35] while assuming a uniform distribution and a length of 5000 DNA symbols. Using this original sequence, consecutive SNP mutations have been applied to the following sequences with a symbol mutation probability of 0.00005. To clarify, the original sequence (time-0) has been mutated while originating the sequence time-1, then the sequence time-1 has been mutated originating the sequence time-2, and this process was followed to the sequence time-10000. Finally, the temporal similarity profiles have been computed using a chosen time point as a reference. Supplementary Figure 1 provides the similarity (NCD) profiles for three time points, namely 0, 2500, and 4000.As depicted, the NCD profiles have a minimum value for the respective time point reference, showing the capability to identify the closest sequence in time under these simplified conditions.

Despite the performance of the previous approach, it is hard to find a real scenario where a time point sequence is perfectly available. In practice, this sequence already contains several mutations. Therefore, to simulate this characteristic, we repeat the above experience, but instead of using a direct time point sequence, we use a mutated time point sequence.

Accordingly, all the time point symbol sequences have been mutated with a probability of 0.05 (mutations in approximately 5% of the sequence) using a uniform distribution and different seeds. Supplementary Figure S2 provides the similarity (NCD) profiles for three-time points mutated, namely 0, 2500, and 4000. Although the average NCD value increased, the similarity is still comparatively high for the selected points. These results suggest that NCD profiles may serve as tolerant mutation measures to predict the temporal occurrence of sequences, but further investigation is required to validate this claim.

In addition to the synthetic data analysis, we also explored the application of NCD profiles to a real-world dataset, specifically focusing on the RaTG13 genome sequence and its similarity to SARS-CoV-2 sequences. This analysis, along with a discussion of its limitations and potential implications, can be found in the Supplementary Material. Applying NCD profiles to this real-world dataset demonstrates their ability to uncover interesting trends and relationships that can guide further research. While the results obtained from NCD profiles should be interpreted cautiously and validated through additional lines of evidence, they serve as a valuable starting point for more focused investigations and hypothesis generation.

It's also worth mentioning that from any NCD results, one can automatically construct a phylogenetic tree for the n closest sequences. The Supplementary Material under the Reproducibility Section illustrates an example of this tree, along with instructions for recreating it.

Complexity profiles (NC) application

Herein, we present a detailed complexity profile (NC) analysis of SARS-CoV-2 sequences using the AltaiR toolkit. By approximating the normalized Kolmogorov complexity through the Normalized Compression metric, we quantitatively assessed the informational content of the viral sequences over time.

The complexity profile, illustrated in Figure 3, captures the temporal fluctuations in the sequence complexity of the virus. The NC values were computed using 5, 20, and 100 window sizes to smooth the data and reveal underlying trends. The plot shows the NC trajectory, where each line represents the complexity calculated over a different window size.

The time frame from January 2020 to January 2022 was particularly interesting due to significant epidemiological events. Our analysis encapsulates this period, showing a dynamic yet subtle evolution of the virus's genomic makeup, namely an average decrease of sequence complexity over time. Usually, to accomplish very similar functions, lower entropy corresponds to less energy required and, hence, more efficiency which can be driven by evolution or selection.

Moreover, the plot indicates periods of relative stability interspersed with spikes and dips in complexity. Notice that some noise exists that can be related to the higher variability of the size of some sequences but that is contained in the filtered interval. On the other hand, some inflexion points may correlate with the emergence of new variants or adaptations in the virus's evolutionary strategy.

Recently, we introduced AlcoR [41]. AlcoR is a mapping and visualization tool for detecting low-complexity regions in biological data. AltaiR complements AlcoR in the sequence complexity analysis, namely by adding the capability to relate sequences using a temporal dimension.

Frequency profiles application

We used the filtered SARS-CoV-2 multi-FASTA to access the frequency application and computed its nucleotide variation over time, as shown in Figure 4.

The figure shows a notable shift in the nucleotide composition of SARS-CoV-2 over the observed period of time. Specifically, the frequency of thymine (T) bases has gradually increased, while there has been a (approximate) corresponding decrease in the frequency of cytosine (C) bases. This trend indicates the virus's ongoing evolutionary process within the human host.

The increase in T bases and the decrease in C bases can be attributed to various factors. Transition mutations, where a purine is substituted for another purine or a pyrimidine for another pyrimidine (in this case, C to T), are common mutations in RNA viruses. These mutations may be driven by the error-prone nature of RNA-dependent RNA polymerase, which is responsible for viral replication [79, 80, 81]. Additionally, such mutations may confer selective advantages to the virus, potentially impacting its transmissibility, pathogenicity, and immune escape capabilities [82, 83].

This trend also raises important considerations for public health and clinical interventions. For instance, changes in the viral genome could affect the efficacy of vaccines and therapeutics, emphasizing the need for continuous surveillance and adaptation of these measures [84, 85]. The observed mutations might also provide insights into the virus-host interaction dynamics, explaining how SARS-CoV-2 adapts to the human host environment over time [86].

Relative singularity (RAWs) profiles

Our study also focused on uncovering the Relative Absent Words (RAWs) of SARS-CoV-2, namely by identifying the shortest words that exist in the SARS-CoV-2 and were absent from the human

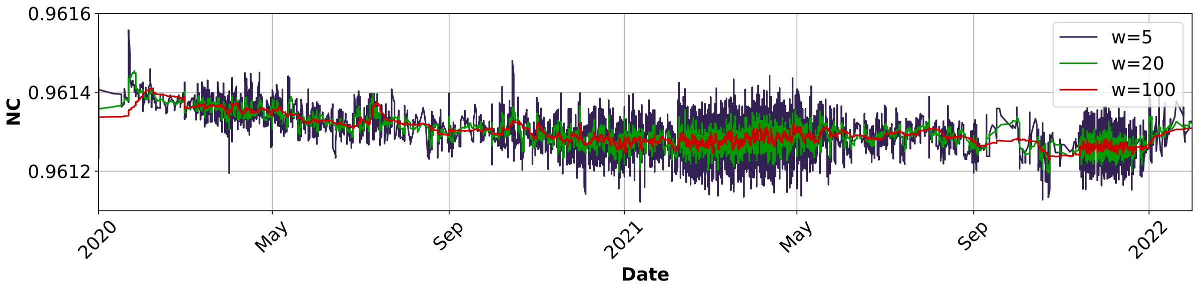

Figure 3. Complexity Plot showing variations in the normalized complexity (NC) values over time. The plot illustrates changes in NC with different window sizes, providing insights into the data trends from 2020 to 2022. To replicate this analysis, see Supplementary Section Reproducibility, specifically Reproducing the "Complexity profiles (NC) application"

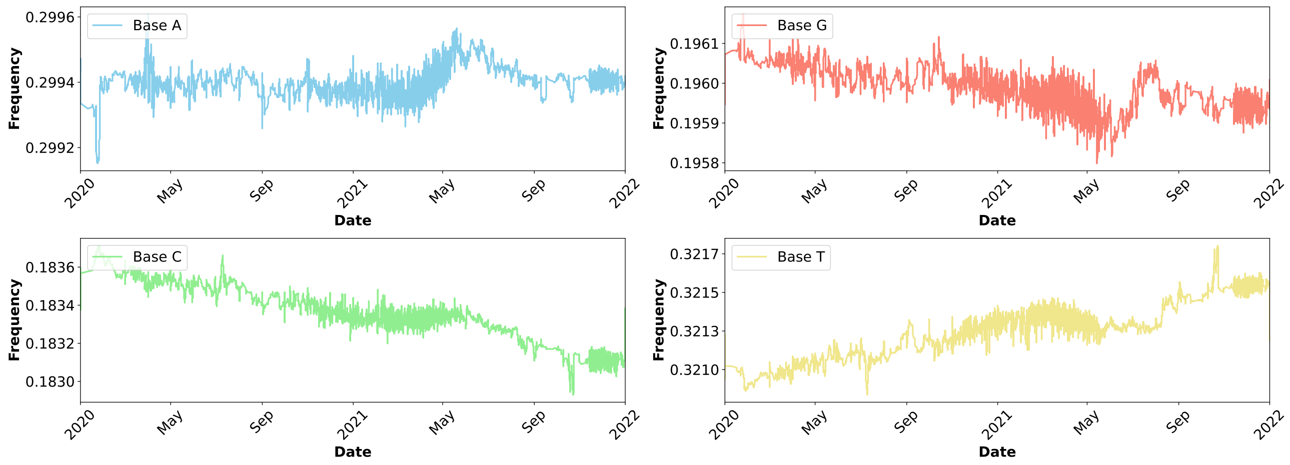

Figure 4. Frequency distribution of nucleotide bases in SARS-CoV-2 from 2020 to 2022, illustrating an increase in thymine (T) bases and a decrease in cytosine (C) bases. To replicate this analysis, see Supplementary Section Reproducibility, specifically Reproducing the "Frequency profiles application"

host genome [87] and transcriptome [88]. Detailed statistics of this analysis are presented in Table 1.

When analyzing the data in the table, it becomes evident that there is a notable shift in nucleotide distribution as the mRAW size increases in the SARS-CoV-2 genome. Specifically, we observe a decrease in guanine (G) and cytosine (C) percentages in larger mRAWs, indicating a declining prevalence of GC content as the sequence size expands. This trend suggests a gradual shift towards AT-rich sequences in the larger mRAWs of the virus.

Interestingly, this finding contrasts with the overall nucleotide composition trend observed in SARS-CoV-2, where globally, GC content approximates 40%, and AT content around 60%. This discrepancy between the overall nucleotide composition of SARS-CoV-2 and the elevated GC percentage in the shortest unique sequences absent from the human genome suggests distinct evolutionary adaptations or functional requirements in the viral genome. Higher GC content in these sequences may confer increased structural stability or efficiency in replication and transcription processes, potentially impacting the virus's interaction with host cellular mechanisms. This pattern may reflect evolutionary pressures shaping these specific genomic regions for optimized functionality within the host environment [89, 90, 91].

On the other hand, from a previous study [69] we found out that some of the mRAWs with high GC content were localized at the surface of important proteins, such as the Spike glycoprotein in the SARS-CoV-2. This region was then found to be related to segments where the protein presents higher dynamics in time, namely higher movement of the protein to interact with the host cell.

Figure 5 and Supplementary Figure S4, through relative singularity profiles, illustrate temporal variations in both the count of mRAWs and their average GC-content in the SARS-CoV-2 genomes. Between June and August 2021, the plots indicate significant shifts in the SARS-CoV-2 genome's mRAWs count and GC content, coinciding with the Delta variant's dominance. The unique mutations of the Delta variant likely drove these genomic changes, reflecting its impact on the virus's nucleotide composition and structure.

The AltaiR tool's efficiency in processing large datasets proves extremely useful for identifying virus evolutionary trends. Additionally, its capability to rapidly analyze and detect mRAWs holds significant potential for diagnostics, as these sequences can be targeted explicitly in tests for accurate and prompt detection of viral infection. Moreover, mRAWs profiles can be used as potential markers for detecting new variants.

Computational efficiency

To evaluate the computational performance of the AltaiR toolkit, using only a single computational thread, we measured the average and total execution times for each of its main methods: filtering, NC calculation, frequency calculation, NCD calculation, and RAWs calculation (Table 2).

Filtering sequences based on length, GC content, and specific patterns took 0.0004 seconds per sequence. NC calculation, which generates Nucleotide Complexity profiles, required an average of 0.0050 seconds per sequence (total 128.26 seconds). Frequency calculation, analyzing nucleotide composition, was the fastest at 0.0001 seconds per sequence on average (total 3.70 seconds). NCD calculation took an average of 0.2294 seconds per sequence (total 2294.13 seconds). RAWs calculation was the most computationally intensive method, requiring an average of 61.5580 seconds per sequence and 1538.95 seconds for the entire dataset. This is expected, as the RAWs method involves complex string matching and

Table 1. Output statistics of AltaiR while computing the RAWs of SARS-CoV-2 that are absent from the human genome and transcriptome.

	Overall mRAWs Statistics			mRAWs Nucleotide Distribution				mRAWs AT/CG Distribution	
**kmer**	**Average**	**Variance**	**Std. Dev.**	**A**	**C**	**G**	**T**	**AT%**	**CG%**
11	0.00	0.00	0.01	3	3	4	1	36.4%	63.6%
12	8.29	0.39	0.62	584,523	561,473	720,587	679,229	49.6%	50.4%
13	119.70	11.26	3.36	8,500,431	10,168,609	11,774,411	9,386,300	44.9%	55.1%
14	726.01	46.92	6.85	60,566,946	65,041,615	67,333,051	67,209,832	49.1%	50.9%
15	2813.56	147.05	12.13	269,913,035	251,842,372	260,427,726	298,014,612	52.6%	47.4%
16	8132.19	227.32	15.08	900,499,378	717,952,376	740,116,031	971,728,183	56.2%	43.8%

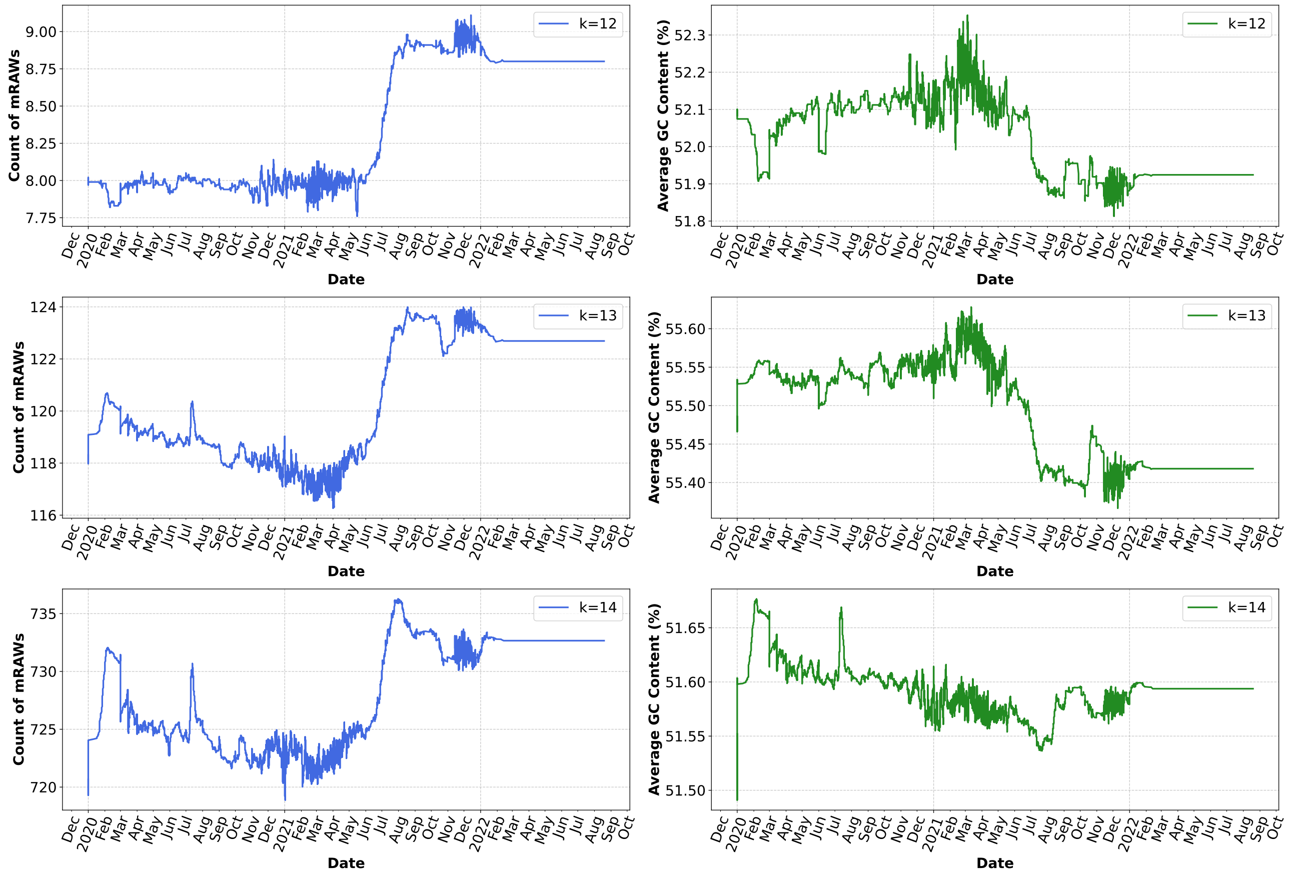

Figure 5. Relative singularity (RAWs) profiles: This set of plots illustrates the variation over time in the number of mRAWs and the average GC content in SARS-CoV-2 sequences. Each subplot corresponds to a different k-mer size (k=12, 13 and 14), showcasing the distribution of mRAWs and GC percentage across various time points. The x-axis represents time, segmented into years and months, while the y-axis shows the count of mRAWs and the GC content percentage, respectively. To replicate this analysis, see Supplementary Section Reproducibility, specifically Reproducing the "Relative singularity (RAWs) profiles"

Table 2. Average computation times for each method in the AltaiR toolkit.

Method	Average time per sequence (seconds)	Total time (seconds)
Filtering	0.0004	598.435
NC	0.005	128.2557
Frequency	0.0001	3.701
NCD	0.229	2294.132
RAWs	61.558	1538.950

comparison operations to identify unique substrings. The modular design of AltaiR allows for the integration of these methods into customizable pipelines, enabling researchers to select the most relevant analyses for their specific studies. The toolkit's ability to efficiently process large datasets, with most methods requiring less than a second per sequence on average, makes it well-suited for the rapidly growing field of genomic data analysis. While the RAWs calculation is more computationally demanding, its unique capabilities in identifying distinctive genomic elements justify its inclusion in the toolkit. These results showcase the overall computational efficiency of AltaiR, demonstrating its potential to streamline the analysis of large-scale genomic datasets across various research domains, from infectious disease studies to broader investigations in biology and genetics.

Discussion

The development of the AltaiR toolkit responds to the growing need for advanced tools capable of analyzing large and complex genomic datasets. This need arises not only from studies on infectious diseases but also from the broader fields of biology and genetics, where researchers are increasingly focusing on diverse organisms, including viruses, bacteria, protozoa, plants, and eukaryotes.

To contextualize AltaiR's capabilities, we performed a qualitative analysis with established toolkits—SeqAn [32], khmer [33, 34],

Feature / Toolkit	AltaiR	SeqAn	khmer	GTO	HaploCoV	bjorn
**Programming Language**	C	C++	Python, C++	C	Perl	Python, Bash
**Parallel Computing**	✓(Multi-threading)	✓	✗	✓	✗	✗
**Alignment-Free Analysis**	✓	✗	✓	✓✗(some cases)	✗	✗
**Genome Filtering**	Advanced (by length, GC content, patterns)	Basic filtering capabilities	Basic filtering capabilities	Basic filtering capabilities	✗	✗
**NCD Profile**	✓	✗	✗	✓✗(with scripting alterations)	✗	✗
**Complexity Profiles (NC)**	✓	✗	✗	✓	✗	✗
**Nucleotide Frequency Analysis**	✓	✓	✓✗(k-mer frequencies)	✓	✗	✗
**RAW Identification**	✓	✗	✗	✗	✗	✗
**Large-Scale Data Handling**	Optimized for millions of sequences	Large data handling but less optimized	Designed for large datasets but focused on k-mer analysis	✗	Designed for large SARS-CoV-2 datasets	Designed for SARS-CoV-2 datasets
**External Dependencies**	None	Some libraries required	Python environment and dependencies	None (if not considering the external tools)	Perl modules	Python package, Docker
**Modular Toolkit Design**	✓	✓	✗	✓	✗	✗
**FASTA Format Support**	✓	✓	✓	✓	✓	✓
**K-mer Counting and Filtering**	✗	✗	✓	✗	✗	✗
**String Matching Algorithms**	✗	✓	✗	✗	✗	✗
**Haplotype Reconstruction**	✗	✗	✗	✗	✓	✗
**Lineage Assignment**	✗	✗	✗	✗	✗	✓

Table 3. Comparative analysis of AltaiR with SeqAn, khmer, GTO, HaploCoV, and bjorn.

GTO [35], HaploCoV [92], and bjorn [93]. Table 3 shows the results of this comparison focusing on key functionalities, efficiency metrics, and capabilities crucial for handling large-scale genomic data.

AltaiR's implementation in C allows for efficient execution and memory management, making it well-suited for handling large genomic datasets. Its support for multi-threading enables parallel computing, resulting in faster processing times compared to toolkits like khmer, HaploCoV [92, 94], and bjorn [93], which lack built-in parallel computing capabilities. AltaiR's strength lies in its alignment-free approach, which is particularly effective for processing large-scale genomic data. While alignment-free methods offer significant advantages in terms of computational efficiency and the ability to handle large-scale genomic datasets, they may not provide the same level of detailed information as alignment-based methods. Researchers should consider the trade-offs between computational efficiency and the depth of information required for their specific research questions when choosing between these approaches.

One of AltaiR's core strengths is its focus on alignment-free analysis methods, such as Normalized Compression Distance (NCD) profiles and Nucleotide Complexity (NC) profiles. These techniques enable efficient and scalable comparisons of genomic sequences without relying on computationally expensive alignment operations, setting it apart from toolkits like SeqAn, which primarily concentrates on alignment algorithms. The toolkit's use of NCD for constructing similarity profiles enables a detailed exploration of temporal patterns in genomic data, providing insights into the genomic evolution of organisms over time. Additionally, AltaiR includes a feature for generating complexity profiles using NC, which is essential for quantitatively assessing the complexity of genomic sequences over time, providing insights into evolutionary pressures and adaptive responses in different organisms.

AltaiR's ability to filter sequences rigorously was demonstrated in the analysis of the SARS-CoV-2 dataset. It allows users to filter sequences based on length, GC content, and specific patterns, offering more comprehensive and customizable functionality compared to the filtering capabilities provided by SeqAn, khmer, and GTO, while HaploCoV and bjorn do not provide explicit filtering options. Like SeqAn, khmer, and GTO, AltaiR performs nucleotide frequency analysis. However, it extends this functionality with its unique NCD profile and RAW (Relative Absent Words) identifica-

tion capability, enabling the detection of rare and atypical words within genomic sequences, a feature not found in the other toolkits. The frequency profile feature, which tracks changes in nucleotide composition, can be used to study molecular adaptations in various organisms and understand their evolutionary biology. AltaiR is optimized for processing millions of sequences efficiently, specifically targeting alignment-free methods. While SeqAn and GTO also handle large datasets, khmer focuses on k-mer analysis, and HaploCoV [92] and bjorn [93] are designed specifically for SARS-CoV-2 datasets. AltaiR's optimization is tailored towards its specific strengths, following a modular toolkit design that allows for flexibility and adaptability in genomic data analysis workflows, similar to SeqAn and GTO. HaploCoV and bjorn are specialized tools for analyzing SARS-CoV-2 datasets, with HaploCoV focusing on haplotype reconstruction and bjorn providing lineage assignment capabilities. These specialized tools complement the broader functionality offered by AltaiR and the other general-purpose toolkits.

A unique capability of AltaiR is its ability to identify RAWs in genomic and proteomic sequences. By comparing RAWs in pathogens with those in host genomes, AltaiR can uncover distinctive genomic and proteomic elements, with potential applications in pathogen-host interaction studies and the discovery of new genomic and proteomic markers for diagnostic and therapeutic purposes, such as through the combination with aptamers. Aptamers, consisting of short sequences of DNA, RNA, or peptides, serve as molecular tools capable of binding to specific target molecules or families of target molecules. They can modulate the function of specific proteins, influencing signalling pathways or exerting inhibitory or enhancing effects [95]. Notably, aptamers have shown promise in therapeutic applications, as highlighted by Keefe et al. [96]. To discover high-affinity aptamers, contemporary computational methodologies leverage deep learning in conjunction with relevant feature extraction techniques, as Emami et al. [97]. The untapped potential of using mRAWs as pertinent features in aptamer discovery remains uncertain, but their distinctive characteristics suggest the possibility of streamlining subsequent phases of drug discovery and bypassing certain hurdles in the quest for novel therapeutic agents.

The versatility of AltaiR's sub-tools, coupled with its programming efficiency, holds significant importance for seamless integration into intricate pipelines involving multiple tools. This applicability is particularly noteworthy in various domains, such as drug discovery, viral genome reconstruction and analysis, and diversity analysis [98, 99, 100].

Consequentially, AltaiR stands out as a comprehensive and efficient toolkit for alignment-free analysis of large-scale genomic datasets. Its unique features, optimization for handling millions of sequences, and broad applicability position it as a powerful resource for a wide range of genomic and proteomic research endeavours, complementing the functionalities offered by other established toolkits and specialized tools.

Conclusions

In this study, we introduced AltaiR, a versatile and robust toolkit designed for the advanced analysis of large-scale genomic datasets. AltaiR's alignment-free methodology efficiently manages extensive data, exemplified by its role in analyzing a large number of SARS-CoV-2 sequences. The toolkit's capabilities, including Normalized Compression Distance (NCD) profiling, Normalized Compression (NC) analysis, temporal nucleotide composition variation, and the identification of Relative Absent Words (RAWs), showcase its adaptability to diverse genomic data types and research requirements.

AltaiR's innovation lies in the integration of well-established filtering methods, such as sequence length, GC content, and pattern matching, with novel and enhanced features into a comprehensive framework designed for efficient, large-scale genomic data analysis. The toolkit introduces new methods, including the Frequency method for detailed nucleotide or amino acid occurrence analysis, and extends the capabilities of the Filter method by supporting multiple simultaneous pattern searches, absent pattern searches, and filtering by GC content, sequence length, or completeness.

Moreover, AltaiR's NCD method introduces a novel approach that uses a conjunction of reference and target files while freezing the models of one and saving computation time for other sequences. This methodology allows for substantial time savings in NCD calculations. The NC analysis provides a quantitative assessment of the complexity of genomic sequences over time, offering insights into evolutionary pressures and adaptive responses in different organisms.

A unique capability of AltaiR is its ability to identify RAWs in genomic and proteomic sequences. The RAWs method in AltaiR supports genomic and protein sequences. By comparing RAWs in pathogens with those in host genomes, AltaiR can uncover distinctive genomic and proteomic elements, with potential applications in pathogen-host interaction studies and the discovery of new genomic and proteomic markers for diagnostic and therapeutic purposes.

The combination of these tools, along with the optimization for analyzing millions of sequences, ensures high portability and ease of installation while providing the necessary capabilities for rigorous analyses. This versatility enables a streamlined workflow, allowing researchers to process raw, unfiltered data and obtain meaningful insights without the need for multiple, disconnected tools.

AltaiR's application in studying SARS-CoV-2 genomes has provided possible insights into the virus's evolution and adaptations. However, the potential of AltaiR extends beyond virology, making it a crucial tool in broader genomic and proteomic research, such as in the analysis of resistant bacteria, unravelling complex temporal patterns, and facilitating studies on the evolution and diversity of various organisms.

As genomic research advances, driven by technological advancements and the increasing complexity of biological data, AltaiR's scalability, and efficiency position it as a powerful resource for a wide range of genomic and proteomic research.

Availability of source code and requirements

- Project name: AltaiR
- Project home page: https://github.com/cobilab/altair
- Operating system(s): Linux
- Programming language: C; Python; Bash
- Other requirements: Conda v4.3.27.
- License: e.g. GPLv3 License.
- RRID: SCR_024752
- Biotools: altair

Declarations

List of abbreviations

DNA - Deoxyribonucleic acid;
HSV-1 - Herpes Simplex Virus 1;
HSV-2 - Herpes Simplex Virus 2;
ID - Identifier;
MAW - minimal absent word;
mRAW - minimal Relative Absent Word;
PmRAW - Persistent minimal Relative Absent Word;
NC - Normalized Compression;
NCD - Normalized Compression Distance;
RAW - Relative Absent Word;
RNA - Ribonucleic acid;

T2T - Telomere-2-Telomere;
VZV - Varicella-Zoster Virus;

Competing Interests

The authors declare no competing interests.

Funding

This work was partially funded by National Funds through the FCT - Foundation for Science and Technology, in the context of the project UIDB/00127/2020. J.M.S. has received funding from the EC under grant agreement 101081813, Genomic Data Infrastructure. D.P. is funded by national funds through FCT – Fundação para a Ciência e a Tecnologia, I.P., under the Scientific Employment Stimulus - Institutional Call - reference CEECINST/00026/2018.

Acknowledgements

The authors wish to thank the Finnish Computing Competence Infrastructure (FCCI) for supporting this project with computational and data storage resources.

Author's Contributions

D.P. and A.P. designed the experiment. D.P. and J.M.S. coded the tools. J.M.S. executed the data analysis. J.M.S. and D.P. discussed the results. J.M.S. and D.P. wrote the manuscript. All authors have revised the manuscript.

References

1. Khare S, Gurry C, Freitas L, Schultz MB, Bach G, Diallo A, et al. GISAID's Role in Pandemic Response. China CDC Weekly 2021;3(49):1049.
2. Hatcher EL, Zhdanov SA, Bao Y, Blinkova O, Nawrocki EP, Ostapchuck Y, et al. Virus Variation Resource–improved response to emergent viral outbreaks. Nucleic acids research 2017;45(D1):D482–D490.
3. Cochrane G, Karsch-Mizrachi I, Takagi T, Sequence Database Collaboration IN. The international nucleotide sequence database collaboration. Nucleic acids research 2016;44(D1):D48–D50.
4. Sayers EW, Cavanaugh M, Clark K, Pruitt KD, Schoch CL, Sherry ST, et al. GenBank. Nucleic acids research 2021;49(D1):D92–D96.
5. Harrison PW, Ahamed A, Aslam R, Alako BT, Burgin J, Buso N, et al. The european nucleotide archive in 2020. Nucleic acids research 2021;49(D1):D82–D85.
6. Okido T, Kodama Y, Mashima J, Kosuge T, Fujisawa T, Ogasawara O. DNA Data Bank of Japan (DDBJ) update report 2021. Nucleic acids research 2022;50(D1):D102–D105.
7. de Vries JJ, Brown JR, Couto N, Beer M, Le Mercier P, Sidorov I, et al. Recommendations for the introduction of metagenomic next-generation sequencing in clinical virology, part II: bioinformatic analysis and reporting. Journal of Clinical Virology 2021;138:104812.
8. López-Labrador FX, Brown JR, Fischer N, Harvala H, Van Boheemen S, Cinek O, et al. Recommendations for the introduction of metagenomic high-throughput sequencing in clinical virology, part I: Wet lab procedure. Journal of Clinical Virology 2021;134:104691.
9. Simon-Loriere E, Holmes EC. Why do RNA viruses recombine? Nature Reviews Microbiology 2011;9(8):617–626.
10. Li X, Giorgi EE, Marichannegowda MH, Foley B, Xiao C, Kong XP, et al. Emergence of SARS-CoV-2 through recombination and strong purifying selection. Science advances 2020;6(27):eabb9153.
11. Palmenberg AC, Spiro D, Kuzmickas R, Wang S, Djikeng A, Rathe JA, et al. Sequencing and analyses of all known human rhinovirus genomes reveal structure and evolution. Science 2009;324(5923):55–59.
12. Plummer M, de Martel C, Vignat J, Ferlay J, Bray F, Franceschi S. Global burden of cancers attributable to infections in 2012: a synthetic analysis. The Lancet Global Health 2016;4(9):e609–e616.
13. Farrell PJ. Epstein–Barr virus and cancer. Annual Review of Pathology: Mechanisms of Disease 2019;14:29–53.
14. Smatti MK, Cyprian FS, Nasrallah GK, Al Thani AA, Almishal RO, Yassine HM. Viruses and autoimmunity: a review on the potential interaction and molecular mechanisms. Viruses 2019;11(8):762.
15. Pyöriä L, Pratas D, Toppinen M, Hedman K, Sajantila A, Perdomo MF. Unmasking the tissue-resident eukaryotic DNA virome in humans. Nucleic Acids Research 2023;51(7):3223–3239.
16. Toppinen M, Sajantila A, Pratas D, Hedman K, Perdomo MF. The Human Bone Marrow Is Host to the DNAs of Several Viruses. Frontiers in cellular and infection microbiology 2021;11:329.
17. Toppinen M, Pratas D, Väisänen E, Söderlund-Venermo M, Hedman K, Perdomo MF, et al. The landscape of persistent human DNA viruses in femoral bone. Forensic Science International: Genetics 2020;48:102353.
18. Land M, Hauser L, Jun SR, Nookaew I, Leuze MR, Ahn TH, et al. Insights from 20 years of bacterial genome sequencing. Functional & integrative genomics 2015;15(2):141–161.
19. Nurk S, Koren S, Rhie A, Rautiainen M, Bzikadze AV, Mikheenko A, et al. The complete sequence of a human genome. Science 2022;376(6588):44–53.
20. Qi W, Lim YW, Patrignani A, Schläpfer P, Bratus-Neuenschwander A, Grüter S, et al. The haplotype-resolved chromosome pairs of a heterozygous diploid African cassava cultivar reveal novel pan-genome and allele-specific transcriptome features. GigaScience 2022;11.
21. Mc Cartney AM, Shafin K, Alonge M, Bzikadze AV, Formenti G, Fungtammasan A, et al. Chasing perfection: validation and polishing strategies for telomere-to-telomere genome assemblies. Nature Methods 2022;p. 1–9.
22. Alkan C, Carbone L, Dennis M, Ernst J, Evrony G, Girirajan S, et al. Implications of the first complete human genome assembly. Genome Research 2022;32(4):595.
23. Vinga S, Almeida J. Alignment-free sequence comparison—a review. Bioinformatics 2003;19(4):513–523.
24. Reinert G, Chew D, Sun F, Waterman MS. Alignment-free sequence comparison (I): statistics and power. Journal of Computational Biology 2009;16(12):1615–1634.
25. Wan L, Reinert G, Sun F, Waterman MS. Alignment-free sequence comparison (II): theoretical power of comparison statistics. Journal of Computational Biology 2010;17(11):1467–1490.
26. Zielezinski A, Vinga S, Almeida J, Karlowski WM. Alignment-free sequence comparison: benefits, applications, and tools. Genome biology 2017;18(1):1–17.
27. Zielezinski A, Girgis HZ, Bernard G, Leimeister CA, Tang K, Dencker T, et al. Benchmarking of alignment-free sequence comparison methods. Genome biology 2019;20(1):1–18.
28. Metcalf JL, Xu ZZ, Bouslimani A, Dorrestein P, Carter DO, Knight R. Microbiome tools for forensic science. Trends in biotechnology 2017;35(9):814–823.
29. Chen Z, Zhao P, Li F, Marquez-Lago TT, Leier A, Revote J, et al. iLearn: an integrated platform and meta-learner for feature engineering, machine-learning analysis and modeling

of DNA, RNA and protein sequence data. Briefings in bioinformatics 2020;21(3):1047–1057.

30. Yang J, Chen X, McDermaid A, Ma Q. DMINDA 2.0: integrated and systematic views of regulatory DNA motif identification and analyses. Bioinformatics 2017;33(16):2586–2588.
31. Min JL, Hemani G, Davey Smith G, Relton C, Suderman M. Meffil: efficient normalization and analysis of very large DNA methylation datasets. Bioinformatics 2018;34(23):3983–3989.
32. Döring A, Weese D, Rausch T, Reinert K. SeqAn an efficient, generic C++ library for sequence analysis. BMC bioinformatics 2008;9(1):1–9.
33. Crusoe MR, Alameldin HF, Awad S, Boucher E, Caldwell A, Cartwright R, et al. The khmer software package: enabling efficient nucleotide sequence analysis. F1000Research 2015;4.
34. Standage D, Aliyari A, Cohen LJ, Crusoe MR, Head T, Irber L, et al. khmer release v2.1: software for biological sequence analysis. Journal of Open Source Software 2017;2(15):272. https://doi.org/10.21105/joss.00272.
35. Almeida JR, Pinho AJ, Oliveira JL, Fajarda O, Pratas D. GTO: a toolkit to unify pipelines in genomic and proteomic research. SoftwareX 2020;12:100535.
36. Drezen E, Rizk G, Chikhi R, Deltel C, Lemaitre C, Peterlongo P, et al. GATB: genome assembly & analysis tool box. Bioinformatics 2014;30(20):2959–2961.
37. Lee J, Lee AJ, Lee JK, Park J, Kwon Y, Park S, et al. Mutalisk: a web-based somatic MUTation AnaLyIS toolKit for genomic, transcriptional and epigenomic signatures. Nucleic acids research 2018;46(W1):W102–W108.
38. Sims D, Ilott NE, Sansom SN, Sudbery IM, Johnson JS, Fawcett KA, et al. CGAT: computational genomics analysis toolkit. Bioinformatics 2014;30(9):1290–1291.
39. Hiltemann S, Mei H, de Hollander M, Palli I, van der Spek P, Jenster G, et al. CGtag: complete genomics toolkit and annotation in a cloud-based Galaxy. GigaScience 2014;3(1):2047–217X.
40. de Koning W, Miladi M, Hiltemann S, Heikema A, Hays JP, Flemming S, et al. NanoGalaxy: Nanopore long-read sequencing data analysis in Galaxy. GigaScience 2020;9(10):giaa105.
41. Silva JM, Qi W, Pinho AJ, Pratas D. AlcoR: alignment-free simulation, mapping, and visualization of low-complexity regions in biological data. GigaScience 2023;.
42. Loman NJ, Quinlan AR. Poretools: a toolkit for analyzing nanopore sequence data. Bioinformatics 2014;30(23):3399–3401.
43. Knight R, Maxwell P, Birmingham A, Carnes J, Caporaso JG, Easton BC, et al. PyCogent: a toolkit for making sense from sequence. Genome biology 2007;8(8):1–16.
44. Shen W, Le S, Li Y, Hu F. SeqKit: a cross-platform and ultrafast toolkit for FASTA/Q file manipulation. PloS one 2016;11(10):e0163962.
45. Alam KK, Chang JL, Burke DH. FASTAptamer: a bioinformatic toolkit for high-throughput sequence analysis of combinatorial selections. Molecular Therapy-Nucleic Acids 2015;4:e230.
46. Ott M, Edunov S, Baevski A, Fan A, Gross S, Ng N, et al. fairseq: A Fast, Extensible Toolkit for Sequence Modeling. In: Proceedings of the 2019 Conference of the North American Chapter of the Association for Computational Linguistics (Demonstrations) Minneapolis, Minnesota: Association for Computational Linguistics; 2019. p. 48–53. https://aclanthology.org/N19-4009.
47. Chen C, Chen H, Zhang Y, Thomas HR, Frank MH, He Y, et al. TBtools: an integrative toolkit developed for interactive analyses of big biological data. Molecular plant 2020;13(8):1194–1202.
48. Gabler F, Nam SZ, Till S, Mirdita M, Steinegger M, Söding J, et al. Protein sequence analysis using the MPI bioinformatics toolkit. Current Protocols in Bioinformatics 2020;72(1):e108.
49. Arkin AP, Cottingham RW, Henry CS, Harris NL, Stevens RL, Maslov S, et al. KBase: the United States department of energy systems biology knowledgebase. Nature biotechnology 2018;36(7):566–569.
50. Kryukov K, Ueda MT, Nakagawa S, Imanishi T. Nucleotide Archival Format (NAF) enables efficient lossless reference-free compression of DNA sequences. Bioinformatics 2019;35(19):3826–3828.
51. Deorowicz S, Danek A, Li H. AGC: compact representation of assembled genomes with fast queries and updates. Bioinformatics 2023;39(3):btad097.
52. Grabowski S, Kowalski TM. MBGC: Multiple Bacteria Genome Compressor. GigaScience 2022;11.
53. Pinho AJ, Pratas D. MFCompress: a compression tool for FASTA and multi-FASTA data. Bioinformatics 2014;30(1):117–118.
54. Hosseini M, Pratas D, Pinho AJ. Cryfa: a secure encryption tool for genomic data. Bioinformatics 2019;35(1):146–148.
55. Lu J, Salzberg SL. Removing contaminants from databases of draft genomes. PLoS computational biology 2018;14(6):e1006277.
56. Sajantila A, Editors' Pick: Contamination has always been the issue! BioMed Central; 2014.
57. Li M, Chen X, Li X, Ma B, Vitányi PM. The similarity metric. IEEE transactions on Information Theory 2004;50(12):3250–3264.
58. Li M, Vitányi P, et al. An introduction to Kolmogorov complexity and its applications, vol. 3. Springer; 2008.
59. Kolmogorov AN. Three approaches to the quantitative definition of information. Problems of information transmission 1965;1(1):1–7.
60. Pratas D, Silva RM, Pinho AJ. Comparison of compression-based measures with application to the evolution of primate genomes. Entropy 2018;20(6):393.
61. Cilibrasi RL, Vitányi PM. Fast Phylogeny of SARS-CoV-2 by Compression. Entropy 2022;24(4):439.
62. Silva M, Pratas D, Pinho AJ. AC2: An Efficient Protein Sequence Compression Tool Using Artificial Neural Networks and Cache-Hash Models. Entropy 2021;23(5):530.
63. Cebrián M, Alfonseca M, Ortega A. Common pitfalls using the normalized compression distance: What to watch out for in a compressor. Communications in Information & Systems 2005;5(4):367–384.
64. Hosseini M, Pratas D, Pinho AJ. On the role of inverted repeats in DNA sequence similarity. In: International Conference on Practical Applications of Computational Biology & Bioinformatics Springer; 2017. p. 228–236.
65. Pratas D, Hosseini M, Pinho AJ. Substitutional tolerant Markov models for relative compression of DNA sequences. In: International Conference on Practical Applications of Computational Biology & Bioinformatics Springer; 2017. p. 265–272.
66. Silva M, Pratas D, Pinho AJ. Efficient DNA sequence compression with neural networks. GigaScience 2020;9(11):giaa119.
67. Pratas D, Pinho AJ. On the approximation of the Kolmogorov complexity for DNA sequences. In: Iberian Conference on Pattern Recognition and Image Analysis Springer; 2017. p. 259–266.
68. Silva RM, Pratas D, Castro L, Pinho AJ, Ferreira PJ. Three minimal sequences found in Ebola virus genomes and absent from human DNA. Bioinformatics 2015;31(15):2421–2425.
69. Pratas D, Silva JM. Persistent minimal sequences of SARS-CoV-2. Bioinformatics 2020 07;36(21):5129–5132.
70. Pratas D. Compression and analysis of genomic data. PhD thesis, Universidade de Aveiro (Portugal); 2016.
71. Béal MP, Mignosi F, Restivo A. Minimal forbidden words and symbolic dynamics. In: Annual Symposium on Theoretical Aspects of Computer Science Springer; 1996. p. 555–566.
72. Crochemore M, Mignosi F, Restivo A. Automata and Forbidden Words. Inf Process Lett 1998 Aug;67(3):111–117.

73. Pinho AJ, Ferreira PJ, Garcia SP, Rodrigues JM. On finding minimal absent words. BMC bioinformatics 2009;10(1):1–11.
74. Kosche M, Koß T, Manea F, Siemer S. Absent subsequences in words. In: International Conference on Reachability Problems Springer; 2021. p. 115–131.
75. Bernardini G, Marchetti-Spaccamela A, Pissis SP, Stougie L, Sweering M. Constructing strings avoiding forbidden substrings. In: 32nd Annual Symposium on Combinatorial Pattern Matching (CPM 2021), vol. 191; 2021. p. 1–9.
76. Koulouras G, Frith MC. Significant non-existence of sequences in genomes and proteomes. Nucleic acids research 2021;49(6):3139–3155.
77. Akon M, Akon M, Kabir M, Rahman MS, Rahman MS. ADACT: a tool for analysing (dis) similarity among nucleotide and protein sequences using minimal and relative absent words. Bioinformatics 2021;37(10):1468–1470.
78. NCBI, NCBI Virus. NCBI; 2023. https://www.ncbi.nlm.nih.gov/labs/virus/vssi/#/virus?SeqType_s=Nucleotide&VirusLineage_ss=Wuhan%20seafood%20market%20pneumonia%20virus,%20taxid:2697049&Completeness_s=complete&HostLineage_ss=Homo%20sapiens%20(human),%20taxid:9606&CreateDate_dt=2000-01-01T00:00:00.00Z%20TO%202022-09-28T23:59:59.00Z, [Online; accessed 27-November-2023].
79. Singh D, Yi SV. On the origin and evolution of SARS-CoV-2. Experimental & Molecular Medicine 2021;53(4):537–547.
80. Drake JW, Holland JJ. Mutation rates among RNA viruses. Proceedings of the National Academy of Sciences 1999;96(24):13910–13913.
81. Sanjuán R, Nebot MR, Chirico N, Mansky LM, Belshaw R. Viral mutation rates. Journal of virology 2010;84(19):9733–9748.
82. Grubaugh ND, Hanage WP, Rasmussen AL. Making sense of mutation: what D614G means for the COVID-19 pandemic remains unclear. Cell 2020;182(4):794–795.
83. Harvey WT, Carabelli AM, Jackson B, Gupta RK, Thomson EC, Harrison EM, et al. SARS-CoV-2 variants, spike mutations and immune escape. Nature Reviews Microbiology 2021;19(7):409–424.
84. Korber B, Fischer WM, Gnanakaran S, Yoon H, Theiler J, Abfalterer W, et al. Tracking changes in SARS-CoV-2 spike: evidence that D614G increases infectivity of the COVID-19 virus. Cell 2020;182(4):812–827.
85. Plante JA, Liu Y, Liu J, Xia H, Johnson BA, Lokugamage KG, et al. Spike mutation D614G alters SARS-CoV-2 fitness. Nature 2021;592(7852):116–121.
86. Walls AC, Park YJ, Tortorici MA, Wall A, McGuire AT, Veesler D. Structure, function, and antigenicity of the SARS-CoV-2 spike glycoprotein. Cell 2020;181(2):281–292.
87. NCBI, Homo sapiens genome assembly T2T-CHM13v2.0. NCBI; 2023. https://www.ncbi.nlm.nih.gov/datasets/genome/GCF_009914755.1/, [Online; accessed 27-November-2023].
88. NCBI, Human Genome Resources at NCBI. NCBI; 2023. https://www.ncbi.nlm.nih.gov/genome/guide/human/, [Online; accessed 27-November-2023].
89. Wang Y, Mao JM, Wang GD, Luo ZP, Yang L, Yao Q, et al. Human SARS-CoV-2 has evolved to reduce CG dinucleotide in its open reading frames. Scientific Reports 2020;10(1):12331.
90. Takata MA, Gonçalves-Carneiro D, Zang TM, Soll SJ, York A, Blanco-Melo D, et al. CG dinucleotide suppression enables antiviral defence targeting non-self RNA. Nature 2017;550(7674):124–127.
91. Afrasiabi A, Alinejad-Rokny H, Khosh A, Rahnama M, Lovell N, Xu Z, et al. The low abundance of CpG in the SARS-CoV-2 genome is not an evolutionarily signature of ZAP. Scientific reports 2022;12(1):2420.
92. Chiara M, Horner DS, Ferrandi E, Gissi C, Pesole G. HaploCoV: unsupervised classification and rapid detection of novel emerging variants of SARS-CoV-2. Communications Biology 2023;6(1):443.
93. Lab A, Bjorn: Bioinformatics Pipeline for Analyzing SARS-CoV-2 Genomes;. Accessed: 2024-06-13. https://github.com/andersen-lab/bjorn.
94. Chiara M, HaploCoV: A Tool for Haplotype Analysis in SARS-CoV-2 Genomes;. Accessed: 2024-06-13. https://github.com/matteo14c/HaploCoV.
95. Crivianu-Gaita V, Thompson M. Aptamers, antibody scFv, and antibody Fab'fragments: An overview and comparison of three of the most versatile biosensor biorecognition elements. Biosensors and Bioelectronics 2016;85:32–45.
96. Keefe AD, Pai S, Ellington A. Aptamers as therapeutics. Nature reviews Drug discovery 2010;9(7):537–550.
97. Emami N, Ferdousi R. AptaNet as a deep learning approach for aptamer–protein interaction prediction. Scientific reports 2021;11(1):6074.
98. Herzig V, Cristofori-Armstrong B, Israel MR, Nixon SA, Vetter I, King GF. Animal toxins—Nature's evolutionary-refined toolkit for basic research and drug discovery. Biochemical pharmacology 2020;181:114096.
99. Pratas D, Toppinen M, Pyöriä L, Hedman K, Sajantila A, Perdomo MF. A hybrid pipeline for reconstruction and analysis of viral genomes at multi-organ level. GigaScience 2020;9(8):giaa086.
100. Adolfo LM, Rao X, Dixon RA. Identification of Pueraria spp. through DNA barcoding and comparative transcriptomics. BMC Plant Biology 2022;22(1):1–18.

Click here to access/download
Supplementary Material
AltaiR_Supplementary_Material.pdf

Click here to access/download
Supplementary Material
Comments of reviewers- AltaiR.pdf

Click here to access/download;Personal Cover;Altair_cover_letter_signed.pdf

Jorge Miguel Ferreira da Silva
IEETA, Aveiro University,Aveiro, Portugal
Jorge.miguel.ferreira.silva@ua.pt
+351 234 370 500
18/12/2023
Editor-in-Chief,
GigaScience

Dear GigaScience Editor,

I am writing to submit our manuscript titled "AltaiR: a C toolkit for alignment-free and spatial-temporal analysis of multi-FASTA data" for consideration for publication in GigaScience. This submission represents a significant contribution to the field of genomic and proteomic analysis, especially in the context of large-scale studies necessitated by recent global health challenges.

The current phase of genome sequencing, notably accelerated by the latest pandemics, has posed unique challenges in analyzing vast sets of viral genomes. Our toolkit, AltaiR, offers a novel solution to these challenges. It is specifically designed for analyzing large-scale numbers of genomes or proteomes, capable of handling millions of sequences in a single FASTA file without restrictions on size or number. This capability is crucial for contemporary genomic research and has wide-reaching implications for the scientific community.

A unique aspect of AltaiR is its alignment-free methodology, which enables the standalone identification of singularity and similarity patterns in sequences, alongside computing static and temporal dynamics. This feature not only enhances the toolkit's efficiency but also broadens its applicability across various research scenarios. In demonstrating its utility, we applied AltaiR to analyze the evolution of SARS-CoV-2 using over 1.5 million full sequences. This analysis revealed significant new insights into evolutionary patterns and characteristics of the virus, including trends in nucleotide composition and sequence complexity over time.

AltaiR's open availability, implemented in C language and optimized with multi-threading, ensures that it is readily accessible and usable by researchers worldwide. Its lack of external dependencies further adds to its practicality and ease of use. We believe that the innovative nature and the practical applications of our toolkit make it a valuable addition to GigaScience, appealing to a wide readership interested in genomic and proteomic analysis, especially in large-scale, data-intensive studies.

Thank you for considering our manuscript for publication. We are confident that our work aligns well with the scope and audience of GigaScience. Should you require any additional information or have any queries regarding our submission, please feel free to contact me.

Sincerely,

Assinado por: **JORGE MIGUEL FERREIRA DA SILVA**
Num. de Identificação: 13848089
Data: 2023.12.18 11:46:16 +0000

CHAVE MÓVEL
