# Supplementary material for: AltaiR: a C toolkit for alignment-free and temporal analysis of multi-FASTA data
Source: Gigascience. 2024 Nov 26;13:giae086. doi: 10.1093/gigascience/giae086 (PMC11590114; doi:10.1093/gigascience/giae086)
Supplement: giae086_GIGA-D-23-00393_Revision_3 [file giae086_giga-d-23-00393_revision_3.pdf]

GigaScience

AltaiR: a C toolkit for alignment-free and temporal analysis of multi-FASTA data

--Manuscript Draft--

**Manuscript Number:**	GIGA-D-23-00393R3
**Full Title:**	AltaiR: a C toolkit for alignment-free and temporal analysis of multi-FASTA data
**Article Type:**	Technical Note
**Funding Information:**	European Union's Digital Europe Programme (101081813) — PhD Jorge Miguel Ferreira da Silva<br>Fundação para a Ciência e a Tecnologia (CEECINST/00026/2018) — PhD Diogo Pratas<br>Fundação para a Ciência e a Tecnologia (UIDB/00127/2020) — Not applicable
**Abstract:**	Background: The vast number of viral genome sequences generated during the latest pandemic has presented new challenges for computational analysis. Analyzing millions of viral genomes in multi-FASTA format is computationally demanding, especially when using alignment-based methods. Most existing methods are not designed to handle such large datasets, often requiring the analysis to be divided into smaller parts to obtain results using available computational resources.<br>Findings: We introduce AltaiR, a toolkit for analyzing multiple sequences in multi-FASTA format using exclusively alignment-free methodologies. AltaiR enables the identification of singularity and similarity patterns within sequences and computes static and temporal dynamics without restrictions on the number or size of input sequences. It automatically filters low-quality, biased, or deviant data. We demonstrate AltaiR's capabilities by analyzing more than 1.5 million full SARS-CoV-2 sequences, revealing interesting observations regarding viral genome characteristics over time, such as shifts in nucleotide composition, decreases in average Kolmogorov sequence complexity, and the evolution of the smallest sequences not found in the human host.<br>Conclusions: AltaiR can identify temporal characteristics and trends in large numbers of sequences, making it ideal for scenarios involving endemic or epidemic outbreaks with vast amounts of available sequence data. Implemented in C with multi-threading and methodological optimizations, AltaiR is computationally efficient, flexible, and dependency-free. It accepts any sequence in FASTA format, including amino acid sequences. The complete toolkit is freely available at https://github.com/cobilab/altair.
**Corresponding Author:**	Jorge Miguel Ferreira da Silva<br>Universidade de Aveiro Instituto de Engenharia Eletrónica e Informática de Aveiro<br>Esmoriz, Seleccione um PORTUGAL
**Corresponding Author Secondary Information:**	
**Corresponding Author's Institution:**	Universidade de Aveiro Instituto de Engenharia Eletrónica e Informática de Aveiro
**Corresponding Author's Secondary Institution:**	
**First Author:**	Jorge Miguel Ferreira da Silva, PhD
**First Author Secondary Information:**	
**Order of Authors:**	Jorge Miguel Ferreira da Silva, PhD
	Armando J Pinho, PhD
	Diogo Pratas, PhD
**Order of Authors Secondary Information:**	
**Response to Reviewers:**	Dear Dr. Zauner,<br><br>Thank you for your email and for accepting our manuscript. We appreciate the valuable feedback and are pleased to provide the necessary revisions as per your editorial requests.

Powered by Editorial Manager® and ProduXion Manager® from Aries Systems Corporation

Additional Information:	
Question	Response
Are you submitting this manuscript to a special series or article collection?	No
**Experimental design and statistics**<br><br>Full details of the experimental design and statistical methods used should be given in the Methods section, as detailed in our Minimum Standards Reporting Checklist. Information essential to interpreting the data presented should be made available in the figure legends.<br><br>Have you included all the information requested in your manuscript?	Yes
**Resources**<br><br>A description of all resources used, including antibodies, cell lines, animals and software tools, with enough information to allow them to be uniquely identified, should be included in the Methods section. Authors are strongly encouraged to cite Research Resource Identifiers (RRIDs) for antibodies, model organisms and tools, where possible.<br><br>Have you included the information requested as detailed in our Minimum Standards Reporting Checklist?	Yes
**Availability of data and materials**<br><br>All datasets and code on which the conclusions of the paper rely must be either included in your submission or deposited in publicly available repositories (where available and ethically appropriate), referencing such data using a unique identifier in the references and in the "Availability of Data and Materials" section of your manuscript.	Yes

Have you have met the above requirement as detailed in our Minimum Standards Reporting Checklist?	

Powered by Editorial Manager® and ProduXion Manager® from Aries Systems Corporation

LaTeX - Main Document (TeX file) Click here to access/download;LaTeX - Main Document (TeX file);main.tex

OXFORD
(GIGA)^n SCIENCE

GigaScience, 2023, 1–0

doi: xx.xxxx/xxxx
Manuscript in Preparation
Technical Note

TECHNICAL NOTE

AltaiR: a C toolkit for alignment-free and temporal analysis of multi-FASTA data

Jorge M. Silva[1,2,*], Armando J. Pinho[1,2] and Diogo Pratas[1,2,3,*]

[1]IEETA/LASI, Institute of Electronics and Informatics Engineering of Aveiro, University of Aveiro, Portugal and [2]DETI, Department of Electronics, Telecommunications and Informatics, University of Aveiro, Portugal and [3]DoV, Department of Virology, University of Helsinki, Finland

[*]Correspondence address. Jorge M. Silva and Diogo Pratas. E-mail: jorge.miguel.ferreira.silva@ua.pt and pratas@ua.pt

Abstract

Background: The vast number of viral genome sequences generated during the latest pandemic has presented new challenges for computational analysis. Analyzing millions of viral genomes in multi-FASTA format is computationally demanding, especially when using alignment-based methods. Most existing methods are not designed to handle such large datasets, often requiring the analysis to be divided into smaller parts to obtain results using available computational resources.
Findings: We introduce AltaiR, a toolkit for analyzing multiple sequences in multi-FASTA format using exclusively alignment-free methodologies. AltaiR enables the identification of singularity and similarity patterns within sequences and computes static and temporal dynamics without restrictions on the number or size of input sequences. It automatically filters low-quality, biased, or deviant data. We demonstrate AltaiR's capabilities by analyzing more than 1.5 million full SARS-CoV-2 sequences, revealing interesting observations regarding viral genome characteristics over time, such as shifts in nucleotide composition, decreases in average Kolmogorov sequence complexity, and the evolution of the smallest sequences not found in the human host.
Conclusions: AltaiR can identify temporal characteristics and trends in large numbers of sequences, making it ideal for scenarios involving endemic or epidemic outbreaks with vast amounts of available sequence data. Implemented in C with multi-threading and methodological optimizations, AltaiR is computationally efficient, flexible, and dependency-free. It accepts any sequence in FASTA format, including amino acid sequences. The complete toolkit is freely available at `https://github.com/cobilab/altair`.

Key words: Alignment-free toolkit; Relative Absent Words; Data compression; Temporal patterns; Viral genomes; multi-FASTA;

Introduction

The recent availability of millions of SARS-CoV-2 complete genomes that emerged from the COVID-19 pandemic has remarkably changed the scientific workflow. It starts with the parallelization of the genome's sequencing and assembly through many scientific and industrial entities, followed by the centralized upload of each SARS-CoV-2 assembled sequence in FASTA format and the respective metadata, while respecting a light data curation, mainly in the following database repositories: GISAID [1], NCBI [2], and INSDC [3], that include GeneBank [4], ENA [5], and DDBJ [6].

Although the incredible growing pace at which these complete genomes emerged and have been made publicly available, their downstream analysis faces new challenges mainly related to the vast number of genomes, characteristics and format of the data, and multiplexed sequencing-reconstruction.

Specifically, one challenge emerged due to the availability of millions of genomes split by the respective headers with basic information in a single file. This file format is known as Multi-FASTA, but it is generically called FASTA. There are alternative and more efficient file format representations, such as those representing only differences according to a reference. Still, the majority of the downstream analysis tools require the FASTA format. Moreover, storing only the differences would require multiple references to perform additional analyses at different taxonomic levels. The alternative way of using a single reference sequence becomes inefficient, if there is high dissimilarity.

Furthermore, for processing up to a hundred thousand genomes,

Compiled on: October 11, 2024.
Draft manuscript prepared by the author.

only a minority of the tools are prepared, while the availability of these tools is very scarce to process millions. The main reason is these programs' use of temporary files to decrease the computational memory and related computational time. However, creating temporary files times millions of entries is not efficiently affordable.

Another challenge is the existence of outlier or recombinant genomes in the set. The outlier entries are unwanted sequences uploaded by entities that show profound differences in sequencing and assembly methodologies. Examples of these features are the presence/absence of targeted capture, the assembly methodology using appropriate/inappropriate references, proper/improper exclusion of contaminants, accurate/inaccurate variant call, accurate/inaccurate base masking, or substantial differences in SNPs caused by postmortem degradation. Although several directives have been provided [7, 8], these genomes still exist and are continuously being uploaded. Recombinant genomes usually show a higher degree of variants or chimeric formations after cell co-infection [9]. Due to these temporary increases in the accumulation of genomic variations, recombinant genomes can be associated with the emergence of outbreak strains, as exemplified by coronaviruses and rhinoviruses [10, 11]. For downstream analysis, the objective is to discard outliers while maintaining the recombinant genomes. However, we currently lack fast genome filtering tools that assert a particular trait distribution to minimize outliers in the data while maintaining the average genome data of these recombinant genomes.

Beyond the SARS-CoV-2 sequences, the upload of large quantities of viral genomes in FASTA format is also substantially increasing. Because viral genomes are sometimes directly associated with hosting health conditions, namely in cancer or autoimmune diseases, the number of these reconstructed genomes is expected to increase dramatically for clinical and forensic purposes [12, 13, 14]. Some examples are the steadily increasing availability of Parvoviruses, Polyomaviruses, Herpesviruses, and Papillomaviruses [15, 16, 17]. Moreover, this increase is also in other types of organisms, such as organisms with larger genome sequences, namely fungi and bacteria [18], especially now with the higher rates of antibiotic resistance and the availability of complete genome sequences [19, 20] associated with the development of the Telomere-2-Telomere (T2T) technology [21, 22].

The current disposal of the massive number of genomes from a single species also provides the opportunity to study a species sequence over time, namely the temporal patterns and characteristics of the genomes or proteomes in a temporal dimension. Accordingly, the following questions emerge:

- How does the nucleotide composition of the genomes change over time?
- How does the sequence entropy of a species change in time?
- How does the similarity of parasitic sequence species vary according to the first known sequence?
- What are the shortest sub-sequences of a parasitic species not in a host?
- How do these shortest sequences change in time?

These complex questions can be answered with this type of data in synergy with the alignment-free method that is provided in this manuscript.

Although alignment methods offer an intuitive and enhanced local resolution that prevails in comparative analysis of specific features in a low number of sequences, large-scale sequence quantities require unfeasible computational resources under a desirable accuracy, limiting their applicability in multiple temporal analyses. On the other hand, the substantial increase in the development and availability of alignment-free methods [23, 24, 25, 26, 27] have provided clear advantages using feasible computational resources.

In the literature, there are multiple toolkits for specific sequence transformation and analyses applications, namely microbiome tools for forensic science [28], for machine-learning analysis and modelling of genomic and proteomic sequence data [29], for visualization of regulatory DNA motif identification and analyses [30], efficient analysis of DNA methylation [31], among many others. On the other hand, there are toolkits or platforms with a much broader application, namely SeqAn [32], khmer [33, 34], GTO [35], GATB [36], Mutalisk [37], CGAT [38], CGtag [39], nanoGalaxy [40], AlcoR [41], Poretools [42], Pycogent [43], SeqKit [44], FASTAptamer [45], fairseq [46], SeqKit [44], TBtools [47], MPI bioinformatics toolkit [48], KBase [49], among many others. Some of these toolkits or platforms are interactive through web browsers offering friendly environments, while others are prone to fast and efficient computation through the command line.

Benchmarking genomic toolkits by direct comparison can be misleading due to their distinctive design philosophies and unique feature sets. Each toolkit is crafted with specific goals in mind, which may not align perfectly with those of another. Hence, simple side-by-side comparisons might not only be uninformative but could obscure the individual strengths of each tool. Instead, a more nuanced approach involves evaluating a toolkit based on its specific features and objectives. Important aspects to consider include the toolkit's innovative features, the research questions it facilitates, its efficiency and ease of use, and any software dependencies it might have. These criteria form the core of high-level benchmarking, focusing on what each toolkit brings to the scientific community rather than how it matches up against others.

On a deeper level, benchmarking should employ both synthetic data, which provides a controlled environment to assess tool performance predictably, and natural data, which offers real-world challenges. Synthetic data testing is essential and should be a standard preliminary test, whereas natural data testing, although occasionally challenging due to the variability of biological data, is crucial for understanding how the tool performs under realistic conditions. Additionally, the reproducibility and, when feasible, the repeatability of results are paramount to confirm the reliability and effectiveness of each tool. This rigorous approach ensures that the tools not only meet theoretical expectations but also hold up under practical application.

In this article, we present AltaiR, a toolkit with alignment-free methods for the temporal analysis of multi-FASTA data, specifically large-scale numbers of genomes or proteomes, including millions (or billions) of sequences in a single FASTA file. The AltaiR toolkit offers an efficient and convenient approach for pathogen-host analyses in endemic or pandemic scenarios (but not limited to). Importantly, the AltaiR method includes a flexible tool to filter sequences undesired from the dataset through parameterized characteristics. AltaiR contains both reference-free and reference-based tools. The reference-free tools are for analysing sequence entropy and nucleotide frequency changes over time. The reference-based tools are divided into two main wings, similarity and singularity. For both, highly efficient implementations are provided. The AltaiR is developed in C language and is provided as open-source software. In the next section, we enumerate the features and characteristics of the AltaiR toolkit, including the description and formalization of the methods. Then, we benchmark each tool from the toolkit using synthetic data while ensuring the full repeatability of the experiment. Afterwards, we present results using natural data, namely an application for a vast SARS-CoV-2 set of genomes. Finally, we discuss the results obtained and draw some conclusions.

Methods

This section describes the methods and their respective implementation into computer tools that constitute the AltaiR toolkit. The details of the parameters and how to reproduce the methods are available in Supplementary Sections 2 and 3. We recommend using compressed data, namely through the compression of the files us-

ing specialized tools. For fast access, NAF [50], AGC [51], and, for bacteria, MBGC [52] can be used. For a higher compression ratio but slower access, we recommend MFCompress [53]. When using sensitive data, Cryfa [54] can be used to compact and encrypt the data.

Figure 1 describes the workflow of AltaiR by dividing the data according to three types: the parasite reference sequences, the parasite sequences (after filtering the outliers or unwanted data), and the host omics sequences. The workflow contains seven main methods.

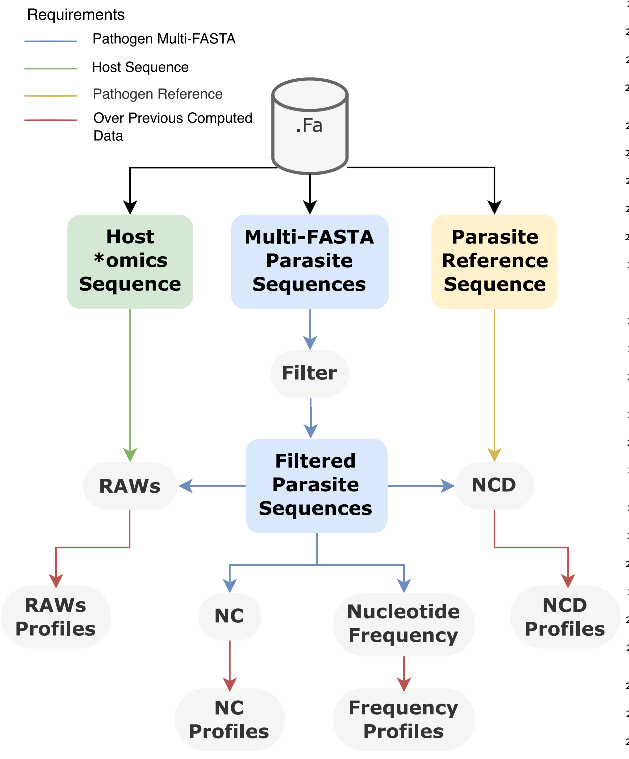

Figure 1. Workflow of the AltaiR toolkit describing the principal seven phases along with the three input types of sequences in FASTA format.

The first method is filtering the sequences by characteristics, essentially removing sequences that contain different traits from what is expected or the average. The second method is the Normalized Compression Distance (NCD) for measuring the similarity between two sequences. The NCD extension is provided in the third method through the use of the NCD profiles. These are generated by the similarity comparison of the existent sequences according to a reference. The fourth method is the Normalized Compression (NC) profiles that depict the compression variability of the sequences. The fifth method is the frequency profiles, allowing to visualize changes in the nucleotide composition over time. The sixth method is the RAWs (Relative Absent Words) mapping that identifies the shortest sequences in the parasite but absent from the host genome/transcriptome. Finally, the seventh method provides profiles regarding the RAWs according to time. It is important to note that for temporal analyses, such as the NC, NCD profiles and frequency profiles, the input sequences must be sorted by date. This sorting is crucial for accurately capturing trends and patterns over time.

The further subsections provide details on the methods, including their mathematical formalizations and characteristics.

Filter sequences by characteristics

The public databases of multiple genome and proteome sequences from a specific organism usually contain sequences not within the average group. The most frequent reasons are the inclusion of contaminant sequences [55, 56] and differences in the sequencing or assembly procedures. Removing some of these sequences from the dataset is important for downstream analyses. However, manual inspection is slow and can introduce errors. Therefore, automatically filtering these sequences by characteristics is important and can provide unbiased selections while maintaining visible static and uniform characteristics. AltaiR incorporates established guidelines and best practices [7, 8] to address potential contamination and quality issues through automated filtering. This process ensures only high-quality, contiguous sequences are included in the analysis, minimizing bias from incomplete or contaminated data.

Accordingly, this sub-method filters a multi-FASTA file, specifically FASTA reads, by input characteristics. These characteristics can be the alphabet composition, sequence completeness or length, GC quantity, multiple string header patterns, or absence of patterns (anti-patterns). Completeness refers to the availability of the full, uninterrupted sequence of an organism's genome, rather than just partial or fragmented sequences. The computation can be applied to different sequences; for example, a specific filtering can be applied to SARS-CoV-2 while another for B19V. Moreover, the filtering characteristics can be intersected through a single run.

Consider a source that has generated n sequences from a finite alphabet Ξ with size $|\Xi|$. The nature of the source is unknown but in each sequence x^i, where $x^0, ..., x^n$, the internal symbols are known.

The filtering aims to evaluate specific characteristics that occur in each x^i, and those which respect the conditions are used for further downstream analyses. Moreover, each x^i contains a header alphanumeric sequence, y^i, that generically identifies x_i. Specifically, the y^i is the metadata of x^i and usually contains the name of the organism, the date, and a unique ID, among others.

The following definitions provide information on the filtering for each main characteristic or feature, including the sequence length, alphabet composition, minimum and maximum GC percentage, header patterns and anti-patterns.

Sequence length

In multiple cases, sequences labelled as complete are nearly complete, or the length is shorter than a certain length. This can occur, for instance, due to the sequencing depth being very low or some scaffolds could not be linked in the assembly process. AltaiR can filter sequences by the minimal and maximal length to minimise these issues. Therefore, for any string x^i with length $|x^i|$ the set of final sequences, Σ, is composed by

$$x^i \in \Sigma \iff \mathcal{N}_{\max} \geq \mathcal{N}(x^i) \geq \mathcal{N}_{\min}, \quad (1)$$

where $\mathcal{N}$ is the length of the sequence x_i and $\mathcal{N}_{\min}$ and $\mathcal{N}_{\max}$ the minimum and maximum length, respectively.

Alphabet composition

It is common to find some reads of a multi-FASTA format containing characters outside a desired set. For example, the 'R' symbol at the DNA sequence level stands for a purine (an A or G base). This characteristic is frequent with other symbols in protein sequences, leading to an unbalance between the set cardinality of the sequences. This ambiguity generates complexity in the analysis when using many software tools and can create deviant results. Therefore, the AltaiR method efficiently filters sequence symbols containing a certain alphabet. Additionally, it can filter the reads by composition, including completeness, when combined with the length.

Minimum and maximum GC percentage

The GC percentage is usually an important feature to evaluate if the distribution of a particular sequence is within a desirable average set. Specifically, the GC percentage is given by the number of cytosine (C) and guanine (G) bases in a string x^i with length $|x^i|$ according to

$$\mathcal{GC}(x^i) = \frac{100}{|x^i|} \sum_{j=1}^{|x^i|} \mathcal{I}_{\Xi}(x^i_j), \quad (2)$$

where x^i_j is each symbol of x^i (assuming causal order), Ξ is a subset alphabet containing the symbols $\{G, C\}$ and $\mathcal{I}$ an indicator function constrained to

$$\mathcal{I}_{\Xi}(x) = \begin{cases} 1, x \in \Xi \\ 0, x \notin \Xi \end{cases}. \quad (3)$$

Then, the set of final sequences, Σ, is composed by

$$x^i \in \Sigma \iff \mathcal{GC}_{\max} \geq \mathcal{GC}(x^i) \geq \mathcal{GC}_{\min}, \quad (4)$$

where $\mathcal{GC}_{\min}$ and $\mathcal{GC}_{\max}$ are two real values where $\mathcal{GC}_{\max} > \mathcal{GC}_{\min} \in [0;100]$.

Header patterns and anti-patterns

The header patterns are strings to match in each sequence header, h^i, that can ignore or consider a certain sequence read if the substring pattern is absent or present. This is the analogous process of the well-known grep tool, but instead of looking into the whole FASTA file, it only filters by the header sequence. This type of filtering is very important when only a certain type of sequence is to be considered or sequences with a certain header substring must not pass to the final set of sequences, Σ.

The AltaiR toolkit provides filtering of the header patterns using substrings that occur in the header or that are absent (ignore pattern) using conjoint filtering that is not limited to the number of strings to match or ignore. For example, using the patterns "Human", "Herpes" and "Alpha" and the anti-pattern "Simplex" in the whole viral reference database from the NCBI will select all the FASTA reads that the headers contain the words Human, Herpes, and Alpha, selecting only the human Alphaherpesvirus that are composed by the HSV-1 (Herpes Simplex Virus 1), HSV-2 (Herpes Simplex Virus 2), and VZV (Varicella-Zoster Virus); because the anti-pattern "Simplex" was also used, then, only the VZV will be contained in Σ.

Similarity (NCD) profiles

In temporal analysis, some applications require measuring the similarity of the most recent genomes to the first ones being sequenced and assembled, such as the first reference genome. These measures will allow us to understand the evolution rate over time and if evolutionary acceleration peaks have been found in specific periods. Accordingly, if the genomes are sorted according to sample isolation date (an NCBI VSSI option while downloading the data), then measuring the distance of the first genome according to the remaining in the multi-FASTA file provides this similarity information.

To compute the similarity between the reference genome and the remaining sequences, the Normalized Compression Distance (NCD), which is a similarity distance that approximates the Kolmogorov complexity through data compression, is used [57, 58, 59]. The NCD has many applications for genomic and proteomic assembled sequences [60], including the similarity measure for the COVID-19 pandemics [61, 62].

Formally, the NCD between a reference y and a target x sequence is given by

$$\mathcal{D}(x, y) = \frac{C(x, y) - \min\{C(x), C(y)\}}{\max\{C(x), C(y)\}}, \quad (5)$$

where $C(x)$ and $C(y)$ represents the number of bits needed to lossless compress x and y, respectively. The $C(x, y)$ represents the number of bits needed to compress x and y conjointly, which usually is approximated by string concatenation.

Since in our application, we are required to compute the whole distances of each x^i according to a reference y, then an array of distances is calculated for creating the NCD profile according to

$$\mathcal{D}_i(x^i, y) = \frac{C(x^i, y) - \min\{C(x^i), C(y)\}}{\max\{C(x^i), C(y)\}}. \quad (6)$$

Moreover, because the conjoint compression follows the commutative property $C(x^i, y) = C(y, x^i)$, it can be rewritten as

$$\mathcal{D}_i(x^i, y) = \frac{C(y, x^i) - \min\{C(x^i), C(y)\}}{\max\{C(x^i), C(y)\}}. \quad (7)$$

This change offers a substantial save in computational resources because it is now possible to compress y and, in the end, freeze its models. Then, the number of bits to compress y is saved, and for each x^i, the compression models are initialized with the frozen models of y. Therefore the complexity time to compute $C(y, x^1), ..., C(y, x^n)$ is now $y + xn$. This change is now affordable for an application considering millions of genomes.

The choice of the data compressor is fundamental to better approximating the Kolmogorov complexity and the NCD [63, 58]. Therefore, besides respecting the common distance characteristics [63] and distance density [58], the data compressors that are designed for the specific use of certain types of data offer a much higher approximation of the Kolmogorov complexity than general-purpose tools. This characteristic is provided by the specific-purpose methodology's ability to efficiently model characteristics that would otherwise not be seen with general-purpose models. For example, two characteristics that play a key role in biological sequences are inverted repeats [64] and high-level substitutions in repetitive data [65].

In the case of the COVID-19 pandemic, a specific-purpose data compressor does not offer a wide advantage over a general-purpose data compressor because, on average, the sequences contain high entropy, are small, and have only a few sequence differences between sequences. However, an efficient specific-data compressor is mandatory for larger genomes such as those from larger viruses (e.g. Herpesvirus), bacterial or fungi. Therefore, we use an implementation derived from GeCo3 [66] and AC2 [62] data compressors that have proven to be state-of-the-art data compressors in genomic and proteomic sequences, respectively. The disadvantage of these data compressors is the necessity to have a solid knowledge of the models for extensive optimization. We provide pre-computed models for different biological sequence types to minimise this disadvantage.

For applications where the size of the distances vector is very large or the variation of the instances in the profile high, AltaiR offers the possibility of averaging the signal through a moving average. The Toolkit implementation subsection provides more information about this option.

Complexity (NC) Profiles

Another important temporal analysis is the perception of how the (normalized) Kolmogorov complexity [59] of a certain set of sequences sorted by time varies. The normalized Kolmogorov complexity is approximated through the Normalized Compression

(NC) [67]. The NC is given by the ratio of the uncompressed quantity sum of bits by the size of the sequence representation assuming a uniform distribution. This analysis is analogous to understanding how the genome or proteome sequence entropy varies over time using a random reference.

As in the previous Subsection, the Kolmogorov complexity is approximated using specific-purpose data compression algorithms, namely with implementations derived from GeCo3 [66] and AC2 [62] for genome and proteome sequences, respectively.

Formally, the NC of a certain sequence is provided by

$$\mathcal{E}(x) = \frac{C(x)}{|x| \log_2(|\Xi|)}, \quad (8)$$

where $|x|$ is the size of the sequence and $|\Xi|$ the number of different symbols in the sequence x.

Since in our application, we require to compute the whole sequence complexity of each x^i, then an array of NCs is calculated for creating the NC profile according to

$$\mathcal{E}_i(x^i) = \frac{C(x^i)}{|x^i| \log_2(|\Xi^i|)}, \quad (9)$$

where $|\Xi^i|$ is the number of different symbols in the sequence x^i.

For genomic sequences, Ξ is 4 for any x^i. However, for proteomic sequences, Ξ may vary, creating changes in the profile. For these cases, alphabet trimming, which involves reducing the alphabet size by removing or substituting non-standard characters, can work as a solution to ensure a consistent alphabet size across all sequences and maintain the comparability of NC values.

For applications where the size of the NC vector is very large or the variation of the instances in the profile high, AltaiR offers the possibility of averaging the signal through a moving average. The Toolkit implementation subsection provides more information about this option.

Frequency profiles

One application that provides evidence of evolutionary events is the variation of the nucleotide or amino acid frequency over time. This information is given by the frequency profiles, defined as the percentage symbol distribution of each sequence.

Formally, the frequency of a certain symbol, s, from Σ in a sequence x is provided by

$$\mathcal{F}(s|x) = \frac{1}{|x|} \sum_{j=1}^{|x|} \mathcal{I}_s(x_j), \quad (10)$$

where $\mathcal{I}$ is an indicator function associated to the number of times that s is seen in x.

Since in our application, we require to compute the symbol frequency of each x^i, then an array of frequencies is calculated for creating the frequency profile according to

$$\mathcal{F}_i(s|x^i) = \frac{1}{|x^i|} \sum_{j=1}^{|x^i|} \mathcal{I}_s(x^i_j). \quad (11)$$

For applications where the size of the frequency vector is very large or the variation of the instances in the profile high, AltaiR offers the possibility of averaging the signal through a moving average. The Toolkit implementation subsection provides more information about this option.

Relative singularity (RAWs) profiles

Identifying the shortest sequence regions present in a set of pathogens but absent from the host genome and transcriptome is an important application in endemic and epidemic contexts. These regions, called Relative Absent Words (RAWs), are of interest because they can distinguish between lineages [68], localize higher GC-content regions, and have applications in optimized diagnosis [69]. Furthermore, RAWs can provide insights into the unique genomic characteristics of pathogens, potentially aiding in the development of targeted therapeutic interventions and diagnostic tools [69].

Specifically, RAWs [68, 69, 70] are a particular subset of Minimal Absent Words (MAWs) that have also been referred to as nullomers or forbidden words [71, 72, 73]. Many MAWs-based applications have been created, including optimized models for their detection [74] and the complementary simulation of sequences while avoiding the creation of MAWs [75].

MAWs in genomic and proteomic sequences have been studied [76]. For example, in viruses, MAWs have been found most frequently in regions that are restriction recognition sites [76]. On the other hand, the subset of MAWs (RAWs) has been found in regions with high GC content, contrary to the host and pathogen genome [69].

An approach to finding MAWs is through the flanking subsequence regions. Consider a set, X, constituted of n target sequences, $x^1, x^{\cdots}, x^n$, and a reference sequence, y, both drawn from the finite alphabet Σ. We say that β is a factor of x^i if x^i can be expressed as $x^i = u\beta v$, with uv denoting the concatenation between sequences u and v. We denote by $\mathcal{W}_k(x^i)$ the set of all k-size words (or factors) of x^i. Also, we represent the set of all k-size words *not in* x^i as $\overline{\mathcal{W}_k(x^i)}$. For each word size k, we define the set of all words that exist in x^i but do not exist in y by

$$\mathcal{R}_k(x^i, \overline{y}) = \mathcal{W}_k(x^i) \cap \overline{\mathcal{W}_k(y)}. \quad (12)$$

The subset of minimal words as

$$\mathcal{M}_k(x^i, \overline{y}) = \{\beta \in \mathcal{R}_k(x^i, \overline{y}) : \mathcal{W}_{k-1}(\beta) \cap \mathcal{M}_{k-1}(x^i, \overline{y}) = \emptyset\}, \quad (13)$$

i.e., a MAW of size k cannot contain any MAW of size less than k. In particular, $l\beta r$ is a MAW of sequence x^i, where l and r are single letters from Σ, if $l\beta r$ is not a word of x^i but both $l\beta$ and βr are. We have defined the non-empty set $\mathcal{M}_k(x^i, \overline{y})$ with the smallest k as minimal Relative Absent Words (mRAWs) [68].

Another subset of MAWs and RAWs are persistent mRAWs (PmRAWs) [69]. Formally, let r be a mRAW of $x^i \in X$ and $P(r, x^i)$ be the predicate "r is a RAW of string x^i". Then, if $\forall_{x^i \in X} P(r, x^i)$, we say that r is persistent in X. This property means the full conservation of the identified mRAWs across all the sequences. A particularity of these sequences is to consider PmRAWs at the whole genome level and at a sub-genome level, meaning that a x^i can be considered a subsequence of a whole genome, namely a gene, extending the power of PmRAWs to local observations.

The RAWs and PmRAWs have been computed with the EAGLE tools version 1 [68] and 2 for DNA sequences [69]. This methodology was included in the online detection of RAWs, namely the ADACT [77].

The AltaiR toolkit can compute the RAWs based on the same methodology as in EAGLE version 2. Still, it extends the capability to deal with any other types of sequences, for example, proteomes, as long as they respect the multi-FASTA format. Moreover, for computing the RAWs, the AltaiR toolkit can avoid writing into temporary files while maintaining low memory consumption. This capability enables the computation of RAWs in millions of genomes/proteomes while expending relatively low computational resources.

Moreover, the Altair toolkit enables the automatic computation

of the GC percentage for each RAW, providing the unprecedented ability to create large-scale studies with a higher divergence between the sequences.

Additionally, the AltaiR toolkit includes the capability to compute RAWs profiles. The RAWs profiles are generated with the additional combination of temporal metadata, namely through the time order usage of the target multi-FASTA file that can be downloaded with that property at the NCBI repository. These profiles directly compute the presence of each RAW according to the sequences sorted by temporal characterization.

Toolkit implementation

The AltaiR methodology is implemented in C language and contains no external dependencies. The source code and application result scripts are freely provided at the repository [78]. The AltaiR toolkit contains one main menu (command: AltaiR) with six sub-menus for computing the methods that it provides, sometimes through the combination of multiple, namely

- **average** - moving average filter of a column float CSV file (the column to use is a parameter);
- **filter** - filters FASTA reads by characteristics: alphabet, completeness, length, GC quantity, multiple string patterns and anti-patterns;
- **frequency** - computes the alphabet frequencies for each FASTA read (it enables alphabet filtering);
- **nc** - computes the Normalized Compression (NC) for all FASTA reads according to a compression level or specific parameters;
- **ncd** - computes the Normalized Compression Distance (NCD) for all FASTA reads according to a reference;
- **raw** - Computes Relative Absent Words (RAWs) with automatic GC percentage estimation for all RAWs.

The toolkit allows reading and writing from standard input and output, respectively, in certain cases. For example, this feature enables the direct piping of the output from the filtering tool to the NC, NCD, and RAWs tools, streamlining the analysis process. Additionally, it allows for the output profiles of the frequency, NC, NCD, and RAWs tools to be easily processed by other tools, such as those performing averaging or visualization. This functionality provides easier integration to build custom pipelines. Furthermore, most tools (except average) use multi-threading to speed up the analysis.

In the next section (results), we provide details of the toolkit features, including the most important parameters and commands to retrieve the results. Nevertheless, more documentation can be retrieved from the Supplementary material and in the README.md file in the code repository.

Results

As a result, we provide the application analysis of the AltaiR toolkit to the challenges described in the previous section. The primary motivation for using the SARS-CoV-2 data is to demonstrate AltaiR's capabilities in handling large-scale genomic datasets and uncover novel insights into the virus's evolution, adaptation, and interaction with the human host. All the results can be repeated using the procedures described in each specific analysis, except for the data collection retrieved manually from the NCBI Virus database. Despite this manual procedure, all the data is included as supplementary material for direct download. The dataset used to compute the results includes a large collection of SARS-CoV-2 genome sequences retrieved from the NCBI viral repository on 29 September 2022 [2]. The SARS-CoV-2 genomes have been filtered from a pool of 6,309,078 genomes according to quality and completeness, namely only considering complete genomes with the host as human, resulting in 1,538,095 genomes [79]. The dataset also contains multiple coronavirus genomes, the reference T2T human genome and transcriptome, and multiple computer-generated sequences; the latter works as an important validation procedure.

Filtering sequences

The SARS-CoV-2 dataset contains nearly 1.5 million sequences labelled as complete genomes in the NCBI viral repository. In the histogram depicted in Figure 2, we notice substantial differences in the length of the sequences. This histogram provides the frequency of the length of the sequences, which was computed considering symbols outside the alphabet $\Sigma = \{A, C, G, T\}$, such as the N symbols and others. Although the plot considers only the sequence length from 29.5k and 30k, there were several sequences with lengths lower than 20k. These smaller sequences were considered complete genomes and showcased the importance of filtering the data by the sequence length.

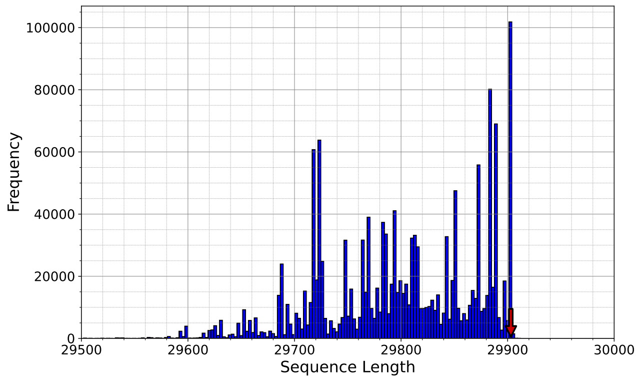

Figure 2. Histogram with the length of the genomes, considering all the existent symbols, from the SARS-CoV-2 dataset. The red arrow line stands for the size of the reference genome. To replicate this analysis, see Supplementary Section Reproducibility, specifically Reproducing the "Data filtering".

The SARS-CoV-2 reference genome (NC_045512.2) has a length of 29,903, and the largest cluster of sequences is almost coincident with this length (around 100k sequences). Therefore, since the SARS-CoV-2 genomes have low variability, this region is a good candidate for sequence length filtering.

However, the SARS-CoV-2 genomes contain a poly(A)-tale with a variable size in the end-tip. This poly(A)-tale is the only low-complexity region and, therefore, it is, ironically, the region more complex to sequence and assemble [69, 41]. To provide some flexibility to this region, we filtered the sequences considering the interval length of 29,885 to 29,921. Notice that many of these sequences contained symbols outside the $\{A, C, G, T\}$ alphabet, for example, N symbols, and, thus, many of them were removed when the total of alphabet symbols was not reached.

Moreover, we included filtering specific patterns and anti-patterns, namely considering only the sequences where the headers did not contain only the year (e.g. "|2020|") but also the month and, if available, the day associated with the virus isolation. Also, sequences without dates were discarded.

In sum, the sequence length and the pattern/anti-pattern decreased by 1.5 million to 25,594 sequences. To assess the impact of our filtering criteria on the representation of viral variants, we compared the distribution of the top 10 most represented variants before and after filtering. Our analysis shows that while the filtering process does reduce the total number of sequences from 1.5 million to 25,594, it maintains a consistent representation of the

major viral variants. The Pearson correlation (0.7348), Spearman correlation (0.7982), and cosine similarity (0.7940) between the before and after distributions indicate a strong positive correlation and overall similarity in variant representation. Figure 3 illustrates the variant distribution before and after filtering, demonstrating that the most prevalent variants remain well-represented in the filtered dataset. Although the dataset was reduced substantially, the data quality substantially increased proportionally. Moreover, nearly 25k of high-quality sequences are sufficient for accurate downstream analysis.

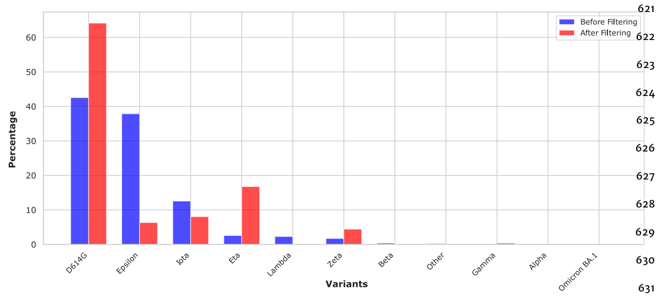

Figure 3. Variant representation before and after filtering.

This sequence filtering step shows its critical importance, proven by the high variability between these sequences, which can harm downstream analyses without proper filtering. For instance, including incomplete, low-quality, or outlier sequences can introduce noise and bias in similarity measurements, leading to inaccurate clustering or phylogenetic inference. Inconsistent or missing metadata can hinder the interpretation of temporal patterns and evolutionary trends, while unusual sequence characteristics can skew statistical analyses and obscure biological patterns. As such, rigorous filtering ensures high data quality and consistency, minimizing spurious results and enabling reliable biological interpretations.

Similarity (NCD) profiles application

To benchmark the similarity profiles described in the Subsection Similarity (NCD) profiles, we recurred to two levels, namely, using synthetic and real data.

The synthetic data (denoted as the original sequence) has been computer-generated with the GTO toolkit [35] while assuming a uniform distribution and a length of 5000 DNA symbols. Using this original sequence, consecutive SNP mutations have been applied to the following sequences with a symbol mutation probability of 0.00005. To clarify, the original sequence (time-0) has been mutated while originating the sequence time-1, then the sequence time-1 has been mutated originating the sequence time-2, and this process was followed to the sequence time-10000. Finally, the temporal similarity profiles have been computed using a chosen time point as a reference. Supplementary Figure 1 provides the similarity (NCD) profiles for three time points, namely 0, 2500, and 4000.As depicted, the NCD profiles have a minimum value for the respective time point reference, showing the capability to identify the closest sequence in time under these simplified conditions.

Despite the performance of the previous approach, it is hard to find a real scenario where a time point sequence is perfectly available. In practice, this sequence already contains several mutations. Therefore, to simulate this characteristic, we repeat the above experience, but instead of using a direct time point sequence, we use a mutated time point sequence.

Accordingly, all the time point symbol sequences have been mutated with a probability of 0.05 (mutations in approximately 5% of the sequence) using a uniform distribution and different seeds. Supplementary Figure S2 provides the similarity (NCD) profiles for three-time points mutated, namely 0, 2500, and 4000. Although the average NCD value increased, the similarity is still comparatively high for the selected points. These results suggest that NCD profiles may serve as tolerant mutation measures to predict the temporal occurrence of sequences, but further investigation is required to validate this claim.

In addition to the synthetic data analysis, we also explored the application of NCD profiles to a real-world dataset, specifically focusing on the RaTG13 genome sequence and its similarity to SARS-CoV-2 sequences. This analysis, along with a discussion of its limitations and potential implications, can be found in the Supplementary Material. Applying NCD profiles to this real-world dataset demonstrates their ability to uncover interesting trends and relationships that can guide further research. While the results obtained from NCD profiles should be interpreted cautiously and validated through additional lines of evidence, they serve as a valuable starting point for more focused investigations and hypothesis generation.

It's also worth mentioning that from any NCD results, one can automatically construct a phylogenetic tree for the n closest sequences. The Supplementary Material under the Reproducibility Section illustrates an example of this tree, along with instructions for recreating it.

Complexity profiles (NC) application

Herein, we present a detailed complexity profile (NC) analysis of SARS-CoV-2 sequences using the AltaiR toolkit. By approximating the normalized Kolmogorov complexity through the Normalized Compression metric, we quantitatively assessed the informational content of the viral sequences over time.

The complexity profile, illustrated in Figure 4, captures the temporal fluctuations in the sequence complexity of the virus. The NC values were computed using 5, 20, and 100 window sizes to smooth the data and reveal underlying trends. The plot shows the NC trajectory, where each line represents the complexity calculated over a different window size.

The time frame from January 2020 to January 2022 was particularly interesting due to significant epidemiological events. Our analysis encapsulates this period, showing a dynamic yet subtle evolution of the virus's genomic makeup, namely an average decrease of sequence complexity over time. Usually, to accomplish very similar functions, lower entropy corresponds to less energy required and, hence, more efficiency which can be driven by evolution or selection.

Moreover, the plot indicates periods of relative stability interspersed with spikes and dips in complexity. Notice that some noise exists that can be related to the higher variability of the size of some sequences but that is contained in the filtered interval. On the other hand, some inflexion points may correlate with the emergence of new variants or adaptations in the virus's evolutionary strategy.

Recently, we introduced AlcoR [41]. AlcoR is a mapping and visualization tool for detecting low-complexity regions in biological data. AltaiR complements AlcoR in the sequence complexity analysis, namely by adding the capability to relate sequences using a temporal dimension.

Frequency profiles application

We used the filtered SARS-CoV-2 multi-FASTA to access the frequency application and computed its nucleotide variation over time, as shown in Figure 5.

The figure shows a notable shift in the nucleotide composition of SARS-CoV-2 over the observed period of time. Specifically, the frequency of thymine (T) bases has gradually increased, while there has been a (approximate) corresponding decrease in the frequency

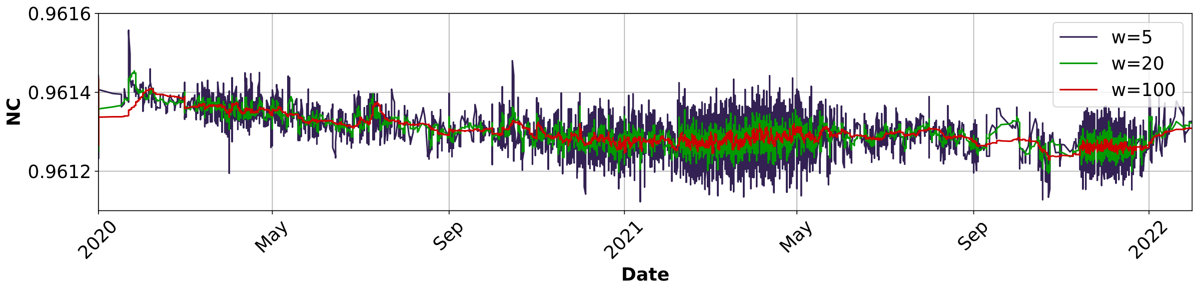

Figure 4. Complexity Plot showing variations in the normalized complexity (NC) values over time. The plot illustrates changes in NC with different window sizes, providing insights into the data trends from 2020 to 2022. To replicate this analysis, see Supplementary Section Reproducibility, specifically Reproducing the "Complexity profiles (NC) application"

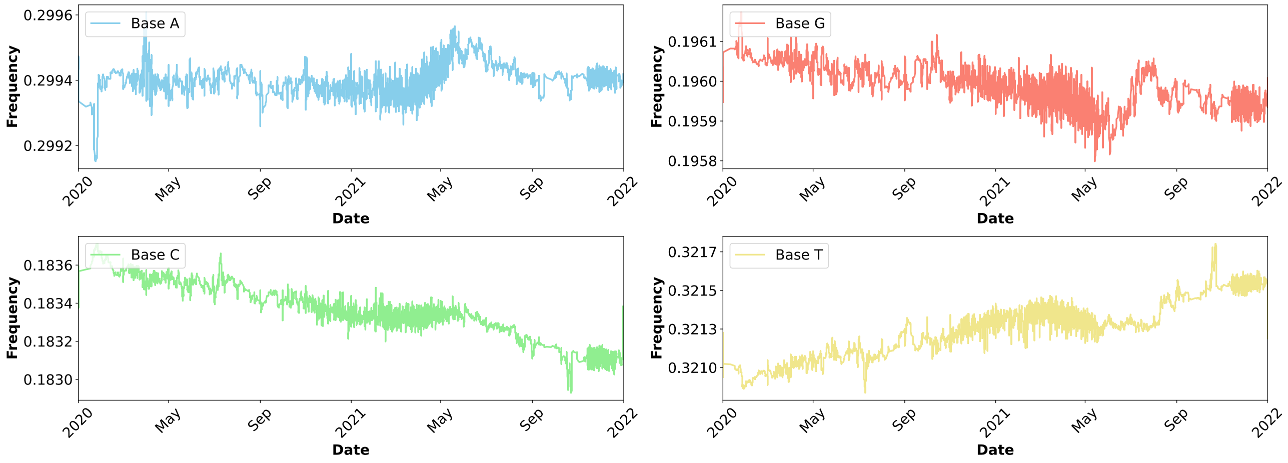

Figure 5. Frequency distribution of nucleotide bases in SARS-CoV-2 from 2020 to 2022, illustrating an increase in thymine (T) bases and a decrease in cytosine (C) bases. To replicate this analysis, see Supplementary Section Reproducibility, specifically Reproducing the "Frequency profiles application"

of cytosine (C) bases. This trend indicates the virus's ongoing evolutionary process within the human host.

The increase in T bases and the decrease in C bases can be attributed to various factors. Transition mutations, where a purine is substituted for another purine or a pyrimidine for another pyrimidine (in this case, C to T), are common mutations in RNA viruses. These mutations may be driven by the error-prone nature of RNA-dependent RNA polymerase, which is responsible for viral replication [80, 81, 82]. Additionally, such mutations may confer selective advantages to the virus, potentially impacting its transmissibility, pathogenicity, and immune escape capabilities [83, 84].

This trend also raises important considerations for public health and clinical interventions. For instance, changes in the viral genome could affect the efficacy of vaccines and therapeutics, emphasizing the need for continuous surveillance and adaptation of these measures [85, 86]. The observed mutations might also provide insights into the virus-host interaction dynamics, explaining how SARS-CoV-2 adapts to the human host environment over time [87].

Relative singularity (RAWs) profiles

Our study also focused on uncovering the Relative Absent Words (RAWs) of SARS-CoV-2, namely by identifying the shortest words that exist in the SARS-CoV-2 and were absent from the human host genome [88] and transcriptome [89]. Detailed statistics of this analysis are presented in Table 1.

When analyzing the data in the table, it becomes evident that there is a notable shift in nucleotide distribution as the mRAW size increases in the SARS-CoV-2 genome. Specifically, we observe a decrease in guanine (G) and cytosine (C) percentages in larger mRAWs, indicating a declining prevalence of GC content as the sequence size expands. This trend suggests a gradual shift towards AT-rich sequences in the larger mRAWs of the virus.

Interestingly, this finding contrasts with the overall nucleotide composition trend observed in SARS-CoV-2, where globally, GC content approximates 40%, and AT content around 60%. This discrepancy between the overall nucleotide composition of SARS-CoV-2 and the elevated GC percentage in the shortest unique sequences absent from the human genome suggests distinct evolutionary adaptations or functional requirements in the viral genome. Higher GC content in these sequences may confer increased structural stability or efficiency in replication and transcription processes, potentially impacting the virus's interaction with host cellular mechanisms. This pattern may reflect evolutionary pressures shaping these specific genomic regions for optimized functionality within the host environment [90, 91, 92].

On the other hand, from a previous study [69] we found out that some of the mRAWs with high GC content were localized at the surface of important proteins, such as the Spike glycoprotein in the SARS-CoV-2. This region was then found to be related to segments where the protein presents higher dynamics in time, namely higher movement of the protein to interact with the host cell.

Figure 6 and Supplementary Figure S4, through relative singularity profiles, illustrate temporal variations in both the count of mRAWs and their average GC-content in the SARS-CoV-2 genomes. Between June and August 2021, the plots indicate significant shifts in the SARS-CoV-2 genome's mRAWs count and GC content, coinciding with the Delta variant's dominance. The unique mutations of the Delta variant likely drove these genomic changes, reflecting its impact on the virus's nucleotide composition and structure.

The AltaiR tool's efficiency in processing large datasets proves

Table 1. Output statistics of AltaiR while computing the RAWs of SARS-CoV-2 that are absent from the human genome and transcriptome.

	Overall mRAWs Statistics			mRAWs Nucleotide Distribution				mRAWs AT/CG Distribution	
**kmer**	**Average**	**Variance**	**Std. Dev.**	**A**	**C**	**G**	**T**	**AT%**	**CG%**
11	0.00	0.00	0.01	3	3	4	1	36.4%	63.6%
12	8.29	0.39	0.62	584,523	561,473	720,587	679,229	49.6%	50.4%
13	119.70	11.26	3.36	8,500,431	10,168,609	11,774,411	9,386,300	44.9%	55.1%
14	726.01	46.92	6.85	60,566,946	65,041,615	67,333,051	67,209,832	49.1%	50.9%
15	2813.56	147.05	12.13	269,913,035	251,842,372	260,427,726	298,014,612	52.6%	47.4%
16	8132.19	227.32	15.08	900,499,378	717,952,376	740,116,031	971,728,183	56.2%	43.8%

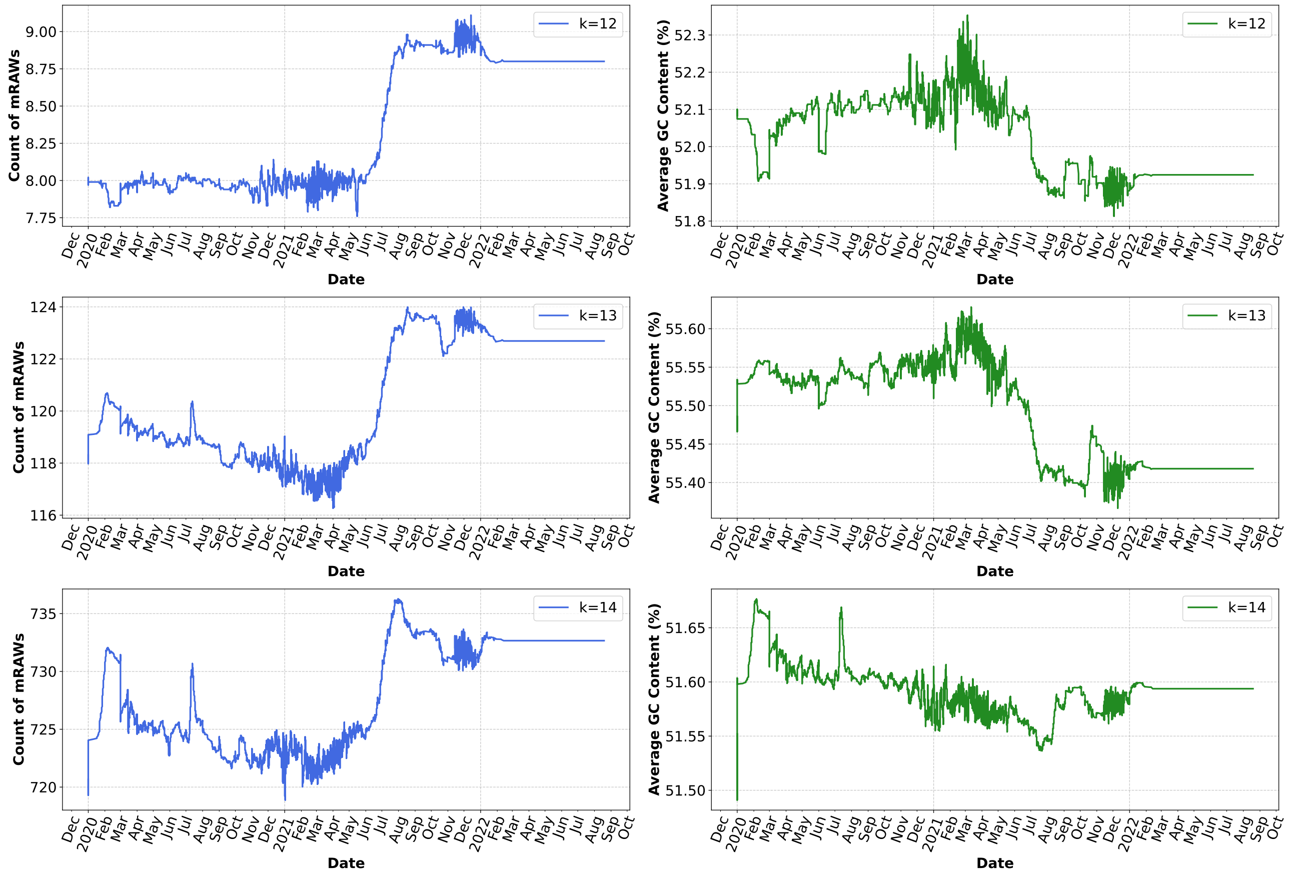

Figure 6. Relative singularity (RAWs) profiles: This set of plots illustrates the variation over time in the number of mRAWs and the average GC content in SARS-CoV-2 sequences. Each subplot corresponds to a different k-mer size (k=12, 13 and 14), showcasing the distribution of mRAWs and GC percentage across various time points. The x-axis represents time, segmented into years and months, while the y-axis shows the count of mRAWs and the GC content percentage, respectively. To replicate this analysis, see Supplementary Section Reproducibility, specifically Reproducing the "Relative singularity (RAWs) profiles"

extremely useful for identifying virus evolutionary trends. Additionally, its capability to rapidly analyze and detect mRAWs holds significant potential for diagnostics, as these sequences can be targeted explicitly in tests for accurate and prompt detection of viral infection. Moreover, mRAWs profiles can be used as potential markers for detecting new variants.

Computational efficiency

To evaluate the computational performance of the AltaiR toolkit, we conducted tests using a system with an Intel(R) Xeon(R) CPU E5-2630 v4 @ 2.20GHz (12 cores, 12 threads), 31.39 GB of RAM, running Linux 5.4.0-190-generic. Using only a single computational thread, we measured the average and total execution times, as well as peak RAM usage for each of AltaiR's main methods: filtering, NC calculation, frequency calculation, NCD calculation, and RAWs calculation (Table 2).

Filtering sequences based on length, GC content, and specific patterns took 0.0004 seconds per sequence with a peak RAM usage of 3.27 MB. To further evaluate AltaiR's efficiency, we compared its filtering capabilities with an equivalent approach using traditional Unix tools (grep and awk). Both methods were tasked with filtering sequences based on length, nucleotide content (ACGT only), and specific header patterns. Using a dataset of SARS-CoV-2 sequences, AltaiR completed the filtering task in 598.435 seconds (0.0004 sec-

Table 2. Computational performance metrics for each method in the AltaiR toolkit.

Method	Time per sequence (seconds)	Total time (seconds)	Peak RAM (MB)
Filtering	0.0004	598.435	3.27
NC	0.005	128.2557	3.41
Frequency	0.0001	3.701	2.36
NCD	0.229	2294.132	168.57
RAWs	61.558	1538.950	258.77

onds per sequence), while the grep/awk approach took 720.381 seconds (0.000816 seconds per sequence). This demonstrates that AltaiR is approximately 5.55 times faster than the traditional Unix tools for this complex filtering task. The superior performance of AltaiR can be attributed to its optimized C implementation and specialized algorithms designed for processing genomic data.

NC calculation, which generates Nucleotide Complexity profiles, required an average of 0.0050 seconds per sequence (total 128.26 seconds) with a peak RAM usage of 3.41 MB. Frequency calculation, analyzing nucleotide composition, was the fastest at 0.0001 seconds per sequence on average (total 3.701 seconds) and used the least amount of RAM at 2.36 MB peak usage. NCD calculation took an average of 0.2294 seconds per sequence (total 2294.132 seconds) and required 168.57 MB of RAM at its peak. RAWs calculation was the most computationally intensive method, requiring an average of 61.5580 seconds per sequence and 1538.95 seconds for the entire dataset, with the highest peak RAM usage of 258.77 MB. This increased resource usage is expected, as the RAWs method involves complex string matching and comparison operations to identify unique substrings.

The modular design of AltaiR allows for the integration of these methods into customizable pipelines, enabling researchers to select the most relevant analyses for their specific studies. The toolkit's ability to efficiently process large datasets, with most methods requiring less than a second per sequence on average and relatively low RAM usage, makes it well-suited for the rapidly growing field of genomic data analysis. Even the most resource-intensive method, RAWs calculation, uses less than 260 MB of RAM, making AltaiR accessible on standard desktop or laptop computers without the need for specialized high-performance computing resources.

These results showcase the overall computational efficiency of AltaiR, demonstrating its potential to streamline the analysis of large-scale genomic datasets across various research domains, from infectious disease studies to broader investigations in biology and genetics, while maintaining modest hardware requirements.

Discussion

The development of the AltaiR toolkit responds to the growing need for advanced tools capable of analyzing large and complex genomic datasets. This need arises not only from studies on infectious diseases but also from the broader fields of biology and genetics, where researchers are increasingly focusing on diverse organisms, including viruses, bacteria, protozoa, plants, and eukaryotes.

To contextualize AltaiR's capabilities, we performed a qualitative analysis with established toolkits—SeqAn [32], khmer (RRID:SCR_001156) [33, 34], GTO [35], HaploCoV [93], and bjorn [94]. Table 3 shows the results of this comparison focusing on key functionalities, efficiency metrics, and capabilities crucial for handling large-scale genomic data.

AltaiR's implementation in C allows for efficient execution and memory management, making it well-suited for handling large genomic datasets. Its support for multi-threading enables parallel computing, resulting in faster processing times compared to toolkits like khmer, HaploCoV [93, 95], and bjorn [94], which lack built-in parallel computing capabilities. AltaiR's strength lies in its alignment-free approach, which is particularly effective for processing large-scale genomic data. While alignment-free methods offer significant advantages in terms of computational efficiency and the ability to handle large-scale genomic datasets, they may not provide the same level of detailed information as alignment-based methods. Researchers should consider the trade-offs between computational efficiency and the depth of information required for their specific research questions when choosing between these approaches.

One of AltaiR's core strengths is its focus on alignment-free analysis methods, such as Normalized Compression Distance (NCD) profiles and Nucleotide Complexity (NC) profiles. These techniques enable efficient and scalable comparisons of genomic sequences without relying on computationally expensive alignment operations, setting it apart from toolkits like SeqAn, which primarily concentrates on alignment algorithms. It also differs from tools like GTO in that, while GTO possesses some alignment-free methods such as sequence compression and can be adapted to compute complexity profiles, it is not primarily designed for comprehensive alignment-free analysis. On the other hand, AltaiR's use of NCD for constructing similarity profiles enables a fast and detailed exploration of temporal patterns in genomic data, providing insights into the genomic trends of organisms over time. Additionally, AltaiR includes a feature for generating complexity profiles using NC, which is essential for quantitatively assessing the complexity of genomic sequences over time, providing insights into evolutionary pressures and adaptive responses in different organisms.

AltaiR's ability to filter sequences rigorously was demonstrated in the analysis of the SARS-CoV-2 dataset. It allows users to filter sequences based on length, GC content, and specific patterns, offering more comprehensive and customizable functionality compared to the filtering capabilities provided by SeqAn, khmer, and GTO, while HaploCoV and bjorn do not provide explicit filtering options. This advanced filtering is particularly useful for processing large-scale genomic datasets, where precise data selection is crucial for downstream analyses. For instance, AltaiR's ability to filter based on GC content can be invaluable in studies focusing on organisms with specific genomic compositions or in identifying potential contamination in sequencing data. While SeqAn and GTO offer basic filtering based on sequence length and simple pattern matching, and khmer provides k-mer-based filtering, AltaiR extends these capabilities by allowing for sophisticated filtering based on multiple criteria simultaneously. Like SeqAn, khmer, and GTO, AltaiR performs nucleotide frequency analysis. However, it extends this functionality with its unique NCD profile and RAW (Relative Absent Words) identification capability, enabling the detection of rare and atypical words within genomic sequences, a feature not found in the other toolkits. The frequency profile feature, which tracks changes in nucleotide composition, can be used to study molecular adaptations in various organisms and understand their evolutionary biology. AltaiR is optimized for processing millions of sequences efficiently, specifically targeting alignment-free methods. While SeqAn and GTO also handle large datasets, khmer focuses on k-mer analysis, and HaploCoV [93] and bjorn [94] are designed specifically for SARS-CoV-2 datasets. AltaiR's optimization is tailored towards its specific strengths, following a modular toolkit design that allows for flexibility and adaptability in genomic data analysis workflows, similar to SeqAn and GTO. HaploCoV and bjorn are specialized tools for analyzing SARS-CoV-2 datasets, with HaploCoV focusing on haplotype reconstruction and bjorn providing lineage assignment capabilities. These specialized tools complement the broader functionality offered by AltaiR and the other general-purpose toolkits.

A unique capability of AltaiR is its ability to identify RAWs in genomic and proteomic sequences. By comparing RAWs in pathogens with those in host genomes, AltaiR can uncover distinctive genomic and proteomic elements, with potential applications in pathogen-host interaction studies and the discovery of new genomic and proteomic markers for diagnostic and therapeutic purposes, such as through the combination with aptamers. Aptamers, consisting of short sequences of DNA, RNA, or peptides, serve as molecular tools capable of binding to specific target molecules or families of target molecules. They can modulate the function of specific proteins, influencing signalling pathways or exerting inhibitory or enhancing effects [96]. Notably, aptamers have shown promise in therapeutic applications, as highlighted by Keefe et al. [97]. To discover high-affinity aptamers, contemporary computational methodologies leverage deep learning in conjunction with relevant feature extraction techniques, as Emami et al. [98]. The untapped potential of using mRAWs as pertinent features in aptamer discovery

Feature / Toolkit	AltaiR	SeqAn	khmer	GTO	HaploCoV	bjorn
**Programming Language**	C	C++	Python, C++	C	Perl	Python, Bash
**Parallel Computing**	✓(Multi-threading)	✓	✗	✓	✗	✗
**Alignment-Free Analysis**	✓	✗	✓	✓(Partial, compression)	✗	✗
**Genome Filtering**	Advanced (by length, GC content, patterns)	Basic (sequence length, simple pattern matching)	Basic (k-mer-based filtering)	Basic (sequence length, simple pattern matching)	✗	✗
**NCD Profile**	✓	✗	✗	✓(with scripting alterations)	✗	✗
**Complexity Profiles (NC)**	✓	✗	✗	✓	✗	✗
**Nucleotide Frequency Analysis**	✓	✓	✓(k-mer frequencies)	✓	✗	✗
**RAW Identification**	✓	✗	✗	✗	✗	✗
**Large-Scale Data Handling**	Optimized for millions of sequences	Large data handling but less optimized	Designed for large datasets but focused on k-mer analysis	✗	Designed for large SARS-CoV-2 datasets	Designed for SARS-CoV-2 datasets
**External Dependencies**	None	Some libraries required	Python environment and dependencies	None (if not considering the external tools)	Perl modules	Python package, Docker
**Modular Toolkit Design**	✓	✓	✗	✓	✗	✗
**FASTA Format Support**	✓	✓	✓	✓	✓	✓
**K-mer Counting and Filtering**	✗	✗	✓	✗	✗	✗
**String Matching Algorithms**	✗	✓	✗	✗	✗	✗
**Haplotype Reconstruction**	✗	✗	✗	✗	✓	✗
**Lineage Assignment**	✗	✗	✗	✗	✗	✓

Table 3. Comparative analysis of AltaiR with SeqAn, khmer, GTO, HaploCoV, and bjorn.

remains uncertain, but their distinctive characteristics suggest the possibility of streamlining subsequent phases of drug discovery and bypassing certain hurdles in the quest for novel therapeutic agents.

The versatility of AltaiR's sub-tools, coupled with its programming efficiency, holds significant importance for seamless integration into intricate pipelines involving multiple tools. This applicability is particularly noteworthy in various domains, such as drug discovery, viral genome reconstruction and analysis, and diversity analysis [99, 100, 101].

Consequentially, AltaiR stands out as a comprehensive and efficient toolkit for alignment-free analysis of large-scale genomic datasets. Its unique features, optimization for handling millions of sequences, and broad applicability position it as a powerful resource for a wide range of genomic and proteomic research endeavours, complementing the functionalities offered by other established toolkits and specialized tools.

Conclusions

In this study, we introduced AltaiR, a versatile and robust toolkit designed for the advanced analysis of large-scale genomic datasets. AltaiR's alignment-free methodology efficiently manages extensive data, exemplified by its role in analyzing a large number of SARS-CoV-2 sequences. The toolkit's capabilities, including Normalized Compression Distance (NCD) profiling, Normalized Compression (NC) analysis, temporal nucleotide composition variation, and the identification of Relative Absent Words (RAWs), showcase its adaptability to diverse genomic data types and research requirements.

AltaiR's innovation lies in the integration of well-established filtering methods, such as sequence length, GC content, and pattern matching, with novel and enhanced features into a comprehensive framework designed for efficient, large-scale genomic data analysis. The toolkit introduces new methods, including the Frequency method for detailed nucleotide or amino acid occurrence analysis, and extends the capabilities of the Filter method by supporting multiple simultaneous pattern searches, absent pattern searches, and filtering by GC content, sequence length, or completeness.

Moreover, AltaiR's NCD method introduces a novel approach that uses a conjunction of reference and target files while freezing the models of one and saving computation time for other sequences. This methodology allows for substantial time savings in NCD calculations. The NC analysis provides a quantitative assessment of the complexity of genomic sequences over time, offering insights into evolutionary pressures and adaptive responses in different organisms.

A unique capability of AltaiR is its ability to identify RAWs in genomic and proteomic sequences. The RAWs method in AltaiR supports genomic and protein sequences. By comparing RAWs in pathogens with those in host genomes, AltaiR can uncover distinctive genomic and proteomic elements, with potential applications in pathogen-host interaction studies and the discovery of new genomic and proteomic markers for diagnostic and therapeutic purposes.

The combination of these tools, along with the optimization for analyzing millions of sequences, ensures high portability and ease of installation while providing the necessary capabilities for rigorous analyses. This versatility enables a streamlined workflow, allowing researchers to process raw, unfiltered data and obtain meaningful insights without the need for multiple, disconnected tools.

AltaiR's application in studying SARS-CoV-2 genomes has provided possible insights into the virus's evolution and adaptations. However, the potential of AltaiR extends beyond virology, making it a crucial tool in broader genomic and proteomic research, such as in the analysis of resistant bacteria, unravelling complex temporal patterns, and facilitating studies on the evolution and diversity of various organisms.

As genomic research advances, driven by technological advancements and the increasing complexity of biological data, AltaiR's scalability, and efficiency position it as a powerful resource for a wide range of genomic and proteomic research.

Availability of source code and requirements

- Project name: AltaiR
- Project home page: https://github.com/cobilab/altair
- Operating system(s): Linux
- Programming language: C; Python; Bash
- Other requirements: Conda v4.3.27.
- License: GPL v3.
- RRID: SCR_024752
- Biotools: altair

Data Availability

An archival copy of the code and the SARS-CoV-2 sequence data used in this study is available via the GigaScience database, GigaDB [102]. The dataset includes the filtered SARS-CoV-2 sequences and the accession identifiers of all the SARS-CoV-2 Sequences input data. Additionally, links to supplementary sequences required for the analysis are provided.
The supplementary material file for this manuscript provides detailed reproducibility instructions, covering all steps for data analysis, tool usage, environment setup, script execution, and additional results. It includes scripts for filtering sequences, generating NCD profiles, conducting complexity and frequency analyses, and constructing phylogenetic trees.

Declarations

List of abbreviations

DNA - Deoxyribonucleic acid;
HSV-1 - Herpes Simplex Virus 1;
HSV-2 - Herpes Simplex Virus 2;
ID - Identifier;
MAW - minimal absent word;
mRAW - minimal Relative Absent Word;
PmRAW - Persistent minimal Relative Absent Word;
NC - Normalized Compression;
NCD - Normalized Compression Distance;
RAW - Relative Absent Word;
RNA - Ribonucleic acid;
T2T - Telomere-2-Telomere;
VZV - Varicella-Zoster Virus;

Competing Interests

The authors declare no competing interests.

Funding

This work was partially funded by National Funds through the FCT - Foundation for Science and Technology, in the context of the project UIDB/00127/2020. J.M.S. has received funding from the EC under grant agreement 101081813, Genomic Data Infrastructure. D.P. is funded by national funds through FCT – Fundação para a Ciência e a Tecnologia, I.P., under the Scientific Employment Stimulus - Institutional Call - reference CEECINST/00026/2018.

Acknowledgements

The authors wish to thank the Finnish Computing Competence Infrastructure (FCCI) for supporting this project with computational and data storage resources.

Author's Contributions

D.P. and A.P. designed the experiment. D.P. and J.M.S. coded the tools. J.M.S. executed the data analysis. J.M.S. and D.P. discussed the results. J.M.S. and D.P. wrote the manuscript. All authors have revised the manuscript.

References

1. Khare S, Gurry C, Freitas L, Schultz MB, Bach G, Diallo A, et al. GISAID's Role in Pandemic Response. China CDC Weekly 2021;3(49):1049.
2. Hatcher EL, Zhdanov SA, Bao Y, Blinkova O, Nawrocki EP, Ostapchuck Y, et al. Virus Variation Resource–improved response to emergent viral outbreaks. Nucleic acids research 2017;45(D1):D482–D490.
3. Cochrane G, Karsch-Mizrachi I, Takagi T, Sequence Database Collaboration IN. The international nucleotide sequence database collaboration. Nucleic acids research 2016;44(D1):D48–D50.
4. Sayers EW, Cavanaugh M, Clark K, Pruitt KD, Schoch CL, Sherry ST, et al. GenBank. Nucleic acids research 2021;49(D1):D92–D96.
5. Harrison PW, Ahamed A, Aslam R, Alako BT, Burgin J, Buso N, et al. The european nucleotide archive in 2020. Nucleic acids research 2021;49(D1):D82–D85.
6. Okido T, Kodama Y, Mashima J, Kosuge T, Fujisawa T, Ogasawara O. DNA Data Bank of Japan (DDBJ) update report 2021. Nucleic acids research 2022;50(D1):D102–D105.

7. de Vries JJ, Brown JR, Couto N, Beer M, Le Mercier P, Sidorov I, et al. Recommendations for the introduction of metagenomic next-generation sequencing in clinical virology, part II: bioinformatic analysis and reporting. Journal of Clinical Virology 2021;138:104812.
8. López-Labrador FX, Brown JR, Fischer N, Harvala H, Van Boheemen S, Cinek O, et al. Recommendations for the introduction of metagenomic high-throughput sequencing in clinical virology, part I: Wet lab procedure. Journal of Clinical Virology 2021;134:104691.
9. Simon-Loriere E, Holmes EC. Why do RNA viruses recombine? Nature Reviews Microbiology 2011;9(8):617–626.
10. Li X, Giorgi EE, Marichannegowda MH, Foley B, Xiao C, Kong XP, et al. Emergence of SARS-CoV-2 through recombination and strong purifying selection. Science advances 2020;6(27):eabb9153.
11. Palmenberg AC, Spiro D, Kuzmickas R, Wang S, Djikeng A, Rathe JA, et al. Sequencing and analyses of all known human rhinovirus genomes reveal structure and evolution. Science 2009;324(5923):55–59.
12. Plummer M, de Martel C, Vignat J, Ferlay J, Bray F, Franceschi S. Global burden of cancers attributable to infections in 2012: a synthetic analysis. The Lancet Global Health 2016;4(9):e609–e616.
13. Farrell PJ. Epstein–Barr virus and cancer. Annual Review of Pathology: Mechanisms of Disease 2019;14:29–53.
14. Smatti MK, Cyprian FS, Nasrallah GK, Al Thani AA, Almishal RO, Yassine HM. Viruses and autoimmunity: a review on the potential interaction and molecular mechanisms. Viruses 2019;11(8):762.
15. Pyöriä L, Pratas D, Toppinen M, Hedman K, Sajantila A, Perdomo MF. Unmasking the tissue-resident eukaryotic DNA virome in humans. Nucleic Acids Research 2023;51(7):3223–3239.
16. Toppinen M, Sajantila A, Pratas D, Hedman K, Perdomo MF. The Human Bone Marrow Is Host to the DNAs of Several Viruses. Frontiers in cellular and infection microbiology 2021;11:329.
17. Toppinen M, Pratas D, Väisänen E, Söderlund-Venermo M, Hedman K, Perdomo MF, et al. The landscape of persistent human DNA viruses in femoral bone. Forensic Science International: Genetics 2020;48:102353.
18. Land M, Hauser L, Jun SR, Nookaew I, Leuze MR, Ahn TH, et al. Insights from 20 years of bacterial genome sequencing. Functional & integrative genomics 2015;15(2):141–161.
19. Nurk S, Koren S, Rhie A, Rautiainen M, Bzikadze AV, Mikheenko A, et al. The complete sequence of a human genome. Science 2022;376(6588):44–53.
20. Qi W, Lim YW, Patrignani A, Schläpfer P, Bratus-Neuenschwander A, Grüter S, et al. The haplotype-resolved chromosome pairs of a heterozygous diploid African cassava cultivar reveal novel pan-genome and allele-specific transcriptome features. GigaScience 2022;11.
21. Mc Cartney AM, Shafin K, Alonge M, Bzikadze AV, Formenti G, Fungtammasan A, et al. Chasing perfection: validation and polishing strategies for telomere-to-telomere genome assemblies. Nature Methods 2022;p. 1–9.
22. Alkan C, Carbone L, Dennis M, Ernst J, Evrony G, Girirajan S, et al. Implications of the first complete human genome assembly. Genome Research 2022;32(4):595.
23. Vinga S, Almeida J. Alignment-free sequence comparison—a review. Bioinformatics 2003;19(4):513–523.
24. Reinert G, Chew D, Sun F, Waterman MS. Alignment-free sequence comparison (I): statistics and power. Journal of Computational Biology 2009;16(12):1615–1634.
25. Wan L, Reinert G, Sun F, Waterman MS. Alignment-free sequence comparison (II): theoretical power of comparison statistics. Journal of Computational Biology 2010;17(11):1467–1490.
26. Zielezinski A, Vinga S, Almeida J, Karlowski WM. Alignment-free sequence comparison: benefits, applications, and tools. Genome biology 2017;18(1):1–17.
27. Zielezinski A, Girgis HZ, Bernard G, Leimeister CA, Tang K, Dencker T, et al. Benchmarking of alignment-free sequence comparison methods. Genome biology 2019;20(1):1–18.
28. Metcalf JL, Xu ZZ, Bouslimani A, Dorrestein P, Carter DO, Knight R. Microbiome tools for forensic science. Trends in biotechnology 2017;35(9):814–823.
29. Chen Z, Zhao P, Li F, Marquez-Lago TT, Leier A, Revote J, et al. iLearn: an integrated platform and meta-learner for feature engineering, machine-learning analysis and modeling of DNA, RNA and protein sequence data. Briefings in bioinformatics 2020;21(3):1047–1057.
30. Yang J, Chen X, McDermaid A, Ma Q. DMINDA 2.0: integrated and systematic views of regulatory DNA motif identification and analyses. Bioinformatics 2017;33(16):2586–2588.
31. Min JL, Hemani G, Davey Smith G, Relton C, Suderman M. Meffil: efficient normalization and analysis of very large DNA methylation datasets. Bioinformatics 2018;34(23):3983–3989.
32. Döring A, Weese D, Rausch T, Reinert K. SeqAn an efficient, generic C++ library for sequence analysis. BMC bioinformatics 2008;9(1):1–9.
33. Crusoe MR, Alameldin HF, Awad S, Boucher E, Caldwell A, Cartwright R, et al. The khmer software package: enabling efficient nucleotide sequence analysis. F1000Research 2015;4.
34. Standage D, Aliyari A, Cohen LJ, Crusoe MR, Head T, Irber L, et al. khmer release v2.1: software for biological sequence analysis. Journal of Open Source Software 2017;2(15):272. https://doi.org/10.21105/joss.00272.
35. Almeida JR, Pinho AJ, Oliveira JL, Fajarda O, Pratas D. GTO: a toolkit to unify pipelines in genomic and proteomic research. SoftwareX 2020;12:100535.
36. Drezen E, Rizk G, Chikhi R, Deltel C, Lemaitre C, Peterlongo P, et al. GATB: genome assembly & analysis tool box. Bioinformatics 2014;30(20):2959–2961.
37. Lee J, Lee AJ, Lee JK, Park J, Kwon Y, Park S, et al. Mutalisk: a web-based somatic MUTation AnaLyIS toolKit for genomic, transcriptional and epigenomic signatures. Nucleic acids research 2018;46(W1):W102–W108.
38. Sims D, Ilott NE, Sansom SN, Sudbery IM, Johnson JS, Fawcett KA, et al. CGAT: computational genomics analysis toolkit. Bioinformatics 2014;30(9):1290–1291.
39. Hiltemann S, Mei H, de Hollander M, Palli I, van der Spek P, Jenster G, et al. CGtag: complete genomics toolkit and annotation in a cloud-based Galaxy. GigaScience 2014;3(1):2047–217X.
40. de Koning W, Miladi M, Hiltemann S, Heikema A, Hays JP, Flemming S, et al. NanoGalaxy: Nanopore long-read sequencing data analysis in Galaxy. GigaScience 2020;9(10):giaa105.
41. Silva JM, Qi W, Pinho AJ, Pratas D. AlcoR: alignment-free simulation, mapping, and visualization of low-complexity regions in biological data. GigaScience 2023;.
42. Loman NJ, Quinlan AR. Poretools: a toolkit for analyzing nanopore sequence data. Bioinformatics 2014;30(23):3399–3401.
43. Knight R, Maxwell P, Birmingham A, Carnes J, Caporaso JG, Easton BC, et al. PyCogent: a toolkit for making sense from sequence. Genome biology 2007;8(8):1–16.
44. Shen W, Le S, Li Y, Hu F. SeqKit: a cross-platform and ultrafast toolkit for FASTA/Q file manipulation. PloS one 2016;11(10):e0163962.
45. Alam KK, Chang JL, Burke DH. FASTAptamer: a bioinformatic toolkit for high-throughput sequence analysis of combinatorial selections. Molecular Therapy-Nucleic Acids 2015;4:e230.
46. Ott M, Edunov S, Baevski A, Fan A, Gross S, Ng N, et al. fairseq: A Fast, Extensible Toolkit for Sequence Modeling. In: Pro-

ceedings of the 2019 Conference of the North American Chapter of the Association for Computational Linguistics (Demonstrations) Minneapolis, Minnesota: Association for Computational Linguistics; 2019. p. 48–53. https://aclanthology.org/N19-4009.

47. Chen C, Chen H, Zhang Y, Thomas HR, Frank MH, He Y, et al. TBtools: an integrative toolkit developed for interactive analyses of big biological data. Molecular plant 2020;13(8):1194–1202.

48. Gabler F, Nam SZ, Till S, Mirdita M, Steinegger M, Söding J, et al. Protein sequence analysis using the MPI bioinformatics toolkit. Current Protocols in Bioinformatics 2020;72(1):e108.

49. Arkin AP, Cottingham RW, Henry CS, Harris NL, Stevens RL, Maslov S, et al. KBase: the United States department of energy systems biology knowledgebase. Nature biotechnology 2018;36(7):566–569.

50. Kryukov K, Ueda MT, Nakagawa S, Imanishi T. Nucleotide Archival Format (NAF) enables efficient lossless reference-free compression of DNA sequences. Bioinformatics 2019;35(19):3826–3828.

51. Deorowicz S, Danek A, Li H. AGC: compact representation of assembled genomes with fast queries and updates. Bioinformatics 2023;39(3):btad097.

52. Grabowski S, Kowalski TM. MBGC: Multiple Bacteria Genome Compressor. GigaScience 2022;11.

53. Pinho AJ, Pratas D. MFCompress: a compression tool for FASTA and multi-FASTA data. Bioinformatics 2014;30(1):117–118.

54. Hosseini M, Pratas D, Pinho AJ. Cryfa: a secure encryption tool for genomic data. Bioinformatics 2019;35(1):146–148.

55. Lu J, Salzberg SL. Removing contaminants from databases of draft genomes. PLoS computational biology 2018;14(6):e1006277.

56. Sajantila A, Editors' Pick: Contamination has always been the issue! BioMed Central; 2014.

57. Li M, Chen X, Li X, Ma B, Vitányi PM. The similarity metric. IEEE transactions on Information Theory 2004;50(12):3250–3264.

58. Li M, Vitányi P, et al. An introduction to Kolmogorov complexity and its applications, vol. 3. Springer; 2008.

59. Kolmogorov AN. Three approaches to the quantitative definition of information. Problems of information transmission 1965;1(1):1–7.

60. Pratas D, Silva RM, Pinho AJ. Comparison of compression-based measures with application to the evolution of primate genomes. Entropy 2018;20(6):393.

61. Cilibrasi RL, Vitányi PM. Fast Phylogeny of SARS-CoV-2 by Compression. Entropy 2022;24(4):439.

62. Silva M, Pratas D, Pinho AJ. AC2: An Efficient Protein Sequence Compression Tool Using Artificial Neural Networks and Cache-Hash Models. Entropy 2021;23(5):530.

63. Cebrián M, Alfonseca M, Ortega A. Common pitfalls using the normalized compression distance: What to watch out for in a compressor. Communications in Information & Systems 2005;5(4):367–384.

64. Hosseini M, Pratas D, Pinho AJ. On the role of inverted repeats in DNA sequence similarity. In: International Conference on Practical Applications of Computational Biology & Bioinformatics Springer; 2017. p. 228–236.

65. Pratas D, Hosseini M, Pinho AJ. Substitutional tolerant Markov models for relative compression of DNA sequences. In: International Conference on Practical Applications of Computational Biology & Bioinformatics Springer; 2017. p. 265–272.

66. Silva M, Pratas D, Pinho AJ. Efficient DNA sequence compression with neural networks. GigaScience 2020;9(11):giaa119.

67. Pratas D, Pinho AJ. On the approximation of the Kolmogorov complexity for DNA sequences. In: Iberian Conference on Pattern Recognition and Image Analysis Springer; 2017. p. 259–266.

68. Silva RM, Pratas D, Castro L, Pinho AJ, Ferreira PJ. Three minimal sequences found in Ebola virus genomes and absent from human DNA. Bioinformatics 2015;31(15):2421–2425.

69. Pratas D, Silva JM. Persistent minimal sequences of SARS-CoV-2. Bioinformatics 2020 07;36(21):5129–5132.

70. Pratas D. Compression and analysis of genomic data. PhD thesis, Universidade de Aveiro (Portugal); 2016.

71. Béal MP, Mignosi F, Restivo A. Minimal forbidden words and symbolic dynamics. In: Annual Symposium on Theoretical Aspects of Computer Science Springer; 1996. p. 555–566.

72. Crochemore M, Mignosi F, Restivo A. Automata and Forbidden Words. Inf Process Lett 1998 Aug;67(3):111–117.

73. Pinho AJ, Ferreira PJ, Garcia SP, Rodrigues JM. On finding minimal absent words. BMC bioinformatics 2009;10(1):1–11.

74. Kosche M, Koß T, Manea F, Siemer S. Absent subsequences in words. In: International Conference on Reachability Problems Springer; 2021. p. 115–131.

75. Bernardini G, Marchetti-Spaccamela A, Pissis SP, Stougie L, Sweering M. Constructing strings avoiding forbidden substrings. In: 32nd Annual Symposium on Combinatorial Pattern Matching (CPM 2021), vol. 191; 2021. p. 1–9.

76. Koulouras G, Frith MC. Significant non-existence of sequences in genomes and proteomes. Nucleic acids research 2021;49(6):3139–3155.

77. Akon M, Akon M, Kabir M, Rahman MS, Rahman MS. ADACT: a tool for analysing (dis) similarity among nucleotide and protein sequences using minimal and relative absent words. Bioinformatics 2021;37(10):1468–1470.

78. cobilab, AltaiR: a C toolkit for alignment-free and temporal analysis of multi-FASTA data. cobilab; 2024. https://github.com/cobilab/altair, [Online; accessed 11-October-2024].

79. NCBI, NCBI Virus. NCBI; 2023. https://www.ncbi.nlm.nih.gov/labs/virus/vssi/#/virus?SeqType_s=Nucleotide&VirusLineage_ss=Wuhan%20seafood%20market%20pneumonia%20virus,%20taxid:2697049&Completeness_s=complete&HostLineage_ss=Homo%20sapiens%20(human),%20taxid:9606&CreateDate_dt=2000-01-01T00:00:00.00Z%20TO%202022-09-28T23:59:59.00Z, [Online; accessed 27-November-2023].

80. Singh D, Yi SV. On the origin and evolution of SARS-CoV-2. Experimental & Molecular Medicine 2021;53(4):537–547.

81. Drake JW, Holland JJ. Mutation rates among RNA viruses. Proceedings of the National Academy of Sciences 1999;96(24):13910–13913.

82. Sanjuán R, Nebot MR, Chirico N, Mansky LM, Belshaw R. Viral mutation rates. Journal of virology 2010;84(19):9733–9748.

83. Grubaugh ND, Hanage WP, Rasmussen AL. Making sense of mutation: what D614G means for the COVID-19 pandemic remains unclear. Cell 2020;182(4):794–795.

84. Harvey WT, Carabelli AM, Jackson B, Gupta RK, Thomson EC, Harrison EM, et al. SARS-CoV-2 variants, spike mutations and immune escape. Nature Reviews Microbiology 2021;19(7):409–424.

85. Korber B, Fischer WM, Gnanakaran S, Yoon H, Theiler J, Abfalterer W, et al. Tracking changes in SARS-CoV-2 spike: evidence that D614G increases infectivity of the COVID-19 virus. Cell 2020;182(4):812–827.

86. Plante JA, Liu Y, Liu J, Xia H, Johnson BA, Lokugamage KG, et al. Spike mutation D614G alters SARS-CoV-2 fitness. Nature 2021;592(7852):116–121.

87. Walls AC, Park YJ, Tortorici MA, Wall A, McGuire AT, Veesler D. Structure, function, and antigenicity of the SARS-CoV-2 spike glycoprotein. Cell 2020;181(2):281–292.

88. NCBI, Homo sapiens genome assembly T2T-CHM13v2.0. NCBI; 2023. https://www.ncbi.nlm.nih.gov/datasets/genome/GCF_009914755.1/, [Online; accessed 27-November-2023].

89. NCBI, Human Genome Resources at NCBI. NCBI; 2023. https://www.ncbi.nlm.nih.gov/genome/guide/human/, [Online; accessed 27-November-2023].
90. Wang Y, Mao JM, Wang GD, Luo ZP, Yang L, Yao Q, et al. Human SARS-CoV-2 has evolved to reduce CG dinucleotide in its open reading frames. Scientific Reports 2020;10(1):12331.
91. Takata MA, Gonçalves-Carneiro D, Zang TM, Soll SJ, York A, Blanco-Melo D, et al. CG dinucleotide suppression enables antiviral defence targeting non-self RNA. Nature 2017;550(7674):124–127.
92. Afrasiabi A, Alinejad-Rokny H, Khosh A, Rahnama M, Lovell N, Xu Z, et al. The low abundance of CpG in the SARS-CoV-2 genome is not an evolutionarily signature of ZAP. Scientific reports 2022;12(1):2420.
93. Chiara M, Horner DS, Ferrandi E, Gissi C, Pesole G. HaploCoV: unsupervised classification and rapid detection of novel emerging variants of SARS-CoV-2. Communications Biology 2023;6(1):443.
94. Lab A, Bjorn: Bioinformatics Pipeline for Analyzing SARS-CoV-2 Genomes;. Accessed: 2024-06-13. https://github.com/andersen-lab/bjorn.
95. Chiara M, HaploCoV: A Tool for Haplotype Analysis in SARS-CoV-2 Genomes;. Accessed: 2024-06-13. https://github.com/matteo14c/HaploCoV.
96. Crivianu-Gaita V, Thompson M. Aptamers, antibody scFv, and antibody Fab'fragments: An overview and comparison of three of the most versatile biosensor biorecognition elements. Biosensors and Bioelectronics 2016;85:32–45.
97. Keefe AD, Pai S, Ellington A. Aptamers as therapeutics. Nature reviews Drug discovery 2010;9(7):537–550.
98. Emami N, Ferdousi R. AptaNet as a deep learning approach for aptamer–protein interaction prediction. Scientific reports 2021;11(1):6074.
99. Herzig V, Cristofori-Armstrong B, Israel MR, Nixon SA, Vetter I, King GF. Animal toxins—Nature's evolutionary-refined toolkit for basic research and drug discovery. Biochemical pharmacology 2020;181:114096.
100. Pratas D, Toppinen M, Pyöriä L, Hedman K, Sajantila A, Perdomo MF. A hybrid pipeline for reconstruction and analysis of viral genomes at multi-organ level. GigaScience 2020;9(8):giaa086.
101. Adolfo LM, Rao X, Dixon RA. Identification of Pueraria spp. through DNA barcoding and comparative transcriptomics. BMC Plant Biology 2022;22(1):1–18.
102. Silva JM, Pinho AJ, Pratas D. Supporting data for "AltaiR: a C toolkit for alignment-free and temporal analysis of multi-FASTA data." GigaScience Database 2024. https://doi.org/10.5524/102587.

Manuscript Click here to access/download;Manuscript;manuscript_pdf.pdf

GigaScience, 2023, 1–0

doi: xx.xxxx/xxxx
Manuscript in Preparation
Technical Note

TECHNICAL NOTE

AltaiR: a C toolkit for alignment-free and temporal analysis of multi-FASTA data

Jorge M. Silva[1,2,*], Armando J. Pinho[1,2] and Diogo Pratas[1,2,3,*]

[1]IEETA/LASI, Institute of Electronics and Informatics Engineering of Aveiro, University of Aveiro, Portugal and [2]DETI, Department of Electronics, Telecommunications and Informatics, University of Aveiro, Portugal and [3]DoV, Department of Virology, University of Helsinki, Finland

[*]Correspondence address. Jorge M. Silva and Diogo Pratas. E-mail: jorge.miguel.ferreira.silva@ua.pt and pratas@ua.pt

Abstract

Background: The vast number of viral genome sequences generated during the latest pandemic has presented new challenges for computational analysis. Analyzing millions of viral genomes in multi-FASTA format is computationally demanding, especially when using alignment-based methods. Most existing methods are not designed to handle such large datasets, often requiring the analysis to be divided into smaller parts to obtain results using available computational resources.
Findings: We introduce AltaiR, a toolkit for analyzing multiple sequences in multi-FASTA format using exclusively alignment-free methodologies. AltaiR enables the identification of singularity and similarity patterns within sequences and computes static and temporal dynamics without restrictions on the number or size of input sequences. It automatically filters low-quality, biased, or deviant data. We demonstrate AltaiR's capabilities by analyzing more than 1.5 million full SARS-CoV-2 sequences, revealing interesting observations regarding viral genome characteristics over time, such as shifts in nucleotide composition, decreases in average Kolmogorov sequence complexity, and the evolution of the smallest sequences not found in the human host.
Conclusions: AltaiR can identify temporal characteristics and trends in large numbers of sequences, making it ideal for scenarios involving endemic or epidemic outbreaks with vast amounts of available sequence data. Implemented in C with multi-threading and methodological optimizations, AltaiR is computationally efficient, flexible, and dependency-free. It accepts any sequence in FASTA format, including amino acid sequences. The complete toolkit is freely available at `https://github.com/cobilab/altair`.

Key words: Alignment-free toolkit; Relative Absent Words; Data compression; Temporal patterns; Viral genomes; multi-FASTA;

Introduction

The recent availability of millions of SARS-CoV-2 complete genomes that emerged from the COVID-19 pandemic has remarkably changed the scientific workflow. It starts with the parallelization of the genome's sequencing and assembly through many scientific and industrial entities, followed by the centralized upload of each SARS-CoV-2 assembled sequence in FASTA format and the respective metadata, while respecting a light data curation, mainly in the following database repositories: GISAID [1], NCBI [2], and INSDC [3], that include GeneBank [4], ENA [5], and DDBJ [6].

Although the incredible growing pace at which these complete genomes emerged and have been made publicly available, their downstream analysis faces new challenges mainly related to the vast number of genomes, characteristics and format of the data, and multiplexed sequencing-reconstruction.

Specifically, one challenge emerged due to the availability of millions of genomes split by the respective headers with basic information in a single file. This file format is known as Multi-FASTA, but it is generically called FASTA. There are alternative and more efficient file format representations, such as those representing only differences according to a reference. Still, the majority of the downstream analysis tools require the FASTA format. Moreover, storing only the differences would require multiple references to perform additional analyses at different taxonomic levels. The alternative way of using a single reference sequence becomes inefficient, if there is high dissimilarity.

Furthermore, for processing up to a hundred thousand genomes,

Compiled on: October 11, 2024.
Draft manuscript prepared by the author.

only a minority of the tools are prepared, while the availability of these tools is very scarce to process millions. The main reason is these programs' use of temporary files to decrease the computational memory and related computational time. However, creating temporary files times millions of entries is not efficiently affordable.

Another challenge is the existence of outlier or recombinant genomes in the set. The outlier entries are unwanted sequences uploaded by entities that show profound differences in sequencing and assembly methodologies. Examples of these features are the presence/absence of targeted capture, the assembly methodology using appropriate/inappropriate references, proper/improper exclusion of contaminants, accurate/inaccurate variant call, accurate/inaccurate base masking, or substantial differences in SNPs caused by postmortem degradation. Although several directives have been provided [7, 8], these genomes still exist and are continuously being uploaded. Recombinant genomes usually show a higher degree of variants or chimeric formations after cell co-infection [9]. Due to these temporary increases in the accumulation of genomic variations, recombinant genomes can be associated with the emergence of outbreak strains, as exemplified by coronaviruses and rhinoviruses [10, 11]. For downstream analysis, the objective is to discard outliers while maintaining the recombinant genomes. However, we currently lack fast genome filtering tools that assert a particular trait distribution to minimize outliers in the data while maintaining the average genome data of these recombinant genomes.

Beyond the SARS-CoV-2 sequences, the upload of large quantities of viral genomes in FASTA format is also substantially increasing. Because viral genomes are sometimes directly associated with hosting health conditions, namely in cancer or autoimmune diseases, the number of these reconstructed genomes is expected to increase dramatically for clinical and forensic purposes [12, 13, 14]. Some examples are the steadily increasing availability of Parvoviruses, Polyomaviruses, Herpesviruses, and Papillomaviruses [15, 16, 17]. Moreover, this increase is also in other types of organisms, such as organisms with larger genome sequences, namely fungi and bacteria [18], especially now with the higher rates of antibiotic resistance and the availability of complete genome sequences [19, 20] associated with the development of the Telomere-2-Telomere (T2T) technology [21, 22].

The current disposal of the massive number of genomes from a single species also provides the opportunity to study a species sequence over time, namely the temporal patterns and characteristics of the genomes or proteomes in a temporal dimension. Accordingly, the following questions emerge:

- How does the nucleotide composition of the genomes change over time?
- How does the sequence entropy of a species change in time?
- How does the similarity of parasitic sequence species vary according to the first known sequence?
- What are the shortest sub-sequences of a parasitic species not in a host?
- How do these shortest sequences change in time?

These complex questions can be answered with this type of data in synergy with the alignment-free method that is provided in this manuscript.

Although alignment methods offer an intuitive and enhanced local resolution that prevails in comparative analysis of specific features in a low number of sequences, large-scale sequence quantities require unfeasible computational resources under a desirable accuracy, limiting their applicability in multiple temporal analyses. On the other hand, the substantial increase in the development and availability of alignment-free methods [23, 24, 25, 26, 27] have provided clear advantages using feasible computational resources.

In the literature, there are multiple toolkits for specific sequence transformation and analyses applications, namely microbiome tools for forensic science [28], for machine-learning analysis and modelling of genomic and proteomic sequence data [29], for visualization of regulatory DNA motif identification and analyses [30], efficient analysis of DNA methylation [31], among many others. On the other hand, there are toolkits or platforms with a much broader application, namely SeqAn [32], khmer [33, 34], GTO [35], GATB [36], Mutalisk [37], CGAT [38], CGtag [39], nanoGalaxy [40], AlcoR [41], Poretools [42], Pycogent [43], SeqKit [44], FASTAptamer [45], fairseq [46], SeqKit [44], TBtools [47], MPI bioinformatics toolkit [48], KBase [49], among many others. Some of these toolkits or platforms are interactive through web browsers offering friendly environments, while others are prone to fast and efficient computation through the command line.

Benchmarking genomic toolkits by direct comparison can be misleading due to their distinctive design philosophies and unique feature sets. Each toolkit is crafted with specific goals in mind, which may not align perfectly with those of another. Hence, simple side-by-side comparisons might not only be uninformative but could obscure the individual strengths of each tool. Instead, a more nuanced approach involves evaluating a toolkit based on its specific features and objectives. Important aspects to consider include the toolkit's innovative features, the research questions it facilitates, its efficiency and ease of use, and any software dependencies it might have. These criteria form the core of high-level benchmarking, focusing on what each toolkit brings to the scientific community rather than how it matches up against others.

On a deeper level, benchmarking should employ both synthetic data, which provides a controlled environment to assess tool performance predictably, and natural data, which offers real-world challenges. Synthetic data testing is essential and should be a standard preliminary test, whereas natural data testing, although occasionally challenging due to the variability of biological data, is crucial for understanding how the tool performs under realistic conditions. Additionally, the reproducibility and, when feasible, the repeatability of results are paramount to confirm the reliability and effectiveness of each tool. This rigorous approach ensures that the tools not only meet theoretical expectations but also hold up under practical application.

In this article, we present AltaiR, a toolkit with alignment-free methods for the temporal analysis of multi-FASTA data, specifically large-scale numbers of genomes or proteomes, including millions (or billions) of sequences in a single FASTA file. The AltaiR toolkit offers an efficient and convenient approach for pathogen-host analyses in endemic or pandemic scenarios (but not limited to). Importantly, the AltaiR method includes a flexible tool to filter sequences undesired from the dataset through parameterized characteristics. AltaiR contains both reference-free and reference-based tools. The reference-free tools are for analysing sequence entropy and nucleotide frequency changes over time. The reference-based tools are divided into two main wings, similarity and singularity. For both, highly efficient implementations are provided. The AltaiR is developed in C language and is provided as open-source software. In the next section, we enumerate the features and characteristics of the AltaiR toolkit, including the description and formalization of the methods. Then, we benchmark each tool from the toolkit using synthetic data while ensuring the full repeatability of the experiment. Afterwards, we present results using natural data, namely an application for a vast SARS-CoV-2 set of genomes. Finally, we discuss the results obtained and draw some conclusions.

Methods

This section describes the methods and their respective implementation into computer tools that constitute the AltaiR toolkit. The details of the parameters and how to reproduce the methods are available in Supplementary Sections 2 and 3. We recommend using compressed data, namely through the compression of the files us-

ing specialized tools. For fast access, NAF [50], AGC [51], and, for bacteria, MBGC [52] can be used. For a higher compression ratio but slower access, we recommend MFCompress [53]. When using sensitive data, Cryfa [54] can be used to compact and encrypt the data.

Figure 1 describes the workflow of AltaiR by dividing the data according to three types: the parasite reference sequences, the parasite sequences (after filtering the outliers or unwanted data), and the host omics sequences. The workflow contains seven main methods.

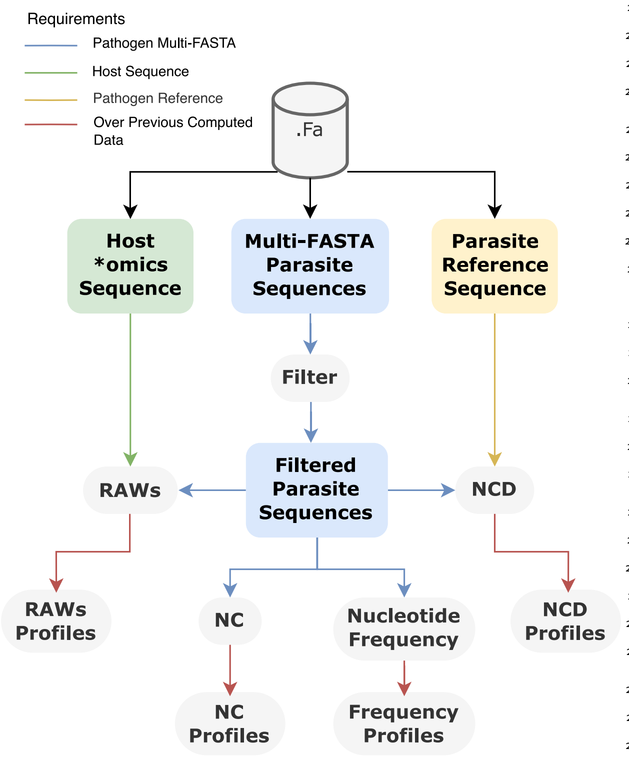

Figure 1. Workflow of the AltaiR toolkit describing the principal seven phases along with the three input types of sequences in FASTA format.

The first method is filtering the sequences by characteristics, essentially removing sequences that contain different traits from what is expected or the average. The second method is the Normalized Compression Distance (NCD) for measuring the similarity between two sequences. The NCD extension is provided in the third method through the use of the NCD profiles. These are generated by the similarity comparison of the existent sequences according to a reference. The fourth method is the Normalized Compression (NC) profiles that depict the compression variability of the sequences. The fifth method is the frequency profiles, allowing to visualize changes in the nucleotide composition over time. The sixth method is the RAWs (Relative Absent Words) mapping that identifies the shortest sequences in the parasite but absent from the host genome/transcriptome. Finally, the seventh method provides profiles regarding the RAWs according to time. It is important to note that for temporal analyses, such as the NC, NCD profiles and frequency profiles, the input sequences must be sorted by date. This sorting is crucial for accurately capturing trends and patterns over time.

The further subsections provide details on the methods, including their mathematical formalizations and characteristics.

Filter sequences by characteristics

The public databases of multiple genome and proteome sequences from a specific organism usually contain sequences not within the average group. The most frequent reasons are the inclusion of contaminant sequences [55, 56] and differences in the sequencing or assembly procedures. Removing some of these sequences from the dataset is important for downstream analyses. However, manual inspection is slow and can introduce errors. Therefore, automatically filtering these sequences by characteristics is important and can provide unbiased selections while maintaining visible static and uniform characteristics. AltaiR incorporates established guidelines and best practices [7, 8] to address potential contamination and quality issues through automated filtering. This process ensures only high-quality, contiguous sequences are included in the analysis, minimizing bias from incomplete or contaminated data.

Accordingly, this sub-method filters a multi-FASTA file, specifically FASTA reads, by input characteristics. These characteristics can be the alphabet composition, sequence completeness or length, GC quantity, multiple string header patterns, or absence of patterns (anti-patterns). Completeness refers to the availability of the full, uninterrupted sequence of an organism's genome, rather than just partial or fragmented sequences. The computation can be applied to different sequences; for example, a specific filtering can be applied to SARS-CoV-2 while another for B19V. Moreover, the filtering characteristics can be intersected through a single run.

Consider a source that has generated n sequences from a finite alphabet Ξ with size $|\Xi|$. The nature of the source is unknown but in each sequence x^i, where $x^0, ..., x^n$, the internal symbols are known.

The filtering aims to evaluate specific characteristics that occur in each x^i, and those which respect the conditions are used for further downstream analyses. Moreover, each x^i contains a header alphanumeric sequence, y^i, that generically identifies x_i. Specifically, the y^i is the metadata of x^i and usually contains the name of the organism, the date, and a unique ID, among others.

The following definitions provide information on the filtering for each main characteristic or feature, including the sequence length, alphabet composition, minimum and maximum GC percentage, header patterns and anti-patterns.

Sequence length

In multiple cases, sequences labelled as complete are nearly complete, or the length is shorter than a certain length. This can occur, for instance, due to the sequencing depth being very low or some scaffolds could not be linked in the assembly process. AltaiR can filter sequences by the minimal and maximal length to minimise these issues. Therefore, for any string x^i with length $|x^i|$ the set of final sequences, Σ, is composed by

$$x^i \in \Sigma \iff \mathcal{N}_{\max} \geq \mathcal{N}(x^i) \geq \mathcal{N}_{\min}, \tag{1}$$

where $\mathcal{N}$ is the length of the sequence x_i and $\mathcal{N}_{\min}$ and $\mathcal{N}_{\max}$ the minimum and maximum length, respectively.

Alphabet composition

It is common to find some reads of a multi-FASTA format containing characters outside a desired set. For example, the 'R' symbol at the DNA sequence level stands for a purine (an A or G base). This characteristic is frequent with other symbols in protein sequences, leading to an unbalance between the set cardinality of the sequences. This ambiguity generates complexity in the analysis when using many software tools and can create deviant results. Therefore, the AltaiR method efficiently filters sequence symbols containing a certain alphabet. Additionally, it can filter the reads by composition, including completeness, when combined with the length.

Minimum and maximum GC percentage

The GC percentage is usually an important feature to evaluate if the distribution of a particular sequence is within a desirable average set. Specifically, the GC percentage is given by the number of cytosine (C) and guanine (G) bases in a string x^i with length $|x^i|$ according to

$$\mathcal{GC}(x^i) = \frac{100}{|x^i|} \sum_{j=1}^{|x^i|} \mathcal{I}_{\Xi}(x^i_j), \tag{2}$$

where x^i_j is each symbol of x^i (assuming causal order), Ξ is a subset alphabet containing the symbols $\{G, C\}$ and $\mathcal{I}$ an indicator function constrained to

$$\mathcal{I}_{\Xi}(x) = \begin{cases} 1, x \in \Xi \\ 0, x \notin \Xi \end{cases} . \tag{3}$$

Then, the set of final sequences, Σ, is composed by

$$x^i \in \Sigma \iff \mathcal{GC}_{\max} \geq \mathcal{GC}(x^i) \geq \mathcal{GC}_{\min}, \tag{4}$$

where $\mathcal{GC}_{\min}$ and $\mathcal{GC}_{\max}$ are two real values where $\mathcal{GC}_{\max} > \mathcal{GC}_{\min} \in [0;100]$.

Header patterns and anti-patterns

The header patterns are strings to match in each sequence header, h^i, that can ignore or consider a certain sequence read if the substring pattern is absent or present. This is the analogous process of the well-known grep tool, but instead of looking into the whole FASTA file, it only filters by the header sequence. This type of filtering is very important when only a certain type of sequence is to be considered or sequences with a certain header substring must not pass to the final set of sequences, Σ.

The AltaiR toolkit provides filtering of the header patterns using substrings that occur in the header or that are absent (ignore pattern) using conjoint filtering that is not limited to the number of strings to match or ignore. For example, using the patterns "Human", "Herpes" and "Alpha" and the anti-pattern "Simplex" in the whole viral reference database from the NCBI will select all the FASTA reads that the headers contain the words Human, Herpes, and Alpha, selecting only the human Alphaherpesvirus that are composed by the HSV-1 (Herpes Simplex Virus 1), HSV-2 (Herpes Simplex Virus 2), and VZV (Varicella-Zoster Virus); because the anti-pattern "Simplex" was also used, then, only the VZV will be contained in Σ.

Similarity (NCD) profiles

In temporal analysis, some applications require measuring the similarity of the most recent genomes to the first ones being sequenced and assembled, such as the first reference genome. These measures will allow us to understand the evolution rate over time and if evolutionary acceleration peaks have been found in specific periods. Accordingly, if the genomes are sorted according to sample isolation date (an NCBI VSSI option while downloading the data), then measuring the distance of the first genome according to the remaining in the multi-FASTA file provides this similarity information.

To compute the similarity between the reference genome and the remaining sequences, the Normalized Compression Distance (NCD), which is a similarity distance that approximates the Kolmogorov complexity through data compression, is used [57, 58, 59]. The NCD has many applications for genomic and proteomic assembled sequences [60], including the similarity measure for the COVID-19 pandemics [61, 62].

Formally, the NCD between a reference y and a target x sequence is given by

$$\mathcal{D}(x, y) = \frac{C(x, y) - \min\{C(x), C(y)\}}{\max\{C(x), C(y)\}}, \tag{5}$$

where $C(x)$ and $C(y)$ represents the number of bits needed to lossless compress x and y, respectively. The $C(x, y)$ represents the number of bits needed to compress x and y conjointly, which usually is approximated by string concatenation.

Since in our application, we are required to compute the whole distances of each x^i according to a reference y, then an array of distances is calculated for creating the NCD profile according to

$$\mathcal{D}_i(x^i, y) = \frac{C(x^i, y) - \min\{C(x^i), C(y)\}}{\max\{C(x^i), C(y)\}}. \tag{6}$$

Moreover, because the conjoint compression follows the commutative property $C(x^i, y) = C(y, x^i)$, it can be rewritten as

$$\mathcal{D}_i(x^i, y) = \frac{C(y, x^i) - \min\{C(x^i), C(y)\}}{\max\{C(x^i), C(y)\}}. \tag{7}$$

This change offers a substantial save in computational resources because it is now possible to compress y and, in the end, freeze its models. Then, the number of bits to compress y is saved, and for each x^i, the compression models are initialized with the frozen models of y. Therefore the complexity time to compute $C(y, x^1), ..., C(y, x^n)$ is now $y + xn$. This change is now affordable for an application considering millions of genomes.

The choice of the data compressor is fundamental to better approximating the Kolmogorov complexity and the NCD [63, 58]. Therefore, besides respecting the common distance characteristics [63] and distance density [58], the data compressors that are designed for the specific use of certain types of data offer a much higher approximation of the Kolmogorov complexity than general-purpose tools. This characteristic is provided by the specific-purpose methodology's ability to efficiently model characteristics that would otherwise not be seen with general-purpose models. For example, two characteristics that play a key role in biological sequences are inverted repeats [64] and high-level substitutions in repetitive data [65].

In the case of the COVID-19 pandemic, a specific-purpose data compressor does not offer a wide advantage over a general-purpose data compressor because, on average, the sequences contain high entropy, are small, and have only a few sequence differences between sequences. However, an efficient specific-data compressor is mandatory for larger genomes such as those from larger viruses (e.g. Herpesvirus), bacterial or fungi. Therefore, we use an implementation derived from GeCo3 [66] and AC2 [62] data compressors that have proven to be state-of-the-art data compressors in genomic and proteomic sequences, respectively. The disadvantage of these data compressors is the necessity to have a solid knowledge of the models for extensive optimization. We provide pre-computed models for different biological sequence types to minimise this disadvantage.

For applications where the size of the distances vector is very large or the variation of the instances in the profile high, AltaiR offers the possibility of averaging the signal through a moving average. The Toolkit implementation subsection provides more information about this option.

Complexity (NC) Profiles

Another important temporal analysis is the perception of how the (normalized) Kolmogorov complexity [59] of a certain set of sequences sorted by time varies. The normalized Kolmogorov complexity is approximated through the Normalized Compression

(NC) [67]. The NC is given by the ratio of the uncompressed quantity sum of bits by the size of the sequence representation assuming a uniform distribution. This analysis is analogous to understanding how the genome or proteome sequence entropy varies over time using a random reference.

As in the previous Subsection, the Kolmogorov complexity is approximated using specific-purpose data compression algorithms, namely with implementations derived from GeCo3 [66] and AC2 [62] for genome and proteome sequences, respectively.

Formally, the NC of a certain sequence is provided by

$$\mathcal{E}(x) = \frac{C(x)}{|x| \log_2(|\Xi|)}, \quad (8)$$

where $|x|$ is the size of the sequence and $|\Xi|$ the number of different symbols in the sequence x.

Since in our application, we require to compute the whole sequence complexity of each x^i, then an array of NCs is calculated for creating the NC profile according to

$$\mathcal{E}_i(x^i) = \frac{C(x^i)}{|x^i| \log_2(|\Xi^i|)}, \quad (9)$$

where $|\Xi^i|$ is the number of different symbols in the sequence x^i.

For genomic sequences, Ξ is 4 for any x^i. However, for proteomic sequences, Ξ may vary, creating changes in the profile. For these cases, alphabet trimming, which involves reducing the alphabet size by removing or substituting non-standard characters, can work as a solution to ensure a consistent alphabet size across all sequences and maintain the comparability of NC values.

For applications where the size of the NC vector is very large or the variation of the instances in the profile high, AltaiR offers the possibility of averaging the signal through a moving average. The Toolkit implementation subsection provides more information about this option.

Frequency profiles

One application that provides evidence of evolutionary events is the variation of the nucleotide or amino acid frequency over time. This information is given by the frequency profiles, defined as the percentage symbol distribution of each sequence.

Formally, the frequency of a certain symbol, s, from Σ in a sequence x is provided by

$$\mathcal{F}(s|x) = \frac{1}{|x|} \sum_{j=1}^{|x|} \mathcal{I}_s(x_j), \quad (10)$$

where $\mathcal{I}$ is an indicator function associated to the number of times that s is seen in x.

Since in our application, we require to compute the symbol frequency of each x^i, then an array of frequencies is calculated for creating the frequency profile according to

$$\mathcal{F}_i(s|x^i) = \frac{1}{|x^i|} \sum_{j=1}^{|x^i|} \mathcal{I}_s(x^i_j). \quad (11)$$

For applications where the size of the frequency vector is very large or the variation of the instances in the profile high, AltaiR offers the possibility of averaging the signal through a moving average. The Toolkit implementation subsection provides more information about this option.

Relative singularity (RAWs) profiles

Identifying the shortest sequence regions present in a set of pathogens but absent from the host genome and transcriptome is an important application in endemic and epidemic contexts. These regions, called Relative Absent Words (RAWs), are of interest because they can distinguish between lineages [68], localize higher GC-content regions, and have applications in optimized diagnosis [69]. Furthermore, RAWs can provide insights into the unique genomic characteristics of pathogens, potentially aiding in the development of targeted therapeutic interventions and diagnostic tools [69].

Specifically, RAWs [68, 69, 70] are a particular subset of Minimal Absent Words (MAWs) that have also been referred to as nullomers or forbidden words [71, 72, 73]. Many MAWs-based applications have been created, including optimized models for their detection [74] and the complementary simulation of sequences while avoiding the creation of MAWs [75].

MAWs in genomic and proteomic sequences have been studied [76]. For example, in viruses, MAWs have been found most frequently in regions that are restriction recognition sites [76]. On the other hand, the subset of MAWs (RAWs) has been found in regions with high GC content, contrary to the host and pathogen genome [69].

An approach to finding MAWs is through the flanking subsequence regions. Consider a set, X, constituted of n target sequences, $x^1, x^{\cdots}, x^n$, and a reference sequence, y, both drawn from the finite alphabet Σ. We say that β is a factor of x^i if x^i can be expressed as $x^i = u\beta v$, with uv denoting the concatenation between sequences u and v. We denote by $\mathcal{W}_k(x^i)$ the set of all k-size words (or factors) of x^i. Also, we represent the set of all k-size words *not in* x^i as $\overline{\mathcal{W}_k(x^i)}$. For each word size k, we define the set of all words that exist in x^i but do not exist in y by

$$\mathcal{R}_k(x^i, \overline{y}) = \mathcal{W}_k(x^i) \cap \overline{\mathcal{W}_k(y)}. \quad (12)$$

The subset of minimal words as

$$\mathcal{M}_k(x^i, \overline{y}) = \{\beta \in \mathcal{R}_k(x^i, \overline{y}) : \mathcal{W}_{k-1}(\beta) \cap \mathcal{M}_{k-1}(x^i, \overline{y}) = \emptyset\}, \quad (13)$$

i.e., a MAW of size k cannot contain any MAW of size less than k. In particular, $l\beta r$ is a MAW of sequence x^i, where l and r are single letters from Σ, if $l\beta r$ is not a word of x^i but both $l\beta$ and βr are. We have defined the non-empty set $\mathcal{M}_k(x^i, \overline{y})$ with the smallest k as minimal Relative Absent Words (mRAWs) [68].

Another subset of MAWs and RAWs are persistent mRAWs (PmRAWs) [69]. Formally, let r be a mRAW of $x^i \in X$ and $P(r, x^i)$ be the predicate "r is a RAW of string x^i". Then, if $\forall_{x^i \in X} P(r, x^i)$, we say that r is persistent in X. This property means the full conservation of the identified mRAWs across all the sequences. A particularity of these sequences is to consider PmRAWs at the whole genome level and at a sub-genome level, meaning that a x^i can be considered a subsequence of a whole genome, namely a gene, extending the power of PmRAWs to local observations.

The RAWs and PmRAWs have been computed with the EAGLE tools version 1 [68] and 2 for DNA sequences [69]. This methodology was included in the online detection of RAWs, namely the ADACT [77].

The AltaiR toolkit can compute the RAWs based on the same methodology as in EAGLE version 2. Still, it extends the capability to deal with any other types of sequences, for example, proteomes, as long as they respect the multi-FASTA format. Moreover, for computing the RAWs, the AltaiR toolkit can avoid writing into temporary files while maintaining low memory consumption. This capability enables the computation of RAWs in millions of genomes/proteomes while expending relatively low computational resources.

Moreover, the Altair toolkit enables the automatic computation

of the GC percentage for each RAW, providing the unprecedented ability to create large-scale studies with a higher divergence between the sequences.

Additionally, the AltaiR toolkit includes the capability to compute RAWs profiles. The RAWs profiles are generated with the additional combination of temporal metadata, namely through the time order usage of the target multi-FASTA file that can be downloaded with that property at the NCBI repository. These profiles directly compute the presence of each RAW according to the sequences sorted by temporal characterization.

Toolkit implementation

The AltaiR methodology is implemented in C language and contains no external dependencies. The source code and application result scripts are freely provided at the repository [78]. The AltaiR toolkit contains one main menu (command: AltaiR) with six sub-menus for computing the methods that it provides, sometimes through the combination of multiple, namely

- **average** - moving average filter of a column float CSV file (the column to use is a parameter);
- **filter** - filters FASTA reads by characteristics: alphabet, completeness, length, GC quantity, multiple string patterns and anti-patterns;
- **frequency** - computes the alphabet frequencies for each FASTA read (it enables alphabet filtering);
- **nc** - computes the Normalized Compression (NC) for all FASTA reads according to a compression level or specific parameters;
- **ncd** - computes the Normalized Compression Distance (NCD) for all FASTA reads according to a reference;
- **raw** - Computes Relative Absent Words (RAWs) with automatic GC percentage estimation for all RAWs.

The toolkit allows reading and writing from standard input and output, respectively, in certain cases. For example, this feature enables the direct piping of the output from the filtering tool to the NC, NCD, and RAWs tools, streamlining the analysis process. Additionally, it allows for the output profiles of the frequency, NC, NCD, and RAWs tools to be easily processed by other tools, such as those performing averaging or visualization. This functionality provides easier integration to build custom pipelines. Furthermore, most tools (except average) use multi-threading to speed up the analysis.

In the next section (results), we provide details of the toolkit features, including the most important parameters and commands to retrieve the results. Nevertheless, more documentation can be retrieved from the Supplementary material and in the README.md file in the code repository.

Results

As a result, we provide the application analysis of the AltaiR toolkit to the challenges described in the previous section. The primary motivation for using the SARS-CoV-2 data is to demonstrate AltaiR's capabilities in handling large-scale genomic datasets and uncover novel insights into the virus's evolution, adaptation, and interaction with the human host. All the results can be repeated using the procedures described in each specific analysis, except for the data collection retrieved manually from the NCBI Virus database. Despite this manual procedure, all the data is included as supplementary material for direct download. The dataset used to compute the results includes a large collection of SARS-CoV-2 genome sequences retrieved from the NCBI viral repository on 29 September 2022 [2]. The SARS-CoV-2 genomes have been filtered from a pool of 6,309,078 genomes according to quality and completeness, namely only considering complete genomes with the host as human, resulting in 1,538,095 genomes [79]. The dataset also contains multiple coronavirus genomes, the reference T2T human genome and transcriptome, and multiple computer-generated sequences; the latter works as an important validation procedure.

Filtering sequences

The SARS-CoV-2 dataset contains nearly 1.5 million sequences labelled as complete genomes in the NCBI viral repository. In the histogram depicted in Figure 2, we notice substantial differences in the length of the sequences. This histogram provides the frequency of the length of the sequences, which was computed considering symbols outside the alphabet $\Sigma = \{A, C, G, T\}$, such as the N symbols and others. Although the plot considers only the sequence length from 29.5k and 30k, there were several sequences with lengths lower than 20k. These smaller sequences were considered complete genomes and showcased the importance of filtering the data by the sequence length.

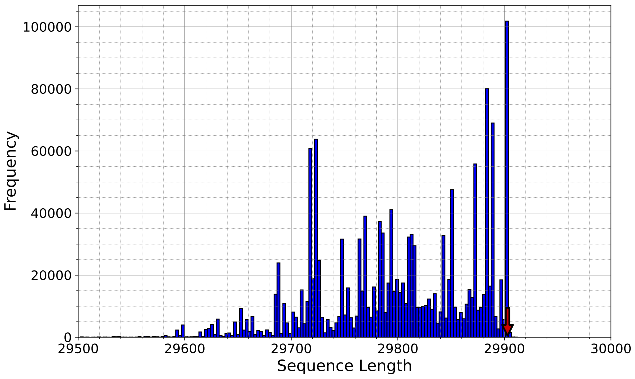

Figure 2. Histogram with the length of the genomes, considering all the existent symbols, from the SARS-CoV-2 dataset. The red arrow line stands for the size of the reference genome. To replicate this analysis, see Supplementary Section Reproducibility, specifically Reproducing the "Data filtering".

The SARS-CoV-2 reference genome (NC_045512.2) has a length of 29,903, and the largest cluster of sequences is almost coincident with this length (around 100k sequences). Therefore, since the SARS-CoV-2 genomes have low variability, this region is a good candidate for sequence length filtering.

However, the SARS-CoV-2 genomes contain a poly(A)-tale with a variable size in the end-tip. This poly(A)-tale is the only low-complexity region and, therefore, it is, ironically, the region more complex to sequence and assemble [69, 41]. To provide some flexibility to this region, we filtered the sequences considering the interval length of 29,885 to 29,921. Notice that many of these sequences contained symbols outside the $\{A, C, G, T\}$ alphabet, for example, N symbols, and, thus, many of them were removed when the total of alphabet symbols was not reached.

Moreover, we included filtering specific patterns and anti-patterns, namely considering only the sequences where the headers did not contain only the year (e.g. "|2020|") but also the month and, if available, the day associated with the virus isolation. Also, sequences without dates were discarded.

In sum, the sequence length and the pattern/anti-pattern decreased by 1.5 million to 25,594 sequences. To assess the impact of our filtering criteria on the representation of viral variants, we compared the distribution of the top 10 most represented variants before and after filtering. Our analysis shows that while the filtering process does reduce the total number of sequences from 1.5 million to 25,594, it maintains a consistent representation of the

major viral variants. The Pearson correlation (0.7348), Spearman correlation (0.7982), and cosine similarity (0.7940) between the before and after distributions indicate a strong positive correlation and overall similarity in variant representation. Figure 3 illustrates the variant distribution before and after filtering, demonstrating that the most prevalent variants remain well-represented in the filtered dataset. Although the dataset was reduced substantially, the data quality substantially increased proportionally. Moreover, nearly 25k of high-quality sequences are sufficient for accurate downstream analysis.

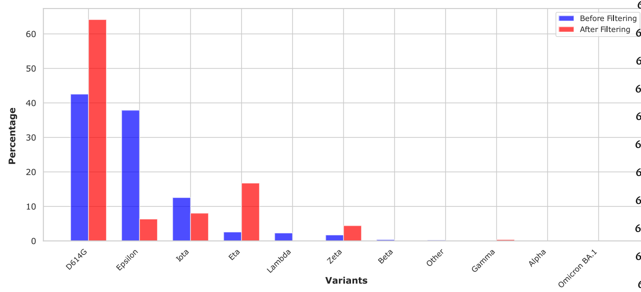

Figure 3. Variant representation before and after filtering.

This sequence filtering step shows its critical importance, proven by the high variability between these sequences, which can harm downstream analyses without proper filtering. For instance, including incomplete, low-quality, or outlier sequences can introduce noise and bias in similarity measurements, leading to inaccurate clustering or phylogenetic inference. Inconsistent or missing metadata can hinder the interpretation of temporal patterns and evolutionary trends, while unusual sequence characteristics can skew statistical analyses and obscure biological patterns. As such, rigorous filtering ensures high data quality and consistency, minimizing spurious results and enabling reliable biological interpretations.

Similarity (NCD) profiles application

To benchmark the similarity profiles described in the Subsection Similarity (NCD) profiles, we recurred to two levels, namely, using synthetic and real data.

The synthetic data (denoted as the original sequence) has been computer-generated with the GTO toolkit [35] while assuming a uniform distribution and a length of 5000 DNA symbols. Using this original sequence, consecutive SNP mutations have been applied to the following sequences with a symbol mutation probability of 0.00005. To clarify, the original sequence (time-0) has been mutated while originating the sequence time-1, then the sequence time-1 has been mutated originating the sequence time-2, and this process was followed to the sequence time-10000. Finally, the temporal similarity profiles have been computed using a chosen time point as a reference. Supplementary Figure 1 provides the similarity (NCD) profiles for three time points, namely 0, 2500, and 4000.As depicted, the NCD profiles have a minimum value for the respective time point reference, showing the capability to identify the closest sequence in time under these simplified conditions.

Despite the performance of the previous approach, it is hard to find a real scenario where a time point sequence is perfectly available. In practice, this sequence already contains several mutations. Therefore, to simulate this characteristic, we repeat the above experience, but instead of using a direct time point sequence, we use a mutated time point sequence.

Accordingly, all the time point symbol sequences have been mutated with a probability of 0.05 (mutations in approximately 5% of the sequence) using a uniform distribution and different seeds. Supplementary Figure S2 provides the similarity (NCD) profiles for three-time points mutated, namely 0, 2500, and 4000. Although the average NCD value increased, the similarity is still comparatively high for the selected points. These results suggest that NCD profiles may serve as tolerant mutation measures to predict the temporal occurrence of sequences, but further investigation is required to validate this claim.

In addition to the synthetic data analysis, we also explored the application of NCD profiles to a real-world dataset, specifically focusing on the RaTG13 genome sequence and its similarity to SARS-CoV-2 sequences. This analysis, along with a discussion of its limitations and potential implications, can be found in the Supplementary Material. Applying NCD profiles to this real-world dataset demonstrates their ability to uncover interesting trends and relationships that can guide further research. While the results obtained from NCD profiles should be interpreted cautiously and validated through additional lines of evidence, they serve as a valuable starting point for more focused investigations and hypothesis generation.

It's also worth mentioning that from any NCD results, one can automatically construct a phylogenetic tree for the n closest sequences. The Supplementary Material under the Reproducibility Section illustrates an example of this tree, along with instructions for recreating it.

Complexity profiles (NC) application

Herein, we present a detailed complexity profile (NC) analysis of SARS-CoV-2 sequences using the AltaiR toolkit. By approximating the normalized Kolmogorov complexity through the Normalized Compression metric, we quantitatively assessed the informational content of the viral sequences over time.

The complexity profile, illustrated in Figure 4, captures the temporal fluctuations in the sequence complexity of the virus. The NC values were computed using 5, 20, and 100 window sizes to smooth the data and reveal underlying trends. The plot shows the NC trajectory, where each line represents the complexity calculated over a different window size.

The time frame from January 2020 to January 2022 was particularly interesting due to significant epidemiological events. Our analysis encapsulates this period, showing a dynamic yet subtle evolution of the virus's genomic makeup, namely an average decrease of sequence complexity over time. Usually, to accomplish very similar functions, lower entropy corresponds to less energy required and, hence, more efficiency which can be driven by evolution or selection.

Moreover, the plot indicates periods of relative stability interspersed with spikes and dips in complexity. Notice that some noise exists that can be related to the higher variability of the size of some sequences but that is contained in the filtered interval. On the other hand, some inflexion points may correlate with the emergence of new variants or adaptations in the virus's evolutionary strategy.

Recently, we introduced AlcoR [41]. AlcoR is a mapping and visualization tool for detecting low-complexity regions in biological data. AltaiR complements AlcoR in the sequence complexity analysis, namely by adding the capability to relate sequences using a temporal dimension.

Frequency profiles application

We used the filtered SARS-CoV-2 multi-FASTA to access the frequency application and computed its nucleotide variation over time, as shown in Figure 5.

The figure shows a notable shift in the nucleotide composition of SARS-CoV-2 over the observed period of time. Specifically, the frequency of thymine (T) bases has gradually increased, while there has been a (approximate) corresponding decrease in the frequency

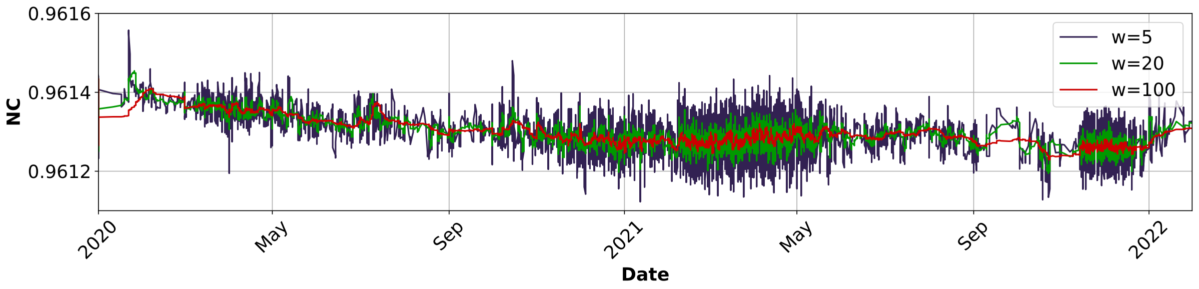

Figure 4. Complexity Plot showing variations in the normalized complexity (NC) values over time. The plot illustrates changes in NC with different window sizes, providing insights into the data trends from 2020 to 2022. To replicate this analysis, see Supplementary Section Reproducibility, specifically Reproducing the "Complexity profiles (NC) application"

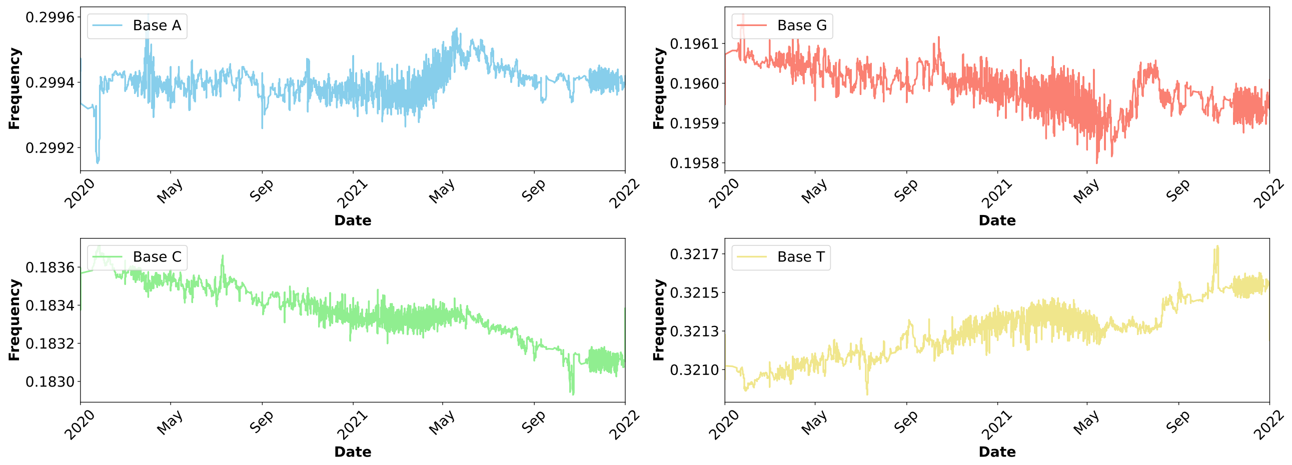

Figure 5. Frequency distribution of nucleotide bases in SARS-CoV-2 from 2020 to 2022, illustrating an increase in thymine (T) bases and a decrease in cytosine (C) bases. To replicate this analysis, see Supplementary Section Reproducibility, specifically Reproducing the "Frequency profiles application"

of cytosine (C) bases. This trend indicates the virus's ongoing evolutionary process within the human host.

The increase in T bases and the decrease in C bases can be attributed to various factors. Transition mutations, where a purine is substituted for another purine or a pyrimidine for another pyrimidine (in this case, C to T), are common mutations in RNA viruses. These mutations may be driven by the error-prone nature of RNA-dependent RNA polymerase, which is responsible for viral replication [80, 81, 82]. Additionally, such mutations may confer selective advantages to the virus, potentially impacting its transmissibility, pathogenicity, and immune escape capabilities [83, 84].

This trend also raises important considerations for public health and clinical interventions. For instance, changes in the viral genome could affect the efficacy of vaccines and therapeutics, emphasizing the need for continuous surveillance and adaptation of these measures [85, 86]. The observed mutations might also provide insights into the virus-host interaction dynamics, explaining how SARS-CoV-2 adapts to the human host environment over time [87].

Relative singularity (RAWs) profiles

Our study also focused on uncovering the Relative Absent Words (RAWs) of SARS-CoV-2, namely by identifying the shortest words that exist in the SARS-CoV-2 and were absent from the human host genome [88] and transcriptome [89]. Detailed statistics of this analysis are presented in Table 1.

When analyzing the data in the table, it becomes evident that there is a notable shift in nucleotide distribution as the mRAW size increases in the SARS-CoV-2 genome. Specifically, we observe a decrease in guanine (G) and cytosine (C) percentages in larger mRAWs, indicating a declining prevalence of GC content as the sequence size expands. This trend suggests a gradual shift towards AT-rich sequences in the larger mRAWs of the virus.

Interestingly, this finding contrasts with the overall nucleotide composition trend observed in SARS-CoV-2, where globally, GC content approximates 40%, and AT content around 60%. This discrepancy between the overall nucleotide composition of SARS-CoV-2 and the elevated GC percentage in the shortest unique sequences absent from the human genome suggests distinct evolutionary adaptations or functional requirements in the viral genome. Higher GC content in these sequences may confer increased structural stability or efficiency in replication and transcription processes, potentially impacting the virus's interaction with host cellular mechanisms. This pattern may reflect evolutionary pressures shaping these specific genomic regions for optimized functionality within the host environment [90, 91, 92].

On the other hand, from a previous study [69] we found out that some of the mRAWs with high GC content were localized at the surface of important proteins, such as the Spike glycoprotein in the SARS-CoV-2. This region was then found to be related to segments where the protein presents higher dynamics in time, namely higher movement of the protein to interact with the host cell.

Figure 6 and Supplementary Figure S4, through relative singularity profiles, illustrate temporal variations in both the count of mRAWs and their average GC-content in the SARS-CoV-2 genomes. Between June and August 2021, the plots indicate significant shifts in the SARS-CoV-2 genome's mRAWs count and GC content, coinciding with the Delta variant's dominance. The unique mutations of the Delta variant likely drove these genomic changes, reflecting its impact on the virus's nucleotide composition and structure.

The AltaiR tool's efficiency in processing large datasets proves

Table 1. Output statistics of AltaiR while computing the RAWs of SARS-CoV-2 that are absent from the human genome and transcriptome.

	Overall mRAWs Statistics			mRAWs Nucleotide Distribution				mRAWs AT/CG Distribution	
**kmer**	**Average**	**Variance**	**Std. Dev.**	**A**	**C**	**G**	**T**	**AT%**	**CG%**
11	0.00	0.00	0.01	3	3	4	1	36.4%	63.6%
12	8.29	0.39	0.62	584,523	561,473	720,587	679,229	49.6%	50.4%
13	119.70	11.26	3.36	8,500,431	10,168,609	11,774,411	9,386,300	44.9%	55.1%
14	726.01	46.92	6.85	60,566,946	65,041,615	67,333,051	67,209,832	49.1%	50.9%
15	2813.56	147.05	12.13	269,913,035	251,842,372	260,427,726	298,014,612	52.6%	47.4%
16	8132.19	227.32	15.08	900,499,378	717,952,376	740,116,031	971,728,183	56.2%	43.8%

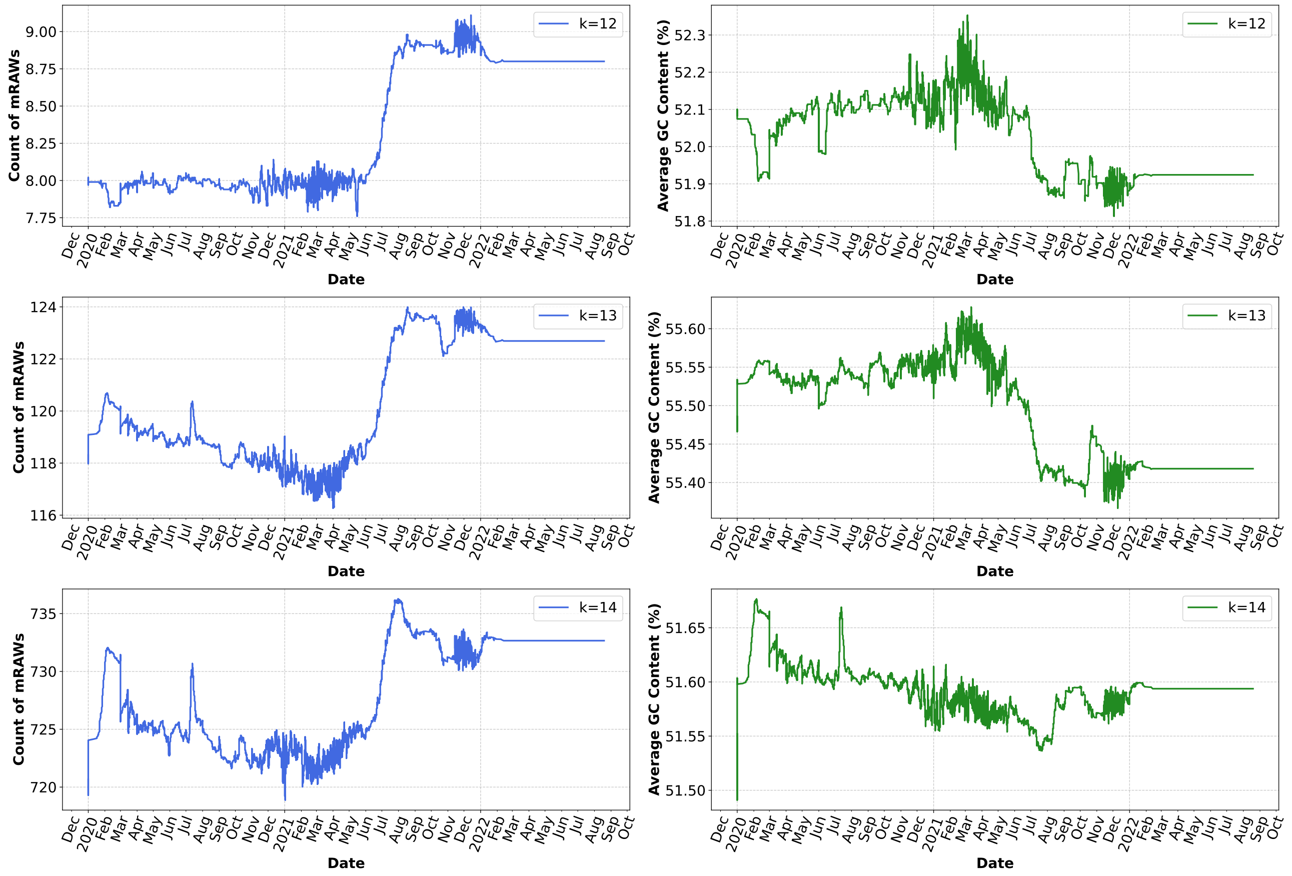

Figure 6. Relative singularity (RAWs) profiles: This set of plots illustrates the variation over time in the number of mRAWs and the average GC content in SARS-CoV-2 sequences. Each subplot corresponds to a different k-mer size (k=12, 13 and 14), showcasing the distribution of mRAWs and GC percentage across various time points. The x-axis represents time, segmented into years and months, while the y-axis shows the count of mRAWs and the GC content percentage, respectively. To replicate this analysis, see Supplementary Section Reproducibility, specifically Reproducing the "Relative singularity (RAWs) profiles"

extremely useful for identifying virus evolutionary trends. Additionally, its capability to rapidly analyze and detect mRAWs holds significant potential for diagnostics, as these sequences can be targeted explicitly in tests for accurate and prompt detection of viral infection. Moreover, mRAWs profiles can be used as potential markers for detecting new variants.

Computational efficiency

To evaluate the computational performance of the AltaiR toolkit, we conducted tests using a system with an Intel(R) Xeon(R) CPU E5-2630 v4 @ 2.20GHz (12 cores, 12 threads), 31.39 GB of RAM, running Linux 5.4.0-190-generic. Using only a single computational thread, we measured the average and total execution times, as well as peak RAM usage for each of AltaiR's main methods: filtering, NC calculation, frequency calculation, NCD calculation, and RAWs calculation (Table 2).

Filtering sequences based on length, GC content, and specific patterns took 0.0004 seconds per sequence with a peak RAM usage of 3.27 MB. To further evaluate AltaiR's efficiency, we compared its filtering capabilities with an equivalent approach using traditional Unix tools (grep and awk). Both methods were tasked with filtering sequences based on length, nucleotide content (ACGT only), and specific header patterns. Using a dataset of SARS-CoV-2 sequences, AltaiR completed the filtering task in 598.435 seconds (0.0004 sec-

Table 2. Computational performance metrics for each method in the AltaiR toolkit.

Method	Time per sequence (seconds)	Total time (seconds)	Peak RAM (MB)
Filtering	0.0004	598.435	3.27
NC	0.005	128.2557	3.41
Frequency	0.0001	3.701	2.36
NCD	0.229	2294.132	168.57
RAWs	61.558	1538.950	258.77

onds per sequence), while the grep/awk approach took 720.381 seconds (0.000816 seconds per sequence). This demonstrates that AltaiR is approximately 5.55 times faster than the traditional Unix tools for this complex filtering task. The superior performance of AltaiR can be attributed to its optimized C implementation and specialized algorithms designed for processing genomic data.

NC calculation, which generates Nucleotide Complexity profiles, required an average of 0.0050 seconds per sequence (total 128.26 seconds) with a peak RAM usage of 3.41 MB. Frequency calculation, analyzing nucleotide composition, was the fastest at 0.0001 seconds per sequence on average (total 3.701 seconds) and used the least amount of RAM at 2.36 MB peak usage. NCD calculation took an average of 0.2294 seconds per sequence (total 2294.132 seconds) and required 168.57 MB of RAM at its peak. RAWs calculation was the most computationally intensive method, requiring an average of 61.5580 seconds per sequence and 1538.95 seconds for the entire dataset, with the highest peak RAM usage of 258.77 MB. This increased resource usage is expected, as the RAWs method involves complex string matching and comparison operations to identify unique substrings.

The modular design of AltaiR allows for the integration of these methods into customizable pipelines, enabling researchers to select the most relevant analyses for their specific studies. The toolkit's ability to efficiently process large datasets, with most methods requiring less than a second per sequence on average and relatively low RAM usage, makes it well-suited for the rapidly growing field of genomic data analysis. Even the most resource-intensive method, RAWs calculation, uses less than 260 MB of RAM, making AltaiR accessible on standard desktop or laptop computers without the need for specialized high-performance computing resources.

These results showcase the overall computational efficiency of AltaiR, demonstrating its potential to streamline the analysis of large-scale genomic datasets across various research domains, from infectious disease studies to broader investigations in biology and genetics, while maintaining modest hardware requirements.

Discussion

The development of the AltaiR toolkit responds to the growing need for advanced tools capable of analyzing large and complex genomic datasets. This need arises not only from studies on infectious diseases but also from the broader fields of biology and genetics, where researchers are increasingly focusing on diverse organisms, including viruses, bacteria, protozoa, plants, and eukaryotes.

To contextualize AltaiR's capabilities, we performed a qualitative analysis with established toolkits—SeqAn [32], khmer (RRID:SCR_001156) [33, 34], GTO [35], HaploCoV [93], and bjorn [94]. Table 3 shows the results of this comparison focusing on key functionalities, efficiency metrics, and capabilities crucial for handling large-scale genomic data.

AltaiR's implementation in C allows for efficient execution and memory management, making it well-suited for handling large genomic datasets. Its support for multi-threading enables parallel computing, resulting in faster processing times compared to toolkits like khmer, HaploCoV [93, 95], and bjorn [94], which lack built-in parallel computing capabilities. AltaiR's strength lies in its alignment-free approach, which is particularly effective for processing large-scale genomic data. While alignment-free methods offer significant advantages in terms of computational efficiency and the ability to handle large-scale genomic datasets, they may not provide the same level of detailed information as alignment-based methods. Researchers should consider the trade-offs between computational efficiency and the depth of information required for their specific research questions when choosing between these approaches.

One of AltaiR's core strengths is its focus on alignment-free analysis methods, such as Normalized Compression Distance (NCD) profiles and Nucleotide Complexity (NC) profiles. These techniques enable efficient and scalable comparisons of genomic sequences without relying on computationally expensive alignment operations, setting it apart from toolkits like SeqAn, which primarily concentrates on alignment algorithms. It also differs from tools like GTO in that, while GTO possesses some alignment-free methods such as sequence compression and can be adapted to compute complexity profiles, it is not primarily designed for comprehensive alignment-free analysis. On the other hand, AltaiR's use of NCD for constructing similarity profiles enables a fast and detailed exploration of temporal patterns in genomic data, providing insights into the genomic trends of organisms over time. Additionally, AltaiR includes a feature for generating complexity profiles using NC, which is essential for quantitatively assessing the complexity of genomic sequences over time, providing insights into evolutionary pressures and adaptive responses in different organisms.

AltaiR's ability to filter sequences rigorously was demonstrated in the analysis of the SARS-CoV-2 dataset. It allows users to filter sequences based on length, GC content, and specific patterns, offering more comprehensive and customizable functionality compared to the filtering capabilities provided by SeqAn, khmer, and GTO, while HaploCoV and bjorn do not provide explicit filtering options. This advanced filtering is particularly useful for processing large-scale genomic datasets, where precise data selection is crucial for downstream analyses. For instance, AltaiR's ability to filter based on GC content can be invaluable in studies focusing on organisms with specific genomic compositions or in identifying potential contamination in sequencing data. While SeqAn and GTO offer basic filtering based on sequence length and simple pattern matching, and khmer provides k-mer-based filtering, AltaiR extends these capabilities by allowing for sophisticated filtering based on multiple criteria simultaneously. Like SeqAn, khmer, and GTO, AltaiR performs nucleotide frequency analysis. However, it extends this functionality with its unique NCD profile and RAW (Relative Absent Words) identification capability, enabling the detection of rare and atypical words within genomic sequences, a feature not found in the other toolkits. The frequency profile feature, which tracks changes in nucleotide composition, can be used to study molecular adaptations in various organisms and understand their evolutionary biology. AltaiR is optimized for processing millions of sequences efficiently, specifically targeting alignment-free methods. While SeqAn and GTO also handle large datasets, khmer focuses on k-mer analysis, and HaploCoV [93] and bjorn [94] are designed specifically for SARS-CoV-2 datasets. AltaiR's optimization is tailored towards its specific strengths, following a modular toolkit design that allows for flexibility and adaptability in genomic data analysis workflows, similar to SeqAn and GTO. HaploCoV and bjorn are specialized tools for analyzing SARS-CoV-2 datasets, with HaploCoV focusing on haplotype reconstruction and bjorn providing lineage assignment capabilities. These specialized tools complement the broader functionality offered by AltaiR and the other general-purpose toolkits.

A unique capability of AltaiR is its ability to identify RAWs in genomic and proteomic sequences. By comparing RAWs in pathogens with those in host genomes, AltaiR can uncover distinctive genomic and proteomic elements, with potential applications in pathogen-host interaction studies and the discovery of new genomic and proteomic markers for diagnostic and therapeutic purposes, such as through the combination with aptamers. Aptamers, consisting of short sequences of DNA, RNA, or peptides, serve as molecular tools capable of binding to specific target molecules or families of target molecules. They can modulate the function of specific proteins, influencing signalling pathways or exerting inhibitory or enhancing effects [96]. Notably, aptamers have shown promise in therapeutic applications, as highlighted by Keefe et al. [97]. To discover high-affinity aptamers, contemporary computational methodologies leverage deep learning in conjunction with relevant feature extraction techniques, as Emami et al. [98]. The untapped potential of using mRAWs as pertinent features in aptamer discovery

Feature / Toolkit	AltaiR	SeqAn	khmer	GTO	HaploCoV	bjorn
**Programming Language**	C	C++	Python, C++	C	Perl	Python, Bash
**Parallel Computing**	✓(Multi-threading)	✓	✗	✓	✗	✗
**Alignment-Free Analysis**	✓	✗	✓	✓✗(Partial, compression)	✗	✗
**Genome Filtering**	Advanced (by length, GC content, patterns)	Basic (sequence length, simple pattern matching)	Basic (k-mer-based filtering)	Basic (sequence length, simple pattern matching)	✗	✗
**NCD Profile**	✓	✗	✗	✓✗(with scripting alterations)	✗	✗
**Complexity Profiles (NC)**	✓	✗	✗	✓	✗	✗
**Nucleotide Frequency Analysis**	✓	✓	✓✗(k-mer frequencies)	✓	✗	✗
**RAW Identification**	✓	✗	✗	✗	✗	✗
**Large-Scale Data Handling**	Optimized for millions of sequences	Large data handling but less optimized	Designed for large datasets but focused on k-mer analysis	✗	Designed for large SARS-CoV-2 datasets	Designed for SARS-CoV-2 datasets
**External Dependencies**	None	Some libraries required	Python environment and dependencies	None (if not considering the external tools)	Perl modules	Python package, Docker
**Modular Toolkit Design**	✓	✓	✗	✓	✗	✗
**FASTA Format Support**	✓	✓	✓	✓	✓	✓
**K-mer Counting and Filtering**	✗	✗	✓	✗	✗	✗
**String Matching Algorithms**	✗	✓	✗	✗	✗	✗
**Haplotype Reconstruction**	✗	✗	✗	✗	✓	✗
**Lineage Assignment**	✗	✗	✗	✗	✗	✓

Table 3. Comparative analysis of AltaiR with SeqAn, khmer, GTO, HaploCoV, and bjorn.

remains uncertain, but their distinctive characteristics suggest the possibility of streamlining subsequent phases of drug discovery and bypassing certain hurdles in the quest for novel therapeutic agents.

The versatility of AltaiR's sub-tools, coupled with its programming efficiency, holds significant importance for seamless integration into intricate pipelines involving multiple tools. This applicability is particularly noteworthy in various domains, such as drug discovery, viral genome reconstruction and analysis, and diversity analysis [99, 100, 101].

Consequentially, AltaiR stands out as a comprehensive and efficient toolkit for alignment-free analysis of large-scale genomic datasets. Its unique features, optimization for handling millions of sequences, and broad applicability position it as a powerful resource for a wide range of genomic and proteomic research endeavours, complementing the functionalities offered by other established toolkits and specialized tools.

Conclusions

In this study, we introduced AltaiR, a versatile and robust toolkit designed for the advanced analysis of large-scale genomic datasets. AltaiR's alignment-free methodology efficiently manages extensive data, exemplified by its role in analyzing a large number of SARS-CoV-2 sequences. The toolkit's capabilities, including Normalized Compression Distance (NCD) profiling, Normalized Compression (NC) analysis, temporal nucleotide composition variation, and the identification of Relative Absent Words (RAWs), showcase its adaptability to diverse genomic data types and research requirements.

AltaiR's innovation lies in the integration of well-established filtering methods, such as sequence length, GC content, and pattern matching, with novel and enhanced features into a comprehensive framework designed for efficient, large-scale genomic data analysis. The toolkit introduces new methods, including the Frequency method for detailed nucleotide or amino acid occurrence analysis, and extends the capabilities of the Filter method by supporting multiple simultaneous pattern searches, absent pattern searches, and filtering by GC content, sequence length, or completeness.

Moreover, AltaiR's NCD method introduces a novel approach that uses a conjunction of reference and target files while freezing the models of one and saving computation time for other sequences. This methodology allows for substantial time savings in NCD calculations. The NC analysis provides a quantitative assessment of the complexity of genomic sequences over time, offering insights into evolutionary pressures and adaptive responses in different organisms.

A unique capability of AltaiR is its ability to identify RAWs in genomic and proteomic sequences. The RAWs method in AltaiR supports genomic and protein sequences. By comparing RAWs in pathogens with those in host genomes, AltaiR can uncover distinctive genomic and proteomic elements, with potential applications in pathogen-host interaction studies and the discovery of new genomic and proteomic markers for diagnostic and therapeutic purposes.

The combination of these tools, along with the optimization for analyzing millions of sequences, ensures high portability and ease of installation while providing the necessary capabilities for rigorous analyses. This versatility enables a streamlined workflow, allowing researchers to process raw, unfiltered data and obtain meaningful insights without the need for multiple, disconnected tools.

AltaiR's application in studying SARS-CoV-2 genomes has provided possible insights into the virus's evolution and adaptations. However, the potential of AltaiR extends beyond virology, making it a crucial tool in broader genomic and proteomic research, such as in the analysis of resistant bacteria, unravelling complex temporal patterns, and facilitating studies on the evolution and diversity of various organisms.

As genomic research advances, driven by technological advancements and the increasing complexity of biological data, AltaiR's scalability, and efficiency position it as a powerful resource for a wide range of genomic and proteomic research.

Availability of source code and requirements

- Project name: AltaiR
- Project home page: https://github.com/cobilab/altair
- Operating system(s): Linux
- Programming language: C; Python; Bash
- Other requirements: Conda v4.3.27.
- License: GPL v3.
- RRID: SCR_024752
- Biotools: altair

Data Availability

An archival copy of the code and the SARS-CoV-2 sequence data used in this study is available via the GigaScience database, GigaDB [102]. The dataset includes the filtered SARS-CoV-2 sequences and the accession identifiers of all the SARS-CoV-2 Sequences input data. Additionally, links to supplementary sequences required for the analysis are provided.
The supplementary material file for this manuscript provides detailed reproducibility instructions, covering all steps for data analysis, tool usage, environment setup, script execution, and additional results. It includes scripts for filtering sequences, generating NCD profiles, conducting complexity and frequency analyses, and constructing phylogenetic trees.

Declarations

List of abbreviations

DNA - Deoxyribonucleic acid;
HSV-1 - Herpes Simplex Virus 1;
HSV-2 - Herpes Simplex Virus 2;
ID - Identifier;
MAW - minimal absent word;
mRAW - minimal Relative Absent Word;
PmRAW - Persistent minimal Relative Absent Word;
NC - Normalized Compression;
NCD - Normalized Compression Distance;
RAW - Relative Absent Word;
RNA - Ribonucleic acid;
T2T - Telomere-2-Telomere;
VZV - Varicella-Zoster Virus;

Competing Interests

The authors declare no competing interests.

Funding

This work was partially funded by National Funds through the FCT - Foundation for Science and Technology, in the context of the project UIDB/00127/2020. J.M.S. has received funding from the EC under grant agreement 101081813, Genomic Data Infrastructure. D.P. is funded by national funds through FCT – Fundação para a Ciência e a Tecnologia, I.P., under the Scientific Employment Stimulus - Institutional Call - reference CEECINST/00026/2018.

Acknowledgements

The authors wish to thank the Finnish Computing Competence Infrastructure (FCCI) for supporting this project with computational and data storage resources.

Author's Contributions

D.P. and A.P. designed the experiment. D.P. and J.M.S. coded the tools. J.M.S. executed the data analysis. J.M.S. and D.P. discussed the results. J.M.S. and D.P. wrote the manuscript. All authors have revised the manuscript.

References

1. Khare S, Gurry C, Freitas L, Schultz MB, Bach G, Diallo A, et al. GISAID's Role in Pandemic Response. China CDC Weekly 2021;3(49):1049.
2. Hatcher EL, Zhdanov SA, Bao Y, Blinkova O, Nawrocki EP, Ostapchuck Y, et al. Virus Variation Resource–improved response to emergent viral outbreaks. Nucleic acids research 2017;45(D1):D482–D490.
3. Cochrane G, Karsch-Mizrachi I, Takagi T, Sequence Database Collaboration IN. The international nucleotide sequence database collaboration. Nucleic acids research 2016;44(D1):D48–D50.
4. Sayers EW, Cavanaugh M, Clark K, Pruitt KD, Schoch CL, Sherry ST, et al. GenBank. Nucleic acids research 2021;49(D1):D92–D96.
5. Harrison PW, Ahamed A, Aslam R, Alako BT, Burgin J, Buso N, et al. The european nucleotide archive in 2020. Nucleic acids research 2021;49(D1):D82–D85.
6. Okido T, Kodama Y, Mashima J, Kosuge T, Fujisawa T, Ogasawara O. DNA Data Bank of Japan (DDBJ) update report 2021. Nucleic acids research 2022;50(D1):D102–D105.

7. de Vries JJ, Brown JR, Couto N, Beer M, Le Mercier P, Sidorov I, et al. Recommendations for the introduction of metagenomic next-generation sequencing in clinical virology, part II: bioinformatic analysis and reporting. Journal of Clinical Virology 2021;138:104812.
8. López-Labrador FX, Brown JR, Fischer N, Harvala H, Van Boheemen S, Cinek O, et al. Recommendations for the introduction of metagenomic high-throughput sequencing in clinical virology, part I: Wet lab procedure. Journal of Clinical Virology 2021;134:104691.
9. Simon-Loriere E, Holmes EC. Why do RNA viruses recombine? Nature Reviews Microbiology 2011;9(8):617–626.
10. Li X, Giorgi EE, Marichannegowda MH, Foley B, Xiao C, Kong XP, et al. Emergence of SARS-CoV-2 through recombination and strong purifying selection. Science advances 2020;6(27):eabb9153.
11. Palmenberg AC, Spiro D, Kuzmickas R, Wang S, Djikeng A, Rathe JA, et al. Sequencing and analyses of all known human rhinovirus genomes reveal structure and evolution. Science 2009;324(5923):55–59.
12. Plummer M, de Martel C, Vignat J, Ferlay J, Bray F, Franceschi S. Global burden of cancers attributable to infections in 2012: a synthetic analysis. The Lancet Global Health 2016;4(9):e609–e616.
13. Farrell PJ. Epstein–Barr virus and cancer. Annual Review of Pathology: Mechanisms of Disease 2019;14:29–53.
14. Smatti MK, Cyprian FS, Nasrallah GK, Al Thani AA, Almishal RO, Yassine HM. Viruses and autoimmunity: a review on the potential interaction and molecular mechanisms. Viruses 2019;11(8):762.
15. Pyöriä L, Pratas D, Toppinen M, Hedman K, Sajantila A, Perdomo MF. Unmasking the tissue-resident eukaryotic DNA virome in humans. Nucleic Acids Research 2023;51(7):3223–3239.
16. Toppinen M, Sajantila A, Pratas D, Hedman K, Perdomo MF. The Human Bone Marrow Is Host to the DNAs of Several Viruses. Frontiers in cellular and infection microbiology 2021;11:329.
17. Toppinen M, Pratas D, Väisänen E, Söderlund-Venermo M, Hedman K, Perdomo MF, et al. The landscape of persistent human DNA viruses in femoral bone. Forensic Science International: Genetics 2020;48:102353.
18. Land M, Hauser L, Jun SR, Nookaew I, Leuze MR, Ahn TH, et al. Insights from 20 years of bacterial genome sequencing. Functional & integrative genomics 2015;15(2):141–161.
19. Nurk S, Koren S, Rhie A, Rautiainen M, Bzikadze AV, Mikheenko A, et al. The complete sequence of a human genome. Science 2022;376(6588):44–53.
20. Qi W, Lim YW, Patrignani A, Schläpfer P, Bratus-Neuenschwander A, Grüter S, et al. The haplotype-resolved chromosome pairs of a heterozygous diploid African cassava cultivar reveal novel pan-genome and allele-specific transcriptome features. GigaScience 2022;11.
21. Mc Cartney AM, Shafin K, Alonge M, Bzikadze AV, Formenti G, Fungtammasan A, et al. Chasing perfection: validation and polishing strategies for telomere-to-telomere genome assemblies. Nature Methods 2022;p. 1–9.
22. Alkan C, Carbone L, Dennis M, Ernst J, Evrony G, Girirajan S, et al. Implications of the first complete human genome assembly. Genome Research 2022;32(4):595.
23. Vinga S, Almeida J. Alignment-free sequence comparison—a review. Bioinformatics 2003;19(4):513–523.
24. Reinert G, Chew D, Sun F, Waterman MS. Alignment-free sequence comparison (I): statistics and power. Journal of Computational Biology 2009;16(12):1615–1634.
25. Wan L, Reinert G, Sun F, Waterman MS. Alignment-free sequence comparison (II): theoretical power of comparison statistics. Journal of Computational Biology 2010;17(11):1467–1490.
26. Zielezinski A, Vinga S, Almeida J, Karlowski WM. Alignment-free sequence comparison: benefits, applications, and tools. Genome biology 2017;18(1):1–17.
27. Zielezinski A, Girgis HZ, Bernard G, Leimeister CA, Tang K, Dencker T, et al. Benchmarking of alignment-free sequence comparison methods. Genome biology 2019;20(1):1–18.
28. Metcalf JL, Xu ZZ, Bouslimani A, Dorrestein P, Carter DO, Knight R. Microbiome tools for forensic science. Trends in biotechnology 2017;35(9):814–823.
29. Chen Z, Zhao P, Li F, Marquez-Lago TT, Leier A, Revote J, et al. iLearn: an integrated platform and meta-learner for feature engineering, machine-learning analysis and modeling of DNA, RNA and protein sequence data. Briefings in bioinformatics 2020;21(3):1047–1057.
30. Yang J, Chen X, McDermaid A, Ma Q. DMINDA 2.0: integrated and systematic views of regulatory DNA motif identification and analyses. Bioinformatics 2017;33(16):2586–2588.
31. Min JL, Hemani G, Davey Smith G, Relton C, Suderman M. Meffil: efficient normalization and analysis of very large DNA methylation datasets. Bioinformatics 2018;34(23):3983–3989.
32. Döring A, Weese D, Rausch T, Reinert K. SeqAn an efficient, generic C++ library for sequence analysis. BMC bioinformatics 2008;9(1):1–9.
33. Crusoe MR, Alameldin HF, Awad S, Boucher E, Caldwell A, Cartwright R, et al. The khmer software package: enabling efficient nucleotide sequence analysis. F1000Research 2015;4.
34. Standage D, Aliyari A, Cohen LJ, Crusoe MR, Head T, Irber L, et al. khmer release v2.1: software for biological sequence analysis. Journal of Open Source Software 2017;2(15):272. https://doi.org/10.21105/joss.00272.
35. Almeida JR, Pinho AJ, Oliveira JL, Fajarda O, Pratas D. GTO: a toolkit to unify pipelines in genomic and proteomic research. SoftwareX 2020;12:100535.
36. Drezen E, Rizk G, Chikhi R, Deltel C, Lemaitre C, Peterlongo P, et al. GATB: genome assembly & analysis tool box. Bioinformatics 2014;30(20):2959–2961.
37. Lee J, Lee AJ, Lee JK, Park J, Kwon Y, Park S, et al. Mutalisk: a web-based somatic MUTation AnaLyIS toolKit for genomic, transcriptional and epigenomic signatures. Nucleic acids research 2018;46(W1):W102–W108.
38. Sims D, Ilott NE, Sansom SN, Sudbery IM, Johnson JS, Fawcett KA, et al. CGAT: computational genomics analysis toolkit. Bioinformatics 2014;30(9):1290–1291.
39. Hiltemann S, Mei H, de Hollander M, Palli I, van der Spek P, Jenster G, et al. CGtag: complete genomics toolkit and annotation in a cloud-based Galaxy. GigaScience 2014;3(1):2047–217X.
40. de Koning W, Miladi M, Hiltemann S, Heikema A, Hays JP, Flemming S, et al. NanoGalaxy: Nanopore long-read sequencing data analysis in Galaxy. GigaScience 2020;9(10):giaa105.
41. Silva JM, Qi W, Pinho AJ, Pratas D. AlcoR: alignment-free simulation, mapping, and visualization of low-complexity regions in biological data. GigaScience 2023;.
42. Loman NJ, Quinlan AR. Poretools: a toolkit for analyzing nanopore sequence data. Bioinformatics 2014;30(23):3399–3401.
43. Knight R, Maxwell P, Birmingham A, Carnes J, Caporaso JG, Easton BC, et al. PyCogent: a toolkit for making sense from sequence. Genome biology 2007;8(8):1–16.
44. Shen W, Le S, Li Y, Hu F. SeqKit: a cross-platform and ultrafast toolkit for FASTA/Q file manipulation. PloS one 2016;11(10):e0163962.
45. Alam KK, Chang JL, Burke DH. FASTAptamer: a bioinformatic toolkit for high-throughput sequence analysis of combinatorial selections. Molecular Therapy-Nucleic Acids 2015;4:e230.
46. Ott M, Edunov S, Baevski A, Fan A, Gross S, Ng N, et al. fairseq: A Fast, Extensible Toolkit for Sequence Modeling. In: Pro-

ceedings of the 2019 Conference of the North American Chapter of the Association for Computational Linguistics (Demonstrations) Minneapolis, Minnesota: Association for Computational Linguistics; 2019. p. 48–53. https://aclanthology.org/N19-4009.

47. Chen C, Chen H, Zhang Y, Thomas HR, Frank MH, He Y, et al. TBtools: an integrative toolkit developed for interactive analyses of big biological data. Molecular plant 2020;13(8):1194–1202.

48. Gabler F, Nam SZ, Till S, Mirdita M, Steinegger M, Söding J, et al. Protein sequence analysis using the MPI bioinformatics toolkit. Current Protocols in Bioinformatics 2020;72(1):e108.

49. Arkin AP, Cottingham RW, Henry CS, Harris NL, Stevens RL, Maslov S, et al. KBase: the United States department of energy systems biology knowledgebase. Nature biotechnology 2018;36(7):566–569.

50. Kryukov K, Ueda MT, Nakagawa S, Imanishi T. Nucleotide Archival Format (NAF) enables efficient lossless reference-free compression of DNA sequences. Bioinformatics 2019;35(19):3826–3828.

51. Deorowicz S, Danek A, Li H. AGC: compact representation of assembled genomes with fast queries and updates. Bioinformatics 2023;39(3):btad097.

52. Grabowski S, Kowalski TM. MBGC: Multiple Bacteria Genome Compressor. GigaScience 2022;11.

53. Pinho AJ, Pratas D. MFCompress: a compression tool for FASTA and multi-FASTA data. Bioinformatics 2014;30(1):117–118.

54. Hosseini M, Pratas D, Pinho AJ. Cryfa: a secure encryption tool for genomic data. Bioinformatics 2019;35(1):146–148.

55. Lu J, Salzberg SL. Removing contaminants from databases of draft genomes. PLoS computational biology 2018;14(6):e1006277.

56. Sajantila A, Editors' Pick: Contamination has always been the issue! BioMed Central; 2014.

57. Li M, Chen X, Li X, Ma B, Vitányi PM. The similarity metric. IEEE transactions on Information Theory 2004;50(12):3250–3264.

58. Li M, Vitányi P, et al. An introduction to Kolmogorov complexity and its applications, vol. 3. Springer; 2008.

59. Kolmogorov AN. Three approaches to the quantitative definition of information. Problems of information transmission 1965;1(1):1–7.

60. Pratas D, Silva RM, Pinho AJ. Comparison of compression-based measures with application to the evolution of primate genomes. Entropy 2018;20(6):393.

61. Cilibrasi RL, Vitányi PM. Fast Phylogeny of SARS-CoV-2 by Compression. Entropy 2022;24(4):439.

62. Silva M, Pratas D, Pinho AJ. AC2: An Efficient Protein Sequence Compression Tool Using Artificial Neural Networks and Cache-Hash Models. Entropy 2021;23(5):530.

63. Cebrián M, Alfonseca M, Ortega A. Common pitfalls using the normalized compression distance: What to watch out for in a compressor. Communications in Information & Systems 2005;5(4):367–384.

64. Hosseini M, Pratas D, Pinho AJ. On the role of inverted repeats in DNA sequence similarity. In: International Conference on Practical Applications of Computational Biology & Bioinformatics Springer; 2017. p. 228–236.

65. Pratas D, Hosseini M, Pinho AJ. Substitutional tolerant Markov models for relative compression of DNA sequences. In: International Conference on Practical Applications of Computational Biology & Bioinformatics Springer; 2017. p. 265–272.

66. Silva M, Pratas D, Pinho AJ. Efficient DNA sequence compression with neural networks. GigaScience 2020;9(11):giaa119.

67. Pratas D, Pinho AJ. On the approximation of the Kolmogorov complexity for DNA sequences. In: Iberian Conference on Pattern Recognition and Image Analysis Springer; 2017. p. 259–266.

68. Silva RM, Pratas D, Castro L, Pinho AJ, Ferreira PJ. Three minimal sequences found in Ebola virus genomes and absent from human DNA. Bioinformatics 2015;31(15):2421–2425.

69. Pratas D, Silva JM. Persistent minimal sequences of SARS-CoV-2. Bioinformatics 2020 07;36(21):5129–5132.

70. Pratas D. Compression and analysis of genomic data. PhD thesis, Universidade de Aveiro (Portugal); 2016.

71. Béal MP, Mignosi F, Restivo A. Minimal forbidden words and symbolic dynamics. In: Annual Symposium on Theoretical Aspects of Computer Science Springer; 1996. p. 555–566.

72. Crochemore M, Mignosi F, Restivo A. Automata and Forbidden Words. Inf Process Lett 1998 Aug;67(3):111–117.

73. Pinho AJ, Ferreira PJ, Garcia SP, Rodrigues JM. On finding minimal absent words. BMC bioinformatics 2009;10(1):1–11.

74. Kosche M, Koß T, Manea F, Siemer S. Absent subsequences in words. In: International Conference on Reachability Problems Springer; 2021. p. 115–131.

75. Bernardini G, Marchetti-Spaccamela A, Pissis SP, Stougie L, Sweering M. Constructing strings avoiding forbidden substrings. In: 32nd Annual Symposium on Combinatorial Pattern Matching (CPM 2021), vol. 191; 2021. p. 1–9.

76. Koulouras G, Frith MC. Significant non-existence of sequences in genomes and proteomes. Nucleic acids research 2021;49(6):3139–3155.

77. Akon M, Akon M, Kabir M, Rahman MS, Rahman MS. ADACT: a tool for analysing (dis) similarity among nucleotide and protein sequences using minimal and relative absent words. Bioinformatics 2021;37(10):1468–1470.

78. cobilab, AltaiR: a C toolkit for alignment-free and temporal analysis of multi-FASTA data. cobilab; 2024. https://github.com/cobilab/altair, [Online; accessed 11-October-2024].

79. NCBI, NCBI Virus. NCBI; 2023. https://www.ncbi.nlm.nih.gov/labs/virus/vssi/#/virus?SeqType_s=Nucleotide&VirusLineage_ss=Wuhan%20seafood%20market%20pneumonia%20virus,%20taxid:2697049&Completeness_s=complete&HostLineage_ss=Homo%20sapiens%20(human),%20taxid:9606&CreateDate_dt=2000-01-01T00:00:00.00Z%20TO%202022-09-28T23:59:59.00Z, [Online; accessed 27-November-2023].

80. Singh D, Yi SV. On the origin and evolution of SARS-CoV-2. Experimental & Molecular Medicine 2021;53(4):537–547.

81. Drake JW, Holland JJ. Mutation rates among RNA viruses. Proceedings of the National Academy of Sciences 1999;96(24):13910–13913.

82. Sanjuán R, Nebot MR, Chirico N, Mansky LM, Belshaw R. Viral mutation rates. Journal of virology 2010;84(19):9733–9748.

83. Grubaugh ND, Hanage WP, Rasmussen AL. Making sense of mutation: what D614G means for the COVID-19 pandemic remains unclear. Cell 2020;182(4):794–795.

84. Harvey WT, Carabelli AM, Jackson B, Gupta RK, Thomson EC, Harrison EM, et al. SARS-CoV-2 variants, spike mutations and immune escape. Nature Reviews Microbiology 2021;19(7):409–424.

85. Korber B, Fischer WM, Gnanakaran S, Yoon H, Theiler J, Abfalterer W, et al. Tracking changes in SARS-CoV-2 spike: evidence that D614G increases infectivity of the COVID-19 virus. Cell 2020;182(4):812–827.

86. Plante JA, Liu Y, Liu J, Xia H, Johnson BA, Lokugamage KG, et al. Spike mutation D614G alters SARS-CoV-2 fitness. Nature 2021;592(7852):116–121.

87. Walls AC, Park YJ, Tortorici MA, Wall A, McGuire AT, Veesler D. Structure, function, and antigenicity of the SARS-CoV-2 spike glycoprotein. Cell 2020;181(2):281–292.

88. NCBI, Homo sapiens genome assembly T2T-CHM13v2.0. NCBI; 2023. https://www.ncbi.nlm.nih.gov/datasets/genome/GCF_009914755.1/, [Online; accessed 27-November-2023].

89. NCBI, Human Genome Resources at NCBI. NCBI; 2023. https://www.ncbi.nlm.nih.gov/genome/guide/human/, [Online; accessed 27-November-2023].
90. Wang Y, Mao JM, Wang GD, Luo ZP, Yang L, Yao Q, et al. Human SARS-CoV-2 has evolved to reduce CG dinucleotide in its open reading frames. Scientific Reports 2020;10(1):12331.
91. Takata MA, Gonçalves-Carneiro D, Zang TM, Soll SJ, York A, Blanco-Melo D, et al. CG dinucleotide suppression enables antiviral defence targeting non-self RNA. Nature 2017;550(7674):124–127.
92. Afrasiabi A, Alinejad-Rokny H, Khosh A, Rahnama M, Lovell N, Xu Z, et al. The low abundance of CpG in the SARS-CoV-2 genome is not an evolutionarily signature of ZAP. Scientific reports 2022;12(1):2420.
93. Chiara M, Horner DS, Ferrandi E, Gissi C, Pesole G. HaploCoV: unsupervised classification and rapid detection of novel emerging variants of SARS-CoV-2. Communications Biology 2023;6(1):443.
94. Lab A, Bjorn: Bioinformatics Pipeline for Analyzing SARS-CoV-2 Genomes;. Accessed: 2024-06-13. https://github.com/andersen-lab/bjorn.
95. Chiara M, HaploCoV: A Tool for Haplotype Analysis in SARS-CoV-2 Genomes;. Accessed: 2024-06-13. https://github.com/matteo14c/HaploCoV.
96. Crivianu-Gaita V, Thompson M. Aptamers, antibody scFv, and antibody Fab'fragments: An overview and comparison of three of the most versatile biosensor biorecognition elements. Biosensors and Bioelectronics 2016;85:32–45.
97. Keefe AD, Pai S, Ellington A. Aptamers as therapeutics. Nature reviews Drug discovery 2010;9(7):537–550.
98. Emami N, Ferdousi R. AptaNet as a deep learning approach for aptamer–protein interaction prediction. Scientific reports 2021;11(1):6074.
99. Herzig V, Cristofori-Armstrong B, Israel MR, Nixon SA, Vetter I, King GF. Animal toxins—Nature's evolutionary-refined toolkit for basic research and drug discovery. Biochemical pharmacology 2020;181:114096.
100. Pratas D, Toppinen M, Pyöriä L, Hedman K, Sajantila A, Perdomo MF. A hybrid pipeline for reconstruction and analysis of viral genomes at multi-organ level. GigaScience 2020;9(8):giaa086.
101. Adolfo LM, Rao X, Dixon RA. Identification of Pueraria spp. through DNA barcoding and comparative transcriptomics. BMC Plant Biology 2022;22(1):1–18.
102. Silva JM, Pinho AJ, Pratas D. Supporting data for "AltaiR: a C toolkit for alignment-free and temporal analysis of multi-FASTA data." GigaScience Database 2024. https://doi.org/10.5524/102587.

Click here to access/download
Supplementary Material
AltaiR_Supplementary_Material (1).pdf

Click here to access/download;Personal Cover;Altair_cover_letter_signed.pdf

Jorge Miguel Ferreira da Silva
IEETA, Aveiro University,Aveiro, Portugal
Jorge.miguel.ferreira.silva@ua.pt
+351 234 370 500
18/12/2023
Editor-in-Chief,
GigaScience

Dear GigaScience Editor,

I am writing to submit our manuscript titled "AltaiR: a C toolkit for alignment-free and spatial-temporal analysis of multi-FASTA data" for consideration for publication in GigaScience. This submission represents a significant contribution to the field of genomic and proteomic analysis, especially in the context of large-scale studies necessitated by recent global health challenges.

The current phase of genome sequencing, notably accelerated by the latest pandemics, has posed unique challenges in analyzing vast sets of viral genomes. Our toolkit, AltaiR, offers a novel solution to these challenges. It is specifically designed for analyzing large-scale numbers of genomes or proteomes, capable of handling millions of sequences in a single FASTA file without restrictions on size or number. This capability is crucial for contemporary genomic research and has wide-reaching implications for the scientific community.

A unique aspect of AltaiR is its alignment-free methodology, which enables the standalone identification of singularity and similarity patterns in sequences, alongside computing static and temporal dynamics. This feature not only enhances the toolkit's efficiency but also broadens its applicability across various research scenarios. In demonstrating its utility, we applied AltaiR to analyze the evolution of SARS-CoV-2 using over 1.5 million full sequences. This analysis revealed significant new insights into evolutionary patterns and characteristics of the virus, including trends in nucleotide composition and sequence complexity over time.

AltaiR's open availability, implemented in C language and optimized with multi-threading, ensures that it is readily accessible and usable by researchers worldwide. Its lack of external dependencies further adds to its practicality and ease of use. We believe that the innovative nature and the practical applications of our toolkit make it a valuable addition to GigaScience, appealing to a wide readership interested in genomic and proteomic analysis, especially in large-scale, data-intensive studies.

Thank you for considering our manuscript for publication. We are confident that our work aligns well with the scope and audience of GigaScience. Should you require any additional information or have any queries regarding our submission, please feel free to contact me.

Sincerely,

Assinado por: **JORGE MIGUEL FERREIRA DA SILVA**
Num. de Identificação: 13848089
Data: 2023.12.18 11:46:16 +0000

CHAVE MÓVEL
