# Supplementary material for: AltaiR: a C toolkit for alignment-free and temporal analysis of multi-FASTA data
Source: Gigascience. 2024 Nov 26;13:giae086. doi: 10.1093/gigascience/giae086 (PMC11590114; doi:10.1093/gigascience/giae086)
Supplement: giae086_Response_to_Reviewer_Comments_Original_Submission [file giae086_response_to_reviewer_comments_original_submission.pdf]

Author's Response To Reviewer Comments

Editor and Reviewer Comments:

We are grateful for the reviewer's positive appreciation of our work and the helpful comments and suggestions. We carefully considered the reviewers' comments and have revised the manuscript to address each comment. (please see if the response is not complete here, due to a system problem, I have attached the response as well as one of the supplementary materials).

Reviewer #1:

Summary

The authors present a toolkit (AltaiR) for analysing multiple sequences in multi-FASTA format that is exclusively characterized by alignment-free methodologies.

It computes:

-- Normalized Compression Distance profiling

-- Normalized Compression (complexity) analysis

-- temporal nucleotide composition variation

-- the identification of Relative Absent Words,

The manuscript is hard to read. However, the tool is well implemented and the GitRepo is user-friendly.

Response:

We appreciate the comments and hope the revised manuscript satisfies the reviewer.

Comments

Major

- The term "spatial-temporal" in the title is misleading because the method takes neither spatial nor temporal dependencies into account.

Response: Thank you for your observation. Firstly, we would like to clarify an oversight regarding the title; it mistakenly referred to "spacial-temporal" rather than the correct term "spatiotemporal." Secondly, we recognize the current title's phrasing as "spatial-temporal" might be misleading. In response, we updated the title and document to focus solely on the "temporal" dimension. Although our initial aim was to indicate the paper's capacity for both space and time analysis, we admit that the space analysis aspect is not fully flushed out in this paper. Specifically, the NCD and NC profiles are the most evident AltaiR functions that can serve to preform space analysis due to their ability to identify similar sequences, and complexity trends across different regions.

However, the paper's strength lies in its temporal analysis, especially evident in tracking pathogen behaviour over time, as demonstrated in the COVID-19 case study through effective metadata utilization and efficient scripting. As such, we will maintain the emphasis on temporal analysis in the title and document.

Update installation instructions:

The one on GitHub doesn't work instead:

mamba create -n altair -c conda-forge -c bioconda altair-mf

Response: We apologize for the oversight. We could not replicate the issue on our side, however, since this issue has occurred in the reviewer's environment, we added the command to the supplementary material of the paper and the GitHub repository.

Without comparison to other methods or toolkits, sentences like "ALtaiR is a significant advancement in the field" (see Discussion) don't seem appropriate. Therefore, it would be useful to include the following comparisons:

Please include a comparison (as a table?) to other toolkits that are mentioned in the introduction (SeqAn, khmer, GTO, etc) in terms of functionalities and efficiency (programming language, parallel computing)

It seems like one of the main advantages of this tool is its clever header filtering. The authors should provide a runtime comparison to the grep method.

Response: Thank you for the suggestion to include a comparison of AltaiR with other bioinformatics toolkits mentioned in the introduction. We agree that providing a comprehensive comparison of AltaiR's functionalities and efficiency concerning other state-of-the-art tools will strengthen the manuscript and help readers better understand AltaiR's unique features and advantages.

We have added to the discussion a detailed comparison of AltaiR with SeqAn, khmer, and GTO, and incorporated this comparison in a table that highlights key features such as programming language, parallel computing support, alignment-free analysis capabilities, genome filtering options, and other relevant functionalities.

The input requirements are not clear:

- the authors should specify explicitly for which pathogens the tool is suitable

Response: Thank you for the valuable feedback. The alignment-free methods and efficient handling of large, complex datasets make AltaiR well-suited for studying the genomic and proteomic sequences of these diverse pathogens. As such it can be used for analyzing a wide range of pathogens, including both small and large viruses (e.g., SARS-CoV-2, influenza viruses, herpesviruses), bacteria (e.g., Escherichia coli, Salmonella species), fungi (e.g., Candida albicans, Aspergillus fumigatus), and protozoa (e.g., Plasmodium species, Toxoplasma gondii).

- what is the minimum input AltaiR requires?

Response: Thank you for the question. The minimum input depends on the task the user desires to accomplish. Although this tool is developed to run with millions/thousands of sequences, you can run it for some tasks with one sequence (filtering, NC and frequency analysis); and for NCD and RAWs you only require 2. For NCD the user only requires a refence sequence to compare toward and another to compare. In the case of the RAWs computation, the process requires one as a reference (the sequence of the host: genome, transcriptome or both) and the genome of the pathogen.

- is the tool using the information of when the sequence was submitted/samples for the temporal mapping? How are these passed to the tool?

Response: Indeed. The tool is assuming order in the multi-fasta. Meaning the fasta provided to the tool is ordered by the analysis intended to be performed. The information of this order is obtained from the headers of the sequences, which have information regarding, date of submission, sample collection data, location, etc. In the case of this paper, we first retrieved the multi-fasta from NCBI with this information. Then, we ordered the sequences based on the header's information (date), after ordering the sequences were fed to AltaiR for analysis. For example, the NCBI viral database already provide this sorting feature.

- what is the minimum number of sequences you would recommend users to use?

Response: Regarding the minimum number of sequences recommended for analysis with AltaiR, we suggest using as many high-quality sequences as possible to obtain robust and meaningful results. The more sequences included in the analysis, the better the tool can capture the diversity and evolutionary patterns within the pathogen population.

However, the exact number of sequences required may vary depending on the specific research question and the stage of the analysis. For example, initial exploratory analyses may benefit from a larger, more inclusive dataset, while downstream analyses may focus on a smaller subset of high-quality, filtered sequences.

- how does the tool behave on substantially larger genomes?

Response: The tool is designed to scale efficiently with increasing sequence sizes. The time complexity of AltaiR's algorithms is approximately linear with respect to the input sequence size, making it highly scalable for analysing larger genomes, such as those of fungi, bacteria, and even eukaryotic organisms, without a significant increase in computational time.

- What is the overall runtime of your SARS-CoV-2 application? Can you put this in relation to other tools? This would give users a better understanding of what to expect.

Response: Directly comparing the runtime of our SARS-CoV-2 with other tools is challenging due to the unique set of features and functionalities offered by AltaiR. Our toolkit focuses on alignment-free methods, such as generating NCD profiles, and complexity profiles, and identifying RAWs, which are not commonly found in other tools. We have also added a section with the runtimes of the program to the paper, so it is possible to evaluate the execution time of the program in different scenarios.

The method section appears to be unnecessarily hard to read (concrete cases are listed in the minor comments section). It contains a lot of seemingly complex formulas for already established and well-known summary statistics (e.g. GC content, nucleotide-/amino acid frequency, etc.). Also, it is not described how AltaiR filters sequences by "completeness". Additionally, the manuscript would benefit from a clear overview figure.

Response: Thank you for your feedback. We have updated the manuscript to address your concerns:

We have already included an overview figure (Figure 1) that illustrates the workflow of the AltaiR toolkit, including the main methods and input types. This figure has been updated to provide a clear overview of the toolkit's functionality.

Regarding the formulas, while they may be well-known to some readers, we believe that including them improves the readability of the paper and allows a broader audience to understand the underlying concepts. The formulas serve as a reference for readers who may not be familiar with the specific methods and provide a clear mathematical foundation for the implemented techniques.

Completeness, in the context of genomic sequences, refers to the availability of the full, uninterrupted sequence of an organism's genome, rather than just partial or fragmented sequences. Filtering based on completeness ensures that only high-quality, contiguous sequences are included in the analysis, minimizing the potential bias introduced by incomplete data. To improve the readability of the manuscript this information was also updated in the manuscript.

Minor

- General: Missing line numbers impedes the reviewing process as it makes it harder to point out things while reading the manuscript.

Response: We thank the reviewer for this comment, in fact the lack of line numbers complicates the reviewing process. We now provide line numbers in the manuscript.

- abstract: Please define sequence complexity!

- abstract: can you use a more specific term than "flexible"?

Response: Thank you for your questions about the abstract. In this context, sequence complexity refers to the Kolmogorov complexity, which is a measure of the computational resources needed to specify an object, such as a sequence. It quantifies the amount of information contained within a sequence, with higher complexity indicating a greater degree of randomness or unpredictability, and lower complexity suggesting more repetitive or predictable patterns. In our study, we approximate the Kolmogorov complexity using compression-based methods.

Regarding the term "flexible", we used it to convey that AltaiR is highly parameterizable and can be easily integrated into various pipeline configurations. The toolkit's modular design allows users to customize the analysis based on their specific requirements and seamlessly combine AltaiR's tools with other bioinformatics software.

- p.2, Introduction: spatial-temporal characteristic: what are those characteristics? And where is the spatial or temporal data coming in?

Response: Thank you for your question. The spatio and temporal characteristics are the characteristics that arise from analysing these multi-fasta across a long period of time and across different countries. It is possible to order the sequences using headers' metadata (date of sequencing, region, etc) by doing so and analysing this information it is possible to extrapolate how the behaviour of the pathogen changes, across region and/ period of time.

- p.3, left column: what is "compact version of the data"? Be more precise compressed data? " to compact data." → is this compressed?

Response: Thank you. We have changed the text to compressed data.

- p.3, left column: "The workflow contains seven main methods" → are those all applied to all the three data inputs?

Response: Indeed, not all of the seven methods utilize the same inputs.
The methods that use only pathogen sequences (e.g. only a large multi-fasta) are filtering, NC computation and nucleotide sequence frequence computation. Some methods require additional sequences. NCD requires a reference sequence (the sequences which all other pathogenic sequences are compared to), whereas the RAWs requires the host omics (can be only the genome of the host, the transcriptome or both).
All profiles are created using the information from these computations (e.g. simple bash or python scripts), so these are derivative and do not require the source data per se.

- p.3, Figure 1: Maybe you can use different symbols for data and methods, and in general make clear which method is used for which data. What is the minimum input?

Response: Thank you for your feedback. The manuscript was updated to accommodate these changes in the image.

Can the input be sequences from different pathogens?

Response: Yes, it is possible in some cases that the input is a sequence for another host. A good example would be to see the similarity over time of a given virus towards another (using the NCD). This could be done as an example with another coronavirus as a reference and analyse the similarity towards sars-cov-2. Another example is in filtering, a user can accidentally place a sequence that does not belong in the mix of

the input and by filtering it would be very straightforward to remove such samples from the pool of sequences.

Which method is for which input data (maybe color-coding in the figure could help)

Response: Thank you for your feedback. The manuscript was updated to accommodate these changes in the image.

- p.3, right column: "The computation can be applied to different sequences;" → the word computation is irritating, this is a filtering step.

Response: Thank you, the text was updated.

- p.3, right column: "The computation can be applied to different sequences;"

→ is the essence of this and the following sentence that different filtering characteristics can be applied to different viruses, and they can be changed while the program is running? If so please, make it precise

Response: Thank you, for your comment. Although filtering is highly parameterizable, and you can apply different filtering characteristics to different virus, this is not possible to do while the program is running. It has to be defined prior to running the filtering step.

- p.3, right column: the phrase "intersected through a single run" is not clear. Are you allowed to change the filter criteria while running? this seems not in accordance with reproducibility

Response: Thank you, you are correct, it is not possible to change the filtering criteria while the program is running. What we meant to explain is that several filtering criteria can be applied at the same time (i.e. doing an AND). This was clearly not well explained and as such this paragraph was changed.

- p.3, right column: term "Alphabet composition" is not clear.

Response: By alphabet composition we mean the number of unique letters (and what letters) are contained in the sequence (e.g. in genomes it is usually ACGT, while for proteins are different amino acids).

- p.3, right column: "Alphabet composition": what you are doing with symbols that don't conform with the given alphabet, is the whole sequence excluded? Is only that symbol excluded, and does that influence the analysis?

Response: The filtering method is done to exclude sequences that are not in conform with the given alphabet. If the user wishes to still use sequences that have a given symbol, they must specify it and treat it priorly (e.g. sometimes sequences contain N symbol, an option is to include the sequence and afterwards run a script to change that symbol randomly to ACGT).

- p.4, left column: "Header patterns and anti-patterns"; it doesn't seem necessary to introduce the mathematical notation "yi", which is not used subsequently.

Response: We prefer to keep notation to provide a mathematical congruence to the text.

- p.4, left column, last paragraph: what is the "reference sequence"? How is it defined by the user and how should it be chosen?

Response: We apologize, in page 4, we did not find any mention of reference sequence only reference genome (which are used interchangeably). If we understood correctly, this section corresponds to the NCD. In this case the reference sequence is the sequence that is going to be compared towards all the other sequences in the multi-fasta. This sequence is defined by the user in the parametrization. Usually is a sequence that is not comprised in the multi-fasta (although it can be), and the objective is to assess the similarity, or “evolution/trend” of the fasta compared to an original sequence. The selection depends on

what the user desires to measure. For instance in sars-cov-2 it might be useful to assess similarity to other different virus that may originated it; in other scenarios such as multi-resistant bacteria, it might be useful to test (within the same species) the variation of a non-resistant strain towards another strains that have evolved resistance to bacteria in order to understand the evolution of this strain over time relatively to the original non-resistant strain.

- p.4, right column, mentions a time complexity of y + xn. It's not quite clear how the value of x is calculated. Does this somehow correspond to the previously mentioned number of bits to compress each individual x^i? If yes, how is this generalized to a single value of x?

Response: The time complexity mentioned, y + xn, represents the total time required to compute the NCD values for n target sequences (x^1, ..., x^n) against a single reference sequence y. Here, y represents the time needed to compress the reference sequence and freeze its compression models. This is done only once, regardless of the number of target sequences. x represents the average time needed to compress a single target sequence x^i, given the frozen compression models of the reference sequence y. Since the compression models for y are already available, compressing each target sequence x^i takes, on average, x units of time. Therefore, the total time complexity to compute the NCD values for all n target sequences against the reference sequence is y (for compressing and freezing the models of the reference sequence) plus x * n (for compressing each of the n target sequences using the frozen models of the reference sequence). The value of x is not directly related to the number of bits needed to compress each x^i. Instead, it represents the average time needed to compress a single target sequence, given the frozen compression models of the reference sequence.

- p.4, right column, "Complexity (NC) Profiles": how does the sorting by time affect the analysis? the first sentence of the section suggests that.

Response: As we have previously explained, the way the set of sequences is ordered will impact the results, since the program assumes the order of sequences in the multi-fasta (e.g. in temporal analysis, the first sequence is the oldest, where the most recent is the last). As such, the way data is provided to the program is of extreme important to obtain a valuable analysis of the data. A good example is the evolution of Sars-Cov-2 across time. If the order was incorrect, different profiles and analysis would be performed.

- p.4, right column, formula (8) introduces Σ as a new symbol for the alphabet of a sequence. We understand that a symbol for the alphabet of a sequence has already been introduced in p.3 as Ξ.

Response: Thank you for noticing this. Yes, the text has been changed accordingly.

- p.5, left column, 2nd paragraph: typo? "For genomic sequences, Σ is 4 for any" should it be the absolute value of Σ?

Response: Thank you for noticing this. Yes, the text has been changed accordingly.

- p.5, left column, 2nd paragraph: "alphabet trimming" - it would be helpful to add a sentence explaining alphabet trimming and why it is solving this issue

Response: Thank you for your suggestion. We agree that clarifying the concept of alphabet trimming would be beneficial for readers. In the context of proteomic sequences, the alphabet size can vary due to the presence of ambiguous or non-standard amino acid characters. This variation can lead to changes in the Normalized Compression (NC) profile, as the NC value is calculated using the alphabet size. Alphabet trimming is a preprocessing step that reduces the alphabet size by removing or substituting non-standard characters. By applying alphabet trimming, we ensure a consistent alphabet size across all sequences, mitigating the impact of alphabet variations on the NC profile. This step helps maintain the comparability of NC values and facilitates the interpretation of the sequence complexity analysis. We added a sentence in the revised manuscript to explain the concept of alphabet trimming and its role in addressing the issue of varying alphabet sizes in proteomic sequences.

- p.5, left column, section Relative singularity (RAWs) profiles, first sentence is hard to read, also add

another sentence to say why these regions are of interest

Response: We appreciate your feedback on the clarity of the first sentence in the "Relative singularity (RAWs) profiles" section. We have revised the sentence to improve readability and added another sentence to explain the significance of these regions.

- p.6, left column, "Results", could you provide another sentence of the motivation or what you want to find out with the SARS-CoV-2 data?

Response: Thank you for your suggestion. We agree that providing additional context about our motivation for analyzing the SARS-CoV-2 data would improve the clarity of the Results section. We have added a sentence to address this.

- p.6, left column, 2nd paragraph: "In some cases" is ambiguous, can you explain those cases

Response: Thank you for pointing out the ambiguity in the phrase "In some cases." We have clarified this by providing specific examples of when reading from standard input and writing to standard output are useful in AltaiR. The revised text is as follows:

"The toolkit allows reading and writing from standard input and output, respectively, in certain cases. For example, this feature enables the direct piping of the output from the filtering tool to the NC, NCD, and RAWs tools, streamlining the analysis process. Additionally, it allows for the output profiles of the frequency, NC, NCD, and RAWs tools to be easily processed by other tools, such as those performing averaging or visualization. This functionality provides easier integration to build custom pipelines. Furthermore, most tools (except average) use multi-threading to speed up the analysis."

The added examples clarify the specific instances where reading from standard input and writing to standard output are beneficial in AltaiR, such as directly connecting the output of one tool to another and facilitating the integration of AltaiR's tools with other software in custom pipelines.

- p.6, left column, 2nd paragraph: typo "README.am" > "README.md"

Response: Thank you for noticing this. Yes, the text has been changed accordingly.

- p.6, right column, 1st paragraph: "sufficient for accurate downstream analysis. This sequence filtering step shows its critical importance, proven by the high variability between these sequences, which can harm downstream analyses without proper filtering." → could you elaborate what potential effects could be?

Response: Thank you for your suggestion to elaborate on the potential effects of high variability between sequences on downstream analyses. We have expanded on this point in the revised text.

- p.6, "In some cases, the toolkit allows reading and writing from standard input and output". What are these cases and what are the limitations? What's exactly meant by "stand input and output"?

Response: Yes, as we have explained previously, the software can make use of stand input and output (i.e. use pipes: “|” in the command line environment), this allows for a more efficient execution of code. E.g. It is possible to filter the sequences, directly perform a NC analysis of the filtered sequences and preform and average of the results to them plot them. Of course, some pipe’s don’t make sense such as feeding the NC results to NCD.

- p.6, Which of the listed tools utilize multithreading? We suggest mentioning this either in a table or directly in the bullet points in the section "Toolkit Implementation".

Response: All of them, with the exception of average use multithreading. Since all tasks are computationally intensive and evolve a large number of sequences tasks the toolkit is developed in low-level language (C language) and make abundant use of multi-threading to make the computations as efficient and as fast as possible given the available resources.

- p.6, Figure 2: Consider limiting the x-axis range to (or closer to) the filtered interval

Response: We appreciate the reviewer insight. Although not visible, data points exist in the points shown in the Instagram, so for congruency, we will keep this graphic as is.

-p.6, right column, 2nd paragraph: "time point sequence" - what is that?

Response: A "time point sequence" refers to a sequence that represents a specific point in time within a series of sequences generated through a mutational process. In the context of the synthetic data example, the original sequence (time-0) is the starting point, and subsequent sequences (time-1, time-2, etc.) are generated by applying consecutive SNP mutations to the previous sequence. Each of these sequences represents a specific time point in the simulated evolutionary process.

In the first example, the time point sequences are used directly as references for computing the temporal similarity profiles. However, in the second example, mutated versions of the time point sequences are used as references to simulate a more realistic scenario where the available reference sequences already contain mutations compared to the true ancestral sequences.

- p.7. Figure 3: What is the "Sequence Number"

Response: In Figure 3 the “Sequence Number” refers to the positional index of the sequence in the multi-fasta file. As explained priorly the program assumes order of the input multi-fasta. Since this multi-fasta was previously ordered by date, the Sequence Number provides a temporal point in the file.

- p.7, Figure 3: difference between A and B is not clear, what sequences are used in A? what is the reference in B?

Response: The image “A” was computed with the reference being the Sars-Cov-2 refence genome sequences, and “B” was computed using RaTG13 sequence genome. The caption text was altered to improve readability and understanding of this.

- p.7. Figure 3, caption: "Similarity (NCD) profiles using (A) the whole filtered SARS-CoV-2 genome sequences and (B) the reference RaTG13 (B)." why twice (B) reference? - not clear

Response: As previously mentioned, the caption was updated since it was not clear. The first profile was computed using the Sars-Cov-2 reference sequence genomes; the second was computed using the RaTG13 sequence genomes, so the profiles are distinct. Although both are compared toward using the whole filtered SARS-CoV-2 genome sequences the reference sequences are different, and as such the profile is consequentially different as well.

- p. 7, left column, 5th paragraph: "The SARS-CoV-2 genomes are approximately 117k, resulting in multiple assembled genomes, sources, and techniques." → this sentence is not understandable

Response: Thank you for pointing out the lack of clarity in the sentence. The sentence has been changed in the manuscript for better clarity.

- p.9, Table 1: Changes in mRAWs Nucleotide Distribution are hard to make out from the table if the magnitude of the number’s changes. Consider normalizing these table entries so changes in nucleotide distributions across kmers are easy to spot.

Response: Thank you for your suggestion. While we agree that normalization could help spot relative changes, providing the absolute number of occurrences is important since absolute values give readers a clear understanding of the scale and magnitude of the mRAWs found in the SARS-CoV-2 genome. Furthermore, the absolute numbers provide context for the subsequent plots and analyses, allowing readers to interpret the results better and draw meaningful conclusions.

- p.9, Conclusion: "AltaiR's technical innovations, particularly in data filtering and processing [...]". AltaiR's filtering revolves on already commonly used and well-known summary statistics. Add more context to this statement.

Response: Thank you for your feedback regarding the statement about AltaiR's technical innovations in data filtering and processing. We acknowledge that some of the individual filtering methods used in AltaiR, such as sequence length and GC content, are well-established techniques. However, AltaiR's innovation lies in the integration of these methods with novel and enhanced features into a comprehensive toolkit specifically designed for efficient, large-scale genomic data analysis.

AltaiR introduces several new methods, such as the Frequency method, which provides a more comprehensive and detailed analysis of nucleotide or amino acid occurrence within the reads, enabling alphabet filtering. The Filter method extends beyond the capabilities of other tools by supporting multiple simultaneous pattern searches, absent pattern searches, and filtering by GC content, sequence length, or completeness.

Moreover, AltaiR's NCD method introduces a novel approach that uses a conjunction of reference and target files while freezing the models of one and saving computation time for other sequences. This methodology, specifically designed for AltaiR, allows for substantial time savings in NCD calculations.
The combination of these tools, along with the optimization for analyzing millions of sequences, ensures high portability and ease of installation while providing the necessary capabilities for rigorous analyses.
AltaiR's flexible integration into various analytical workflows through standard input and output combinations makes it a versatile choice for bioinformatics research.
We have revised the text in the to provide more context and clarity regarding AltaiR's technical innovations and their significance in the field of genomic data analysis.

- Supplementary Figure 1: Please add to the figure caption what the Time point is

Response: Thank you for the suggestion. We have updated the figure captions to include the definition of a "time point" in the context of the synthetic data experiment.

Reviewer #2:
In their manuscript, Silva et al introduce Altair, a toolkit written in the C programming language for the rapid analysis of sets of sequences in multi fasta format.
The authors showcase potential application of their tool on a collection of more than 6 million genomes from the SARS-CoV-2 coronavirus collected over the first 2 years of the COVID-19 pandemic.
In general, the paper is well written, although sometimes some biological concepts are expressed using inaccurate or imprecise/wording.
In the opinion of this reviewer the paper suffers from several important limitations, which should all be addressed for considering the work by Silva et al for publication.

Response: We appreciate the comments and hope the current revised manuscript satisfies the reviewer.

A list of the main point of criticism follows:
1# Abstract. Authors use some very strong wording to explain the rationale that led to the development of Altair. This reviewer would beg to disagree on the following concepts.
#1.1 Abstract/background: the first sentence needs to be completely rewritten, and specifically the reference to "the current phase of genome sequencing and reconstruction".

#1.2 Abstract/findings: please rephrase evolutionary patterns. What you highlight in the results is not evolutionary patterns, but rather interesting observations regarding viral genome characteristics in time. The epiphenomenon that you describe is more complex, and is the product of several factors, including natural selection, sampling bias, and mitigation policies by health authorities among the other things.

Response: We would like to thank the reviewer for the comments. We agree and the abstract has been updated to accommodate this information.

2# Introduction and rationale.

#2.1 I do not understand/agree with the choice to design Altair to work with information extracted from the headers of fasta files. For SARS-CoV-2 (and several other pathogens) structured metadata are available. See for example: https://nextstrain.org/ncov/open/global/6m and "All sequences and metadata".

Response: Thank you for your feedback. We understand your concern regarding the use of information extracted from the headers of FASTA files in AltaiR. While structured metadata, such as those available for SARS-CoV-2 through platforms like Nextstrain, can provide valuable information for analysis, AltaiR is designed to work at a lower level and serve a different purpose.

AltaiR's approach is intended to be more flexible and adaptable to various datasets, including those where structured metadata may not be readily available or consistently formatted. By extracting relevant information directly from the FASTA headers, AltaiR can be applied to a wider range of datasets and organisms without relying on external metadata sources.

Moreover, AltaiR's low-level approach allows for the analysis of raw sequence data, which can be particularly useful for exploratory studies, quality control, and cases where metadata may be incomplete or inconsistent.

#2.2 Please provide references for the section when you state: "However, creating temporary files ... in not efficiently affordable". Millions of SARS-CoV-2 sequences were analysed during the COVID-19 pandemic by using or adapting previously available tools. See for example: https://github.com/andersen-lab/bjorn and https://github.com/matteo14c/HaploCoV.

Response: Thank you for bringing the HaploCoV and bjorn tools to our attention, we will be sure to add them to the manuscript. These tools demonstrate the successful application of computational methods for analyzing large volumes of SARS-CoV-2 genomic data. However, we believe that AltaiR offers several unique advantages and capabilities that complement and extend the functionality of existing tools.

Firstly, AltaiR is specifically designed to handle vast amounts of genomic data efficiently. Its implementation in C and support for multi-threading enable faster processing times compared to tools primarily written in interpreted languages like Python or Perl. This efficiency is crucial when dealing with the ever-growing number of SARS-CoV-2 sequences.

Secondly, AltaiR's focus on alignment-free methods, such as generating Normalized Compression Distance (NCD) profiles and complexity profiles, sets it apart from tools that rely on alignment-based approaches. These methods enable efficient and scalable comparisons of genomic sequences without the computational burden of sequence alignment, making AltaiR well-suited for analyzing large, diverse datasets.

Moreover, AltaiR offers advanced filtering capabilities based on sequence length, GC content, and specific patterns, ensuring that high-quality, relevant data is used for downstream analyses. This rigorous filtering step, demonstrated in our analysis of the SARS-CoV-2 dataset, is essential for minimizing noise and bias in the results.

Another key strength of AltaiR is its ability to identify Relative Absent Words (RAWs) in genomic sequences. By comparing RAWs in SARS-CoV-2 genomes with those in the human genome, AltaiR can uncover distinctive genomic elements that may be relevant for understanding virus-host interactions and developing diagnostic tools. This functionality is not available in other toolkits (as far as we know).

While HaploCoV and bjorn have demonstrated their utility in specific aspects of SARS-CoV-2 genomic analysis, such as variant detection and data processing, AltaiR offers a more comprehensive suite of tools that enable a wide range of analyses, from similarity and complexity profiling to RAW identification and temporal analysis.

#2.3 Lack of tools for flagging/filtering low-quality sequences: incomplete and low quality sequences are normally flagged by sequence providers/submitters. Authors do explicitly refer to additional guidelines and

best practice, see [7,8]. Are these used/implemented by Altair. Please clarify.

Response: Thank you for your question. AltaiR has a highly parameterizable and customizable filtering system, which allows for the adherence to established guidelines and best practices to address potential contamination and quality issues. This automated filtering step removes non-complete, low-quality, biased, or deviant data based on criteria such as completeness, sequence length, GC content, and other patterns. By ensuring only high-quality, contiguous sequences are included in the analysis, AltaiR minimizes bias from incomplete or contaminated data, providing accurate and reliable downstream analyses. We have added this information to the text of the paper.

#2.4 The description of the properties of a collection of sequences over time does not necessarily represent evolution (see above). Please refrain from using expressions such as "accelerated evolution".

Response: Thank you for raising this important point. You are correct that the observed changes in the viral genomic characteristics over time may not solely represent evolutionary processes. We acknowledge that our use of the term "evolutionary patterns" and "evolutionary acceleration rates" in the manuscript may be an oversimplification.

In reality, the observed changes in the viral genome composition and complexity over time are likely the result of a complex interplay of various factors, including natural selection, sampling bias, and the implementation of public health measures. While some of these changes may indeed reflect the virus's adaptation and evolution within the host population, others may be influenced by non-evolutionary processes, such as the oversampling of certain viral lineages or the effects of containment strategies on viral transmission dynamics.

To address this, we have revised the manuscript to provide a more nuanced description of the observed temporal changes in the viral genome.

2.5 The long section where authors explain why a benchmark/comparison with other tools can not be performed is not very convincing, and above all it seems a bit out of context to this reviewer. Please adapt or remove.

Response: Thank you for your feedback. We understand that the previous section discussing the challenges of benchmarking and comparing toolkits may not have been entirely convincing or relevant to the overall context of the manuscript.

To address this concern, we have adapted the section to focus on a qualitative analysis of AltaiR's capabilities concerning other established toolkits, such as SeqAn, khmer, GTO, HaploCoV and bjorn. This comparative analysis highlights the key functionalities, efficiency metrics, and capabilities of each toolkit that are crucial for handling large-scale genomic data. By providing a more targeted and contextually relevant comparison, we aim to better illustrate AltaiR's unique features and its potential to complement and extend the capabilities of existing tools in the field of genomic data analysis.

#3 Methods

#3.1 In the opinion of this reviewer authors should better clarify which methods were specifically developed within Altair and which methods are "simply" adapted from other pre-existing software packages.

Response: The current methods include average, filter, frequency, NC, NCD, and RAW. Of these, only the average method was previously established. This is a straightforward program that has undergone a minor modification to allow selection of the column to use.

The AltaiR average method is very similar to the GTO average, with few modifications made.

The Frequency method in AltaiR is designed to compute the alphabet frequencies for each FASTA read, enabling alphabet filtering. Unlike other tools that offer alphabet filtering, AltaiR provides a more

comprehensive and detailed analysis by calculating the occurrence of each nucleotide or amino acid within the reads.

The Filter method shares small similarities with GTO, as it can filter FASTA reads. However, unlike GTO, AltaiR implementation supports multiple pattern searches simultaneously and also it include the ability to perform absent pattern searches. Furthermore, and more importantly, AltaiR's filter method includes the ability to filter by GC content, sequence length, or completeness.

The automatic computation of NCD and NC for multiple files is also a new feature. Previously, this was done using scripting, but AltaiR now provides a single tool for this purpose. Notably, scripting could not accommodate the new NCD methodology. AltaiR's NCD method, which uses a conjunction of reference and target files while freezing the models of one and saving the computation for other sequences, allows for substantial time savings. This novel NCD methodology is specifically designed for AltaiR, including a tool tailored for this specific functionality.

Finally, AltaiR RAW is an evolution of EAGLE2 (from the same authors), now including support for protein sequences, which was a limitation of EAGLE2, besides some other small additions. Thus, AltaiR RAW can be considered an EAGLE3 version (note that EAGLE3 was never published).

Moreover, the combination of these tools in a single package with optimization for millions of sequences ensures high portability and ease of installation while providing the necessary capabilities to perform rigorous analyses. Besides the capability to perform combinations of standard input and output, AltaiR allows for flexible integration into various analytical workflows, making it a versatile choice for bioinformatics research.

#3.2 But see also #2.1 I am not completely convinced that the utility for extracting/filtering information from the fasta header would be desirable and/or useful at all.

Response: Thank you for raising this concern. We understand that the utility of filtering information from the FASTA header may not be immediately apparent or universally applicable. However, we believe that this feature can be valuable in certain scenarios, particularly when dealing with large, heterogeneous datasets from various sources.

In many cases, FASTA headers contain valuable metadata, such as sample origin, date of isolation, sequencing platform, and other relevant information. This metadata can be crucial for downstream analyses, such as temporal studies, geographic comparisons, or quality control. By providing a flexible and efficient way to extract and filter this information, AltaiR enables researchers to quickly subset their datasets based on specific criteria without the need for additional preprocessing steps.

For example, when working with SARS-CoV-2 genomes from public repositories, the ability to filter sequences based on the date of isolation or geographic location can help researchers focus on specific subsets of interest, such as isolates from a particular outbreak or time period. Similarly, filtering based on sequencing platform or assembly method can help identify and remove low-quality or inconsistent sequences that may introduce biases in the analysis.

#4 Results

#4.1 Authors do not benchmark the computational requirements of Altair in terms of resources used. This needs to be addressed, authors need to demonstrate that Altair provide clear advantages w.r.t "alignment based" methods for the analysis of the same data.

Response: While we acknowledge the reviewer's suggestion to benchmark AltaiR's computational requirements against alignment-based methods, it's essential to recognize the inherent differences between alignment-free and alignment-based approaches. AltaiR's methodology, rooted in compression-based techniques like Normalized Compression Distance (NCD) and Normalized Compression (NC), fundamentally diverges from alignment-based methods by focusing on sequence compressibility rather than exact matching. This difference makes direct comparisons in computational resource usage

challenging, as the methodologies serve different purposes and exhibit distinct computational characteristics. Moreover, alignment-based methods may inadvertently underestimate sequence similarity due to their reliance on exact matches, whereas compression-based techniques, as encapsulated in AltaiR, leverage the Kolmogorov complexity notion to capture similarity more comprehensively. Additionally, AltaiR is developed in optimized C language, theoretically enhancing its efficiency compared to applications developed in higher-level languages such as Python, Bash, R, or Perl. While alignment-based methods may entail significant computational overhead due to sequence alignment requirements, AltaiR's alignment-free approach offers efficiency and scalability, particularly suited for large and diverse genomic datasets. Thus, while quantitative comparisons may not be directly feasible, AltaiR's unique methodology represents a valuable alternative, enriching the toolkit available to researchers for comprehensive genomic analyses.

#4.2 Criteria applied for filtering SARS-CoV-2 sequences are too stringent. In the end only ~25k sequences are retained, starting from 6.3M. This is a very big issue in the opinion of this reviewer. Filters on length in particular are very stringent. I am not an expert in the field, but are alignment-free methods applicable when sequences are not all of the same size? This could be an important limitation. Please discuss.

Response: We must have quality data even if we have quantity. We have analyzed epidemiologic context but also in medical and forensic contexts (papers below) where it was required to perform intra-host analysis between large genomes such as herpesvirus, and therefore in these cases it is better to have the best quality data even if the number of sequences is lower.

Clinic Setting example:
-Pyöriä, Lari, et al. "Unmasking the tissue-resident eukaryotic DNA virome in humans." Nucleic Acids Research 51.7 (2023): 3223-3239.

Forensic setting example:
-Toppinen, Mari, et al. "The human bone marrow is host to the DNAs of several viruses." Frontiers in cellular and infection microbiology 11 (2021): 657245.

Notice that we used of ~25k high-quality sequences to showcase the toolkit. Here, we decided to choose less sequences but of higher quality. The toolkit is flexible to perform other analysis including when the size of the sequences differs substantially.

#4.3 NCD: the whole point of the simulation is unclear to me. The analysis is rather qualitative, to confidently state that "NCD profiles can ... predict where sequences occurred in time", you need to provide accuracy metrics (how confidently can you predict?) and evaluate also additional parameters. Further assume uniform sampling and uniform evolution are assumed: this is not very realistic.

Response: We thank the reviewer for the comments. We agree that the simulation and analysis presented are qualitative and may not provide sufficient evidence to confidently state the predictive power of NCD profiles in determining the temporal occurrence of sequences.

In light of this fact, we have revised the NCD profiles section to clarify that the primary purpose of presenting these experiments is to showcase the functionality and potential applications of the AltaiR toolkit. We have also emphasized that while NCD profiles have shown promising trends and have been successful in some cases, further investigation and more extensive research are necessary to validate their predictive power.

Our current focus is on introducing the AltaiR toolkit and its various features, and we believe that the revised section better reflects this objective. We appreciate your insights and have incorporated them into the manuscript to provide a more correct representation of the NCD profiles application.

#4.4 Since NCD is not equivalent to a molecular clock and non-calibration with sequences with "certain" temporal origins were made authors cannot conclude anything from their analysis of the RaTG13 genome. To be honest I would not include that analysis in the main text.

Response: Thank you for your comment regarding the analysis of the RaTG13 genome using NCD profiles.

You raise a valid point that NCD is not equivalent to a molecular clock, and without proper calibration using sequences with known temporal origins, drawing definitive conclusions about the temporal origin of RaTG13 based solely on this analysis may be premature.

We agree that the NCD analysis of RaTG13 should be interpreted with caution, as several factors could influence the observed similarity patterns, such as the reconstruction process used for RaTG13 (e.g., reference-based assembly or de novo assembly) and the diversity of the samples used. Additionally, the lack of a robust temporal calibration limits the ability to make precise inferences about the timing of RaTG13's origin. Given these limitations, we have decided to remove the RaTG13 analysis from the main text and instead include it as a supplementary discussion.

#4.5 The sections "Frequency profile application" and "Relative singularity (RAWs) profiles" are the most interesting parts of the paper in my opinion. However, a far larger number of sequences should be included in the analysis (see #4.1).

Response: Thank you for your positive feedback. Regarding your suggestion to include a larger number of sequences in the analysis, we understand the potential benefits of analyzing a more extensive dataset. However, our primary focus in this study was to prioritize the quality and reliability of the sequences used, rather than solely aiming for a larger quantity. By carefully filtering the SARS-CoV-2 genomes based on their completeness, quality, and the presence of reliable metadata, we aimed to ensure that the downstream analyses would be based on high-quality data. The inclusion of incomplete, low-quality, or poorly annotated sequences could potentially introduce biases or artifacts that might obscure the true biological patterns and trends we aim to uncover.

#5 Discussion

#5.1 Authors should present the limitations of alignment-free methods as compared to alignment-based methods. Although the former are more computationally efficient, they do not generate the same knowledge/amount of information. This should be set out.

Response: Thank you for your suggestion. We agree that this is an important point to address, and we have added this information to the manuscript.

#5.2 Authors should also test Altair on a different use case. Several large-scale collections of bacterial pathogens (see Listeria, pathogenic E.coli, Neisseria for example) are available through INSDC databases.

Response: Thank you for your suggestion. The primary aim of our study was to demonstrate the functionality of the tool and its applicability to large datasets. Therefore, we applied it to both synthetic and real data to maintain clarity and focus in our narrative. While testing Altair on additional datasets like bacterial pathogens could be insightful, we believe it might overcomplicate this particular article. We appreciate your input and might explore these applications in future research.
