# Supplementary material for: AltaiR: a C toolkit for alignment-free and temporal analysis of multi-FASTA data
Source: Gigascience. 2024 Nov 26;13:giae086. doi: 10.1093/gigascience/giae086 (PMC11590114; doi:10.1093/gigascience/giae086)
Supplement: giae086_Response_to_Reviewer_Comments_Revision_1 [file giae086_response_to_reviewer_comments_revision_1.pdf]

Author's Response To Reviewer Comments

Editor and Reviewer Comments:

We are grateful for the reviewer’s positive appreciation of our work and revisions to the manuscript. We carefully considered the reviewers’ comments and have revised the manuscript to address each comment. We hope the manuscript is now acceptable for publication.

Reviewer #1:
Thanks for addressing our comments. In the following, please find some minor comments and additional comments to your responses to the previous major comments (answers marked by 'A').

Minor
- l.284: it would be useful to be clearer that the samples sorted by date are required for parts of the analysis.
Response: Response: We appreciate the reviewer's suggestion. We have updated the manuscript to clarify this important point. The following sentence has been added:
“It is important to note that for temporal analyses, such as the NC, NCD profiles and frequency profiles, the input sequences must be sorted by date. This sorting is crucial for accurately capturing trends and patterns over time.”

- Table 3: What does "some cases" in the row "Alignment-free analysis for GTO mean? Either address this in the table or in the discussion section.

Response: We appreciate the reviewer's request for clarification. We have revised Table 3 to provide more precision regarding GTO's alignment-free capabilities. The entry for GTO now reads:
"Partial, compression"
Additionally, we have added the following explanation to the discussion section:
"It also differs from tools like GTO in that, while GTO possesses some alignment-free methods such as sequence compression and can be adapted to compute complexity profiles, it is not primarily designed for comprehensive alignment-free analysis.

- Table 3: "Basic filtering" in the row "Genome Filtering" is unclear. Can you include short descriptions, as provided in the column for AltaiR, either in the table or add it as information to the sentence in line 798-803?

Response: Thank you for bringing this to our attention. You're correct that the "Basic filtering" description in Table 3 for the Genome Filtering row lacks clarity. To address this, we will provide more detailed information both in the table and in the text.

We have also added the following sentence to provide additional context:
"AltaiR's ability to filter sequences rigorously was demonstrated in the analysis of the SARS-CoV-2 dataset. It allows users to filter sequences based on length, GC content, and specific patterns, offering more comprehensive and customizable functionality compared to the filtering capabilities provided by SeqAn, khmer, and GTO, while HaploCoV and bjorn do not provide explicit filtering options. This advanced filtering is particularly useful for processing large-scale genomic datasets, where precise data selection is crucial for downstream analyses. For instance, AltaiR's ability to filter based on GC content can be invaluable in studies focusing on organisms with specific genomic compositions or in identifying potential contamination in sequencing data. While SeqAn and GTO offer basic filtering based on sequence length and simple pattern matching, and khmer provides k-mer-based filtering, AltaiR extends these capabilities by allowing for sophisticated filtering based on multiple criteria simultaneously..

- Figure 1: the figure appears to be slightly cut off on the left side
Response: We appreciate the reviewer's keen observation regarding Figure 1. We have carefully examined the figure and made the necessary adjustments to ensure that it is no longer cut off on the left side.

- abstract: it would be useful to be more precise regarding the sequence complexity: "[…] decreases in average Kolmogorov sequence complexity" to make it clear.
Response: We appreciate your suggestion to clarify the concept of sequence complexity in the abstract. You are correct that being more precise will enhance the clarity of our findings. We have updated the abstract to specifically mention Kolmogorov complexity.

- It seems like one of the main advantages of this tool is its clever header filtering. The authors should provide a runtime comparison to the grep method. Please add this to the section "Computational efficiency".

Response: We appreciate the reviewer's suggestion to compare AltaiR's header filtering capabilities with traditional Unix tools. We have conducted a comprehensive comparison between AltaiR and an equivalent grep/awk-based approach for filtering FASTA sequences. The comparison includes filtering based on sequence length, nucleotide content, and specific header patterns, ensuring a fair assessment of both methods. We have added this information to the "Computational efficiency" section of the manuscript.

- Check the ambiguity of the letter y^i and y in the sections "Header patterns and anti-patterns" and "Similarity (NCD) profiles"
Response: We appreciate the reviewer's observation regarding the potential ambiguity in our notation. Upon careful consideration, we agree that the current use of y^i and y across different sections may lead to confusion. To address this, we have replaced y^i with h^i to represent sequence headers.

Reviewer #2:

Authors have adequately addressed the majority of the points raised by this reviewer in the revised version of the manuscript. However a couple of points still need some clarifications in my opinion.

Point 4.1:

I understand the point of view of the authors and I appreciate that a section detailing the execution times for different task performed by Altair was added to the manuscript (Table 2 in particular). However, a computational requirements analysis should also report the amount of resources used, including the amount of RAM memory and disk space to be complete. Could this information be added? I expect that probably Altair will consume only a limited amount of RAM and disk space, but this might be an important information as well for s

Response: We appreciate the reviewer's suggestion to include information about RAM usage and disk space requirements. As requested, we have updated the manuscript to include peak RAM usage for each method. Regarding disk space, AltaiR's output file sizes vary depending on the input data and specific operation being performed. Since these requirements can change based on the dataset and analysis parameters, we have included the system's specifications for the tests we performed in the computational efficiency section.

Point 4.2:

Although I get the point, very stringent quality filters might as well bias the results. Specifically in the case of SARS-CoV-2 these filters might skew the representation of the sequences included in the analyses towards some specific sequencing strategies and/or sequencing centers, and even worse discard some viral variants for which "high quality" genome sequences might not be available.
Could the authors please at least assess/demonstrate that the filters applied do not change substantially the composition of the dataset in terms of the viral variants therein represented? A simple table with the proportion of sequences assigned to the 10 most represented viral variants before and after filters are applied could be enough.

Response: We appreciate the reviewer's concern regarding the potential bias introduced by stringent

quality filters. To address this, we conducted a comprehensive analysis of the variant distribution before and after filtering. Our results demonstrate that while the filtering process does reduce the total number of sequences, it maintains a consistent representation of the major viral variants.
To assess the impact of our filtering criteria, we analyzed the distribution of variants before and after filtering. The results are summarized in the following table, which shows the top 10 most represented viral variants:

Variant	Before %	After %
D614G	42.52	64.13
Epsilon	37.85	6.33
Iota	12.55	8.01
Eta	2.58	16.74
Lambda	2.29	0.01
Zeta	1.72	4.40
Beta	0.34	0.00
Other	0.15	0.00
Gamma	0.01	0.37

To quantify the overall consistency of the variant distribution before and after filtering, we calculated the following similarity metrics, the Pearson correlation: 0.7348; the. Spearman correlation: 0.7982 and the Cosine similarity: 0.7940.
These metrics indicate a strong positive correlation and similarity between the variant distributions before and after filtering. The all correlations show values above 0.73, suggesting that the overall structure of the variant distribution is largely maintained.
While there are changes in the proportions of individual variants, the most prevalent variants (D614G, Epsilon, and Iota) remain well-represented in the filtered dataset. The observed changes in proportions can be attributed to the removal of low-quality sequences, which may have been overrepresented in certain variants due to sequencing or assembly issues.
It's important to note that our filtering criteria were designed to remove sequences with potential quality issues while retaining a representative sample of high-quality sequences across variants. This approach allows for more reliable downstream analyses by focusing on sequences that meet stringent quality standards.
To provide a clear visualization of these results, we have added a new figure (Figure 3) to the manuscript, which illustrates the variant distribution before and after filtering. This figure has been placed in the Filtering sequences subsection of the Results section.
We have also updated the manuscript to include this analysis in the "Filtering sequences" subsection of the Results section. The added text reads as follows:
“To assess the impact of our filtering criteria on the representation of viral variants, we compared the distribution of the top 10 most represented variants before and after filtering. Our analysis shows that while the filtering process does reduce the total number of sequences from 1,538,095 to 25,594, it maintains a consistent representation of the major viral variants. The Pearson correlation (0.7348), Spearman correlation (0.7982), and cosine similarity (0.7940) between the before and after distributions indicate a strong positive correlation and overall similarity in variant representation. Figure 3 illustrates the variant distribution before and after filtering, demonstrating that the most prevalent variants remain well-represented in the filtered dataset.”
