# Supplementary material for: AltaiR: a C toolkit for alignment-free and temporal analysis of multi-FASTA data
Source: Gigascience. 2024 Nov 26;13:giae086. doi: 10.1093/gigascience/giae086 (PMC11590114; doi:10.1093/gigascience/giae086)
Supplement: giae086_Response_to_Reviewer_Comments_Revision_2 [file giae086_response_to_reviewer_comments_revision_2.pdf]

Author's Response To Reviewer Comments

Dear Dr. Zauner,

Thank you for your email and for accepting our manuscript. We appreciate the valuable feedback and are pleased to provide the necessary revisions as per your editorial requests.
