# Supplementary material for: AltaiR: a C toolkit for alignment-free and temporal analysis of multi-FASTA data
Source: Gigascience. 2024 Nov 26;13:giae086. doi: 10.1093/gigascience/giae086 (PMC11590114; doi:10.1093/gigascience/giae086)
Supplement: giae086_Reviewer_1_Report_Original_Submission — Niko Beerenwinkel -- 2/26/2024 [file giae086_reviewer_1_report_original_submission.pdf]

Reviewer Report

Title: AltaiR: a C toolkit for alignment-free and temporal analysis of multi-FASTA data

Version: Original Submission **Date:** 2/26/2024

Reviewer name: Niko Beerenwinkel

Reviewer Comments to Author:

SummaryThe authors present a toolkit (AltaiR) for analysing multiple sequences in multi-FASTA format that is exclusively characterized by alignment-free methodologies.It computes:-- Normalized Compression Distance profiling-- Normalized Compression (complexity) analysis-- temporal nucleotide composition variation-- the identification of Relative Absent Words,The manuscript is hard to read. However, the tool is well implemented and the GitRepo is user-friendly.CommentsMajor- The term "spatial-temporal" in the title is misleading because the method takes neither spatial nor temporal dependencies into account.Update installation instructions:The one on the GitHub doesn't work instead:mamba create -n altair -c conda-forge -c bioconda altair-mfWithout comparison to other methods or toolkits, sentences like "ALtaiR is a significant advancement in the field" (see Discussion) don't seem appropriate. Therefore it would be useful to include the following comparisons:Please include a comparison (as a table?) to other toolkits that are mentioned in the introduction (SeqAn, khmer, GTO, etc) in terms of functionalities and efficiency (programming language, parallel computing)It seems like one of the main advantages of this tool is its clever header filtering. The authors should provide a runtime comparison to the grep method.The input requirements are not clear:- the authors should specify explicitly for which pathogens the tool is suitable- what is the minimum input AltaiR requires?- is the tool using the information of when the sequence was submitted/samples for the temporal mapping? How are these passed to the tool ?- what is the minimum number of sequences you would recommend users to use?- how does the tool behave on substantially larger genomes?- What is the overall runtime of your SARS-CoV-2 application? Can you put this in relation to other tools? This would give users a better understanding of what to expect.The method section appears to be unnecessarily hard to read (concrete cases are listed in the minor comments section). It contains a lot of seemingly complex formulas for already established and well known summary statistics (e.g. GC content, nucleotide-/amino acid - frequency, etc.). Also, it is not described how AltaiR filters sequences by "completeness". Additionally, the manuscript would benefit from a clear overview figure.Minor-General: Missing line numbers impedes the reviewing process as it makes it harder to point out things while reading the manuscript.- abstract: Please define sequence complexity!- abstract: can you use a more specific term than "flexible"?- p.2, Introduction: spatial-temporal characteristic: what are those characteristics? And where is the spatial or temporal data coming in?- p.3, left column: what is "compact version of the data"? Be more precise compressed data? " to compact data .." â†’ is this compressed?- p.3, left column: "The workflow contains seven main methods" â†’ are those all applied to all the three data inputs?- p.3, Figure 1: Maybe you can use different symbols for data and methods, and in general make clear which method is used for which data. What is the minimum input ?Can the input be sequences from different pathogens ?Which method is for which input data (maybe color-coding in the

figure could help)- p.3, right column: "The computation can be applied to different sequences;" â†' the word computation is irritating, this is a filtering step.- p.3, right column: "The computation can be applied to different sequences;"â†' is the essence of this and the following sentence that different filtering characteristics can be applied to different viruses and they can be changed while the program is running? If so please, make it precise- p.3, right column: the phrase "intersected through a single run" is not clear. Are you allowed to change the filter criteria while running? this seems not in accordance with reproducibility- p.3, right column: term "Alphabet composition" is not clear.- p.3, right column: "Alphabet composition": what you are doing with symbols that don't conform with the given alphabet, is the whole sequence excluded? Is only that symbol excluded, and does that have an effect on the analysis?- p.4, left column: "Header patterns and anti-patterns"; it doesn't seem necessary to introduce the mathematical notation "yi", which is not used subsequently- p.4, left column, last paragraph: what is the "reference sequence"? How is it defined by the user and how should it be chosen?- p.4, right column, mentions a time complexity of y + xn. It's not quite clear how the value of x is calculated. Does this somehow correspond to the previously mentioned number of bits to compress each individual x^i? If yes, how is this generalized to a single value of x?- p.4, right column, "Complexity (NC) Profiles": how does the sorting by time affect the analysis? the first sentence of the section suggests that.- p.4, right column, formula (8) introduces Î£ as a new symbol for the alphabet of a sequence. We understand that a symbol for the alphabet of a sequence has already been introduced in p.3 as Îž.- p.5, left column, 2nd paragraph: typo? "For genomic sequences,Î£ is 4 for any" should it be the absolute value of Î£ ?- p.5, left column, 2nd paragraph: "alphabet trimming" - it would be helpful to add a sentence explaining alphabet trimming and why it is solving this issue- p.5, left column, section Relative singularity (RAWs) profiles, first sentence is hard to read, also add another sentence to say why these regions are of interest- p.6, left column, "Results", could you provide another sentence of the motivation or what you want to find out with the SARS-CoV-2 data?- p.6, left column, 2nd paragraph: "In some cases" is ambiguous, can you explain those cases- p.6, left column, 2nd paragraph: typo "README.am" > "README.md"- p.6, right column, 1st paragraph: "sufficient for accurate downstream analysis.This sequence filtering step shows its critical importance, proven by the high variability between these sequences, which can harm downstream analyses without proper filtering." â†' could you elaborate what potential effects could be?- p.6, "In some cases, the toolkit allows reading and writing from standard input and output". What are these cases and what are the limitations? What's exactly meant by "stand input and output"?- p.6, Which of the listed tools utilize multithreading? We suggest mentioning this either in a table or directly in the bullet points in the section "Toolkit Implementation".- p.6, Figure 2: Consider limiting the x-axis range to (or closer to) the filtered interval-p.6, right column, 2nd paragraph: "time point sequence" - what is that?- p.7. Figure 3: What is the "Sequence Number"- p.7, Figure 3: difference between A and B is not clear, what sequences are used in A ? what is the reference in B?- p.7. Figure 3, caption: "Similarity (NCD) profiles using (A) the whole filtered SARS-CoV-2 genome sequences and (B) the reference RaTG13 (B)." why twice (B) reference? - not clear- p. 7, left column, 5th paragraph: "The SARS-CoV-2 genomes are approximately 117k, resulting in multiple assembled genomes, sources, and techniques." â†' this sentence is not understandable- p.9, Table 1: Changes in mRAWs Nucleotide Distribution are hard to make out from the table if the magnitude of the numbers changes. Consider normalizing these table entries so changes in nucleotide distributions across kmers are easy to spot.- p.9, Conclusion: "AltaiR's technical innovations, particularly in data filtering and processing [...]". AltaiR's filtering revolves on

already commonly used and well known summary statistics. Add more context to this statement.- Supplementary Figure 1: Please add to the figure caption what the Time point is

Level of Interest

Please indicate how interesting you found the manuscript: Choose an item.

Quality of Written English

Please indicate the quality of language in the manuscript: Choose an item.

Declaration of Competing Interests

Please complete a declaration of competing interests, considering the following questions:

- Have you in the past five years received reimbursements, fees, funding, or salary from an organisation that may in any way gain or lose financially from the publication of this manuscript, either now or in the future?
- Do you hold any stocks or shares in an organisation that may in any way gain or lose financially from the publication of this manuscript, either now or in the future?
- Do you hold or are you currently applying for any patents relating to the content of the manuscript?
- Have you received reimbursements, fees, funding, or salary from an organization that holds or has applied for patents relating to the content of the manuscript?
- Do you have any other financial competing interests?
- Do you have any non-financial competing interests in relation to this paper?

If you can answer no to all of the above, write 'I declare that I have no competing interests' below. If your reply is yes to any, please give details below.

I declare that I have no competing interests.

I agree to the open peer review policy of the journal. I understand that my name will be included on my report to the authors and, if the manuscript is accepted for publication, my named report including any attachments I upload will be posted on the website along with the authors' responses. I agree for my report to be made available under an Open Access Creative Commons CC-BY license (http://creativecommons.org/licenses/by/4.0/). I understand that any comments which I do not wish to be included in my named report can be included as confidential comments to the editors, which will not be published.

Choose an item.

To further support our reviewers, we have joined with Publons, where you can gain additional credit to further highlight your hard work (see: https://publons.com/journal/530/gigascience). On publication of this paper, your review will be automatically added to Publons, you can then choose whether or not to claim your Publons credit. I understand this statement.

Yes Choose an item.
