# Supplementary material for: AltaiR: a C toolkit for alignment-free and temporal analysis of multi-FASTA data
Source: Gigascience. 2024 Nov 26;13:giae086. doi: 10.1093/gigascience/giae086 (PMC11590114; doi:10.1093/gigascience/giae086)
Supplement: giae086_Reviewer_1_Report_Revision_1 — Niko Beerenwinkel -- 7/12/2024 [file giae086_reviewer_1_report_revision_1.pdf]

Reviewer Report

Title: AltaiR: a C toolkit for alignment-free and temporal analysis of multi-FASTA data

Version: Revision 1 **Date:** 7/12/2024

Reviewer name: Niko Beerenwinkel

Reviewer Comments to Author:

Thanks for addressing our comments. In the following, please find some minor comments and additional comments to your responses to the previous major comments (answers marked by 'A').## Minor- l.284: it would be useful to be clearer that the samples sorted by date is a requirement for parts of the analysis.- Table 3: What does "some cases" in the row "Alignment-free analysis for GTO mean? Either address this in the table or in the discussion section.- Table 3: "Basic filtering" in the row "Genome Filtering" is unclear. Can you include short descriptions, as provided in the column for AltaiR, either in the table or add it as information to the sentence in line 798-803?- Figure 1: the figure appears to be slightly cut off on the left side- abstract: it would be useful to be more precise regarding the sequence complexity: "[…] decreases in average Kolmogorov sequence complexity" to make it clear.- It seems like one of the main advantages of this tool is its clever header filtering. The authors should provide a runtime comparison to the grep method. Please add this to the section "Computational efficiency".- Check ambiguity of the letter y^i and y in the sections "Header patterns and anti-patterns" and "Similarity (NCD) profiles"## Revisions* The term "spatial-temporal" in the title is misleading because the method takesneither spatial nor temporal dependencies into account.A: Thanks for updating the title of the manuscript.* Update installation instructionsA: Thanks, we believe this could help other users that might struggle with the installation as well.* Without comparison to other methods or toolkits, sentences like "ALtaiR is a significant advancement in the field" (see Discussion) don't seem appropriate to us. Therefore it would be useful to include the following comparisons:Please include a comparison (as a table?) to other toolkits that are mentioned in the introduction (SeqAn, khmer, GTO, etc) in terms of functionalities and efficiency (programming language, parallel computing)A: Thanks a lot for adding the table. This will make it easier for potential users to assess the advantages of AltaiR. However, what does "some cases" in the row "Alignment-free analysis for GTO mean? Either address this in the table or in the discussion section. Also, "Basic filtering" in the row "Genome Filtering" is unclear. Can you include short descriptions, as provided in the column for AltaiR, either in the table or add it as information to the sentence in line 798-803? Finally, the last 4 rows of the table could be summarized in a single row called "further functions".It seems like one of the main advantages of this tool is its clever header filtering. The authors should provide a runtime comparison to the grep method.A: This was not addressed. Please add this to the section "Computational efficiency".* The input requirements are not clear.A: We thank the author for improving Figure 1 this clarifies the input requirements for each part of the analysis. Note, that the figure appears to be slightly cut off on the left side.* What is the overall runtime of your SARS-CoV-2 application? Can you put this in relation to other tools? This would give users a better understanding of what to expect.A: Thanks for adding the section "Computational efficiency".* The method section appears to be unnecessarily hard to read (concrete

cases are listed in the minor comments section). It contains a lot of seemingly complex formulas for already established and well known summary statistics (e.g. GC content, nucleotide-/amino acid -frequency, etc.). Also, it is not described how AltaiR filters sequences by "completeness". Additionally, the manuscript would benefit from a clear overview figure.A: We thank the authors for improving Figure 1 and including the definition of sequence completeness in the manuscript.*Abstract: Please define sequence complexity!A: Thanks you for the clarification w.r.t. the Kolmogorov complexity. We think it would be helpful if you included this in the abstract, e.g. "[…] decreases in average Kolmogorov sequence complexity" to make it clear.*p.4, left column: "Header patterns and anti-patterns"; it doesn't seem necessary to introduce the mathematical notation "yi", which is not used subsequently.Response: We prefer to keep notation to provide a mathematical congruence to the text.A: This might introduce more confusion than it does actually contribute to congruence. In the section "Header patterns and anti-patterns", y^i corresponds to a sequence header, but in the next section, "Similarity (NCD) profiles", y is used in formula 5 to represent a reference sequence or genome (but not the header).

Level of Interest

Please indicate how interesting you found the manuscript: Choose an item.

Quality of Written English

Please indicate the quality of language in the manuscript: Choose an item.

Declaration of Competing Interests

Please complete a declaration of competing interests, considering the following questions:

- Have you in the past five years received reimbursements, fees, funding, or salary from an organisation that may in any way gain or lose financially from the publication of this manuscript, either now or in the future?
- Do you hold any stocks or shares in an organisation that may in any way gain or lose financially from the publication of this manuscript, either now or in the future?
- Do you hold or are you currently applying for any patents relating to the content of the manuscript?
- Have you received reimbursements, fees, funding, or salary from an organization that holds or has applied for patents relating to the content of the manuscript?
- Do you have any other financial competing interests?
- Do you have any non-financial competing interests in relation to this paper?

If you can answer no to all of the above, write 'I declare that I have no competing interests' below. If your reply is yes to any, please give details below.

I declare that I have no competing interests.

I agree to the open peer review policy of the journal. I understand that my name will be included on my report to the authors and, if the manuscript is accepted for publication, my named report including any attachments I upload will be posted on the website along with the authors' responses. I agree for my report to be made available under an Open Access Creative Commons CC-BY license (http://creativecommons.org/licenses/by/4.0/). I understand that any comments which I do not wish to be included in my named report can be included as confidential comments to the editors, which will not be published.

Choose an item.

To further support our reviewers, we have joined with Publons, where you can gain additional credit to further highlight your hard work (see: https://publons.com/journal/530/gigascience). On publication of this paper, your review will be automatically added to Publons, you can then choose whether or not to claim your Publons credit. I understand this statement.

Yes Choose an item.
