# Supplementary material for: AltaiR: a C toolkit for alignment-free and temporal analysis of multi-FASTA data
Source: Gigascience. 2024 Nov 26;13:giae086. doi: 10.1093/gigascience/giae086 (PMC11590114; doi:10.1093/gigascience/giae086)
Supplement: giae086_Reviewer_2_Report_Original_Submission — MATTEO CHIARA -- 3/10/2024 [file giae086_reviewer_2_report_original_submission.pdf]

Reviewer Report

Title: AltaiR: a C toolkit for alignment-free and temporal analysis of multi-FASTA data

Version: Original Submission **Date:** 3/10/2024

Reviewer name: MATTEO CHIARA

Reviewer Comments to Author:

In their manuscript Silva et al introduce Altair, a toolkit written in the C programming language for the rapid analysis of sets of sequences in multi fasta format.The authors showcase potential application of their tool on a collection of more than 6 million genomes from the SARS-CoV-2 coronavirus collected over the first 2 years of the COVID-19 pandemic.In general the paper is well written, although sometimes some biological concepts are expressed using inaccurate or imprecise/wording.In the opinion of this reviewer the paper suffers from several important limitations, which should all be addressed for considering the work by Silva et al for publication.A list of the main point of criticism follows:1# Abstract. Authors use some very strong wording to explain the rationale that lead to the development of Altair. This reviewer would beg to disagree on the following concepts.#1.1 Abstract/background: the first sentence needs to be completely rewritten, and specifically the reference to "the current phase of genome sequencing and reconstruction".#1.2 Abstract/findings: please rephrase evolutionary patterns. What you highlight in the results is not evolutionary patterns, but rather interesting observations regarding viral genome characteristics in time. The epiphenomenon that you describe is more complex, and is the product of several factors, including natural selection, sampling bias, and mitigation policies by health authorities among the other things.2# Introduction and rationale.#2.1 I do not understand/agree with the choice to design Altair to work with information extracted from the headers of fasta files. For SARS-CoV-2 (and several other pathogens) structured metadata are available. See for example: https://nextstrain.org/ncov/open/global/6m and "All sequences and metadata". #2.2 Please provide references for the section when you state: "However, creating temporary files ... in not efficiently affordable". Millions of SARS-CoV-2 sequences were analysed during the COVID-19 pandemic by using or adapting previously available tools. See for example: https://github.com/andersen-lab/bjorn and https://github.com/matteo14c/HaploCoV.#2.3 Lack of tools for flagging/filtering low quality sequences: incomplete and low quality sequences are normally flagged by sequence providers/submitters. Authors do explicitly refer to additional guidelines and best practice, see [7,8]. Are these used/implemented by Altair. Please clarify.#2.4 The description of the properties of a collection of sequences over time does not necessarily represent evolution (see above). Please refrain from using expressions such as "accelerated evolution".#2.5 The long section where authors explain why a benchmark/comparisons with other tools can not be performed is not very convincing, and above all it seems a little bit out of context to this reviewer. Please adapt or remove.#3 Methods#3.1 In the opinion of this reviewer authors should better clarify which methods were specifically developed within Altair and which methods are "simply" adapted from other pre-existing software packages.#3.2 But see also #2.1 I am not completely convinced that the utility for extracting/filtering information from the fasta header would be desirable and/or useful at all.#4

Results#4.1 Authors do not benchmark the computational requirements of Altair in terms of resources used. This needs to be addressed, authors need to demonstrate that Altair provide clear advantages w.r.t "alignment based" methods for the analysis of the same data.#4.2 Criteria applied for filtering SARS-CoV-2 sequences are too stringent. In the end only ~25k sequences are retained, starting from 6.3M. This is a very big issue in the opinion of this reviewer. Filters on length in particular are very stringent. I am not an expert in the field, but are alignment free methods applicable when sequences are not all of the same size? This could be an important limitation. Please discuss.#4.3 NCD: the whole point of the simulation is unclear to me. The analysis is rather qualitative, to confidently state that "NCD profiles can ... predict where sequences occurred in time", you need to provide accuracy metrics (how confidently can you predict?), and evaluate also additional parameters. Further assume uniform sampling and uniform evolution are assumed: this is not very realistic.#4.4 Since NCD is not equivalent to a molecular clock and non calibration with sequences with "certain" temporal origins were made authors can not conclude anything from their analysis on the RaTG13 genome. To be honest I would not include that analysis in the main text.#4.5 The sections "Frequency profile application" and "Relative singularity (RAWs) profiles" are the most interesting part of the paper in my opinion. However a far larger number of sequences should be included in the analysis (see #4.1).#5 Discussion#5.1 Authors should clearly present the limitations of alignment free methods as compared to alignment based methods. Although the former are more computationally efficient, they do not generate the same knowledge/amount of information. This should be cleary set out.#5.2 Authors should also test Altair on a different use case. Several large scale collection of bacterial pathogens (see Listeria, pathogenic E.coli, Neisseria for example) are available through INSDC databases.

Level of Interest

Please indicate how interesting you found the manuscript: Choose an item.

Quality of Written English

Please indicate the quality of language in the manuscript: Choose an item.

Declaration of Competing Interests

Please complete a declaration of competing interests, considering the following questions:

- Have you in the past five years received reimbursements, fees, funding, or salary from an organisation that may in any way gain or lose financially from the publication of this manuscript, either now or in the future?
- Do you hold any stocks or shares in an organisation that may in any way gain or lose financially from the publication of this manuscript, either now or in the future?
- Do you hold or are you currently applying for any patents relating to the content of the manuscript?

- Have you received reimbursements, fees, funding, or salary from an organization that holds or has applied for patents relating to the content of the manuscript?
- Do you have any other financial competing interests?
- Do you have any non-financial competing interests in relation to this paper?

If you can answer no to all of the above, write 'I declare that I have no competing interests' below. If your reply is yes to any, please give details below.

I declare that I have non competing interests

I agree to the open peer review policy of the journal. I understand that my name will be included on my report to the authors and, if the manuscript is accepted for publication, my named report including any attachments I upload will be posted on the website along with the authors' responses. I agree for my report to be made available under an Open Access Creative Commons CC-BY license (http://creativecommons.org/licenses/by/4.0/). I understand that any comments which I do not wish to be included in my named report can be included as confidential comments to the editors, which will not be published.

Choose an item.

To further support our reviewers, we have joined with Publons, where you can gain additional credit to further highlight your hard work (see: https://publons.com/journal/530/gigascience). On publication of this paper, your review will be automatically added to Publons, you can then choose whether or not to claim your Publons credit. I understand this statement.

Yes Choose an item.
