# Supplementary material for: AltaiR: a C toolkit for alignment-free and temporal analysis of multi-FASTA data
Source: Gigascience. 2024 Nov 26;13:giae086. doi: 10.1093/gigascience/giae086 (PMC11590114; doi:10.1093/gigascience/giae086)
Supplement: giae086_Reviewer_2_Report_Revision_1 — MATTEO CHIARA -- 8/5/2024 [file giae086_reviewer_2_report_revision_1.pdf]

Reviewer Report

Title: AltaiR: a C toolkit for alignment-free and temporal analysis of multi-FASTA data

Version: Revision 1 **Date:** 8/5/2024

Reviewer name: MATTEO CHIARA

Reviewer Comments to Author:

Authors have adequately addressed the majority of the points raised by this reviewer in the revised version of the manuscript. However a couple of points still need some clarifications in my opinion.Point 4.1:I understand the point of view of the authors and I apprecciate that a section detailing the execution times for different task performed by Altair was added to the manuscript (Table 2 in particular). However, a computational requirements analysis should also report the amount of resources used, including the amount of RAM memory and disk space to be complete. Could this information be added? I expect that probably Altair will consume only a limited amount of RAM and disk space, but this might be an important information as well for sPoint 4.2:Although I get the point, very stringent quality filters might as well bias the results. Specifically in the case of SARS-CoV-2 these filters might skew the representation of the sequences included in the analyses towards some specific sequencing strategies and/or sequencing centers, and even worse discard some viral variants for which "high quality" genome sequences might not be available.Could the authors please at least assess/demonstrate that the filters applied do not change substantially the composition of the dataset in terms of the viral variants therein represented? A simple table with the proportion of sequences assigned to the 10 most represented viral variants before and after filters are applied could be enough.

Level of Interest

Please indicate how interesting you found the manuscript: Choose an item.

Quality of Written English

Please indicate the quality of language in the manuscript: Choose an item.

Declaration of Competing Interests

Please complete a declaration of competing interests, considering the following questions:

- Have you in the past five years received reimbursements, fees, funding, or salary from an organisation that may in any way gain or lose financially from the publication of this manuscript, either now or in the future?
- Do you hold any stocks or shares in an organisation that may in any way gain or lose financially from the publication of this manuscript, either now or in the future?

- Do you hold or are you currently applying for any patents relating to the content of the manuscript?
- Have you received reimbursements, fees, funding, or salary from an organization that holds or has applied for patents relating to the content of the manuscript?
- Do you have any other financial competing interests?
- Do you have any non-financial competing interests in relation to this paper?

If you can answer no to all of the above, write 'I declare that I have no competing interests' below. If your reply is yes to any, please give details below.

No competing interests

I agree to the open peer review policy of the journal. I understand that my name will be included on my report to the authors and, if the manuscript is accepted for publication, my named report including any attachments I upload will be posted on the website along with the authors' responses. I agree for my report to be made available under an Open Access Creative Commons CC-BY license (http://creativecommons.org/licenses/by/4.0/). I understand that any comments which I do not wish to be included in my named report can be included as confidential comments to the editors, which will not be published.

Choose an item.

To further support our reviewers, we have joined with Publons, where you can gain additional credit to further highlight your hard work (see: https://publons.com/journal/530/gigascience). On publication of this paper, your review will be automatically added to Publons, you can then choose whether or not to claim your Publons credit. I understand this statement.

Yes Choose an item.
